# Proceedings to the 59th Annual Conference of the Particle Therapy Cooperative Group (PTCOG59 2021 Online)

**DOI:** 10.14338/IJPT-22-PTCOG59-9.3

**Published:** 2022-10-25

## Program Description

### Overview

A virtual program with multiple tracks offering original and seminal scientific and educational content presented live and available on-demand to registered participants. Panel discussions covered topical issues including FLASH, Innovative Computational Technologies, and Clinical Research Ethics. Keynote speaker sessions included an overview of the NASA program, research portfolio and the overlap with medicine; adaptive proton therapy; immunotherapy; mini beams / MBRT advanced therapies; and SPARC and hepatocellular carcinoma advanced therapies.

Objectives for the 59^th^ annual conference were to:

- Address individual needs in compliance with their Continuous Professional Development (CPD) plan.
- Discuss the latest technological and clinical innovations in particle therapy.
- Identify educational resources, networks and other for exchange of knowledge and learning about particle therapy,
- Discuss the current research projects within the Particle Therapy Co-Operative Group (PTCOG) and enhance opportunities for future collaboration between groups of young researchers.
- Discuss the latest developments about practical clinical application of particle therapy.
- Describe diagnostics and treatments in the field of particle radiation therapy.

### Target Audience

Healthcare professionals who treat cancer patients using radiation therapy/particle therapy and specifically:

- Radiation Oncologists
- Medical Physicists
- Dosimetrists
- Residents
- Radiation Therapists

### Particle Therapy Cooperative Group (PTCOG) 2021 Committees

***PTCOG Executive and Organizing Committee***

**Jay Flanz, Ph.D., Chairman**

**Tadashi Kamada, M.D., Vice-Chairman**

**Marco Durante, Ph.D., Vice-Chairman**

**Martin Jermann, MSc, Secretary**

**Eugen Hug, M.D., Past PTCOG Chairman**

***PTCOG59 Scientific Program Subcommittee Members***

**Michael Story, Ph.D.**

*Co-Chairman of Scientific Program Subcommittee, Biology*

**Anita Mahajan, M.D.**

*Co-Chairman of Scientific Program Subcommittee, Clinics*

**Antony J. Lomax, Ph.D.**

*Co-Chairman of Scientific Program Subcommittee, Physics*

***PTCOG59 Educational Subcommittee Members***

**James Metz, M.D.**

*Co-Chairman of Education Subcommittee, Clinics*

**Niek Schreuder, M.Sc. DABR**

*Co-Chairman of Education Subcommittee, Physics & Technology*

## Oral Abstracts **O001 -**

### An empirical model of proton RBE based on the linear correlation between proton and photon radiosensitivity

#### David Flint^1^, Scott Bright^1^, David Martinus^1^, Broderick Turner^1^, Mandira Manandhar^1^, Mariam Ben Kacem^1^, Simona Shaitelman^2^, Gabriel Sawakuchi^1^

##### ^1^UT MD Anderson Cancer Center, Radiation Physics, Houston, USA ^2^UT MD Anderson Cancer Center, Radiation Oncology, Houston, USA

**Purpose:** To show that the linear correlation between ion and photon radiosensitivity can be used to model proton RBE.

**Methods:** We performed clonogenic assays to characterize the survival of human cancer cell lines (n=8-11) exposed to 6 MV x-rays and protons with dose-weighted LETs of 1.2, 2.6 and 9.9 keV/μm. We observed that cell radiosensitivity to protons, defined by the dose required to achieve 10% survival, D_10%,protons_, is strongly linearly correlated with D_10%,x-rays_ (r=0.9728-0.9889) (Fig. 1A-C), and that the slope of this correlation varies exponentially with dose-weighted LET (Fig. 1D).This allows a cell's D_10%,protons_ to be expressed as a function of the cell's D_10%,x-rays_, the dose-weighted LET, and three free parameters, c, f and g as follows: D_10%,protons_=c*exp(-f*LET)*D_10%,x-rays_+g. RBE can also be predicted as: RBE_predicted_=D_10%,x-rays_/D_10%,protons,predicted_. Th e same trends hold for other radiosensitivity parameters, e.g. D_50%_ (Fig. 1E-G), which allows α_proton_ and β_proton_ to be predicted by fitting multiple endpoints to the linear quadratic model (Fig. 2). To train our model, we combined our survival data with that in the PIDE database (n=180 LET-cell-line combinations, up to LETs of 37.8 keV/μm) and performed leave-one-out cross-validation to validate the model's predictive accuracy.

**Results:** Our model provided a good fit for the training data (R^2^=0.8929 for D_10%_). It predicted D_10%_ within [-26%,20%] and RBE_D10%_ within [-16%,37%] at the 68.3% confidence level. The whole proton cell survival curves (parameterized by the curves' integrals) were predicted (Fig. 2) within [-26%,20%] at the 68.3% confidence level.

**Conclusions:** Our simple empirical model predicts proton RBE within ∼±25%.

## O002 -

### A quantitative assessment of the role of nuclear reaction processes in the observed boron induced radiosensitization of proton beams

#### Serena Fattori^1^, Francesco Tommasino^2^, Emanuele Scifoni^3^, Giada Petringa^1^, Pablo Cirrone^1^, Lorenzo Manti^4^, Davide Chiappara^5^, Giacomo Cuttone^1^, Andrea Attili^6^

##### ^1^INFN- LNS - Laboratori Nazionali del Sud, Physics, Catania, Italy ^2^Università degli Studi di Trento, Physics, Trento, Italy ^3^INFN- TIFPA - Trento Institute for Fundamental Physics and Applications, Physics, Trento, Italy ^4^Università degli Studi di Napoli - Federico II, Physics, Naples, Italy ^5^Università degli Studi di Padova, Physics, Padova, Italy ^6^INFN Sez. Roma Tre, Physics, Rome, Italy

One of the approaches to gain a tumour-confined increase of RBE by irradiation with a neutron beam is BNCT, which exploits the 10B(n, α)7Li reaction. Recently, another approach exploiting the 11B(p, α)8Be reaction has been investigated using BSH carrier with natural boron content as an enhancement agent in proton-therapy, resulting in a relevant sensitization factor observed on clonogenic survival and chromosome aberrations [Cirrone et al. Sci Rep 2018]. The main aim of this study is the investigation of the radiobiological impact of the secondary particles produced in the nuclear reactions by means of computational modelling. The study exploits a large dataset composed by the latter data and recent clonogenic data for D145 and PANC cells irradiated with protons at LNS and CNAO (SOBPs with max energies in 60-160 MeV) with boron tied in BSH and BPA carriers [Manti et al. Front. Phys, 2021 in prep]. Geant4 Monte Carlo simulations updated with low energy CS data have been implemented to estimate the primary and secondary particle spectra produced by the nuclear reactions [figure1]. The spectra interacting with the cells have been used to evaluate the RBE with different Boron concentrations [figure2]. A modified Microdosimetric Kinetic Model has been specifically designed to account for the breaking of the track-segment condition due to the low-energy components of the secondary spectra. To disentangle the contribution of the different reaction channels, a sensitivity analysis of the RBE as a function of isotope concentration and nuclear cross sections has been performed.

## O003 -

### A comparison of in vivo head and neck cancer response to proton radiotherapy at different segments of the spread-out Bragg-peak

#### Li Wang^1^, Matthew D Kerr^2^, Yupeng Li^2^, Yinhan Zhang^1^, Ming Yang^2^, Xiaodong Zhang^2^, Xiaorong Ronald Zhu^2^, Narayan Sahoo^2^, Steven J. Frank^3^

##### ^1^The University of Texas MD Anderson Cancer Center, Experimental Radiation Oncology, Houston, USA ^2^The University of Texas MD Anderson Cancer Center, Radiation Physics, Houston, USA ^3^The University of Texas MD Anderson Cancer Center, Radiation Oncology, Houston, USA

**Purpose:** Relative biological effectiveness (RBE) of proton (PRT) versus photon radiation is thought to be higher at the distal (PRT-DB) than at the middle (PRT-MB) of the spread-out Bragg-peak (SOBP). The effect of this difference on tumor control is unknown. We investigated this phenomenon in nude mouse human head and neck cancer (HNSCC) xenograft models.

**Methods:** When HNSCC tumors (in hind leg, 6/group) reached 7.3 mm (6.8−8.1 mm for human papillomavirus [HPV]-positive UM-SCC-47), or 8.6 mm (7.3−10.4 mm for HPV-negative Detroit-562) in average-diameter (ave-D), PRT-MB and PRT-DB (2-Gy/day, 5 days/week for 3 [UM-SCC-47] or 2 [Detroit-562] weeks) were initiated. Mice were euthanized when tumors reached 12.0 mm in ave-D, or at 138 days (UM-SCC-47), or at 100 days (Detroit-562). The overall survival times (OSs, days from treatment initiation to mice euthanasia) of mice were compared by a Kaplan-Meier analysis.

**Results:** In mice with UM-SCC-47 tumors, the mean OSs of PRT-MB versus PRT-DB were 61.72±19.87 versus 100.33±14.47 days, median OSs were 26.10±10.29 versus 100.00±0.00 days; PRT-DB (versus PRT-MB) prolonged both mean (1.6 times) and median (3.83 times) OS. In mice with Detroit-562 tumors, the mean OSs of PRT-MB versus PRT-DB were 50.45±14.40 versus 51.33±10.84 days, median OSs were 26.70±7.23 versus 35.00±3.67 days; PRT-DB presented similar mean OS and prolonged median OS (1.31 times) than PRT-MB (**Figure**).

**Conclusion**: PRT-DB (versus PRT-MB) prolonged the OS of mice with HNSCC tumors in a tumor-type-dependent manner. Studies in more tumor types are warranted to validate this finding and to provide further rational for clinical application.

## O004 -

### Radiation Chemistry based analysis of Carbon-FLASH feasibility

#### Daria Boscolo^1^, Martina Fuss^1^, Uli Weber^1^, Michael Krämer^1^, Marco Durante^1^, Emanuele Scifoni^2^

##### ^1^GSI - Helmholtzzentrum für Schwerionenforschung, Biophysics, Darmstadt, Germany ^2^TIFPA-INFN- Istituto Nazionale di Fisica Nucleare, Trento Institute for Fundamental Physics and Applications, Trento, Italy

**Purpose**: Unlike electrons and proton beams, the LET distribution for carbon ion fields ranges over several tens of keV/mum. Thus, a possible FLASH effect and its variability across an irradiated field are contingent on the normal tissue protective mechanism dependence from radiation LET. To date, no published in vivo data on FLASH effects with high LET radiation exists.

**Methods**: We analysed several dependencies of the main mechanisms supposed responsible for a FLASH effect at different LETs in order to search for possible differential effects between tumor and healthy tissues. We considered the impact of oxygenation, different radical production and intertrack recombination. Intertrack was evaluated both at physical (Fig.1) and explicitly on chemical level with TRAX-CHEM [1] MC code for different dose rates. We compared the carbon ion data with similar quantities for equivalent, isorange proton irradiation.

**Results**: The impact of transient induced hypoxia, plays an even smaller effect as compared to low LET radiation (Fig.2), requiring doses above hundreds of Gy to be delivered. In comparison with protons at the same dose, carbon ions show a much smaller absolute number of generated radicals, and a much lower probability to realize intertrack superposition within the timeframe of a chemical stage.

**Conclusions**: All the evaluated mechanistic pathways are indicating a reduced FLASH effect under carbon irradiation conditions. If experimentally demonstrated, the persistence of FLASH effect with C beams could contribute to rule out some candidate pathways considered also for explaining the mechanism at low LET radiation.

## O005 -

### The protective effect of FLASH proton irradiation on the skin relies on oxygen depletion

#### Qixian Zhang^1^, Ethan Cascio^1^, Qiyuan Yang^1^, Leo Gerweck^1^, Peigen Huang^1^, Aimee McNamara^1^, Bernard Gottschalk^1^, Jacob Flanz^1^, Schuemann Jan^1^

##### ^1^Massachusetts General Hospital & Harvard Medical School, Department of Radiation Oncology, Boston, USA

Flash irradiation has received great attention due to its normal tissue sparing effect. Several mechanisms for the normal tissue sparing effect of FLASH irradiation have been proposed. Here, we plan to investigate the protective role of Flash proton irradiation on the skin and verify the impact of oxygen. A double scattering system was established to provide a 1.2X1.6 cm^2^ elliptical field at the dose rate of ∼120 Gy/s with a 228.9 MeV proton beam. Two biologically independent experiments were conducted at the doses of 25 Gy and 27 Gy respectively. The mouse skin was labeled with Indian ink through intracutaneous injection enabling us to detect the skin contraction. The legs of the hypoxic cohorts (Flash-H, CDR-H) were tied to get an anoxic condition. For oxygen-breathing cohorts, the mice were fixed on a holder inside the chamber and were allowed to inhale oxygen for 6 minutes before irradiation and throughout the whole exposure phase (Figure 1). The results indicate that Flash proton irradiation reduced the skin contraction fraction after 27 Gy irradiation. The oxygen breathing procedure eliminated the Flash effect on the skin (Figure 2A). An essentially identical result was obtained at 25 Gy (*P* = 0.0073, Flash vs CDR). The preliminary epidermis thickness at 75 days following 25 Gy exposure suggests a long-term skin protective function of Flash proton irradiation (Figure 2B). The presented results provide evidence that the fast oxygen depletion during irradiation contributes significantly to the Flash effect on normal tissue.

## O006 -

### Experimental validation of LET spectra in mixed radiation fields with the TimePix technology for advanced radiobiological modeling in proton therapy

#### Paulina Stasica^1,2^, Jakub Baran^1^, Carlos Granja^3^, Grzegorz Korcyl^4^, Lukas Marek^3^, Szymon Niedźwiecki^4^, Cristina Oancea^3^, Monika Pawlik-Niedźwiecka^4^, Antoni Ruciński^1^, Marzena Rydygier^5^, Angelo Schiavi^6^, Jan Gajewski^1^

##### ^1^The Henryk Niewodniczanski Institute of Nuclear Physics Polish Academy of Sciences, Proton Radiotherapy Group NZ62, Kraków, Poland ^2^AGH University of Science and Technology, Faculty of Physics and Applied Computer Science, Krakow, Poland ^3^ADVACAM s.r.o., Advacam, Prague, Czech Republic ^4^Jagiellonian University, Faculty of Physics- Astronomy and Applied Computer Science, Krakow, Poland ^5^The Henryk Niewodniczanski Institute of Nuclear Physics Polish Academy of Sciences, Cyclotron Center Bronowice, Kraków, Poland ^6^Sapienza University of Rome, Department of Basic and Applied Sciences for Engineering, Rome, Italy

The assumption of constant RBE in proton radiotherapy neglects the varying nature of linear energy transfer (LET) of protons, hence, causes additional biological dose uncertainty. It is hypothesized that the entire LET spectra in a given point of mixed radiation field is relevant for evaluation of radiobiological effect. We propose an experimental method to measure LET spectra in water for commissioning and validation of variable RBE-based treatment planning. Novel single-quantum sensitive TimePix technology enables to measure energy deposition and track length of individual particles. An experimental setup consisting of a commercial TimePix detector, water phantom and waterproof holder (figure 1) was used for measurements in water. Measurements were performed for various beam energies at many depths and distances from the beam axis. The obtained LET spectra were systematically compared to Gate/Geant4 Monte Carlo simulations. Figure 2a shows an example LET spectrum and dose-averaged LET (LETd) for 150 MeV proton pencil beam obtained from measurements and simulations at the Bragg peak depth and 45 mm away from the beam core. Figure 2b shows results of particle identification for experimental data. We will summarize the results of more than 150 measurements and simulations of LET spectra in water. Timepix allowed for a precise experimental characterization of LET spectra produced by therapeutic proton beams in a given point in water. LETd value is not representative for the LET spectrum, thereby does not seem to be a robust indicator of RBE.

## O007 -

### Proton reirradiation for recurrent or new primary breast cancer in the setting of prior breast irradiation

#### J. Isabelle Choi^1,2^, Atif Khan^2^, Dennis Mah^3^, Beryl McCormick^2^, Alicia Lozano^4^, Henry K. Tsai^3^, Gabriely Del Rosario^3^, Jacqueline Mamary^3^, Haoyang Liu^3^, Pamela Fox^3^, Erin Gillespie^2^, Lior Braunstein^2^, Simon N. Powell^2^, Oren Cahlon^2^

##### ^1^New York Proton Center, Radiation Oncology, New York, USA ^2^Memorial Sloan Kettering Cancer Center, Radiation Oncology, New York, USA ^3^ProCure Proton Therapy Center, Radiation Oncology, New York, USA ^4^Virginia Tech, Department of Statistics, Roanoke, USA

**Purpose**: Proton beam therapy (PBT) reirradiation (reRT) for breast cancer local recurrences and second primaries can optimize normal tissue sparing and may allow safer delivery of a second definitive radiotherapy (RT) course. We analyzed clinical outcomes and toxicities of patients with recurrent or new primary breast cancer who received prior RT and a subsequent course of reRT.

**Methods/Materials**: In an IRB-approved retrospective study, all consecutive patients with recurrent or new primary breast cancer previously treated with breast or chest wall (CW) RT who underwent PBT reRT from a single institution were identified. Patient and tumor characteristics, treatment parameters, outcomes, and toxicities were collected.

**Results**: Forty-six patients were treated with reRT using uniform scanning (70%) or pencil beam scanning (30%) PBT. Median first RT course, reRT, and cumulative doses were 60Gy (range 45-66Gy), 50.4Gy(RBE) (40-66.6Gy), and 110Gy(RBE) (96.6-169.4Gy(RBE)), respectively. Median follow-up was 21 months. There were no local or regional recurrences; 17% developed distant recurrence. Three-year DMFS and OS were 60% and 88%, respectively. Of 13 patients who underwent implant or flap reconstruction, 69% (n=9) developed capsular contracture, 3 of whom required surgical intervention. One patient developed grade 3 breast pain requiring mastectomy. No acute or late grade 4-5 toxicities were seen.

**Conclusion**: In the largest series to date of PBT reRT for breast cancer recurrence or new primary after prior definitive breast or CW RT, excellent locoregional control and low rates of high-grade toxicities were achieved. These data suggest PBT reRT may provide a relatively safe and highly effective salvage option.

## O008 -

### Dosimetry and toxicity profiles of esophageal cancer patients treated with proton radiotherapy: outcomes from the Proton Collaborative Group REG001-09 trial

#### Jacob Parzen^1^, Michael Choung^2^, John Chang^3^, Lane Rosen^4^, James Urbanic^5^, William Hartsell^3^, Henry Tsai^6^, Christopher Sinesi^7^, Jing Zeng^8^, Mark Mishra^9^, Carlos Vargas^10^, Craig Stevens^1^, Peyman Kabolizadeh^1^

##### ^1^Beaumont Proton Therapy Center, Radiation Oncology, Royal Oak, USA ^2^Miami Cancer Institute, Radiation Oncology, Miami, USA ^3^Northwestern Medicine Chicago Proton Center, Radiation Oncology, Warrenville, USA ^4^Willis-Knighton Cancer Center, Radiation Oncology, Shreveport, USA ^5^California Protons Therapy Center, Radiation Oncology, San Diego, USA ^6^Princeton ProCure Proton Therapy Center, Radiation Oncology, Kendall Park, USA ^7^Hampton University Proton Therapy Institute, Radiation Oncology, Hampton, USA ^8^Seattle Cancer Care Alliance Proton Therapy Center, Radiation Oncology, Seattle, USA ^9^Maryland Proton Treatment Center, Radiation Oncology, Baltimore, USA ^10^Mayo Clinic Proton Center, Radiation Oncology, Phoenix, USA

**Background***:* Concurrent chemoradiation plays an integral role in the treatment of esophageal cancer. Proton beam radiotherapy has the potential to spare adjacent critical organs, improving toxicity profiles and potentially improving clinical outcomes.

**Methods***:* We evaluated the Proton Collaborative Group REG001-09 registry for patients undergoing proton radiotherapy for esophageal cancer. Demographic, clinicopathologic, toxicity, and dosimetry information were compiled.

**Results***:* We identified 161 patients treated at 10 institutions between 2010-2019. One hundred twenty-three (76%) had adenocarcinoma and 37 (23%) had squamous cell carcinoma. One hundred forty one (88%) received concurrent chemotherapy. The median delivered dose was 50.51 GyE (range, 18.0-70.1). Grade ≥3 toxicities occurred in 23 (14%) of patients and were most commonly dysphagia (6%), esophagitis (4%), anorexia (4%), and nausea (2%). There were no episodes of grade ≥4 lymphopenia and no grade 5 toxicities. The average mean heart, lung, and liver doses and average maximum spinal cord dose were 10.2 GyE, 4.9 GyE, 3.9 GyE, and 33.4 GyE, respectively. For gastroesophageal junction (GEJ) tumors, 7% of patients developed acute grade ≥3 toxicity and the mean heart, liver, right kidney, and left kidney doses were 10.5 GyE, 3.9 GyE, 0.4 GyE, and 4.9 GyE, respectively. GEJ location was protective against development of grade ≥3 toxicity on univariate (p = 0.0023) and multivariate (p = 0.0079) analysis.

**Conclusions***:* Proton beam radiotherapy affords excellent dosimetric parameters and low toxicity in patients with esophageal cancer treated with curative intent. Prospective trials are underway investigating the comparative benefit of proton-based therapy.

## O009 -

### Proton beam therapy for large hepatocellular carcinomas in Western patients

#### Matthew Greer^1^, Stephanie Schaub^1^, Avril O'Ryan-Blair^2^, Tony Wong^2^, Smith Apisarnthanarax^1,2^

##### ^1^University of Washington, Department of Radiation Oncology, Seattle, USA ^2^Seattle Proton Therapy Center, Seattle Cancer Care Alliance, Seattle, USA

**Purpose/Objectives**: High rates of local control with low risk of hepatotoxicity have been reported in Eastern patients with large hepatocellular carcinomas(HCCs) treated with proton beam therapy (PBT). How generalizable these data are to Western patients is unclear. We sought to characterize the clinical outcomes of Western patients with large HCCs treated with PBT at a single institution.

**Materials/Methods:** Forty-four HCC patients with tumors ≥ 5 cm and ineligible for other liver-directed therapies were treated with PBT between 2014-2019 with a 15-fraction regimen of 45.0-67.5 Gy (RBE). Radiation-induced liver disease (RILD) was defined by a Child-Pugh (CP) score increase of 2 or greater and/or RTOG grade 3 enzyme elevation. Overall survival (OS), progression-free survival (PFS), and local control (LC) were calculated using the Kaplan-Meier method.

**Results**: Patients represented a high-risk cohort with 50% BCLC stage C and 27% CP-B/C. Median gross tumor volume (GTV) size was 11.1 cm (range 5.6-21.5). Median follow-up for survivors was 11 months. 1-year OS and PFS were 53% and 19%, respectively. 1-yr LC was 93%, and crude local failure (LF) rate was 14% (n=6) with one isolated LF. Out of field liver recurrences were the dominant pattern of failure occurring in 61% of patients with disease progression. On univariate analysis, only tumor size was prognostic of OS (HR 1.27, p<0.001). Six patients (14%) experienced RILD; all but one patient had baseline CP-B liver function.

**Conclusions:** In our series of Western HCC patients with high-risk large tumors, PBT results in excellent local control rates and acceptable toxicities.

## O010 -

### Intensity Modulated Proton Therapy for gynecological malignancies: initiation of a program

#### Pranshu Mohindra^1^, Hunter Risher^1^, Ariel Pollock^1^, Mark Zachary^1^, Nrusingh Biswal^1^, Elizabeth M Nichols^1^

##### ^1^University of Maryland School of Medicine and Maryland Proton Treatment Center, Radiation Oncology, Baltimore, USA

**Background**: Dosimetric studies have demonstrated a benefit of proton therapy (PT) over conventional techniques in gynecologic malignancies though supporting clinical data is limited. We initiated our gynecological PT program in 2016.

**Objectives**: To describe our initial patient selection and approach with intensity modulated proton therapy (IMPT).

**Methods**: A retrospective analysis was performed for patients with a diagnosis of gynecologic and female peri-urethral malignancies who underwent IMPT between 02/2016-06/2020. Descriptive analyses were performed.

**Results**: In the study period, IMPT was used in 76 patients with median age of 64.4 years (range, 34-95). Patients were predominantly white (57%) though with a fair proportion of black population (29%). Disease sub-sites were uterine (55%), cervical (21%), vulvar (11%), vaginal (5%), adnexal (7%) malignancies. Nearly 37% of these were primary diagnoses, with IMPT re-irradiation done in 46% of the patients. Systemic therapy, concurrent or sequential, was employed in 54% of the patients. IMPT was planned using both single-field and multiple-field optimization techniques with robustness evaluation for set-up (3-5mm) and range uncertainty (3.5%), KV/CBCT-based image guidance and regular quality assurance CT (QACT) based monitoring. The median planned dose was 50.4 Gy (range 18-70.8 Gy). IMPT target volume included pelvis (59%), abdomen and pelvis (24%), abdomen only (7%) and other body sites (10%). Only 11 of the 76 patients were treated with prone position.

**Conclusions**: We successfully initiated our IMPT program for gynecological cancers, with patient demographics reflecting typical population and re-irradiation being the most common indication. Ongoing outcomes analysis have led to initiation of a prospective study (NCT04527900).

## O011 -

### Proton therapy for high risk prostate cancer: Results from the Proton Collaborative Group PCG 001-09 prospective registry trial

#### Shaakir Hasan^1^, Stanslav Lazarev^2^, Madhur Garg^3^, John Chang^4^, William Hartsell^5^, Jonathan Chen^6^, Henry Tsai^7^, Carlos Vargas^8^, Mark Mishra^9^, Lane Rosen^10^, Charles Simone^1^, Daniel GOrovets^11^

##### ^1^New York Proton Center, Radiation Oncology, New York, USA ^2^Mount Sinai Medical Center, Radiation Oncology, New York, USA ^3^Montefiore Medical Center, Radiation Oncology, New York, USA ^4^Oklahoma Proton Center, Radiation Oncology, Oklahoma City, USA ^5^Northwestern University, Radiation Oncology, Chicago, USA ^6^University of Washington, Radiation Oncology, Seattle, USA ^7^ProCure New Jersey, Radiation Oncology, Somserset, USA ^8^Mayo Clinic, Radiation Oncology, Phoenix, USA ^9^University of Maryland, Radiation Oncology, Baltimore, USA ^10^Willis Knighton Medical Center, Radiation Oncology, Shrieveport, USA ^11^Memorial Sloan Kettering Cancer Center, Radiation Oncology, New York, USA

**Introduction**: Using the Proton Collaborative Group (PCG) prospective registry, we report outcomes for high risk prostate cancer (HRPC) treated with proton therapy.

**Methods**: After exclusion, 605 HRPC patients from 8/2009-3/2019 at nine institutions were analyzed for freedom from progression (FFP), metastasis free survival (MFS), overall survival (OS), and toxicity. Multivariable cox/binomial regression models were used to assess for predictors of FFP and toxicity.

**Results**: [CS1] Median age was 71 years. Gleason grade groups 4 (49.4%) and 5 (31.7%) were most common, as were stage T1c (46.1%) and T2 (41.3%). The median pre-treatment prostate specific antigen was 9.18. Median dose was 79.2 GyE in 44 fractions. Pelvic lymph nodes were treated in 58.2% of cases and 63.6% of patients received androgen deprivation therapy. Pencil beam scanning was used in 54.5%, uniform scanning in 38.8%, and a rectal spacer in 14.2%. At a median follow-up of 2 years, the 3- and 5-year FFP were 90.7% and 81.4%, respectively. The 5-year MFS and OS were 92.8% and 95.9%, respectively. Independent correlates of FFP included Gleason ≥8, PSA >10, and cT2 (all P<0.05). There were no grade 4 or 5 adverse events. Late grade 2 and 3 genitourinary toxicity was 5.8% and 1.7%, respectively, while late grade 2 and 3 gastrointestinal toxicity was 5% and 0%. Grade 2 and 3 erectile dysfunction at 2 years was 48.4% and 8.4%, respectively.

**Conclusion**: In the largest series published to date, our results suggest that early safety and efficacy outcomes using proton therapy for HRPC are encouraging.

## O012 -

### A feasibility study of focal dose escalations to multiparametric MRI-defined dominant intraprostatic lesions using intensity-modulated proton therapy

#### Jun Zhou^1^, Tonghe Wang^1^, Sibo Tian^1^, Pretesh Patel^1^, Ashesh B. Jani^1^, Sagar Patel^1^, Katja Langen^1^, Yinan Wang^1^, Mark W. McDonald^1^, Jeffrey Bradley^1^, Tian Liu^1^, Xiaofeng Yang^1^

##### ^1^Emory University, Radiation Oncology, Atlanta, USA

**Purpose:** The purpose of this study is to investigate the dosimetric feasibility and clinical impact of delivering a focal radiotherapy boost to multiparametric MRI (mp-MRI)-defined prostate dominant intraprostatic lesions (DILs) using proton therapy.

**Methods:** We retrospectively investigated 36 patients with pre-treatment mp-MRIs and CTs who received whole prostate pencil beam scanning (PBS) proton radiotherapy. DILs were delineated on co-registered mp-MRIs. Simultaneous integrated boost (SIB) plans using intensity-modulated proton therapy (IMPT) were created based on conventional whole-prostate-irradiation plans and optimized with additional DIL coverage goals and organ-at-risk (OAR) constraints. Both conventional and SIB plans were evaluated for DIL coverage and OAR sparing. Tumor control probability (TCP) and normal tissue complication probability (NTCP) were estimated to evaluate the clinical impact of DIL boosts.

**Results:** SIB plans were able to achieve >95% coverage by V125%, V150% and V175% of the prescription dose (70 Gy in 28 fractions) in 74%, 54% and 17% of patients, respectively. This is expected to significantly increase DIL TCP by 7.3-13.3% (p < 0.001) depending on α/β ratio and DIL risk levels. SIB plans did not significantly differ with conventional plans in terms of the bladder and rectal NTCP. SIB plans showed 2.3% and 0.6% higher NTCP for urethral and femoral heads.

**Conclusion:** Mp-MRI-guided DIL boost using proton radiotherapy is feasible without violating OAR constraints, and may translate into a clinically significant improvement in DIL TCP. IMPT-based DIL SIB may represent a strategy to improve tumor control.

## O013 -

### Determinant role of the immune system in the anti-tumoral response to proton minibeam radiation therapy (pMBRT)

#### Annaïg Bertho^1^, Elise Brisebard^2^, Marjorie Juchaux^1^, Cristèle Gilbert^1^, Charlotte Lamirault^3^, Annalisa Patriarca^4^, Ludovic De Marzi^4^, Frederic Pouzoulet^3^, Yolanda Prezado^1^

##### ^1^Institut Curie, U1021/umr3347, Orsay, France ^2^Oniris, UMR703 PAnther APEX, Nantes, France ^3^Institut Curie, Experimental Radiotherapy Platform, Orsay, France ^4^Institut Curie - Centre de Protontherapie, Oncological radiotherapy department, Orsay, France

Proton mini beam radiation therapy (pMBRT) is a novel therapeutic approach which uses spatially modulated narrow (< 1 mm) proton beams [1]. pMBRT has shown a remarkable reduction of neurotoxicity [2] and an equivalent or superior tumor control than conventional irradiations in small animals [3], and that with highly heterogeneous dose distributions. The dose distribution consists in high (peak) doses in the path of the beam and low (valley) doses in between them. The underlying biological mechanism under pMBRT efficiency remain elusive. Improving the radiobiological knowledge pertaining to these points could provide a better understanding of the mechanisms involved in pMBRT response. We collected original *in vivo* radiopathological data in rats, thanks to the implementation of pMBRT at the Institut Curie Protontherapy Center. The aim of this work was to investigate the possible participation of the immune system in the anti-tumor response to pMBRT. We compare the response of RG2-bearing nude (athymic) rats versus immunocompetent (fischer) rats. The dose prescription was 30 Gy (average) in one fraction, with approximately one third of the tumor volume receiving (valley) doses of less than 10 Gy. While a significant increase of lifespan was observed in the irradiated immunocompetent rats with respect non-irradiated controls, the irradiated nude rats showed no response to the treatment (Figure 1). Immunochemistry evaluations to assess immune cell infiltration and tumor morphology after irradiation are ongoing. These results suggest that immune activation plays a central role in indirect tumor cell kill in response to pMBRT.

## O014 -

### Carbon ions in combination with immune checkpoint inhibitors reduce the number of lung metastases in a mouse osteosarcoma model

#### Alexander Helm^1^, Walter Tinganelli^1^, Palma Simoniello^2^, Claudia Fournier^1^, Takashi Shimokawa^3^, Marco Durante^1^

##### ^1^GSI Helmholtz Center for Heavy Ion Research, Biophysics, Darmstadt, Germany ^2^Parthenope University of Naples, Department of Science and Technology, Naples, Italy ^3^National Institute of Radiological Sciences- National Institutes of Quantum and Radiological Science and Technology, National Institute of Radiological Sciences, Chiba, Japan

The combination of radiotherapy and immunotherapy is recognized as a very promising strategy for metastatic cancer treatment. Our purpose was to compare the effectiveness of X-ray and high-energy carbon ion therapy in combination with checkpoint inhibitors in a murine model. We used an osteosarcoma mouse model irradiated with either carbon ions or X-rays in combination with anti-PD-1 and anti-CTLA-4. LM8 osteosarcoma cells were injected in both hind limbs of C3H/He mice 7 days prior to exposure to carbon ions or X-rays. In experimental groups receiving irradiation, only the tumor on the left limb was exposed, whereas the tumor on the right limb served as an abscopal mimic. Checkpoint inhibitors were injected 1 day before exposure as well as concomitant to and after exposure. Tumor growth was measured regularly up to day 21 after exposure. Tumors and lungs were extracted. A reduced growth of the abscopal tumor was most pronounced after the combined protocol of carbon ions and the immune checkpoint inhibitors. Radiation or checkpoint inhibitors alone were insufficient to reduce the growth of the abscopal tumors. Carbon ions alone reduced the number of lung metastases more efficiently than X-rays, and in combination with immunotherapy both radiation types essentially suppressed the metastases, with carbon ions being again more efficient. Investigation of the infiltration of immune cells in the abscopal tumors of animals treated with combination revealed an increase in CD8+ cells. The combination of checkpoint inhibitors with carbon ion radiotherapy can be an effective strategy for the treatment of advanced tumors.

## O015 -

### Proton-FLASH – Radiation effects of ultrahigh dose-rate irradiation

#### Sarah Rudigkeit^1^, Matejka Nicole^1^, Ce-Belle Chen^2^, Annique Dombrowsky^3^, Matthias Sammer^1^, Thomas Schmid^3^, Günther Dollinger^1^, Judith Reindl^1^

##### ^1^Institut für angewandte Physik und Messtechnik- Fakultät für Luft- und Raumfahrttechnik, Universität der Bundeswehr München, Neubiberg, Germany ^2^National University of Singapore, Center for Ion Beam Applications- Department of Physics, Singapore, Singapore ^3^Helmholtz Zentrum München, Institute of innovative Radiotherapy, Neuherberg, Germany

The application of radiation with ultra-high dose-rates in radiotherapy shows a sparing effect of healthy tissue compared to cancerous tissue. This so-called FLASH-effect is mainly studied by using electrons or x-rays. Radiotherapy using protons already shows benefits in the low dose-rate application compared to conventional treatment. Therefore, a combination of both the particle based sparing and the FLASH effect could further widen the therapeutic window. Here, we investigated the FLASH effect in proton treatment using an in-vivo mouse ear model. For the experiment the right ears of 63 Balb/c mice were irradiated with 20 MeV protons at the ion microprobe SNAKE at the 14 MV tandem accelerator in Garching near Munich by using three dose- rates (3.7 Gy/min, 558 Gy/min and 55,800 Gy/min). Additionally we compared the FLASH-effect at 23 Gy and 33 Gy. For quantification we measured the ear thickness, desquamation and erythema for 180 days. No difference in the 23 Gy group for the different dose-rates was visible, whereas for the 33 Gy group it was significant. For 558 Gy/min we received a 57 % reduction of ear swelling and a 40 % reduction for 55,800 Gy/min compared to the conventional dose-rate of 3.7 Gy/min. Desquamation and erythema were reduced by 68 % and 50 %. By using FLASH-dose-rates for low LET proton irradiation a tissue sparing effect can be achieved. This effect seems to be more significant with increased dose and was also observed at a dose-rate four times smaller than usually used FLASH-dose-rates (≥ 2400 Gy/min).

## O016 -

### Comparative response of head and neck cancer cells to photons and protons: targeting the DNA damage response to optimise radiosensitivity

#### Maria Rita Fabbrizi^1^, Rachel Carter^1^, Eirini Terpsi Vitti^1^, Catherine Nickson^1^, Andrzej Kacperek^2^, Mark Hill^3^, Jason Parsons^1^

##### ^1^University of Liverpool, Department of Molecular and Clinical Cancer Medicine, Liverpool, United Kingdom ^2^Clatterbridge Cancer Centre NHS Foundation Trust, The National Centre for Eye Proton Therapy, Bebington, United Kingdom ^3^University of Oxford, CRUK/MRC Oxford Institute for Radiation Oncology, Oxford, United Kingdom

Radiotherapy is a mainstay for treatment of head and neck squamous cell carcinoma (HNSCC), and where proton beam therapy (PBT) is increasingly being utilised as a precision targeted approach. However, PBT displays increases in linear energy transfer (LET) at and around the Bragg peak leading to complex DNA damage (CDD), where several lesions are induced in close proximity within DNA, which contributes significantly to enhanced biological effectiveness. Consequently, there are still uncertainties regarding the optimal strategies using PBT and the comparisons to photon radiotherapy in tumour models such as HNSCC. Utilising the 60 MeV cyclotron at the Clatterbridge Cancer Centre, we have comparatively analysed the response of HNSCC cell lines to low-LET (58 MeV, 1 keV/μm) protons at entrance dose, versus high-LET (11 MeV, 12 keV/μm) protons generated at the Bragg peak distal end, in addition to low-LET x-rays (100 kV). We have demonstrated that high-LET protons induce significant decreases in cell survival post-irradiation versus low-LET protons/photons due to elevated levels of CDD. We have also identified specific enzymes within the cellular DNA damage response that when targeted using siRNA/inhibitors can further exacerbate the HNSCC cell killing effects under the different irradiation conditions. Indeed, inhibition of the DNA single strand break sensor PARP-1 or the DNA glycosylase enzyme OGG1 can enhance cellular radiosensitivity specifically to high-LET protons. In contrast, inhibitors against the DNA double strand break protein kinases ATM, ATR and DNA-Pkcs are effective in reducing the survival of both 2D and 3D HNSCC models to low-LET protons and photons.

## O017 -

### Does oxygen depletion occur in FLASH-RT? Experimental evaluation for photons, protons and carbon ions

#### Jeannette Jansen^1,2^, Jan Knoll^1,2^, Elke Beyreuther^3,4^, Jörg Pawelke^3,5^, Raphael Skuza^1,2^, Rachel Hanley^1,2^, Stephan Brons^6^, Francesca Pagliari^1^, Joao Seco^1,2^

##### ^1^German Cancer Research Center DKFZ, Biomedical Physics in Radiation Oncology E041, Heidelberg, Germany ^2^Ruprecht-Karls-Universität Heidelberg, Faculty of Physics and Astronomy, Heidelberg, Germany ^3^OncoRay - National Center for Radiation Research in Oncology, Faculty of Medicine and University Hospital Carl Gustav Carus- Technische Universität Dresden- Helmholtz-Zentrum- Dresden- Rossendorf, Dresden, Germany ^4^Helmholtz-Zentrum Dresden - Rossendorf, Institute of Radiation Physics, Dresden, Germany ^5^Helmholtz-Zentrum Dresden - Rossendorf, Institute of Radiooncology - OncoRay, Dresden, Germany ^6^Heidelberg Ion-Beam Therapy Center HIT- Heidelberg University Hospital, Department of Radiation Oncology, Heidelberg, Germany

The investigation of FLASH radiotherapy (RT), i.e. the irradiation with high dose rates of 40 Gy/s or more, has gained increasing interest in the past years. It has been observed in vivo, that the same amount of dose delivered by FLASH-RT causes less healthy tissue damage while tumor control probability remains comparable to conventional RT[1,2]. This phenomenon is referred to as FLASH effect. The mechanism behind the FLASH effect is still unknown but various mathematical models suggest a potential contribution of oxygen. Our study presented here measured oxygen depletion on-line in sealed water phantoms during photon, proton and carbon irradiation. The results of our study suggest that oxygen gets consumed during radiation, but the amount of oxygen depleted per dose is by far too small to explain the FLASH effect by radiation-induced hypoxia alone. Moreover, we found that less oxygen was consumed at higher dose rates for all investigated radiation conditions, i.e. photon, proton and carbon ion beams. Hence, we can exclude the oxygen depletion hypothesis as driving factor behind the FLASH mechanism. Instead, the presented results hint towards self-interaction of radicals leading to less solvated electrons e_aq present in the water. [3]

## O018 -

### The impact of cardiac sparing on long-term pulmonary vascular recovery in proton-irradiated rats

#### Julia Wiedemann^1^, Sai K. Paruchuru^1^, Lisette E. den Boef1^1^, Uilke Brouwer^2^, Elisabeth M. Schouten^3^, Michael G. Dickinson^3^, Christina T. Muijs^1^, Rudolf A. de Boer^3^, Robert P. Coppes^2^, Peter van Luijk^1^

##### ^1^University Medical Center Groningen, Department of Radiation Oncology, Groningen, Netherlands ^2^University Medical Center Groningen, Department of Biomedical Sciences of Cells and System- Section Molecular Cell Biology-, Groningen, Netherlands ^3^University Medical Center Groningen, Department of Cardiology, Groningen, Netherlands

Compared to photon irradiation, proton therapy can facilitate and improve sparing of specific organs. In a rat model of lung irradiation, loss of cardiopulmonary function 8 weeks after irradiation is aggravated if the heart is co-irradiated. Therefore, active sparing of the heart could reduce severity of cardiopulmonary side effects after thoracic radiotherapy. Here, we tested long-term benefit of cardiac sparing on cardiopulmonary side effects. Using 150 MeV protons, 50% of the rat lung was irradiated with or without heart co-irradiation. Significant increases in respiratory rate were observed 8-12 and 18-24 weeks after irradiation of the lung, while a significant recovery was observed at later times (36-42 weeks, Figure 1A). Such recovery did not occur after co-irradiation of heart and lung. In line with this, vascular remodeling assessed from Verhoeff-stained lung sections (Figure1B) only showed recovery if the heart was spared (Figure 1C). Lack of recovery of right ventricle contraction on echocardiography as an overall measure of pulmonary vascular resistance if the heart was co-irradiated confirmed this finding (Figure 1D). Although recovery of pulmonary vasculature and therefore of cardiopulmonary function is possible, this requires effective heart-sparing. These results indicate that sparing of the heart is important to preserve the potential to recover from early cardiopulmonary side effects. As such, use of proton irradiation for treatment of thoracic cancers to improve heart-sparing may potentially improve clinical outcome.

## O019 -

### MRI-based tumour localization after tantalum clip placement for proton beam therapy planning of uveal melanoma

#### Myriam Jaarsma-Coes^1^, Teresa Ferreira^2^, Marina Marinkovic^3^, Khanh Vu^3^, Myra Rodriques^4^, Yvonne Klaver^4^, Berit Verbist^2^, Gregorius Luyten^3^, Coen Rasch^5^, Jan-Willem Beenakker^1^

##### ^1^Leiden University Medical Center, Radiology & Ophthalmology, Leiden, Netherlands ^2^Leiden University Medical Center, Radiology, Leiden, Netherlands ^3^Leiden University Medical Center, Ophthalmology, Leiden, Netherlands ^4^HollandPTC, Radiotherapy, Delft, Netherlands ^5^Leiden University Medical Center, Radiotherapy, Leiden, Netherlands

**Purpose**: In ocular proton beam therapy (PBT), 2.5mm tantalum clips are surgically sutured near the tumour border. The clip-tumour distances are assessed peroperative for CTV marking. To support the clip localisation, target deliniation and 3D tumour modelling, a dedicated MRI-protocol was developed and evaluated.

**Methods**: Twenty-three consecutive uveal melanoma (UM) patients, planned for PBT, were scanned on a 3T MRI. Clip artefacts were minimized by localized shimming and increased gradient strengths. Clip-tumour distances were measured and compared with the peroperative measurements. For clips with more than 1mm difference, the most likely origin of this discrepancy was discussed in a multidisciplinary setting.

**Results**: In 55% of the 87 evaluated clips, MRI and peroperative measurements differed less than 1mm, fig 1. Largest differences were observed in flat UM, where MRI was generally considered less reliable. In 15% of the clips, the discrepancy was attributed to a complex tumour geometry, fig 2AB. In 10% of the clips, the anterior localized tumour casted a shadow, resulting in an overestimation of the tumour dimensions during surgery, fig 2C. For these patients, MR-imaging was considered more reliable.

**Conclusion**: MRI and peroperative measurements are of equal value in most cases. For flat UM, MRI underestimates tumour dimensions. For complex and anterior UM, MRI-based clip-tumour measurements are more reliable, which could result in more accurate target definition, thereby possibly reducing toxicity and improving probability to retain vision.

## O020 -

### Toxicity analysis of reirradiation with proton therapy for central nervous system tumors: a prospective proton collaborative group study

#### Shaakir Hasan^1^, Robert Press^1^, J Isabelle Choi^1^, George Laramore^2^, Henry Tsai^3^, Mark Mishra^4^, John Chang^5^, Carlos Vargas^6^, Sujay Vora^6^, Vinai Gondi^7^, Charles Simone^1^

##### ^1^New York Proton Center, Radiation Oncology, New York, USA ^2^University of Washington, Radiation Oncology, Seattle, USA ^3^ProCure Proton Therapy Center, Radiation Oncology, Somerset, USA ^4^University of Maryland, Radiation Oncology, Baltimore, USA ^5^Oklahoma Proton Center, Radiation Oncology, Oklahoma City, USA ^6^Mayo Clinic, Radiation Oncology, Phoenix, USA ^7^Northwestern University, Radiation Oncology, Chicago, USA

**Introduction**: This is an expanded analysis of the acute toxicities of reirradiation with proton therapy (PT) for central nervous system (CNS) tumors using the Proton Collaborative Group (PCG).

**Methods**: The multi-institutional, prospectively collected PCG registry was queried for consecutive patients with CNS tumors treated with PT reirradiation between 2010-2020. Acute grade ≥2 toxicities were reported, with binomial regression analysis to identify correlates thereof.

**Results**: Overall, 176 patients (97 male, 79 female) 19-85 years old (median 49) were identified, with 117 gliomas, 37 benign tumors, and 22 medulloblastoma/ependymomas/neuroendocrine tumors located in cerebral hemispheres (n=130), infratentorium (n=24), base of the skull (n=14), and spinal cord (n=8). Median time to PT reirradiation was 63 months. Median PT dose and cumulative dose (EQD2) were 50 Gy_10_ (13 – 66 Gy) and 104 Gy_10_ (51-210 Gy_10_), respectively. Chemotherapy was given concurrently in 86 patients. Baseline ECOG was 0(n=55), 1(n=56), ≥2(n=42). Median follow-up was 10 months. Acute Grade 2 and Grade 3 toxicities occurred in 51.1% and 7.9% of patients, respectively. Eighteen patients had G3 symptoms at baseline, and all but one of which resolved after PT. There were no grade 4 or 5 toxicities. Independent correlates of Grade 3 toxicity per multivariable binomial regression analysis include ECOG ≥2 (HR=18.7, P=0.003) and cumulative EQD2 dose over 115 Gy (HR=4.7, P =0.03).

**Conclusion**: In the largest analysis to date, proton reirradiation in appropriately selected patients with CNS tumors is well tolerated. Poor performance status and higher cumulative radiation doses in the salvage setting correlated with developing Grade 3 toxicities

## O021 -

### Pitfalls in ocular treatment planning involving CT, MRI and/or Fundus imaging

#### Jens Heufelder^1,2^, Dino Cordini^1,2^, Sophie Seidel^3^, Roland Stark^1,2^, Andreas Weber^1,2^, Antonia M Joussen^2^, Johannes Gollrad^4^

##### ^1^Charité - Universitätsmedizin Berlin, BerlinProtonen am HZB, Berlin, Germany ^2^Charité - Universitätsmedizin Berlin, Department of Ophthalmology, Berlin, Germany ^3^Helmholtz-Zentrum Berlin für Materialien und Energie, Proton therapy, Berlin, Germany ^4^Charité - Universitätsmedizin Berlin, Department of Radiation Oncology and Radiotherapy, Berlin, Germany

The new CE-certified treatment planning system RayOcular enables the utilization of computer tomography (CT) and magnetic resonance imaging (MRI) additionally to the standard fundus imaging in ocular proton therapy (OPT) treatment planning. Since 2006, more than 3062 individual treatment plans were created using our in-house treatment planning system OCTOPUS based on CT and fundus imaging. In 1078 of those cases, the patient received additional MRI for treatment planning. In our experience, there is no Gold standard imaging for the treatment planning in OPT. Each imaging modality has its pros, cons and sometimes pitfalls. CT is a good tool creating an eye model and measuring the position of the tantalum clips. The fundus image is used to define the base of the clinical target volume (CTV). Though this could be difficult for amelanotic or very big tumors. In some cases, the registration of the fundus image to the eye model based on clip positions, macula and optic disc can be difficult. At first glance, MRI seems to be the optimal tool for eye modeling and tumor delineation, but its resolution is too low for small tumors. Typical Voxel sizes are 0.5×0.5×0.5 mm^3^ or 0.4×0.4×1.0 mm^3^. This is not sufficient to detect small or flat tumor extensions with a height of less than 1 mm. To overcome the problems, we use a combination of CT, fundus imaging and MRI along with additional verifications on the based on ultrasound imaging and ocular coherence tomography (OCT). Cases and details will be shown in the presentation.

## O022 -

### Long-term clinical outcomes with dedicated proton ocular beam and helium ion radiation for uveal melanoma patients aged 45 and younger

#### Carly Zako^1^, Jessica Scholey^1^, Inder Daftari^1^, Vivian Weinberg^1^, Carisa Swason^2^, Jeanne Quivey^1^, Devron Char^2^, Kavita Mishra^1^

##### ^1^University of California San Francisco, Radiation Oncology, San Francisco, USA ^2^The Tumori Foundation, Ocular Oncology, San Francisco, USA

**Purpose:** Uveal melanoma (UM) is particularly rare and potentially devastating for patients <45 years old (yo). Long-term clinical outcomes are presented.

**Methods:** A single institution's prospectively maintained database (n=2558) used to identify UM patients <45 yo at proton beam radiation (PBRT, n=247, 1994-2020) and helium RT (n=80, 1979-1992). PBRT patients undergoing 56 GyE (n=240) analyzed with univariate Kaplan-Meier and multivariate Cox's proportional hazard.

**Results:** Median follow-up PBRT pts 85.7 months (3.0-317.5). 5-year (y) local control (LC) 95% and 10y 94%. Ciliary body (CB) tumors showed lower LC (p = 0.02). Largest tumor diameter was the LC independent multivariate predictor (p = 0.04). Three patients with very late local failures (LF) showed spindle-cell features [time-to-LF 13.2, 16.1, 17.5 years]. 10y eye-preservation 83%. Enucleation risk decreased with greater tumor-disc distance (p=0.0001). Improved eye-preservation with <50% dose to lens (p=0.01), <20% CB (p=0.02), disc (p=0.004), macula (p=0.03), and nerve (p=0.009). Multivariate identified nerve length receiving 50% dose (p=0.0003) and tumor height (p=0.0008) as significant independent predictors of enucleation. 10y Distant metastasis (DM)-free rate 81%; 10y overall survival 83%. LTD most significant DM predictor (p =0.0001) followed by age >30 vs <30 (p=0.03). Age<30 (n=44) had only 2% CB tumors, 5y DM-free 100%. Two of 7 early (1994-95) proton patients receiving 48 GyE, had LF. Helium cohort median follow-up 160.9 months (23.9-292.8); median dose 70 GyE (range 48-80); 10y LC 99%, 10y Eye preservation 82%.

**Conclusion:** Excellent LC (10y 94% PBRT, 99% Helium) and eye preservation long-term in UM patients aged <45. Clinical/dose-volume parameters may be utilized to assess prognosis.

## O023 -

### Protontherapy for ocular melanoma with active scanning beam at CNAO (National Center Oncological Hadrontherapy) and National Cancer Institute of Milan

#### Maria Rosaria Fiore^1^, Martina Angi^2^, Francesco Baldo Lanza^2^, Edoardo Mastella^3^, Andrea Pella^4^, Guido Baroni^4^, Davide Davide Maestri^3^, Jessica Sergenti^2^, Rachele Antonini^2^, Agnieszka Chalaszczyk^1^, Alessandro Cicchetti^2^, Ester Orlandi^1^, Mario Ciocca^3^

##### ^1^National center of hadrontherapy CNAO, Radiotherapy department, Pavia, Italy ^2^Fondazione IRCCS Istituto Nazionale Tumori, Ocular oncology service, Milan, Italy ^3^National center of hadrontherapy CNAO, Physics department, Pavia, Italy ^4^National center of hadrontherapy CNAO, Bioengineering department, Pavia, Italy

**Introduction**: Proton beam radiotherapy for ocular melanoma (OM) is delivered worldwide with a dedicated beamline and passive scattering modality. At CNAO proton and carbon ion beams accelerated by variable energy synchrotron are delivered with active scanning modality. Design and commissioning of the first non-dedicated ocular beamline, based on pencil beam scanning, was realized according to technical requirements of ocular treatment. The aim of this study was to present preliminary results of scanning and collimated proton beams under single anterior field geometry in the ocular clinical practice.

**Methods**: Between March 2018 and April 2020, 78 patients with OM were treated at the CNAO, using scanning collimated proton beams. Patients and tumors characteristics in table 1. All patients underwent surgical placement of tantalum clips defining tumor location. Straight-ahead Computed Tomography (CT) scan of the ocular region was acquired in supine position for clips localization and eye model verification.Target was defined using clips and high-resolution fundus photograph. A custom eye tracking system with fixation light was used to monitor patient gaze direction.Patients were treated on a dedicated chair using one anterior field. The total dose was 60 Gy (RBE) in four consecutive daily sessions.

**Results**: With a median follow-up of 12.4 months (range 1-27) two local failures occurred (2.6%). All 78 patients developed acceptable toxicity.

**Conclusions**: All patients successfully completed the treatment course without any unexpected severe toxicity.Patients accrual is still ongoing and follow-up is being extended to confirm promising local tumor control with acceptable toxicity.

## O024 -

### On the necessity of extended DRR/Kv images for CSI setup accuracy and efficiency

#### Chengyu Shi^1^, Weijun Xiong^1^, Qing chen^1^, Minglei Kang^1^, Suzanne Wolden^2^, Jonathan Yang^2^, Charles Simone^1^, Haibo Lin^1^

##### ^1^New York Proton Center, Medical Physics, New York, USA ^2^Memorial Sloan Kettering Cancer Center, Radiation Oncology, New York, USA

Proton radiation therapy provides significant dosimetry advantages over photon for Cranial-Spinal Irradiation (CSI) by sparing the normal organs in the thorax and abdomen regions. However, the reproducibility of the patient positioning during treatment especially at the field junction are the major clinical concerns. The traditional IGRT alignment depends on the paired kV images and shifts among brain, upper spine, and lower spine targets. Deviations in alignment exist due to shifts and limited field of view of kV images. Repeating imaging and fine adjustment may be needed to minimize the setup uncertainties, which not only takes time but also increases the imaging dose to the patients. We proposed a new virtual imaging technique called extended DRR/kV, which will utilize pair kV images from the patient's different isocenter and fuse the kV images into extended kV images. We can align the patient with the extended DRR/kV. Matlab program was developed to process the exported DRR and kV DICOM images. Automatic rigid image registration was used to align the extended DRR with the kV images. Structural SIMilarity index (SSIM) was used to compare the traditional alignment vs. the proposed extended DRR/kV alignment. For the test patient, SSIM results show the proposed method has averaged SSIM 0.522±0.033 vs. 0.491±0.008 and 0.717±0.114 vs. 0.564±0.015 based on AP and LAT images for all the fractions (n=20) of the treatment images respectively. Thus, preliminary results show the extended DRR/kV method with auto-alignment implementation for the CSI patient alignment is superior to the current setup procedure.

## O025 -

### Photon and proton therapy outcome assessment with dose painting prescription based on an in silico oxygen distribution model

#### Ana Ureba^1^, Jakob Öden^2^, Iuliana Toma-Dasu^1,3^, Lazzeroni Marta^1,3^

##### ^1^Karolinska Institute, Medical Radiation Physics, Stockholm, Sweden ^2^Raysearch Laboratories, Research Department, Stockholm, Sweden ^3^Stockholm University, Medical Radiation Physics, Stockholm, Sweden

Tumour hypoxia may reduce tumour control because it induces radioresistance. PET images can be used for the identification of hypoxic volumes and guide dose painting strategies (DPS) for treatment planning. The aim of the study is to determine the clinical feasibility of two DPS accounting for the tumour oxygen distribution. An *in silico* model for tumour oxygenation at sub-millimetre scale was used for simulating a lung tumour and its radiosensitivity in a whole-body phantom. The PET image of the tumour was simulated and used as input for DPS. Two different dose distributions aiming at 95% TCP were created: by contour (DPBC) with two levels of uniform dose, in the hypoxic target volume (HTV) and in the rim between the GTV and the HTV (GTV-HTV); and by box (DPBOX) consisting of dividing the GTV in 10mm side cubic sub-volumes and assigning the maximum dose within each box. The photon and proton planning was made in RayStation v10 (RaySearch Laboratories) using minimax robust optimisation considering ±5mm setup errors for all plans and 0% and ±3.5% density change for proton plans. Figure 1 shows the prescribed and the nominal dose distributions of both DPS. Figure 2 shows the lower and upper bounds (1 sigma) of the robust evaluation in terms of DVHs and TCP. The TCP was >89% for DPBC and >95% for the DPBOX in the GTV. In conclusion, both DPS demonstrated robust target coverage, although the DPBOX outperforms the DPBC with respect to TCP in all cases regardless the beam quality.

## O026 -

### Combined fixed beam line proton and photon therapy for lung cancer treatments

#### Florian Amstutz^1,2^, Silvia Fabiano^3^, Louise Marc^3^, Damien C Weber^1,3,4^, Antony J Lomax^1,2^, Jan Unkelbach^3^, Ye Zhang^1^

##### ^1^Paul Scherrer Institute, Center for Proton Therapy, Villigen PSI, Switzerland ^2^ETH Zurich, Department of Physics, Zurich, Switzerland ^3^University Hospital Zurich, Department of Radiation Oncology, Zurich, Switzerland ^4^University Hospital Bern, Department of Radiation Oncology, Bern, Switzerland

**Purpose:** Proton therapy is still a scarce resource. By optimally combining proton and photon treatments (CPPT) however, increased accessibility to proton therapy could be achieved. Potential dosimetric benefits of adding a fixed horizontal proton beam line to a conventional photon treatment room is here investigated for non-small-cell lung cancer (NSCLC) patients treated in deep inspiration breath-hold (DIBH).

**Methods:** Treatment plans to 70 Gy-RBE were calculated for seven NSCLC patients on voluntary DIBH CTs. 9-field IMRT and simultaneously optimized CPPT plans (allowing for non-homogenous contributions from both modalities) were calculated. For CPPT, 9-field IMRT was combined with two proton fields assumed to be delivered from a fixed horizontal beam line brought into the photon vault. To study effects of anatomical changes, re-planning was performed on repeated DIBH CTs, acquired regularly through the treatment course, thus simulating an adaptive therapy approach (Fig1). To assess uncertainty due to breath-hold variability, all plans were recalculated on additional DIBH CTs.

**Results:** Adaptive CPPT planning achieved mean D95% doses to the PTV of 97.8±1.3% over all patients and CTs, compared to 98.5±0.1% for IMRT. Medium and low OARs doses were substantially reduced by CPPT (Fig2) with lungs without GTV V20Gy being 17.9±0.2% vs. 28.3±0.1% and heart V30Gy 7.4±0.2% vs. 12.6±0.4% for adaptive CPPT and IMRT, respectively.

**Conclusions:** Adding a fixed horizontal proton beam line to a photon site could benefit treatment of NSCLC by reducing low and medium doses to OARs. Plan adaption may however be necessary to mitigate effects of anatomical changes on the proton component of treatments.

## O027 -

### Automated robust machine learning IMPT planning for oropharyngeal cancer

#### Ilse Van Bruggen ^1^

##### ^1^UMCG, Radiotherapy, Groningen, Netherlands

The aim of this study was to automatically generate Intensity Modulated Proton Therapy (IMPT) plans for oropharyngeal patients and to test if plan quality was at least equal compared to clinically and ‘manually' optimized robust IMPT plans. Machine Learning Optimization (MLO) planning involved training of a model using data of 73 oropharyngeal cancer patients (CT-scans, structures and dose distributions) to predict the dose distribution for new patients. A robust mimicking optimization algorithm using voxel-based mimicking and 21 perturbed scenarios was then used to generate a machine deliverable plan from the predicted dose distributions. Cross-validation was performed with 3x5 validation patients to tune prediction and mimicking settings. Plans were considered clinically acceptable when robust target coverage, conformity and normal tissue doses were within the following limits; clinical target volume D98 voxelwise minimum dose >94% (using multi-scenario dose evaluation strategy), conformity index decreased <10% and Normal Tissue Complication Probability (NTCP) (sum of grade-2 dysphagia and xerostomia) increased <2%, respectively. In 8/15 plans the MLO resulted in clinically acceptable plans. In these plans, the sum of NTCPs decreased on average with 0.37% (range: -2.3 - 1.8). The target conformity decreased more than 10% in 4/15 plans and the sum NTCP increased by more than 2% in 3/15 plans. MLO plans were generated including robustness evaluation in 67 +/- 8 minutes. Figure 1 and 2 represent average results. MLO with dose predictions and robust optimization automatically generated clinical acceptable robust IMPT plans for oropharyngeal cancer patients. Future work aims to increase the autoplanning success rate.

## O028 -

### Prostate cancer FLASH therapy treatments with electrons of high energy: a feasibility study

#### Gaia Franciosini^1,2^, Patrizia De Maria^2,3^, Micol De Simoni^1,2^, Marta Fischetti^2,4^, Michela Marafini^2,5^, Massimiliano Pacilio^6^, Damiana Rubeca^4^, Alessio Sarti^2,4^, Angelo Schiavi^2,4^, Marco Schwarz^7,8^, Vincenzo Tombolini^9^, Marco Toppi^4,10^, Giacomo Traini^2^, Antonio Trigilio^1,2^, Vincenzo Patera^2,4^

##### ^1^Sapienza University of Rome, Physics, Rome, Italy ^2^INFN Istituto Nazionale Fisica Nucleare, Sezione di Roma I, Rome, Italy ^3^Sapienza University of Rome, Post-graduate School in Medical Physics Department of Medico-Surgical Sciences and Biotechnologies, Rome, Italy ^4^Sapienza University of Rome, Scienze di Base e Applicate per l'ingegneria, Rome, Italy ^5^Museo Storico della Fisica e Centro Studi e Ricerche, “E. Fermi”, Rome, Italy ^6^Azienda Ospedaliera-Universitaria Policlinico Umberto I, Unità di Fisica Sanitaria, Rome, Italy ^7^Trento Institute for Fundamental Physics and Applications TIFPA, National Institute for Nuclear Physics- INFN, Povo, Italy ^8^Azienda Provinciale per i Servizi Sanitari APSS, Protontherapy, Trento, Italy ^9^Sapienza University of Rome, Scienze Radiologiche- Oncologiche e Anatomo Patologiche, Rome, Italy ^10^INFN Istituto Nazionale Fisica Nucleare, Sezione dei Laboratori di Frascati, Rome, Italy

Over the past decades, technological advances have improved the efficacy of radiation therapy (RT) cancer treatment. Nevertheless, the RT potential is still limited by normal tissue complications. In this context, FLASH radiotherapy represents a promising perspective, thanks to the achievable significant reduction of the healthy tissue damage. Several laboratories are performing first tests of FLASH irradiation. A possible limitation in implementing FLASH protons treatments is currently represented by their delivery performed, so far, using the pencil beam scanning approach. Several seconds are needed to deliver a treatment reducing the dose-rate possibly below the limit from which the FLASH effect appears. For what concern photon beams instead, the implementation is currently limited by technical aspects related to the delivery of FLASH intensities. In this contribution an approach to cure deep-seated tumors exploiting the FLASH effect, using Very High Energy Electron (VHEE) beams with energies above 50 MeV will be shown together with the potential in reshaping the landscape of prostate treatments. An example of VHEE treatment optimised combining an accurate Monte Carlo simulation with a simple modelling of the FLASH effect will be compared with conventional RT and proton therapy. The results demonstrate that FLASH therapy with VHEE beams of 70-130 MeV could represent a valid alternative to standard RT allowing a better sparing of the healthy tissues surrounding the tumour, in the framework of an affordable technological development. The impact on prostate cancer will be discussed also in view of the results obtained when comparing with proton treatments.

## O029 -

### The first investigation of the feasibility to use bladder-preserving proton SBRT for muscle-invasive bladder cancer patients

#### Peilin Liu^1^, Xianshu Gao^1^, Shangbin Qin^1^, Zishen Wang^2^, Xiaomei Li^1^, Siwei Liu^1^, Chenghao Jia^1^, Xuanfeng Ding^3^

##### ^1^Peking University First Hospital, Radiation Oncology, Beijing, China ^2^Yizhou Proton Therapy Center, Radiation oncology, Zhuozhou, China ^3^Beaumont Health System- Rose Cancer Center, Radiation Oncology, Beaumont Health System- Rose Cancer Center, USA

**Purpose:** To quantitatively investigate inter-fraction bladder size and shape variations and assess plan dosimetric robustness and feasibility for the use of proton SBRT for muscle-invasive bladder cancer patients.

**Methods:** Nine muscle-invasive bladder cancer patients who were treated in our institution with VMAT SBRT were retrospectively selected in this study. Lipiodol was used to label the target (GTV) location. Pre-treatment' bladder volume was controlled by intravesical instillation (200-500cc). CBCT was used to evaluate the bladders' center mass, bladder wall, volume changes and proton dose reconstruction. GTV displacement was assessed based on the lipiodol location. Two field IMPT plan was created with a prescription of 18Gy in 3 fx.

**Results:** In comparison with initial planning CT, the inter-fraction mean bladders' center mass' shifts in the left-right (LR),superior-inferior(SI), and anterior-posterior (AP) direction was (0±1.05)mm, (-0.01±2.12) mm, (-0.11±3.53) mm during the treatment course. The mean displacement of the lipiodol (GTV) in the L-R, S-I, A-P direction was (-0.24±1.91)mm,(-0.64±1.81)mm,(-0.23±1.50)mm. The average bladder volume's change was 7.46±8.02 %. The mean Dice similarity of bladder's coefficient was 0.91. We found that the superior bladder wall has the most uncertainties, which shifted about 7.9 ±7.1mm on average. Overall, the V95 of GTV coverage based on CBCT was 96.74±1.59% where GTV degraded to 91.88% for the GTV located on the superior portion of the bladder.

**Conclusion:** Bladder geometry changes and deformation can be effectively mitigated using the intravesical instillation approach for bladder-preserving proton SBRT with target not located on the bladder's superior portion.

## O030 -

### Variable relative biological effectiveness (RBE) in proton therapy of gliomas

#### Jan Eulitz^1^, Felix Raschke^1^, Annekatrin Seidlitz^2^, Christian Hahn^2^, Felicia Permatasari^1^, Benjamin Lutz^3^, Erik Schulz^1^, Caroline Karpowitz^2^, Arne Grey^4^, Kay Engellandt^4^, Steffen Löck^2^, EstherGC Troost^2^, Krause Mechthild^2^, Armin Lühr^5^

##### ^1^Helmholtz-Zentrum Dresden - Rossendorf, Institute of Radiooncology – OncoRay, Dresden, Germany ^2^Faculty of Medicine and University Hospital Carl Gustav Carus- Technische Universität Dresden- Dresden- Germany, Department of Radiotherapy and Radiation Oncology, Dresden, Germany ^3^Helmholtz-Zentrum Dresden - Rossendorf, Institute of Radiation Physics, Dresden, Germany ^4^Faculty of Medicine and University Hospital Carl Gustav Carus- Technische Universität Dresden- Dresden- Germany, Department of Diagnostic and Interventional Neuroradiology, Dresden, Germany ^5^TU Dortmund University, Department of Medical Physics and Radiotherapy- Faculty of Physics, Dortmund, Germany

Currently, there is an intense debate on the need to consider variable clinical relative biological effectiveness (RBE) in proton therapy. Here, the variability of the clinical RBE was studied for late radiation-induced brain injuries (RIBI) observed after proton therapy in glioma patients. In total, 42 patients out of a consecutive WHO grade 2-3 glioma patient cohort that received (adjuvant) proton radio(chemo)therapy between 2014 and 2017 were eligible for analysis. RIBI lesions (symptomatic or clinically silent) were diagnosed and delineated on T1-weighted magnetic resonance imaging with contrast agent scans obtained in the first two years of follow-up. Correlation of RIBI location and occurrence with simulated dose (D), linear energy transfer (LET) and variable RBE dose parameters were tested in voxel- and in patient-wise logistic regression analyses, including anatomical and clinical parameters. Model performance was estimated through cross-validated area-under-the-curve (AUC) values. In 23 patients, 69 RIBI lesions were diagnosed. RIBI location and occurrence were significantly correlated with D×LET and variable RBE dose in voxel- and patient-wise regression analysis with cross-validated AUC values of 0.90 (95% confidence interval: 0.90-0.90) and 0.83 (0.60-1.00), respectively, when incorporating the periventricular region and tumor histology in the analysis (figure 1). Models without considering RBE variability revealed reduced AUC values of 0.88 (0.88-0.88) and 0.78 (0.51-1.00). Models with variable RBE performed substantially better in predicting the occurrence and location of RIBI when compared to constant RBE models. These results provide clinical evidence for a variable proton RBE and suggest its consideration in proton treatment planning of brain tumors.

## O031 -

### Physical characterization of 3He ion beams for radiotherapy and comparison with 4He

#### Felix Horst^1^, Dieter Schardt^1^, Hiroshi Iwase^2^, Christoph Schuy^1^, Marco Durante^1,3^, Uli Weber^1^

##### ^1^GSI Helmholtzzentrum für Schwerionenforschung, Biophysics, Darmstadt, Germany ^2^KEK, Radiation Science, Tsukuba, Japan ^3^Technical University Darmstadt, Institut für Festkörperphysik, Darmstadt, Germany

There is increasing interest in using helium ions for radiotherapy, complementary to protons and carbon ions. A large number of patients were treated with ^4^He ions in the US heavy ion therapy project in Berkeley and novel ^4^He ion treatment programs are under preparation, for instance in Germany and Japan. ^3^He ions have been proposed as an alternative to ^4^He ions because the acceleration of ^3^He is technically less difficult than ^4^He. In particular, beam contaminations have been pointed out as a potential safety issue for ^4^He ion beams. This motivated a series of experiments with ^3^He ion beams at GSI, Darmstadt. Measured ^3^He Bragg curves and fragmentation data in water are presented in this work. The physical characteristics of ^3^He ion beams are compared to those of ^4^He, for which a large set of data became available in recent years from the preparation work at the Heidelberg Ion Beam Therapy Center. The experimental comparison of ^3^He and ^4^He is supported by Monte Carlo simulations using the FLUKA code. The dose distributions (spread out Bragg peaks, lateral profiles) that can be achieved with ^3^He ions are found to be competitive to ^4^He dose distributions. The peak-to-entrance ratio is found to be slightly better for ^3^He ions. The effect of beam contaminations on ^4^He depth dose distribution is also addressed. It is concluded that ^3^He ions can be a viable alternative to ^4^He, especially for future compact therapy accelerator designs and upgrades of existing ion therapy facilities.

## O032 -

### Increased efficiency with PBS proton therapy delivery for moving targets

#### Oxana Actis^1^, David Meer^1^, Alexandre Mayor^1^, Urs Rechsteiner^1^, Jörg Rottmann^1^, Damien Charles Weber^1,2^

##### ^1^Paul Scherrer Institute, Center for Proton Therapy, Villigen, Switzerland ^2^University Hospital of Zürich, Radiation Oncology, Zürich, Switzerland

The treatment of mobile tumors with protons has become more prevalent in the last decade. To achieve the same treatment effectiveness as for static tumors however, standard beam delivery is typically combined with motion mitigation techniques. Those are not limited but include re-scanning or gating, both of which prolong treatment time significantly. In this work we present the novel approach for increasing the efficiency of re-scanning and show the results from the first year of operation using this strategy. At our institute mobile tumors are treated combining PBS with gating and volumetric re-scanning (VR) where the whole tumor volume is scanned multiple times. Initial implementation of VR used only decreasing beam energies, creating a substantial dead time due to the need to ramp the beam line before each re-scan. Therefore, in 2019, we commissioned an energy meandering strategy that allows us to avoid beam line ramping in-between energy series whilst maintaining beam delivery quality. After one year of operation with this mode, we have analyzed the individual beam delivery time for each patient and have compared it to simulations of the timing behavior assuming unidirectional energy application. 12 out of 83 analyzed patients were treated employing re-scanning/gating. Depending on complexity, plan delivery times were reduced from 25% to 65%, with a median time gain of 47% for all 4D treatments. In addition, we observed that together with high efficiency increase for re-scanning, other modalities like patching and treatments with preabsorber also benefit from our new method.

## O033 -

### Relationship between beam current and scan speed for total irradiation time

#### Nagaaki Kamiguchi^1^, Nobuaki Takahashi^1^, Kenzo Sasai^2^, Takuya Miyashita^2^, Yukio Mikami^1^

##### ^1^Sumitomo Heavy Industries- Ltd., Technology Research Center, Yokosuka, Japan ^2^Sumitomo Heavy Industries- Ltd., Industrial Equipment Division, Tokyo, Japan

The breath holding method is one of the approaches for a moving target with respiration in the radiation treatment field. On the other hand, proton therapy systems comprising cyclotrons can potentially deliver rather high beam current (few 10 nA). High beam current can make dose rate higher and it makes short time dose delivery into targets possible within single breath holding. Sumitomo Heavy Industries has employed the Line scanning method as PBS method, which scans the beam continuously with scan speed modulated as intensity modulation method. The beam current is determined from the minimum weight of irradiation unit in each single layer and the minimum weight is given maximum scan speed. The beam currents may get higher if the maximum scanning speed becomes higher. In addition, the optimization for each weight affect total irradiation time. If minimum weight is restricted to higher value, the beam current may increase but this must take into account DVH and dose distribution. In this presentation, the effect of the shortening irradiation time with these variables was evaluated for each clinical mock case. Fast layer switching which had been developed takes at most 0.3 s to change the beam energy. By combining FLS and fast scanning speed, it was made clear that 2s irradiation is possible, which requires 100 mm/ms scanning speed as the maximum. Since the tolerance of breath holding time is known as 6-7s, this system can deliver a less stressful treatment for patients.

## O034 -

### First test of FLASH with continuous line scanning

#### Nagaaki Kamiguchi^1^, Takuya Miyashita^2^, Kenzo Sasai^2^, Masanori Tachibana^2^, Yukio Mikami^1^

##### ^1^Sumitomo Heavy Industries- Ltd., Technology Research Center, Yokosuka, Japan ^2^Sumitomo Heavy Industries- Ltd., Industrial Equipment Divisinon, Tokyo, Japan

In current situation of radiation therapy, the FLASH effect is attracting attention. Originally this effect was discovered at the conventional linacs but also is expected at the proton therapy because of the feature of the proton accelerator which can output higher dose rate among the commercial any accelerators. Sumitomo Heavy Industries, Ltd. (SHI) has been developing the proton therapy system using cyclotrons. Besides, the ripple filters have been developed to form SOBP as the approach of fast irradiation. The capability of application of this technology has been considered for the FLASH. To obtain the FLASH effect, over 40 Gy/s dose rate and over 8 Gy dose are required within single irradiation. OARs and skin require these dose amount and dose rate as the FLASH effect spears the normal tissue. In this study, SHI defined the dose-average dose rate was used as the dose rate. This means the irradiation must complete in 0.2 s when 8 Gy is delivered with 40 Gy/s. The fastest way is to select 230 MeV mono-layer which can be available highest beam current and to employ the ripple filters to make 2 cm width SOBP. SHI's system can deliver over 100 nA beam for 230 MeV with non-clinical mode and this current can deliver uniformly 8 Gy dose to 2 × 2 × 2 cm^3 volume within 0.1 s. The results of this measurement will be presented.

## O035 -

### Measuring beam intensity in FLASH experiments with a non-intercepting and non-saturating monitor

#### Sudharsan Srinivasan^1^, Jacobus Maarten Schippers^1^, Pierre-André Duperrex^1^, Serena Psoroulas^2^, Vivek Jaysukhlal Maradia^2^

##### ^1^Paul Scherrer Institute, GFA, Villigen, Switzerland ^2^Paul Scherrer Institute, Cpt, Villigen, Switzerland

In experiments at PSI's proton therapy facility PROSCAN, the FLASH effect is being investigated with the 250 MeV proton beam from the superconducting cyclotron. For these experiments, it will deliver high beam currents during pulses of 1-1000 milliseconds. A major challenge in these FLASH-beam conditions is the use of accurate dosimetry devices and the characterization of suitable detectors. Until now, invasive monitors such as ionization chambers measure the high beam currents used in FLASH research. However; these monitors are prone to significant recombination effects with increased dose-rates, which are dependent on the beam shape. For FLASH, these monitors could limit the absolute dose accuracy since they need complicated and extensive calibration procedures. A novel type of cavity resonator for non-interceptive beam current measurements has been developed at PSI. This new monitor, with our existing signal-processing unit (signal integration of 1 second), has demonstrated accurate measurements and a beam shape independent linear response for beam currents in the normally used range of 0.1-10 nA. With the same signal-processing unit, we have measured beam currents up to 100 nA accurately with no saturation, which indicates their advantage in FLASH experiments. To comply with the short FLASH pulses, we are adapting the readout electronics to take advantage of the fast response (signal integration of few microseconds) the cavity monitor offers for FLASH intensities. In this paper, we report on the new monitor's observed response for beam currents corresponding to FLASH dose-rates and the advantages it presents compared to the ionization chambers.

## O036 -

### Feasibility of 3D printed ridge filter to enable SBRT FLASH therapy using scanning proton beam

#### Ruirui Liu^1^, Serdar Charyyev^1^, Jun Zhou^1^, Xiaofeng Yang^1^, Tian Liu^1^, Mark McDonald^1^, Kristin Higgins^1^, Jeffrey Bradley^1^, Liyong Lin^1^

##### ^1^Emory University, Radiation Oncology, Atlanta, USA

**Purpose:** Due to long proton energy switching time, most FLASH therapy is limited to single energy and to spare normal tissue without conformity to treatment target along the depth direction. We investigate if 3D ridge filter can enable dose conformity to stereotactic radiation therapy (SBRT) target in FLASH treatment using scanning proton beams.

**Methods:** Varian ProBEAM's FLASH energy of 250 MeV was degraded to 120 MeV to treat simulated SBRT spherical targets of 30mm and 50mm diameters at depths of ∼80mm of a water phantom. We developed a method to optimize the height and location of PMMA pins of ridge filter so that spread-out Bragg peaks width at each pin location is conformal to the extension of treatment target. The ridge filter pins must meet the 0.1-mm lateral and height resolutions of 3D printer. Geant4 (Fig. 1) was used to simulate the radiation transport. The spot weights were also optimized to achieve a lateral target conformity.

**Results:** The simulation results (Fig. 2) showed that it is feasible to use 3D printed ridge filters to conform location dependent spread-out Bragg peaks to SBRT spherical targets for one fixed energy. Delivering 10Gy dose in such spot maps is estimated to be < 250ms, achieving Flash dose rate of 40Gy/s. The validation of timing and simulated dosimetric distributions will be performed since FLASH mode has been recently delivered at Emory.

**Conclusion:** According to Geant4 simulation, it is feasible to use 3D ridge filter to enable proton SBRT FLASH therapy up to 50-mm diameter sphere target.

## O037 -

### Computational simulation modelling to guide precision medicine in paediatric cranial proton and photon radiotherapy: towards a patient specific approach

#### Mikaela Dell'oro^1,2^, Puthenparampil Wilson^2,3^, Michala Short^1^, Chia-Ho Hua^4^, Thomas E. Merchant^4^, Eva Bezak^1,5^

##### ^1^University of South Australia, Cancer Research Institute, Adelaide, Australia ^2^Royal Adelaide Hospital- SA Health, Radiation Oncology, Adelaide, Australia ^3^University of South Australia, UniSA STEM, Adelaide, Australia ^4^St. Jude Children's Research Hospital, Radiation Oncology, Memphis, USA ^5^University of Adelaide, Department of Physics, Adelaide, Australia

**Objectives**: As normal tissue complication risk (NTCP) for paediatric organs are not well understood, we base radiation response on adult data. This study adjusted NTCP models to estimate sex-specific radiosensitivity and predict cranial side-effects following intensity-modulated proton therapy (IMPT) and intensity-modulated radiotherapy (IMRT).

**Methods**: A total of 216 IMRT and IMPT plans were generated in Eclipse (Varian, version 13.7) for different medulloblastoma volumes and locations using age-specific paediatric CT scans (5, 9 and 13 years-of-age). Dose-volume histograms were extracted for a range of simulated tumour volumes and plans. Lyman-Kutcher-Burman (LKB) radiobiological model was used to calculate NTCP values for the endpoints, where TD50, as in adult literature, was adjusted to simulate represent sex dependence (assumption being females are ∼ 20% more radiosensitive). Sensitivity analyses of sex-specific radiosensitivity were performed to gauge the influence of factors on radiation response.

**Results**: IMPT plans demonstrated lower NTCP compared to IMRT across all models (*p* < 0.0001). Figure 1 illustrates the females (dashed line) could be up to 20% more radiosensitive compared to males (dotted line). The risk of brainstem necrosis (> 10%) and cochlea tinnitus (> 20%) for females could potentially be underestimated if sex-specific TD50 was not considered (Table 1).

**Conclusion**: Based on these assumptions, the difference in NTCP between sexes was significant (*p* < 0.0001), indicating female cranial paediatric patients could be twice as likely to experience side-effects of brainstem necrosis and cochlea tinnitus at lower doses compared to males, highlighting the need for considering the gender effect in NTCP models.

## O038 -

### Setup uncertainty of pediatric proton therapy patients prospectively evaluated with daily robotic cone-beam CT

#### Chiaho Hua^1^, Andrew Saunders^1^, Matthew Marker^1^, Yimei Li^2^, Yuanyuan Han^2^, Matthew Krasin^1^, Thomas Merchant^1^

##### ^1^St Jude Children's Research Hospital, Radiation Onclogy, Memphis, USA ^2^St Jude Children's Research Hospital, Biostatistics, Memphis, USA

**Objective**: To report findings from a prospective trial quantifying setup uncertainty with and without volumetric image guidance, intra-fractional patient movement, and differences between planar and volumetric image guidance in children receiving proton therapy.

**Methods**: Protocol-specified imaging included daily pre-treatment cone-beam CT (CBCT), weekly post-treatment CBCT, verification CBCT after applying setup corrections with a 6-degree-of-freedom robotic couch, and planar radiographs. Data of 47 patients (aged 10 months-25 years, 17 anesthetized) with tumors above the neck were analyzed. Patients were immobilized with a full head thermoplastic mask. Therapists positioned patients based on the 3-point marking on the mask and a sagittal alignment mark on the chin.

**Results**: Setup uncertainty (2-sigma) from manual positioning was 3.5mm, 5.5mm, 5.1mm, 2.5°, 2.2°, and 2.3° in lateral, longitudinal, vertical, pitch, roll, and yaw directions, respectively. After applying CBCT-derived corrections via automatic robotic couch movements, residual setup uncertainty reduced to 0.7mm, 1mm, 0.6mm, 0.6°, 0.5°, and 0.7°. The most frequent (26%) >1mm/1° discrepancy between planar and volumetric image guidance occurred in the axial head rotation. Intra-fractional movements were largely attributed to poor fitting face masks from weight and hair loss. Positional uncertainty measured at the end of fractional treatments increased to 1.4mm, 1.9mm, 2.1mm, 1°, 1.1°, and 1.2° for awake patients but stayed within 1mm/1° for those treated under general anesthesia.

**Conclusions**: Daily CBCT image guidance reduced setup uncertainty to ≤1mm/1° in each direction. For anesthetized children, uncertainty remained small throughout treatment. The study findings guide non-uniform planning margin design, robust optimization settings, and immobilization improvement.

## O039 -

### Auto-contouring of organs-at-risk using artificial intelligence deep learning in pediatric brain cancer at the Pediatric Proton/Photon Consortium Registry (PPCR)

#### Dora Correia^1^, Hanlin Wan^2^, Sara Gallotto^1^, Benjamin Bajaj^1^, Ethan Platt^2^, Miranda P. Lawell^1^, Nicholas DeNunzio^3^, Paul Jacobs^2^, Torunn I. Yock^1^

##### ^1^Massachusetts General Hospital- Harvard Medical School, Radiation Oncology, Boston- MA, USA ^2^MIM Software Inc., Research, Cleveland- OH, USA ^3^John Theurer Cancer Center- Hackensack Meridian Health Network, Radiation Oncology, Hackensack- NJ, USA

**Background**: Artificial intelligence (AI) may improve normal tissue delineation by increasing efficiency and, potentially, quality. We explore the feasibility of the MIM® AI deep-learning algorithm to automate pediatric brain organs-at-risk (OAR) segmentation.

**Methods**: AI was trained using expert delineation of 17 pediatric intracranial OAR on 50 PPCR treatment plans (CT/MRI; median age 8 years, range: 1-21). After fivefold cross-validation, we applied it in a PPCR pediatric brain tumor set (n=20), evaluated contouring time, concordance (manual, AI, and AI+manual), and compared dosimetry. Dice similarity coefficient (DSC) was used to assess the segmentation performance against the manual contours.

**Results**: Higher concordance (DSC>0.85) was found in 35.3% of structures, in all larger volume OARs (>22.9 cc's, median DSC=0.89) such as brain, infra-/supratentorial brain, brainstem, and temporal lobes (Table 1), despite 10% (and 16% of the training model) presenting major anatomical changes from surgery/tumor. As expected, smaller volume OARs presented an intermediate median DSC=0.59. No correlation between age and concordance was found. On average, manual, AI, and AI+manual contouring for 17 structures took, respectively, 144, 21, and 82 minutes. The mean dose (Dmean) of 88.2% of structures contoured by AI was within 2GyRBE of the manual contour dose. All AI+manual contours were within 1GyRBE Dmean of the manually contoured structures.

**Conclusions:** The MIM AI deep-learning algorithm proved both feasible to auto-contour most brain OARs and efficient, and accuracy was not affected by patient age. While manual corrections of AI structures improved DSCs and contour appearance, it only marginally improved mean dosimetry to OARs.

## O040 -

### Proton therapy in the management of soft tissue sarcoma: multi-institutional prospective results from the Proton Collaborative Group

#### Arpit Chhabra^1^, Stephanie Rice^2^, J. Isabelle Choi^1^, Robert Press^1^, Shaakir Hasan^1^, John Chang^3^, Henry Tsai^4^, Michael Durci^5^, Craig Stevens^6^, William Hartsell^7^, Dan Kunaprayoon^8^, George Laramore^9^, Carlos Vargas^10^, Charles B. Simone 2nd^1^

##### ^1^New York Proton Center, Department of Radiation Oncology, New York, USA ^2^SUNY Upstate Medical University, Department of Radiation Oncology, Syracuse, USA ^3^Oklahoma Proton Center, Department of Radiation Oncology, Oklahoma City, USA ^4^Procure Proton Therapy Center, Department of Radiation Oncology, Somerset, USA ^5^Willis-Knighton Cancer Center, Department of Radiation Oncology, Shreveport, USA ^6^Beaumont Health, Department of Radiation Oncology, Royal Oak, USA ^7^Northwestern Medicine Proton Center, Department of Radiation Oncology, Warrenville, USA ^8^Maryland Proton Treatment Center, Department of Radiation Oncology, Baltimore, USA ^9^University of Washington, Department of Radiation Oncology, Seattle, USA ^10^Mayo Clinic - Arizona, Department of Radiation Oncology, Phoenix, USA

**Purpose:** Radiation therapy is an important modality to achieve local control in soft tissue sarcomas (STS). Data on proton beam therapy (PBT) in the management of STS remain limited. We hypothesize that PBT can achieve durable local control with a low side effect risk profile.

**Methods:** We analyzed the multicenter Proton Collaborative Group (PCG) registry for patients with benign and malignant STS that received preoperative, definitive, or postoperative PBT. Overall survival (OS), freedom from local-regional recurrence (FFLR), and freedom from distant metastases (FFDM) were calculated for the entire sample and for associated subgroups (preoperative, definitive, postoperative). Toxicity was calculated as per CTCAE-v.4.0.

**Results:** Thirty-six consecutive patients with STS underwent preoperative (11.1%), definitive (n=3, 8.3%), or postoperative (80.6%) PBT between 2009 and 2017. Predominant histologies included malignant peripheral nerve sheath (13.9%), leiomyosarcoma (13.9%), spindle cell (11.1%). Median preoperative, definitive, and postoperative PBT doses were 50.0Gy, 53.7Gy, and 61.7Gy, respectively. Median follow-up time was 11.6 months. Median, 1-year, and 2-year OS rates were 27.5 months, 77%, and 67%, respectively. The 1-year and 2-year rates of FFLR were 93% and 86%, respectively. The 1-year and 2-year rates of FFDM were both 89%. No statistical difference in outcomes was observed between the subgroups. Toxicities were mild, with a Grade 2 acute toxicity rate of 47.1%, and Grade >3 acute and late toxicity rates of 2.9%, and 2.9%, respectively.

**Conclusions:** This study shows excellent local control following PBT in the management of STS, with a lower high-grade side effect profile as compared to previous photon therapy reports.

## O041 -

### Carbon ion radiotherapy for retroperitoneal sarcoma

#### Reiko Imai^1^, Kazutoshi Murata^1^, Hirotoshi Takiyama^1^, Masaru Wakatsuki^1^, Shigeru Yamada^1^, Hiroshi Tsuji^1^

##### ^1^National Institutes for Quantum and Radiological Science and Technology, QST Hospital, Chiba, Japan

Between 2000 and 2014, 50 patients with retroperitoneal sarcomas treated with carbon ion radiotherapy (CIRT) at a single institute were analyzed. Tumors originating from pre- and intra-sacral space were excluded. Median age was 57 years, and the patients who did not have surgery prior to CIRT were 32 cases. The 32 cases were determined to be medically inoperable. The most common histology was undifferentiated pleomorphic sarcoma. The median total applied dose was 70.4 Gy (RBE) in 16 fractions over 4 weeks. The 3-year and 5-year overall survival rates were 60% and 55%, respectively. The 5-year local control rate was 65%. For adverse events, vertebral fracture causing walking impairment was observed in 1 patient and 3 patients experienced G3 intestinal adverse events. CIRT was useful for retroperitoneal sarcomas that are not candidate for resection, however the oncologic outcome of CIRT needs further improvement. Recently absorbable spacer sheets were developed and approved by the Japanese public insurance system, which can make a distance between tumors and intestines. It will be of great assistance for CIRT for retroperitoneal sarcoma.

## O042 -

### Proton therapy for bone and soft tissue sarcomas: initial clinical outcomes from the UK Proton Overseas Programme

#### Simona Gaito^1^, Neil Burnet^2^, Philip Foden^3^, Cy Howells^3^, Shermaine Pan^1^, Dan Saunders^1^, Adrian Crellin^4^, Ed Smith^1^

##### ^1^The Christie NHS FT, Clinical Oncology, Manchester, United Kingdom ^2^University of Manchester, Division of Cancer Sciences, Manchester, United Kingdom ^3^The Christie NHS FT, Proton Clinical Outcomes Unit PCOU, Manchester, United Kingdom ^4^NHS England National Clinical Lead Proton Beam Therapy, Clinical Oncology, Leeds, United Kingdom

**Purpose**: We report the long term outcomes on UK patients treated for bone and soft tissue sarcomas within the Proton Overseas Programme (POP). This serves as an important comparator for the newly established UK Proton Beam Therapy (PBT) service which started in December 2018.

**Material and methods**: This is a retrospective series of 405 patients with sarcomas, referred from 46 UK centres for consideration of PBT between 2008 and 2018. All cases were of performance status 0-1,appropriately staged and received treatment in five experienced PBT centres. Systematic follow-up data was available for 327 patients.Local control (LC) and 5-years survival rates were analysed.

**Results**: Patients and treatment related characteristics are listed in Table 1. Of note, PBT was delivered with passive scatter technique. Systemic treatment was given as per current disease-specific European protocols. After a median follow-up of 26 months (range 0-111), the Local Control (LC) rate for the whole cohort is 84.7%; LC rates are 85.6 % for ≤25y vs 66.7 % for >25y. LC rates by histological subgroups and tumour sites are shown in Table 1. Figure 1 illustrates the Kaplan-Meier survival estimates for histology. One year survival rate for rhabdomyosarcoma, Ewing's sarcoma and other sarcoma subgroups are 92.9% (95% CI: 89.1-96.9%), 92.5% (88.0-97.4%) and 94.3% (86.9-100%),respectively.

**Conclusions**: In this cohort, PBT was an effective modality for LC. Preliminary survival results are encouraging, but longer follow up is needed. Analyses of toxicities are ongoing. The POP has facilitated equitable access to PBT abroad for patients with complex needs from across the UK without disadvantaging patient outcome.

## O043 -

### Degradation of particle depth dose in lung tissue: An efficient and consistent model for Monte Carlo and analytical dose calculation

#### Noa Homolka^1,2,3^, Paul Anton Meder^1,3^, Lucas Burigo^1,3^, Hans-Peter Wieser^1,3,4^, Mark Bangert^1,3^, Oliver Jäkel^1,3,5^, Malte Ellerbrock^3,5^, Niklas Wahl^1,3^

##### ^1^German Cancer Research Center DKFZ, Medical Physics in Radiation Oncology, Heidelberg, Germany ^2^Ruprecht Karl University of Heidelberg, Medical Faculty, Heidelberg, Germany ^3^Heidelberg Institute for Radiation Oncology HIRO, x, Heidelberg, Germany ^4^Ludwig Maximilian University of Munich, Faculty for Physics, Munich, Germany ^5^Heidelberg Ion Therapy Center HIT, x, Heidelberg, Germany

Microscopic lung tissue structures can cause a degradation of the Bragg peaks in particle treatment plans, potentially causing under-dosage of the target and over-dosage distal to the target. We present a consistent and efficient method for degradation modeling in deterministic and Monte Carlo dose calculation based on binomial voxel-sampling. The model relies on averaging a series of simulations with lung voxel densities sampled from a binomial distribution, whose parameters depend on the assumed modulation power representing microscopic substructures and the voxel density estimated from the planning CT. Compared to previous approaches, the method only yields physically reasonable voxel densities, allows free choice of parameters and requires no substantial precomputations. Pencil-beams in a water phantom with a lung slab were calculated for protons, helium and carbon ions. Analytical dose calculations and heterogeneity corrections with a worst-case modulation power (800μm) were computed with the open-source treatment planning toolkit matRad according to Winter et al. (2020). Monte Carlo doses were calculated with TOPAS 3.5 using 1e6 histories across 10 lung samples. The calculations for carbon ions include RBE using tabulated LEM I data. Figure 1 shows integrated depth doses for protons, helium and carbon ions. The results demonstrate agreement between the sampling model and the analytical convolution correction. For carbon ions, we could also demonstrate transferability to RBE-weighted dose. In conclusion, our lung degradation simplifies previous approaches and generalizes to Monte Carlo and other dose calculation methods. We are currently validating the model for patient treatment plans.

## O044 -

### Efficient uncertainty estimates in Monte Carlo dose calculation using importance re-weighting

#### Pia Stammer^1,2,3^, Lucas Burigo^2^, Oliver Jäkel^2^, Martin Frank^1^, Niklas Wahl^2^

##### ^1^Karlsruhe Institute of Technology, Steinbuch Centre for Computing, Karlsruhe, Germany ^2^German Cancer Research Center - DKFZ, Department of Medical Physics in Radiation Oncology, Heidelberg, Germany ^3^HIDSS4Health, Helmholtz Information and Data Science School for Health, Karlsruhe/Heidelberg, Germany

Range and set-up uncertainties can cause significant discrepancies between planned and delivered dose. Comprehensive uncertainty analyses, which are usually based on scenario samples, can however quickly become infeasible when using Monte Carlo (MC) dose calculation due to its computational cost. We propose a technique to mitigate this computational challenge by inferring dose uncertainties from one MC dose calculation. We exploit the approximately Gaussian shape of individual beamlets, using importance sampling in an event-by-event mode. Given the phase space of primary particles, the dose of the corresponding histories is re-weighted to reconstruct error scenarios and subsequent uncertainty estimates, making scenario computation a pure scoring problem. For Gaussian beamlets and uncertainties, the expected value can be directly sampled from their joint density and therefore only requires one additional calculation. Thus, depending on the quantities of interest, either (1) the nominal or (2) the expected distribution can be used as a basis for reconstruction. Importance weights are computed based on the underlying multivariate uncertainty model. Therefore, correlation models can be explicitly modeled, e.g. to reflect beam application or movement patterns. For set-up errors, the re-weighting approach could reproduce the expected dose and standard deviation with high accuracy using approach (1) (Figure 1.a). Estimates for range errors, which were approximated by re-weighting the energy distribution, yield less accurate results, which can be improved using approach (2) (Figure 1.b). Implemented in Matlab, run-times decrease by a factor of 5-10, showing that importance re-weighting can be an efficient tool for uncertainty estimates with MC simulations.

## O045 -

### Validation of the GPU-based AcurosPT

#### Chih-Wei Chang^1^, Katja Langen^1^, Liyong Lin^1^

##### ^1^Emory University, Radiation Oncology, Atlanta, USA

**Purpose:** GPU-based proton Monte Carlo dose calculation (MCDC) has shown the advantage in gaining computing efficiency without sacrificing its accuracy. It potentially can benefit dose-averaged linear energy transfer (LET_d_) prediction by implementing detailed physics models such as the transportation of δ-rays and propagation of secondary neutrons. The scope of this study is to evaluate the Varian newly developed GPU-based AcurosPT.

**Methods:** A standardized MCDC commissioning framework was applied to validate GPU-based AcurosPT v16.1 using non-water materials, a CIRS M701 anthropomorphic phantom, and IROC proton phantoms, and patient plans. TOPAS V3.1.3 was used to benchmark AcurosPT and quantify the uncertainty of proton characteristics in artificial materials such as polymethyl methacrylate (PMMA), polyethylene, and newly available titanium alloy (Ti6Al7Nb). Measurements were compared to AcurosPT using gamma criteria of 3%/3 mm and doses greater than 5% maximum dose.

**Results:** The benchmark results for discrepancies of 80% distal ranges (R80) are 0.4, 0.8, and 0.4 mm for PMMA, polyethylene, and Ti6Al7Nb. The dose calculation by AcurosPT shows good agreement to the measurement using the anthropomorphic phantom, and gamma passing rates are 99.4%, 96.5%, 95.2%, 93.3%, and 94.7% for the brain, HN, lung, abdomens, and pelvis. GPU-based AcurosPT generally can accelerate MCDC about ∼2.5 times faster than CPU-based AcurosPT.

**Conclusion:** The GPU-based AcurosPT v16.1 has been validated including proton ranges, proton scattering models, and time improvement over the CPU-based AcurosPT. The GPU-based AcurosPT shows significant improvements in computational economics without scarifying the accuracy of dose calculation.

## O046 -

### Comparison of Monte Carlo code choice for out-of-field dose calculation in pencil beam scanning proton therapy

#### Keith Griffin^1,2^, Yeon Soo Yeom^1^, Matthew Mille^1^, Choonik Lee^3^, Jae Won Jung^4^, Nolan Hertel^2^, Choonsik Lee^1^

##### ^1^National Cancer Institute, Radiation Epidemiology Branch- Division of Cancer Epidemiology and Genetics, Rockville, USA ^2^Georgia Institute of Technology, George W. Woodruff School of Mechanical Engineering, Atlanta, USA ^3^University of Michigan, Department of Radiation Oncology, Ann Arbor, USA ^4^East Carolina University, Department of Physics, Greenville, USA

Normal tissue dose estimates are an important input for epidemiological studies of late effects from proton therapy. Since most proton therapy treatment planning systems are designed to focus on in-field dose calculations, Monte Carlo (MC) methods are considered a gold-standard approach for out-of-field dose calculation from secondary particles. However, the physics modelling of secondary particle production from the beamline is uncertain, with various models used to reenact the nuclear breakup. In the present study, we compared three MC codes (MCNP6, PHITS, and TOPAS) with the developer-recommended physics settings to investigate the variation in normal tissue dose estimation for pencil beam scanning proton therapy patients. First, we compared total yield and double-differential production of two major secondary particles, neutrons and gammas, through proton pencil beam irradiation of a water phantom at four energies (80, 90, 100, and 110 MeV). This comparison showed a roughly 15% variation in neutron yield and a nearly two-fold variation in gamma yield per primary proton. Across the three codes, we then compared the out-of-field organ doses for an intracranial irradiation using whole-body computational human phantoms. The resulting out-of-field organ doses were comparable between MCNP and TOPAS. PHITS organ doses were lower than those from the other codes, by nearly a factor of two for organs far out-of-field. Investigation into these results found a stronger bias in forwardly-directed neutron production by PHITS. A figure of merit comparison showed, however, that PHITS performed the most efficient MC simulation – remarkably by two orders of magnitude.

## O047 -

### Robust alternating RBE- and LET-weighted beam orientation and fluence map optimization for intensity modulated proton therapy

#### Pavitra Ramesh^1^, Qihui Lyu^1^, Wenbo Gu^2^, Dan Ruan^1^, Ke Sheng^1^

##### ^1^University of California Los Angeles, Radiation Oncology, Los Angeles, USA ^2^University of Pennsylvania, Radiation Oncology, Philadelphia, USA

Empirical relative biological effectiveness (RBE) models and dose averaged LET are separately used to estimate biological dose in proton therapy. We incorporate McNamara RBE (RBE_M_) and LET in a single beam orientation (BOO) and fluence map optimization (FMO) framework with alternating objective functions. Based on robust sensitivity-regularized and heterogeneity weighted BOO (SHBOO), we formulated the optimization problem using two methods. The first optimized RBE_M_-weighted dose, updating RBE_M_ values with each round of optimization until they converged (RBEwFMO). The second alternated RBE_M_-weighted and LET-weighted objectives, until RBE_M_ convergence (RBELETwFMO). We compare their performance with an RBE_1.1_ physical dose optimizer (PHYSFMO). Three head and neck patients were planned with the techniques. Compared to PHYSFMO, CTV homogeneity and Dmax improved by an average of 0.02 and 1.4 GyRBE for RBELETwFMO, and 0.03 and 2.3 GyRBE for RBEwFMO. D98% improved by RBEwFMO by 0.6 GyRBE. Slight decrease in mean and maximum RBE dose was seen in larynx, esophagus, and constrictors with RBEwFMO and RBELETwFMO. Worst [Dmax, V95%, D95%, D98%] for CTVs improved by [4.2%, 31.9%, 5.5%, 4.7%] with RBEwFMO and [2.4%, 33.2%, 6.7%, 5.4%] with RBELETwFMO under range and setup uncertainties. For OARs, worst [Dmax, Dmean] improved by an average of [3.1, 0.8] GyRBE for RBEwFMO and [5.3, 2.0] GyRBE for RBELETwFMO under setup uncertainties. The three techniques deliver comparable physical doses for the H&N cases. Beside modest OAR sparing improvement, CTV coverage and robustness were substantially improved with RBEwFMO and RBELETwFMO, showing potential for directly incorporating McNamara RBE in proton treatment planning.

## O048 -

### A systematic review on the usage of averaged LET in radiation biology for particle therapy

#### Fredrik Kalholm^1,2^, Leszek Grzanka^3^, Erik Traneus^4^, Niels Bassler^1,5,6,7^

##### ^1^Stockholm University, Medical Radiation Physics, Stockholm, Sweden ^2^Karolinska Institutet, Department of Oncology and Pathology, Stockholm, Sweden ^3^Polish Academy of Sciences, Institute of Nuclear Physics, Cracow, Poland ^4^RaySearch Laboratories AB, Not applicable, Stockholm, Sweden ^5^Aarhus University Hospital, Danish Centre for Particle Therapy, Aarhus, Denmark ^6^Aarhus University Hospital, Department of Experimental Clinical Oncology-, Aarhus, Denmark ^7^Aarhus University, Department of Clinical Medicine, Aarhus, Denmark

Averaged Linear Energy Transfer (LET) is widely used to express the radiation quality of ion beams with respect to relative biological effect. However, this quantity may be defined in multiple ways, and the chosen definition may impact the resulting reported value. We systematically reviewed the definitions of averaged LET found in the literature, and quantified which impact these various definitions have for different reference cell survival setups, by Monte Carlo simulations. A Pubmed-search yielded 734 results to be manually investigated, from which 354 publications were finally included in the review. The averaged LET definitions quantifying the relative biological effect (RBE) of hadronic beams were recorded for each included publication. We find that the exact specification of averaged LET is, generally, poorly defined. Some differences in definitions of averaged LET may lead to differences in reported values up to an order of magnitude. For publications concerning protons, dose averaged LET was mostly applied when reporting RBE. 60 % of the publications did not specify which secondary particles were included, further contributing to an ill-defined averaged LET, corresponding up to a 25 % and 14 % difference in RBE in the entry region and in the middle of a spread out bragg peak for protons, respectively. The inconsistent usage of averaged LET definitions also contributes to uncertainty in LET-based RBE models. Moving forward, we recommend that averaged LET reporting should be standardized to avoid inconsistencies, and to minimize the error contributed from the physics side when reporting RBE.

## O049 -

### Towards ultrasound assisted in vivo proton range verification with injectable superheated nanodroplets: a feasibility study

#### Sophie Heymans^1^, Bram Carlier^2^, Yosra Toumia^3^, Sjoerd Nooijens^4^, Marcus Ingram^4^, Andrea Giammanco^5^, Gaio Paradossi^3^, Emiliano d'Agostino^5^, Wouter Crijns^2^, Alexander Bertrand^6^, Uwe Himmelreich^7^, Jan D'hooge^4^, Edmond Sterpin^2^, Koen Van Den Abeele^1^

##### ^1^KU Leuven, Department of Physics, Kortrijk, Belgium ^2^KU Leuven, Department of Oncology, Leuven, Belgium ^3^University of Rome Tor Vergata, Department of Chemical Sciences and Technology, Rome, Italy ^4^KU Leuven, Department of Cardiovascular Sciences, Leuven, Belgium ^5^DoseVue, Physics, Diepenbeek, Belgium ^6^KU Leuven, Department of Electrical Engineering, Leuven, Belgium ^7^KU Leuven, Department of Imaging and Pathology, Leuven, Belgium

Range uncertainties currently prevent proton therapy from reaching its full potential, and constrain clinicians to adopt large safety margins and conservative planning strategies. Therefore, *in vivo* proton range verification techniques are actively sought. Superheated liquids, once deprived from homogeneous nucleation sites, have been shown to vaporize when exposed to ionizing radiation, a concept exploited in Glaser's bubble chamber, as well as in superheated drop detectors. We proposed to downsize this approach to injectable, coated superheated nanodroplets used in contrast enhanced ultrasound imaging. In particular, we investigated whether nanodroplets could be vaporized by proton radiation, and whether ultrasound imaging of the generated microbubbles could provide information about the proton range. To that aim, we dispersed perfluorobutane (PFB) nanodroplets encapsulated by two different shells (PCDA and PVA) in aqueous phantoms and irradiated them with a monoenergetic proton beam (62 MeV, doses from 2 to 20 Gy) at increasing temperatures (25°C, 37°C, and 50°C). We observed an inverse relationship between the LET vaporization threshold of the nanodroplets and the degree of superheat, in agreement with the thermal spike theory. Therefore, droplet vaporization was only induced by secondary reaction products at 25°C and 37°C, while vaporization was also triggered by primary protons at the end of their range at 50°C, as shown by the ultrasound contrast peak. Moreover, ultrasound imaging enabled to retrieve the proton range with sub-millimeter accuracy. Importantly, the thermal stability and radiation response of the nanodroplets at 37°C are incentives to initiate *in vivo* studies.

## O050 -

### Wireless device for adaptive planning based upon thermoacoustic range estimates - design and benchtop validation

#### Sarah Patch^1^, Mahesh Narayanaswamy^2^

##### ^1^Acoustic Range Estimates- UW-Milwaukee, all- Physics, Milwaukee, USA ^2^Swamy Enterprises, Design, Milwaukee, USA

**Purpose:** Transitioning thermoacoustic range verification from benchtop experiments with cumbersome research equipment to clinical practice is the purpose of our work (Fig. 1). A wireless device for thermoacoustic range verification with correlation to online ultrasound images has been developed for initial application to adaptive planning.

**Materials and Methods:** A 7 × 5.5 × 3.5 cm detector (NucSafe) requiring 5V power supply replaces the 0.5 m long prompt gamma detector that required -2 kV (Fig. 2a). Oscilloscopes are replaced by a wireless data acquisition board (DAQ) with vertical resolution of 38 uV (Swamy Enterprises). 4-6 acoustic channels are amplified; compact gamma detector signal is not amplified. Data is acquired continuously and stored on a secure data card for retrieval via serial port after treatment. Two different types of transducers provide broadband response in a compact form factor.

**Results:** A custom receive chain provides improved 10-18 dB greater sensitivity over the relevant frequency band (10-100 kHz, Fig 2b). Transducers positioned to either side of the ultrasound imaging array are up to 8 dB more sensitive to low frequencies (Fig 2c). The new DAQ provides 10 dB greater amplification. A wireless ultrasound imaging array (Clarius P4-1) provides greater imaging depth (30 cm vs 7 cm) and smaller footprint (Fig. 2d).

**Conclusions:** This improved device provides greater ease of use, positioning flexibility and receive sensitivity which could enable range verification during pulsed delivery of a 2 Gy fraction.

## O051 -

### The value of measurement-based proton peer review for clinical trials

#### Paige Taylor^1^, Jessica Lowenstein^1^, David Followill^1^, Stephen Kry^1^

##### ^1^MD Anderson Cancer Center, Radiation Physics, Houston, USA

IROC performed 38 on-site measurement-based peer reviews of proton centers participating in NCI-funded clinical trials. The review covered beam calibration, lateral and depth measurements, mechanicals, treatment planning, and quality assurance (QA) practices. Institutions received an average of 3 [1, 8] recommendations for practice improvements. The most common deficiencies were QA (97% of centers), CT Number-Relative Linear Stopping Power (RLSP) conversion (50%), and treatment planning (45%). 32% of institutions failed at least one lateral profile measurement (<90% of pixels passing 3%/3mm, 10% threshold), despite passing internal QA measurements. Table 1 shows that these failures occurred for several different plan configurations (large, small, shallow, and deep targets), and at different depths in the beam path (proximal to target, central, and distal), and all had passed institutional plan QA. Figure 1 shows the mean CT Number-RLSP conversion curve and ±2 sigma based on all collected data. This test highlights areas of inconsistency between proton centers, with many centers falling outside 2 sigma at some point along the curve. All deficiencies from the peer review were discussed with the institutions, and many implemented dosimetric, treatment planning, and practice changes to improve the accuracy of their system and consistency with other institutions. This peer review program has been integral in confirming comparability and consistency across proton centers for clinical trials, minimizing deviations for outcomes data.

## O052 -

### Deep-learning and CBCT-based synthetic CT for online dose verification of the proton treatment of sinonasal cancer patient

#### An Qin^1^, Lewei Zhao^1^, Shupeng Chen^1^, Xiaoqiang Li^1^, weili zheng^1^, Di Yan^1^, Deraniyagala Rohan^1^, Xuanfeng Ding^1^

##### ^1^Beaumont Health System, Proton Treatment Center, Royal Oak, USA

**Purpose:** Proton dose distributions of sinonasal cancer patients are sensitive to daily nasal cavity fillings variation and tumor shrinkage. With CBCT increasingly available, we hypothesized the treatment dose can be accurately reconstructed from CBCT using a deep-learning model for sinonasal patients.

**Materials/Methods:** Thirty-five pairs of pre-treatment CT and first-fraction CBCT were selected for training. A Generative-Adversarial-Networks(GAN) model, including one generator with Residual-Unet architecture, and one discriminator using a multi-layer convolutional-neural-net(CNN), was trained from scratch to predict CT from CBCT, with 3D B-spline random deformation as data augmentation. Five additional sinonasal patients with the same-day replan CT and CBCT were selected for dosimetric evaluation. The re-plan CTs were deformable registered to the same-day CBCT and served as the ground-truth (GT-CT). The synthetic CTs (Syn-CT) predicted by the trained model were compared to the GT-CT in Mean-Absolute-Error (MAE). The clinical plans were re-calculated on both Syn-CT and GT-CT using a commercial Monte-Carlo algorithm with 0.5% uncertainty. Clinically relevant DVH parameters and γ passing-rates were utilized to quantify the dosimetric discrepancy.

**Results**: The prediction time is within seconds. The MAE, γ passing rates, and DVH discrepancy between syn-CT and GT-CT were listed in Fig.1. The GT-CT, Syn-CT, and CBCT of an example patient and its dose distribution were shown in Fig.2.

**Conclusions**: The Synthetic CT predicted by the GAN model achieved clinically acceptable dosimetric accuracy as evaluated by the same-day replan CT. The method could be utilized to monitor treatment dose online and trigger plan adaptation for patients with an undesired dosimetric variation.

## O053 -

### Daily quality assurance trend analysis in pencil beam scanning FLASH proton therapy system for first in-human clinical trial

#### Joseph Speth^1,2^, Eunsin Lee^1,2^, Zhiyan Xiao^1,2^, Yongbin Zhang^1,2^, Mascia Anthony^1,2^

##### ^1^University of Cincinnati Medical Center, Proton Therapy Center, Cincinnati, USA ^2^Cincinnati Children's Hospital Medical Center, Proton Therapy Center, Cincinnati, USA

**Purpose:** In support of the ongoing *Feasibility Study of FLASH Radiotherapy for the Treatment of Symptomatic Bone Metastases (FAST-01)* clinical trial a daily quality assurance (QA) protocol was implemented to ensure safe delivery of pre-defined pencil beam scanning FLASH treatment plans. Data collected over a two-month period was analyzed to confirm the accuracy and constancy of the FLASH proton system.

**Methods:** A set of pre-defined 250 MeV transmission fields were delivered. The absolute dose measurements were performed at 5 cm water equivalent depth with an ADCL calibrated parallel plate ion chamber and electrometer. Cross-calibrated EBT3 GafChromic film was irradiated at the same depth, scanned on an Epson 11000XL scanner, and analyzed using OmniPro I'mRT software for 2D dose profiles. Field size was defined by the full width at half maximum, flatness by the variation over mean value within the central 80% of the field size, and symmetry by the largest point difference over the central 80% of the field.

**Results:** All measurements were within appropriate clinical tolerances. The average value of absolute dose measurements was 8.49±0.07 Gy. The mean dose rate was 55.7±2.4 Gy/s. Field size measurements showed constancy within a ±0.1 cm range from baseline. Flatness and symmetry measurements were all <5% and within ±3% from the baseline.

**Conclusions:** Daily QA trend analysis demonstrated that FLASH pencil beam scanning proton fields can be delivered with accuracy and stability in support of the first human clinical trial.

**Acknowledgements:** Support and funding from Varian Medical Systems

## O054 -

### The impact of rotational transformation in evaluating proton QA-CT dose

#### Lei Hu^1^, Francis Yu^1^, Haibo Lin^1^

##### ^1^New York Proton Center, Department of Physics, New York, USA

**Background**: To evaluate the dose deviations caused by rotations between QA-CT and Planning CT in Eclipse when image registration corrects the rotation between the two image sets, but the treatment planning system ignores the rotational corrections in the forward dose calculation for evaluation plans. A phantom study was carried out to investigate this deficiency.

**Materials and Methods**: A head phantom was scanned to generate a proton plan on two CTVs (2.5cm diameter and 5cm apart). The Isocenter is set in CTV1. The phantom was re-scanned with 10° rotations in roll, pitch and yaw respectively, and these QA-CT images were successfully registered to the Plan-CT for forward dose calculation and evaluation (Eval-plans1). The DVHs were compared between the Eval-Plans and the nominal Plan regarding the targets' coverages using the metric of D95%. The image registration between the QA-CT and the Plan-CT was also exported to the Velocity Imaging System (Varian) for resampling and the Eval-Plans2 were generated on the resampled QA-CT images for a separate DVH comparison.

**Results**: Losses of target coverage were found for CTV2, but not for CTV1 in the Eval-Plans1, with D95% reduced from 100% to 89% (roll), 76.3% (pitch) and 70% (yaw), respectively. The target coverage remains the same in the resampling employed Eval-Plans2.

**Conclusion**: Eclipse doesn't properly consider rotation corrections between QA-CT and Plan-CT in generating evaluation plans, which can result in incorrect dose deviation. This deficiency can be solved by resampling the registered QA-CT externally and sending it back to Eclipse for dose calculation.

## O055 -

### Slice2Volume: A registered dataset of multimodal medical imaging and light microscopy data in irradiation-injured brain tissue

#### Johannes Müller^1,2^, Theresa Suckert^1,3^, Elke Beyreuther^1,4^, Moritz Schneider^1^, Marc Boucsein^5^, Elisabeth Bodenstein^1^, Liane Stolz-Kieslich^1^, Mechthild Krause^1,2,3,6,7^, Cläre von Neubeck^1,3,8^, Robert Haase^9^, Armin Lühr^1,2,3,10^, Antje Dietrich^1,3^

##### ^1^Faculty of Medicine and University Hospital Carl Gustav Carus- Technische Universität Dresden- Helmholtz-Zentrum Dresden - Rossendorf, OncoRay – National Center for Radiation Research in Oncology, Dresden, Germany ^2^Helmholtz-Zentrum Dresden-Rossendorf, Institute of Radiooncology - OncoRay, Dresden, Germany ^3^German Cancer Consortium DKTK- and German Cancer Research Center DKFZ, Partner Site Dresden, Heidelberg, Germany ^4^Helmholtz - Zentrum Dresden - Rossendorf, Institute of Radiation Physics, Dresden, Germany ^5^German Cancer Research Center DKFZ- and Department of Radiation Oncology- Heidelberg University Hospital, Clinical Cooperation Unit Radiation Oncology, Heidelberg, Germany ^6^German Cancer Research Center DKFZ- Heidelberg- Germany- Faculty of Medicine and University Hospital Carl Gustav Carus- Technische Universität Dresden- Dresden- Germany- and- Helmholtz Association / Helmholtz-Zentrum Dresden - Rossendorf HZDR, National Center for Tumor Diseases NCT- Partner Site Dresden, Dresden, Germany ^7^Faculty of Medicine and University Hospital Carl Gustav Carus- Technische Universität Dresden, Department of Radiotherapy and Radiation Oncology, Dresden, Germany ^8^University Hospital Essen- University of Duisburg-Essen, Department of Particle Therapy, Essen, Germany ^9^TU Dresden, DFG Cluster of Excellence Physics of Life, Dresden, Germany ^10^Faculty of Physics- TU Dortmund University, Medical Physics and Radiotherapy, Dortmund, Germany

Recent years have shown that particle therapy offers highly conformal brain irradiation and optimized healthy tissue sparing. Nevertheless, elevated dose levels in healthy tissue, particularly in the distal beam region, can lead to undesirable long-term side effects. The biological mechanisms of such effects, however, remain unclear. A major obstacle towards correlating effects on clinical and cellular imaging levels is the mapping of radiation dose to specific brain regions or individual cell populations. We present a publicly available dataset of registered, multimodal imaging data of nine mice that received proton brain irradiation of different doses in a clinically relevant setting. It is available open access (https://rodare.hzdr.de/record/801) and comprises a baseline computed tomography (CT) scan, simulated distributions of dose and linear energy transfer, a co-aligned mouse brain atlas as well as magnetic resonance imaging (MRI) follow-up of up to six months. Additionally, we provide registered histological brain sections with eight histological stainings, reflecting all major cell types in adult mice brains. We used the self-developed tool Slice2Volume together with existing methods (Elastix and Big Warp) to fuse image data. The software is available open source: https://github.com/jo-mueller/Slice2Volume The provided image data spans several orders of magnitude of scale. Images of all modalities can be freely overlaid for every mouse as is demonstrated on Figure 1. This, for instance, allows tracing MRI image changes to specific cell populations. Hence, the dataset enables direct correlations and mechanistic observations regarding effects of proton radiation on the anatomical (atlas), clinical (MRI) and microscopic level (histology).

## O056 -

### Ultra-high dose rate proton radiobiology at the Dresden platform for high dose-rate radiobiology

#### Elke Beyreuther^1,2^, Michael Brand^3^, Katalin Hideghéty^4,5^, Steffen Löck^6,7,8^, Josefine Metzkes-Ng^1^, Jörg Pawelke^2,6^, Jens Pietzsch^9^, Joao Seco^10,11^, Ulrich Schramm^1^

##### ^1^Helmholtz-Zentrum Dresden-Rossendorf, Institute of Radiation Physics, Dresden, Germany ^2^Oncoray - National Center for Radiation Research in Oncology, Laser-Radiooncology, Dresden, Germany ^3^Center for Regenerative Therapies Dresden CRTD, Patterning and Regeneration of the Vertebrate Brain, Dresden, Germany ^4^University of Szeged, Oncotherapy Department, Szeged, Hungary ^5^ELI-ALPS, ELI-HU Non-Profit Ltd, Szeged, Hungary ^6^Helmholtz-Zentrum Dresden-Rossendorf, Institute of Radiooncology - OncoRay, Dresden, Germany ^7^Faculty of Medicine and University Hospital Carl Gustav Carus- Technische Universität Dresden, Department of Radiotherapy and Radiation Oncology, Dresden, Germany ^8^German Cancer Consortium DKTK- Partner Site Dresden- and German Cancer Research Center DKFZ- Heidelberg, Modeling and Biostatistics in Radiation Oncology, Dresden, Germany ^9^Helmholtz-Zentrum Dresden-Rossendorf, Institute of Radiopharmaceutical Cancer Research, Dresden, Germany ^10^German Cancer Research Center DKFZ, Division of Biomedical Physics in Radiation Oncology-, Heidelberg, Germany ^11^Ruprecht-Karls-University Heidelberg, Faculty of Physics and Astronomy, Heidelberg, Germany

The recent rediscovery of the “Flash-effect” revived the interest in high dose-rate radiation effects throughout the radiobiology community, promising protection of normal tissue, while simultaneously not altering tumour control. Several preclinical and clinical studies are presently dedicated to electron FLASH with Linacs. However, the mechanisms of Flash is still unresolved albeit its clear impact in tissue sparing to radiation toxicity. For protons, the Flash effect was confirmed in a few animal experiments using the beam parameters available at clinical cyclotrons. Laser-driven proton accelerators offer the possibility to extend the range of proton beam parameters to higher dose rates enabling the investigation *in vivo* of high dose-rate effects for up to 10^9 Gy/s. The general applicability of these beams for radiobiological studies was proven with simple cellular models and zebrafish embryos. One-step further, systematic *in vivo* experiments were prepared with a proof-of-principle mouse irradiation campaign at the Draco laser accelerator and, for comparison, at the University Proton Therapy Dresden (UPTD). Moreover, to investigate the interplay of oxygen tension, oxygen consumption and proton dose rate in more detail, an experiment with zebrafish embryos was performed at both proton sources. At the conference, we will give an overview of our radiobiological experiments performed at both DRACO and Oncoray/UPTD, for different dose rates and varying oxygen pressures.

## O057 -

### Photosensitive drug activation by accelerated protons, with concomitant generation of fluorescence and singlet oxygen: the emergence of Protondynamic therapy

#### Mantas Grigalavicius^1^, Maria Mastrangelopoulou^1^, Tine Raabe^1^, Mathilde Menard^1^, Delmon Arous^2^, Efim Bondz^2^, Sunniva Siem^2^, Andreas Görgen^2^, Nina Edin^2^, Eirik Malinen^2^, Kristian Berg^1^, Theodossis Theodossiou^1^

##### ^1^Institute for Cancer Research- Oslo University Hospital, Department of Radiation Biology, Oslo, Norway ^2^University of Oslo, Department of Physics, Oslo, Norway

Deep lying cancers like the brain cancer *glioblastoma multiforme* (GBM) are difficult to reach and practically incurable by the current standard-of-care. Even though *photosensitive drugs* (PSs) are used for fluorescence-guided resection of GBM, photomedical treatments like *photodynamic therapy* (PDT) or *photochemical internalization* (PCI) are limited by the depth of light penetration into tissue. *Proton radiotherapy* is selective for cancer and can reach deep-lying disease. Here we show the results of radical hybridization of the principles behind PDT and proton therapy. Our hypothesis was that accelerated protons can excite the electrons of PSs so that the latter can be activated into generating singlet oxygen and thus eliminating cancers like GBM. Indeed, we irradiated PS solutions and gels and verified the PS proton-activation, by fluorescence, i.e. radiative de-excitation of the photosensitive molecules (Fig. 1). Furthermore, we verified the population of PS triplet states in dry gels, and subsequently, we registered the production of singlet oxygen, either directly through its characteristic luminescence at 1270 nm or indirectly through the formation of singlet-oxygen-associated photoproducts. Following proof of principle in solutions and gels we proceeded with testing our hypothesis in GBM cell cultures: M059K, T98G and U87, by comparing the survival in cell groups ±PS (cercosporin), irradiated with various proton doses (2-20 Gy). The results revealed increased cell death with the use of PSs in M059K and T98G cells (fig. 2), but not in the U87 cell line.

## O058 -

### Towards a renewed use of “very” heavy ions for therapy: Neon minibeam radiation therapy

#### Yolanda Prezado^1^, Ryochi Hirayama^2^, Naruhiro Matsufuji^2^, Taku Inaniwa^2^, Judith Bergs^3^, Olivier Seksek^4^, Dalila Labiod^5^, Annaig Bertho1, Ibrahim Yousef^6^, Immaculada Martinez-Rovira^7^, Takashi Shimokawa2

##### ^1^CNRS-Institut Curie, Signalisation- radiobiology and cancer- UMR 3347, Orsay, France ^2^National Institute of Radiological Sciences (NIRS) - Quantum Medical Science Directorate- National Institutes for Quantum and Radiological Science and Technology (QST) , Department of Charged Particle Therapy Research, Chiba, Japan ^3^Charité Universitätsmedizin, Research Training Group BIOQIC, Berlin, Germany ^4^CNRS, IJCLAB- University Paris-Saclay, Orsay, France ^5^Institut Curie, Translational research, Orsay, France ^6^ALBA synchrotron, MIRAS beamline, Barcelona, Spain ^7^Universidad Autonoma de Barcelona, Fisica, Barcelona, Spain

Heavy ions, like Neon or Argon, offer a reduced oxygen effect, which is an advantage for the treatment of hypoxic tumors. The adverse normal tissue response observed in pioneering heavy ion therapy clinical trials in the 70s [1] led to the discontinuation of their use for therapy. Minibeam radiation therapy [2] shows highly promising reduction of toxicity to normal tissue, so that potentially heavy ions, with extremely high LET, might become applicable to clinical situations. To validate our hypothesis, the legs of C57BL/6 mice were irradiated with 230 MeV/A Ne beams at HIMAC/NIRS [3], and the skin response was evaluated. Three groups of animals were considered: i) one group received broad beam (BB) conventional irradiation (20 Gy, N=8); ii) a second group received Ne MBRT (mean dose 20 Gy, peak dose 60 Gy, N=8); iii) a third group received Ne MBRT with peak dose equal 20 Gy (N=8). The animals were followed for 4 weeks. Two weeks after irradiation 6 out of 8 animals in the BB group had to be sacrificed as they reached damage scores 4-5 (ulceration, necrosis), while the animals receiving Ne MBRT exhibited only mild dermatitis (damage score 1-1.5), with an almost total recovery 4 weeks after irradiation. The histology evaluations have confirmed those observations.These results suggest a net reduction of toxcity after Ne MBRT and support a re-exploration of heavy ions for a renewed use in future therapy.

## O059 -

### Effect of carbon ion radiotherapy on the immune systems of prostate cancer patients

#### Qi Yu^1^,^2^, Yiyang Li^3^, Xue Chen^4^, Ping Li^4^, Lin Lin^1^,^2^, Fengjiao Zhang^1^,^2^, Xianting Ding^3^, Shen Fu1,^2^,^5^

##### ^1^Proton & Heavy Ion Medical Center, State Key Laboratory of Radiation Medicine and Protection- School of Radiation Medicine and Protection- Soochow University, Suzhou, China ^2^Department of Radiation Oncology, Shanghai Concord Cancer Hospital, Shanghai, China ^3^School of Biomedical Engineering, Institute for Personalized Medicine- Shanghai Jiao Tong University, Shanghai, China ^4^Department of Radiation Oncology, Shanghai Proton and Heavy Ion Center- Fudan University Cancer Hospital, Shanghai, China ^5^Key Laboratory of Nuclear Physics and Ion-Beam Application MOE, Fudan University, Shanghai, China

Radiotherapy can change the distribution of some immune cell subsets and immune cell functional states in cancer patients. Although carbon ion radiotherapy (CIRT) has a high local tumor control rate, in most cases, radiotherapy must be combined with systemic therapy to control metastasis and increase survival. Therefore, it is necessary to systematically study the immune effects of CIRT. Here we used single-cell mass cytometry to test immune cell subsets in the peripheral blood of prostate cancer (PCa) patients before and after CIRT. At the same time, we used mass spectrometry to detect the plasma proteomics of the corresponding patients. The changes and mechanism of immune cell subsets in patients after CIRT were studied from a multi-omics perspective. Studies on more than 30 T cell subsets have found that CIRT can affect the distribution ratio of multiple immune cell subsets and the expression of immune functional proteins, such as the expression of T regulatory cells, PD1 and PDL-1. It indicates that multiple immune indexes are closely related to the therapeutic effect of carbon ion radiotherapy. This study demonstrated for the subgroup marker spectra of T cells in PCa patients before and after CIRT, providing an important reference for combined immunotherapy with CIRT.

## O060 -

### The FLASH factor of pencil beam scanning proton FLASH in a mouse model of acute skin damage

#### Brita Sørensen^1^, Mateusz Mateusz Sitarz^1^, Christina Ankjærgaard^2^, Jacob Johansen^1^, Claus E Andersen^2^, Eleni Kanouta^1^, Cathrine Overgaard^3^, Cai Grau1, Per Poulsen^1^

##### ^1^Aarhus University Hospital, Danish Center for Particle Therapy, Aarhus N, Denmark ^2^DTU, Health Tech, Roskilde, Denmark ^3^Aarhus University Hospital, Department of Experimental Clinical Oncology, Aarhus N, Denmark

**Introduction:** Preclinical studies indicate a normal tissue sparing effect using ultra-high dose rate (FLASH) radiation with comparable tumor response. The aim of the present study was to validate the effect of proton FLASH delivered with a scanning pencil beam in a mouse leg model of acute skin damage, and to quantify the normal tissue sparring factor, the FLASH factor, through full dose response curves.

**Materials and Methods:** The right hind limb of CDF1 mice were irradiated with a single fraction of protons at either conventional or FLASH dose rate in the entrance of a pencil beam scanning proton beam. Conventional dose rate was 0.4 Gy/sec (field dose rate), 244 MeV. FLASH dose rate was 69.7-88.7 Gy/s (Field dose rate), 250 MeV. In total, 292 mice were irradiated in four separate experiments. The endpoint was acute moist desquamation to the skin of the foot within 25 days post irradiation.

**Results:** The MDD50 (dose causing skin toxicity in 50% of mice) values with 95% confidence interval acute damage to the skin at conventional dose rate was 33.7Gy (32.8-34.5) and FLASH dose rate it was 48.4Gy (47.2-49.8). The resulting normal tissue sparring factor (ratio of MDD50 for FLASH dose rate to conventional dose rate) was 1.44 (1.36-1.52).

**Conclusions:** This study validates the normal tissue sparing effect of PBS proton FLASH. Full dose response curves for acute skin damage in a mouse leg model were obtained, which enabled the quantification of the normal tissue sparing factor for proton FLASH.

**Acknowledgements**: This study was supported by Varian.

## O061 -

### Patient and machine specific evaluation of intensity-modulated proton therapy (IMPT) for thoracic indications with large motion

#### Cássia O. Ribeiro^1^, Erik W. Korevaar^1^, Sabine Visser^1^, Johannes A. Langendijk^1^, Robin Wijsman^1^, Arturs Meijers^1^, Antje Knopf^1^, Stefan Both^1^

##### ^1^University Medical Center Groningen, Department of Radiation Oncology, Groningen, Netherlands

**Purpose:** Clinical benefits are anticipated when treating thoracic indications with IMPT. However, in cases of large motion amplitudes, the current concern of plan robustness hampers its clinical implementation. We present a comprehensive 4D robustness evaluation of IMPT for tumours with motion over 10mm, based on patient 4DCTs and machine log files.

**Materials and Methods:** For 9 lung and 1 thymoma cancer patients, planning and weekly verification 4DCTs were collected. Point maximum CTV motion on the planning 4DCT was extracted for all patients (Table 1). Layered rescanned (x5) 3D robust optimised IMPT (IMPT_3D) plans were generated on the averaged planning 4DCT, and approved clinically, for all patients. All plans were delivered in dry runs at our proton facility to obtain log files, and subsequently evaluated through our 4D robustness evaluation method (4DREM) on the end-exhale planning CT phase. With this method, for each evaluated plan, 14 4D accumulated scenario doses were obtained, representing 14 possible fractionated treatment courses.

**Results:** Target coverage obtained with the 4DREM was consistent with the nominal dose distribution (Fig. 1A). For most patients, robustness in terms of target homogeneity (D2-D98(CTV)) was slightly worse, compared to the nominal values (Fig. 1B.I). Additionally, averaged Dmean(lungs-GTV) and Dmean(heart) over all 4DREM scenarios changed only slightly (maximum(SD) = 0.59GyRBE), and were comparable to the respective nominal plan (Fig. 1B.II).

**Conclusion:** Rescanned IMPT_3D was shown to be robust and clinically suitable to treat thoracic tumours in free breathing for motion up to 16mm. Nevertheless, accurate patient positioning and recurrent volumetric imaging remain compulsory.

## O062 -

### Lung proton treatment for moving targets: planning strategy and 4D robust evaluation

#### Vicki Taasti^1^, Djoya Hattu^1^, Femke Vaassen^1^, Richard Canters^1^, Marije Velders^1^, Jolein Mannens^1^, Judith van Loon^1^, Ilaria Rinaldi^1^, Mirko Unipan^1^, Wouter van Elmpt^1^

##### ^1^Department of Radiation Oncology MAASTRO, GROW – School for Oncology- Maastricht University Medical Centre+, Maastricht, Netherlands

**Purpose:** Intensity-modulated proton therapy for lung tumors with large tumor movement is challenging. Often a 5mm tumor movement cut-off is used for proton patient selection. We propose a robust and easily implementable treatment planning strategy for lung tumors moving more than 5mm, and a 4DCT-robust evaluation strategy.

**Methods:** We created an internal target volume (ITV) based treatment planning strategy. The plans were robustly optimized on the 4DCT average-CT. Clinical plan acceptability was judged on robust evaluation, computing voxel-wise minimum and maximum (VWmin/VWmax) doses over 28 error-scenarios on the average-CT. We developed a 4D-robust evaluation (4DRobAvg). The 28 scenario-doses were computed on all eight 4DCT-phases. For each scenario, the doses on the individual phases were deformed to the reference phase and combined to an average scenario-dose. From these, VWmin/VWmax were computed. 4DRobAvg was compared to two other 4D-evaluations: re-computing nominal dose on each 4DCT-phase (4DNom), and computing robust VWmin/VWmax-doses on each phase (4DRobInd). This planning and evaluation strategy was validated for sixteen lung patients with tumor movement up to 26mm in RayStation (RaySearch Laboratories, Stockholm, Sweden) for our Mevion S250i Hyperscan system (Mevion Medical Systems, Littleton, MA).

**Results:** Clinically acceptable plans were feasible for all patients. The 4DNom and 4DRobInd were found to under- or overestimate the dosimetric effect of tumor movement on OARs and targets.

**Conclusion:** The proposed ITV-based planning strategy was found clinically feasible with adequate tumor coverage and no OAR over-dosage even for large tumor movement. 4DRobAvg evaluation was shown to give an easily interpretable understanding of the effect of respiratory motion on the dose.

## O063 -

### Validation of proton multiCT optimization treatment planning approach for hypofractionated pancreatic tumor cancer with use of the 4dCT

#### Joanna GORA^1^, Piero Fossati^1^, Antonio Carlino^1^, Piotr Andrzejewski^1^, Stock Markus^1^

##### ^1^MedAustron Ion Therapy Center, Medical Department, Wiener Neustadt, Austria

Since 2019, hypofractionated radiotherapy for locally advanced pancreatic cancer has been introduced at MedAustron. High dose per fraction(5x7.5Gy(RBE)), tumor localization, its relation to OARs and organ motion/filling brought a number of treatment planning related technical issues (4dCT protocols for proton dose calculation have been commissioned yet). Previous planning strategy was based entirely on static planning CT(pCT) reimported to the TPS (RayStationV8B, RaySearch Laboratories) thrice. The estimated organ motion/filling was simulated on extra CTs (overwritten with air/soft tissue creating airCT and tissueCT, respectively). Proton dose was calculated on the pCT with multiCT optimization including airCT and tissueCT (accounting for the worst-case-scenario motion and OAR filling). Once 4dCT protocols were commissioned, direct proton dose computation on 4dCT was possible, including different breathing phases for multiCT optimization. The purpose of this study was to validate both planning approaches, simplify clinical workflow and identify current issues related to new planning strategy. After, evaluating both planning approaches for 5 patients, we concluded that the pCT optimized strategy was sufficient to account for the OAR motion and filling, however comparing to the 4dCT optimization, workflow was less efficient and more time consuming. On the other hand, when evaluating 4dCT optimized approach, we realized that using only 2 most extensive breathing phases was as precise as optimization on 5 or even 8 breathing phases CTs(fig1). Therefore, computation time and number of scenarios could be much reduced. Additionally, using 4dCT was good enough to account for organ motion, but not for organ filling variations, therefore we still practice air/tissue overwrite.

## O064 -

### Helium ion imaging to improve particle therapy accuracy

#### Lennart Volz^1^, Esther Bär^2^, Charles-Antoine Collins-Fekete^2^, Armin Runz^3^, Stephan Brons^4^, Robert P. Johnson^5^, Christina Sarosiek^6^, Reinhard W. Schulte^7^, Joao Seco^1^

##### ^1^German Cancer Research Center, Biomdeical Physics in Radiation Oncology, Heidelberg, Germany ^2^University College London, Department of Medical Physics and Biomedical Engineering, London, United Kingdom ^3^Deutsches Krebsforschungszentrum Heidelberg, Medical Physics in Radiation Oncology, Heidelberg, Germany ^4^Universitätsklinikum Heidelberg, Heidelberg Ion-Beam Therapy Center, Heidelberg, Germany ^5^University of California at Santa Cruz, Department of Physics, Santa Cruz, USA ^6^Northern Illinois University, Department of Physics, DeKalb, USA ^7^Loma Linda University, Department of Basic Sciences- Division of Biomedical Engineering Sciences, Loma Linda, USA

Particle imaging promises improved relative stopping power (RSP) accuracy compared to current CT methods at better dose efficiency. Helium ion CT (HeCT) could offer an ideal balance between spatial resolution, noise and dose. Here, we present a thorough experimental evaluation of the image quality of HeCT. The US pCT collaboration prototype scanner was installed at the Heidelberg Ion-Beam Therapy Center (HIT). Experimental HeCTs were acquired in a step-and-shoot mode from 360 projections (1° steps) acquiring ∼3x10^6^ particles each. The RSP accuracy of HeCT was investigated for the tissue equivalent plastic inserts of a Catphan© CTP404 module. The spatial resolution was assessed using a sharp edge gradient technique on the plastic cube inserts of a custom phantom (20cm diameter). A phantom comprising a pig head sample was scanned with HeCT, proton CT (pCT, 180 projections) and single-/dual-energy x-ray CT (SECT/DECT) for comparison.

The mean RSP accuracy of HeCT was 0.24±0.65%. The spatial resolution was 6.5±0.5lp/cm in the middle of the cube phantom (Figure 1). HeCT and DECT of the tissue phantom agreed well with each other, with mean difference in a central line profile being -0.16±6.9%. pCT did not accurately resolve small bone features compared to DECT (Figure 2). Only minor artifacts were present in the HeCTs. Image dose for HeCT was 7 times lower than for DECT/SECT. In conclusion, HeCT yields high RSP accuracy and spatial resolution at lower dose compared to SECT/DECT. Evaluation of the range accuracy with each method is ongoing. Future clinical prospects of HeCT will be discussed.

## O065 -

### First experiments with radioactive carbon beams at GSI for the BARB project (Biomedical Applications of Radioactive ion Beam)

#### Daria Boscolo on behalf of the BARB collaboration^1^

##### ^1^GSI Helmholtzzentrum für Schwerionenforschung, Biophysics, Darmstadt, Germany

Thanks to the favourable depth-dose distribution and the radiobiological properties of heavy ion radiation, charged particle therapy (CPT) shows an improved success/toxicity ratio compared to conventional radiotherapy. The sharp dose gradients and very high doses in the distal ends, which represent the larger physical advantage of CPT, make it also extremely sensitive to range uncertainties, thus limiting its application. To overcome this limitation in the current practice, the tumor coverage is guaranteed by adding a significant margin to the target volume. The use of radioactive ion beams would potentially be ideal for simultaneous treatment and accurate online range verification by means of PET imaging. Positron emitting ions have been already studied for therapy applications demonstrating an improved image quality compared to stable ions. However, their challenging production and difficulties in reaching high intensities, have discouraged their clinical application. In this context, the project Biomedical Applications of Radioactive ion Beams (BARB) started at GSI with the main goal to assess the technical feasibility and investigate possible advantages 10,11C and 14,15O beams. The first experimental campaign took place early this year at the fragment separator (FRS) facility of GSI. 10,11C ion beams have been produced with intensities of 1E6 and 1E7 particles per second, respectively. High quality depth dose profile measurements of 12C and radioactive 11C and 10C have been measured in water together with a series of PET images in PMMA phantom. In this contribution, the project together with the first experimental results will be here presented.

## O066 -

### Cone-beam CT image correction vs deformed planning CT for head and neck proton therapy adaptation evaluation

#### Jun Zhou^1^, Sebastian Andersson^2^, Rasmus Nilsson^2^, Katja Langen^1^, Soumon Rudra^1^, Xiaofeng Yang^1^, Liyong Lin^1^, Pretesh Patel^1^, Jeffery Bradley^1^, Sagar Patel^1^, Mark W. McDonald^1^, Tian Liu^1^

##### ^1^Emory University, Radiation Oncology, Atlanta, USA ^2^RaySearch Laboratories AB, RaySearch, Stockholm, Sweden

**Purpose:** We investigate the accuracy of online evaluation using a cone-beam CT (CBCT) image correction and conversion method vs a deformed planning CT (vCT) method in head and neck (HN) proton therapy.

**Methods:** Nine previously treated proton HN patients with same-day CBCT and quality assurance CT (QACT) were evaluated. For corrected CBCT (cCBCT), joint histograms are used to convert the HU of the CBCT images, and correction maps are created to correct low-frequency artifacts. The vCT is created by deforming the TPCT to the CBCT and air differences are replaced with values from the cCBCT. Both cCBCT and vCT images were generated using a research version of the RayStation 10B planning system. Deformable registrations were created in both forward (TPCT->CBCT) and reverse (CBCT->TPCT) directions, resulting cCBCT1, vCT1, and cCBCT2, vCT2 images, respectively. The QACT is deformed to the cCBCT1 and used as a reference (dQACT). All contours on the TPCT were deformed to the cCBCT1 and then rigidly copied to the rest of the images. Original plans were applied to all the images, and the dosimetric parameters including CTV_D95, plan maximum dose (Dmax), parotid mean dose, oral cavity mean dose, and cord max dose are compared to those from the reference. ANOVA analysis was conducted for all the dosimetric parameters.

**Results:** The CTV_D95 and Dmax from generated images are almost identical to those from the reference dQACT. There are no clinically significant differences for the other dosimetric parameters.

**Conclusion:** All generated images provide accurate dosimetric evaluations with both deformable registration directions.

## O067 -

### An experimental validation of a filtering approach for prompt gamma prediction in a proton treatment planning system

#### Ze Huang^1^, Liheng Tian^1^, Guillaume Janssens^2^, Julien Smeets^2^, Yunhe Xie^3^, Boon-Keng Kevin Teo^4^, Rasmus Nilsson^5^, Erik Traneus^5^, Katia Parodi1, Marco Pinto^1^

##### ^1^Ludwig-Maximilians-Universität München, Department of Medical Physics- Faculty of Physics, Garching b. München, Germany ^2^Ion Beam Applications SA, Ion Beam Applications SA, Louvain-la-Neuve, Belgium ^3^Massachusetts General Hospital, Department of Radiation Oncology, Boston MA, USA ^4^University of Pennsylvania, Department of Radiation Oncology, Philadelphia PA, USA ^5^RaySearch Laboratories AB, RaySearch Laboratories AB, Stockholm, Sweden

A typical clinical workflow for prompt gamma (PG) range verification compares a detected PG profile with a predicted one. Monte Carlo simulations are often used for the prediction, but they are computationally demanding and may require dedicated staff. Recently, a fast novel analytical PG prediction algorithm based on the so-called filtering formalism has been proposed and implemented in a research version of the treatment planning system RayStation (RaySearch Laboratories AB). In this work, the said approach is validated against experimental data measured in an anthropomorphic phantom by a knife-edge slit camera at the Roberts Proton Therapy Center (Philadelphia, USA), and is compared to another well-established PG prediction algorithm implemented in REGGUI [1]. Furthermore, an innovative workflow, based on several PG profile quality criteria, and a sensitivity and specificity analysis are proposed for the analysis of measured and calculated data without neighbouring-spot aggregation. This workflow, adapted to features of the considered PG camera, allows to first select PG profiles exhibiting higher data reliability under realistic experimental conditions, and to then assess the data on their merits to provide higher confidence regarding the detection of PG signal shifts, thus improving the quality assurance of the treatment leveraging the PG radiation. As computed by the workflow, the mean shifts between the experimental data and the simulated PG detection by the two algorithms are estimated to be -1.2±2.0 mm and -2.4±1.6 mm for the filtering and REGGUI prediction methods, respectively.

## O068 -

### Treatment verification with prompt-gamma-imaging: Detection of anatomical changes in prostate-cancer proton therapy

#### Jonathan Berthold^1,2^, Nick Piplack^1,3^, Erik Traneus^4^, Julian Pietsch^1,2^, Chirasak Khamfongkhruea^1,5^, Julia Thiele^6^, Tobias Hölscher^6^, Guillaume Janssens^7^, Julien Smeets^7^, Kristin Stützer^1,2^, Christian Richter^1,2,6,8^

##### ^1^OncoRay – National Center for Radiation Research in Oncology- Faculty of Medicine and University Hospital Carl Gustav Carus- Technische Universität Dresden- Helmholtz-Zentrum Dresden - Rossendorf, Dresden, Germany ^2^Helmholtz-Zentrum Dresden - Rossendorf- Institute of Radiooncology - OncoRay, Dresden, Germany ^3^Technische Universität, Dresden, Germany ^4^RaySearch Laboratories AB, Stockholm, Sweden ^5^Faculty of Medicine and Public Health- HRH Princess Chulabhorn College of Medical Science- Chulabhorn Royal Academy, Bangkok, Thailand ^6^Faculty of Medicine and University Hospital Carl Gustav Carus- Technische Universität Dresden, Department of Radiotherapy and Radiation Oncology, Dresden, Germany ^7^Ion Beam Applications SA, Louvain-la-Neuve, Belgium ^8^German Cancer Consortium DKTK- partner site Dresden- Germany and German Cancer Research Center DKFZ, Heidelberg, Germany

**Introduction:** We present results of the worldwide first systematic study on the sensitivity of prompt-gamma-imaging (PGI) to detect anatomical changes in proton therapy for the ongoing evaluation in prostate-cancer treatments.

**Materials and Methods**: Spot-wise range shifts were monitored with a PGI-slit-camera during 40 fractions of hypo-fractionated prostate-cancer treatments (5 patients, 2 fields, each 1.5GyE). In-room CTs were acquired for these fractions and range shifts of spot-wise integrated depth-dose (IDD) profiles serve as ground-truth. For both PGI and IDD data, spots were clustered based on Bragg-peak position and proton number to mitigate statistical uncertainty in the PGI measurement using a low-dose spot cut-off at 5e7 protons, a minimum number of 3e9 protons per cluster, and a minimum/maximum cluster volume of 1cm^3^/8cm^3^. Clusters with absolute range shift ≥5mm were classified as relevant anatomical changes**.**

**Results:** A strong correlation (r_Pearson_=0.72) was found between ground-truth IDD and PGI range shifts per cluster with an average absolute deviation of 1.3mm over all fractions. In total, 245/7143 (3.4%) clusters (found within 24/72 fields) contained relevant IDD-based range shifts. PGI detected these changes with a sensitivity of 68%, specificity of 96%, and accuracy of 95%. The results might be affected by potential intra-fractional changes between in-room CT acquisition and treatment delivery. A higher sensitivity is also expected for a gantry-mounted camera system with decreased positioning uncertainty.

**Conclusion:** Our systematic investigation on the sensitivity of a PGI-slit-camera with a first quantitative comparison of range shifts from PGI and IDD profiles demonstrates the capability to locally detect relevant anatomical changes in patients.

## O069 -

### Towards quantitative helium-beam radiography (αRAD) using thin silicon pixel detectors and energy painting

#### Tim Gehrke^1,2,3^, Catherine Knobloch^2,3^, Sonja Surla^2,3^, Margareta Metzner^2,3^, Florian Kehrein^2,3^, Laura Ghesquiere-Dierickx^2,3^, Christian Schömers^4^, Stefan Scheloske^4^, Stephan Brons^4^, Andreas Peters^4^, Oliver Jäkel^2,3,4^, Maria Martisikova^2,3^

##### ^1^Heidelberg University Hospital, Radiation Oncology, Heidelberg, Germany ^2^German Cancer Research Center DKFZ, Medical Physics in Radiation Oncology, Heidelberg, Germany ^3^National Center for Radiation Research in Oncology NCRO, Heidelberg Institute for Radiation Oncology HIRO, Heidelberg, Germany ^4^Heidelberg Ion-Beam Therapy Center HIT, Radiation Oncology- Heidelberg University Hospital, Heidelberg, Germany

To take full advantage of the features of ion-beam therapy, it is important to minimize uncertainties that could arise during the determination of the actual relative stopping power (RSP) of tissue or due to anatomical changes during the course of the therapy. To this end, ion-beam radiography at minimal dose exposure that is performed right before treatment could provide additional information with respect to the standardly-used imaging modalities. The major advantage is the potential for direct measurements of the integrated RSP, i.e. the water-equivalent thickness (WET) of the patient along the beam direction. In previous works a compact detection system for αRAD that exclusively contains thin silicon pixel detectors (WET<0.8mm) was built. The detection system is used with helium-beam energies exceeding the maximum therapeutic energy of 220MeV/u at the Heidelberg Ion-Beam Therapy Centre (HIT). These newly-established beams at HIT enable us to image even body regions with the largest WET—up to 550mm. In this contribution, the performance of the detection system will be presented, and the image quality concerning spatial resolution and quantitative WET precision are compared to other detection systems. A working solution for the main obstacle of using thin detectors—the limited WET-range in which high WET-precisions can be achieved—will be suggested. It involves the tailoring of initial beam energies to the expected WET of multiple smaller segments, referred to as energy painting. WET-calibrations for different initial energies that are crucial for energy painting and their application to first helium-beam radiographs of a pelvis phantom based on energy painting are presented.

## O070 -

### A high-granularity digital tracking calorimeter optimized for proton CT by the Bergen pCT collaboration

#### Pierluigi Piersimoni ^1^

##### ^1^University of Bergen, Department of Physics and Technology, Bergen, Norway

Proton CT (pCT) is an imaging technique effective in noticeably reducing hadrontherapy treatment planning uncertainties, providing direct measures of relative stopping power (RSP). The Bergen (Norway) pCT collaboration was established with the purpose of building a pCT scanner, with a segmented high-granularity digital tracking calorimeter (DTC) used both as tracker system and energy/range detector. The device is optimized for pCT, in order to handle pencil beams with high particle rate and localized dose depositions. The DTC is a multilayer structure made of 43 detector/carrier sandwich layers, the first 2 functioning as tracking system and having thin (∼200 μm) carbon-epoxy fleece carriers to minimize the scattering. For the remaining layers, 3.5 mm thick slabs of aluminum are employed as sensor carriers and beam degraders. Each sensor layer consists of 108 ALPIDE chips (ALICE, CERN) grouped in strings of 9, ultrasonically bonded onto thin traces of pure aluminum on a polyimide flex and subsequently bonded to a larger flexible printed circuit board. The read-out electronics is fast enough to acquire 2D images in few seconds. Monte Carlo simulation of the system assessed the RSP accuracy achievable with the DTC being well below 1%. Beam tests of the single ALPIDE chip and the 9-chips string have been performed in the lab and at medical beam facilities (e.g., HIT, Heidelberg, Germany). Intelligent machine learning techniques and visualization methods are employed to advance the prototypical approaches toward increased speed and safety. The pCT scanner can be employed for additional online applications during the treatment, such as in-situ proton range verification.

## O071 -

### Deep learning-based dose estimation methods for carbon ion therapy

#### Harley Rutherford^1,2^, Andrew Chacon^1,2^, Daniel Franklin^3^, Akram Mohammadi^4^, Hideaki Tashima^4^, Taiga Yamaya^4^, Katia Parodi^5^, Anatoly Rosenfeld^1,6^, Susanna Guatelli^1,6^, Mitra Safavi-Naeini^1,2,7^

##### ^1^University of Wollongong, Centre for Medical and Radiation Physics CMRP, Wollongong, Australia ^2^Australian Nuclear Science and Technology Organisation, Human Health, Lucas Heights, Australia ^3^University of Technology, School of Electrical and Data Engineering, Sydney, Australia ^4^National Institute of Radiological Sciences, National Institutes for Quantum and Radiological Science and Technology, Chiba, Japan ^5^Ludwig-Maximilians-Universitat Munchen, Department of Medical Physics- Faculty of Physics, Munich, Germany ^6^University of Wollongong, Illawarra Health and Medical Research Institute, Wollongong, Australia ^7^University of Sydney, Brain and Mind Centre, Sydney, Australia

Quantifying the dose delivered in particle therapy from the spatial distribution of positron-emitting fragments is difficult due to the complex and differing underlying physics processes creating a non-linear relationship between dose deposition and fragmentation. In recent years, deep-learning has proven to be a useful approach for a variety of non-linear problems in radiotherapy and medical imaging. This study investigates two deep-learning methods for PET-image-based dose estimations in carbon therapy: a feed-forward, and convolutional neural network (FFNN and CNN). The performance of the neural network approaches are compared to a recently published iterative optimisation technique for dose estimation, for a range of Monte Carlo simulated carbon-12 spread-out Bragg peaks (SOBPs) [1]. To quantify the performance of each method, two quantities are considered: 1) The mean relative error (MRE); and 2) the error in the calculation of the distal edge, using the d20 and d50 points. FFNN, CNN and the iterative methods were able to calculate the dose profile with an MRE of within 2.5%+/-0.5%, 2.0%+/-0.1% and 1.9%+/-0.4%, respectively. the CNN and iterative methods are able to calculate the dose profiles of 116mm SOBPs with an MRE of within 0.8%+/-0.4% and 0.9%+/-0.7%, respectively, while the feed-forward neural network produces an average mean relative error of 1.8%+/-0.7%. The distal edge of the SOBPs was estimated with an average accuracy of within 2.0mm+/-1.5mm by the FFNN, 1.5mm+/-1.6mm by the CNN, and 1.6mm+/-2.2mm by the iterative method, for all SOBP sizes.

## O072 -

### Scanned carbon-ion beam dose reconstruction based on in-room CT, patient positioning, and patient-specific treatment delivery log-files over treatment course

#### Masashi Yagi^1^, Yushi Wakisaka^2^, Masaaki Takashina^3^, Toshiro Tsubouchi^3^, Noriaki Hamatani^3^, Osamu Suzuki^4^, Yuji Seo^1^, Kazuhiko Ogawa^5^, Tatsuaki Kanai^3^

##### ^1^Osaka University Graduate School of Medicine, Department of Carbon Ion Radiotherapy, Suita, Japan ^2^Osaka Heavy Ion Therapy Center, Department of Radiation Technology, Osaka, Japan ^3^Osaka Heavy Ion Therapy Center, Department of Medical Physics, Osaka, Japan ^4^Osaka Heavy Ion Therapy Center, Department of Radiology, Osaka, Japan ^5^Osaka University Graduate School of Medicine, Department of Radiation Oncology, Suita, Japan

**Introduction**: The purpose of this work was to establish a method to reconstruct the scanned carbon-ion dose distribution delivered to a patient using the in-room CT, patient positioning and treatment delivery log-files and evaluate the validity of the method.

**Materials and Methods**: For four prostate cancer patients, in-room CT acquisition were performed on every fraction over the treatment course. The recorded scanning parameters were extracted from treatment delivery log-files by using in-house software. The dose distribution was then reconstructed on the respective in-room CT with the corresponding patient positioning information and recorded scanning parameters on a treatment planning system. The scanning parameters, reconstructed dose distribution and DVHs were compared with those of the treatment plan.

**Results**: The averaged deviation in the log-files from the planned spot position and dose was within 1.0 mm and 0.1%, respectively. The reconstructed dose distribution over the treatment course revealed that dosimetric changes due to anatomic variations caused by setup inaccuracy, or organ deformation (or a combination of these) were observed, representing dose change about -1.1% to the CTV and -1.3% to the bladder compared to the treatment plan (Figure 1).

**Conclusion**: The established methodology could have ability to evaluate dosimetric changes over treatment course. The method would afford an objective dosimetric basis for the clinical decision on a necessity of replanning during the treatment course and the clinical evaluation comparing to the treatment result and provides a valuable platform for ART in the future.

## O073 -

### A scoping review of global proton therapy patient selection methods

#### Nicole Zientara^1,2^, Eileen Giles^1^, Hien Le^1,3^, Michala Short^1^

##### ^1^University of South Australia, UniSA Cancer Research Institute, Adelaide, Australia ^2^Liverpool & Macarthur Cancer Therapy Centres, Radiation Therapy, Sydney, Australia ^3^Royal Adelaide Hospital, Radiation Oncology, Adelaide, Australia

The aim was to conduct a scoping review exploring clinical decision-making tools and dose comparison methods used globally for Proton Therapy (PT) versus Photon therapy patient selection. A literature search using established scoping review methods was performed in Medline and Embase databases as well as grey literature for articles published from January 1, 2015 to August 4, 2020. Articles were eligible for inclusion if they clearly stated methods of patient selection and were in English. In total 321 studies were identified; 49 studies met this study's inclusion criteria, representing PT patient selection from 13 countries. Of these 13 countries, only 9 of the 19 countries with PT clinically operational were represented. Six different clinical decision-making tools and 14 dose comparison methods were identified, demonstrating variability within countries and internationally. PT was indicated for all pediatric patients except those with lymphoma and re-irradiation where individualized model-based selection was required. The most commonly reported patient selection tools included Normal Tissue Complication Probability models, followed by cost-effectiveness modelling and dosimetry comparison. Model-based selection methods were most commonly applied for Head and Neck clinical indications in adult cohorts. While no “Gold Standard” currently exists for PT patient selection with variations evidenced globally, some of the patient selection methods identified in this review can be used to inform future practice in Australia and elsewhere. As literature was not identified from all countries where PT centres are available, further research is needed to evaluate patient selection methods in these jurisdictions for a comprehensive overview.

## O074 -

### The impact of implementing proton therapy as a radical innovation on healthcare operations: a quantitative study of the management perspective

#### Luca Heising^1,2^, Maria Jacobs^1,2^, Geert Bosmans^1^, Rachelle Swart^1^, Carol Ou^2^

##### ^1^Maastro Clinic, Innovation Research, Maastricht, Netherlands ^2^Tilburg School of Economics and Management, Department of Management, Tilburg, Netherlands

**Introduction**: Improvement of healthcare is largely dependent on the implementation of Radical Innovation (RI) and requires adjustments in management approach, skills and knowledge. The RI observed in this study is the implementation of proton therapy within an existing radiotherapy department. We quantity the impact of the RI by comparing with the Business as usual (BaU) from photon therapy.

**Methods**: We collected data from both modalities, including reported incidents (defined as an unforeseen event in the process), downtime, hard- and software upgrades, quality control activities, and patient satisfaction. All the data is normalized against the number of fractions.

**Results**: We found that more incidents occurred in the RI implementation than BaU. The root causes were identified more on an organizational error for proton therapy, whereas in photon therapy, a much more stable process, the root causes were more related to human errors. Additionally, implementation of the RI led to an increase in number of human root cause errors on BaU procedures. The RI did not affect the patient's satisfaction despite a higher downtime, as expected with a new technique/machine.

**Conclusion**: Conclusively, the RI implementation has an impact on all variables, some worse than others. We encountered a larger impact on the incidents than we had anticipated. Contrarily, a lesser impact on patients' satisfaction was found than initially expected. The patients were informed that the treatment was new for the organization, probably leading to a higher acceptability and more tolerant attitude for interruption of the equipment and hence longer waiting times.

## O075 -

### Transnational Access, networking and joint research for heavy ion therapy research: the HITRIplus project

#### Sandro Rossi^1^, Manuela Cirilli^2^, Manjit Dosanjh^3^, Marco Durante^4^, Angelica Facoetti^1^, Piero Fossati^5^, Christian Graeff^4^, Thomas Haberer^6^, Maria Vittoria Livraga^1^, Monica Necchi^1^, Mark Plesko^7^, Lucio Rossi^8^, Nicholas Sammut^9^, Ulrike Schoetz^10^, Maurizio Vretenar^11^

##### ^1^CNAO Foundation, National Center for Oncological Hadrontherapy, Pavia, Italy ^2^CERN, Knowledge Transfer Department, Geneva, Switzerland ^3^SEEIIST, South East European International Institute for Sustainable Technologies, Geneva, Switzerland ^4^GSI Helmholtz Centre for Heavy Ion Research, Biophysics department, Darmstadt, Germany ^5^MedAustron Ion Therapy Center, Medical department, Wiener Neustadt, Austria ^6^HIT Heidelberg Ion-Beam Therapy Center- Heidelberg University Hospital, Department of Radiation Oncology, Heidelberg, Germany ^7^COSYLAB, Control System Laboratory, Ljubljana, Slovakia ^8^INFN National Institute for Nuclear Physics, Milan Unit, Milano, Italy ^9^University of Malta, Faculty of Information and Communications Technology, Msida, Malta ^10^Philipps-University- University Hospital Giessen and Marburg, Department of Radiotherapy and Radiooncology, Marburg, Germany ^11^CERN, ATS/DO Department, Geneva, Switzerland

The Heavy Ion Therapy Research Integration *plus* (HITRI*plus*) is a project that aims to integrate and propel biophysics and medical research on cancer treatment with heavy ions beams while jointly developing its sophisticated instruments. The wider objective of HITRI*plus* is to provide radiation oncologists with a cutting-edge tool to treat the fraction of tumours that are not curable with X-rays or protons or have better survival rates or lower recurrences with ions. For this major initiative, HITRI*plus* has gathered a consortium, led by CNAO, engaging all relevant stakeholders and for the first time bringing together all four European ion therapy centres with leading EU industries, academia and research laboratories. They all share the ambition to jointly build a strong pan-European Heavy Ion Therapy Research Community. A strategic partner is the South East European International Institute for Sustainable Technologies, which federates eight countries in South East Europe with the ambition to build a next generation heavy ion Research Infrastructure in the area. HITRI*plus* Transnational Access will integrate and open to external researchers the experimental programme of the five European facilities providing therapeutic ion beams. Its Networks will structure and foster the research on heavy ion therapy, including clinical and pre-clinical research. Joint Research Activities will develop new accelerator and beam delivery technologies to extend the reach of the present generation centres and to define a new European reference design, at lower cost and dimensions, to make cancer ion therapy more accessible and to open new markets to European industry.

## O076 -

### The COVID-19 pandemic impacts to proton therapy system installation and commissioning

#### Li Shen^1^, Li Zuofeng^2^, Yuan Xiaogang^3^, Liu Shengwen^3^, Xie Qishan^3^, Li Shu^3^, Liu Bing^3^, Yu Yue^3^

##### ^1^Guangzhou Concord Cancer Center, Particle Technology Department, Guangzhou, China ^2^Guangzhou Concord Cancer Center, Radiotherapy, Guangzhou, China ^3^Guangzhou Concord Cancer Center, Particle Technology, Guangzhou, China

Guangzhou Concord Cancer Center (GCCC) is equipped with a Varian ProBeam proton therapy system with 4 gantry rooms. The major installation has been completed and the system is currently under commissioning, with the first room handover scheduled for the end of 2021. GCCC was at the stage of finalizing the building construction of the proton therapy center when the first COVID-19 pandemic outbreak arrived at the beginning of 2020. From the emergency responses at the beginning, to today's regular epidemic prevention and control, we experienced unprecedented difficulties in this year. The major challenges were the shortage of human resources and the insidious risks raised by the pandemic. The epidemic impacts to the proton system installation and commissioning mainly embody in 3 aspects: 1. Delay of the building readiness for proton equipment. 2. Delay of the equipment rigging and slowing down the whole installation/commissioning process. 3. Increase of the uncertainty and risk to the proton project. Different measures were taken to minimize the epidemic impacts: 1. Prevent the coronavirus infection for the personnel of the proton project. 2. Prepare the needs of resources ahead of time. 3. Facilitate the international travel for the key technical experts from abroad to work at hospital site. 4. Maximize system commissioning by local team with remote supports, train the local team to make them to take more responsibilities.

## O077 -

### Toxicity in patients with autoimmune diseases treated with curative particle therapy: a single institution matched case-control study

#### Giulia Riva^1^, Barbara Vischioni^1^, Sara Gandini^2^, Stefano Cavalieri^3^, Amelia Barcellini^1^, Sara Ronchi^1^, Ester Orlandi^1^

##### ^1^National Center of Oncological Hadrontherapy CNAO, Clinical Department, Pavia, Italy ^2^European Institute of Oncology- IRCCS- Milan- Italy, Department of Experimental Oncology, Milan, Italy ^3^Fondazione IRCCS Istituto Nazionale dei Tumori di Milano- Milan- Italy., Head and Neck Cancer Medical Oncology 3 Unit, Milan, Italy

**Background**. It is unclear if the presence of autoimmune diseases (ADs) may predispose to higher radiation-induced toxicity. No data are available on protons (PT) or carbon ions (CIRT) therapy. The objective of the study is to determine whether ADs patients have an increased incidence of complications after PT and CIRT for cancer.

**Methods**. Thirty-eight ADs patients were treated with PT or CIRT between 2011 and 2020. 13 patients had collagen-vascular disease (CVD), 5 an inflammatory bowel disease (IBD) diseases and 20 patients an organ-specific ADs. Each patient was matched with two control patients without ADs on basis of type/site of cancer, type of particle, age, sex, comorbidities (hypertension and diabetes), previous surgery.

**Results**. The incidence of acute G≥3 toxicity for ADs group was statistically higher than in match control group (15.8%vs2.6%, p=0.016) especially coinsidering CVD-IBD patients compared to their control group counterparts (27.7 vs 2.6% p=0.0003). There was no difference between ADs patients and controls in late G≥3 toxicity (7.9% vs 2.6%, p=0.33). Age, type of particle, radiation dose, comorbidities and previous surgery do not influence acute and late toxicity in the two groups. No G4-5 complications were reported.

**Conclusions**. A higher rate of G3 acute toxicity was found in ADs patients treated with PT and CIRT, but no G4-G5 complications are reported. The presence of ADs should not be considered an absolute contraindication to particle therapy, but careful attention is needed in the management of toxicity. Further prospective studies on particle therapy and ADs are needed.

## O078 -

### Rapid comparative planning to predict oropharyngeal toxicity reduction for proton therapy versus VMAT

#### Mark McDonald^1^, William Stokes^1^, Sibo Tian^1^, Vishal Dhere^1^, Karen Xu^1^, Neil Pfister^1^, Jill Remick^1^, Soumon Rudra^1^, James Bates^1^, Katja Langen^1^, Ryan Zielan^1^, Roelf Slopsema^1^

##### ^1^Winship Cancer Institute of Emory University, Department of Radiation Oncology, Atlanta, USA

**Purpose:** To assess the accuracy of rapid comparative planning (RCP) vs fully optimized clinical plans (OCP) using normal tissue complication probability (NTCP) models to estimate differences in clinical outcomes between proton therapy (PT) and photon radiation (RT) plans for oropharyngeal cancer.

**Materials/Methods:** We performed RCP of photon VMAT and pencil beam PT in 30 consecutive patients (pts) with oropharyngeal cancer requiring bilateral neck RT to 60-70 Gy, using published NTCP models of Grade 3+ mucositis, G3+ dysphagia, G4 xerostomia, G3+ aspiration, G2 hypothyroidism, and G2 trismus. Predicted risk reduction >10% for G2 and >5% for G3+ toxicity were considered clinically relevant. RCP used diagnostic imaging (n = 16) or simulation CT (n = 14). The latter group also had OCP VMAT and PT plans using the same CT dataset (4 plans each) and served as a validation cohort (n=14) for the RCP process and individual NTCP models.

**Results:** RCP predicted ≥1 clinically relevant toxicity reduction in all 30 pts with ≥1 toxicity threshold achieved in all validation cohort patients, yielding a PPV of 100%. Models for mucositis and trismus had the highest PPV and the xerostomia model the lowest. Patients denied insurance coverage for proton therapy had, on average, more NTCP toxicity reduction thresholds met by PT in RPC than those approved (5 vs 4, p = 0.049).

**Conclusions:** RCP incorporating NTCP-based toxicity estimation by RT modality appears to be a reliable predictor based on concordant finding of at least one clinically relevant toxicity reduction in both RCP and OCP.

## O079 -

### Accelerator-based BNCT for patients with recurrent GBM: a multicenter phase II study

#### Shinji Kawabata^1^, Minoru Suzuki^2^, Katsumi Hirose^3^, Hiroki Tanaka^2^, Takahiro Kato^3^, Hiromi Goto^4^, Yoshitaka Narita^5^, Shin-Ichi Miyatake^1^

##### ^1^Osaka Medical College, Department of Neurosurgery, Takatsuki, Japan ^2^Kyoto University, Institute for Integrated Radiation and Nuclear Science, Kumatori- Osaka, Japan ^3^Southern Tohoku Research Institute for Neuroscience, Southern Tohoku BNCT Research Center, Koriyama- Fukushima, Japan ^4^Southern Tohoku Research Institute for Neuroscience, Department of Neurosurgery, Koriyama- Fukushima, Japan ^5^National Cancer Center Hospital, Department of Neurosurgery and Neuro-Oncology, Tokyo, Japan

**Background**: Boron neutron capture therapy (BNCT) utilizes tumor-selective particle radiation. We aimed to assess the safety and efficacy of accelerator-based BNCT (AB-BNCT) using a cyclotron-based neutron generator (BNCT30) and ^10^B-boronophenylalanine (SPM-011) in patients with recurrent malignant gliomas (MGs), primarily glioblastoma (GB).

**Methods**: This multi-institutional, open-label, phase II clinical trial involved 27 recurrent cases of MG, including 24 GB cases, who were enrolled from February 2016 to June 2018. The study was conducted using the above mentioned AB-BNCT system, with 500 mg/kg of SPM-011 (study code: JG002). The patients were bevacizumab-naïve and had recurrent MG after standard treatment. The primary endpoint was 1-year survival rate, and the secondary endpoints were overall survival (OS), and progression-free survival (PFS). The results were compared to those of a previous Japanese domestic bevacizumab trial for recurrent GB (JO22506).

**Results**: The 1-year survival rate and median OS of the recurrent GB cases in the current trial was 79.2% (95% CI: 57.0–90.8) and 18.9 months (95% CI: 12.9–NE), respectively, while those of JO22506 were 34.5% (90% CI: 20.0–49.0) and 10.5 months (95% CI: 8.2–12.4), respectively. The median PFS was 0.9 months (95% CI: 0.8–1.0) by RANO criteria. The most prominent adverse event was brain edema. Twenty-one of 27 cases were treated with bevacizumab following progressive disease.

**Conclusions**: AB-BNCT demonstrated acceptable safety, and prolonged survival for recurrent MG. AB-BNCT may increase the risk of brain edema due to re-irradiation for recurrent MG; however, this appears to be controlled well with bevacizumab.

## O080 -

### BNCT for F98 glioma bearing rats using a TSPO-targeted novel boron compound

#### Hideki Kashiwagi^1^, Shinji Kawabata^1^, Yoshihide Hattori^2^, Yusuke Fukuo^1^, Takuya Kanemitsu^1^, Koji Takeuchi^1^, Ryo Hiramatsu^1^, Tsubasa Watanabe^3^, Takushi Tanaka^3^, Hiroki Tanaka^3^, minoru Suzuki^3^, Koji Ono^4^, Shin-Ichi Miyatake^4^, Mistunori Kirihata^2^, masahiko Wanibuchi^1^

##### ^1^Osaka Medical College, Neurosurgery, Takatsuki-shi, Japan ^2^Osaka Prefecture University, Research Center of Boron Neutron Capture Therapy, Sakai-shi, Japan ^3^Kyoto University, Institute for Integrated Radiation and Nuclear Science, Kumatori-cho, Japan ^4^Osaka Medical College, Kansai BNCT Medical Center, Takatsuki-shi, Japan

**Background**: Boron neutron capture therapy (BNCT) is a treatment that selectively destroys tumor cells and has been shown to be effective in clinical trials for malignant gliomas. Recently, the 18k-Da translocator protein (TSPO) targeted compounds have been shown to be highly accumulated in malignant gliomas by positron emission tomography. We thought that TSPO may be a novel target of BNCT for malignant glioma.

**Materials and Methods**: *In vitro*, the boron (^10^B) concentration of F98 glioma cells and the biological effectiveness factors specific for the irradiated tissue and the boron compound with neutron capture reaction, named CBE factor, by using boronophenylalanine (BPA), borocaptate sodium (BSH), and TSPO-targeted ^10^B compounds (DPB15) were measured. *In vivo,* biodistribution for the F98 glioma bearing rats were administered with ^10^B compounds (BPA (i.v.; intravenous administration) or DPB15 (CED; convection-enhanced delivery)) were evaluated. Neutron irradiation experiments for the F98 glioma bearing rats were performed and evaluated by Kaplan-Meier survival curves.

**Results**: *In vitro*, ^10^B concentration and CBE factor of DPB15 were highest. *In vivo*, DPB15 group showed higher tumor boron concentrations than BPA. And the F98 glioma bearing rats were divided to six groups; untreated controls, neutron irradiation only, DPB15 only, BNCT with BPA, BNCT with DPB15, and BNCT with combination of BPA and DPB15. The combination group had significant longer survival times than BPA group.

**Conclusion**: TSPO-targeted ^10^B compounds may be useful for BNCT of malignant gliomas. The combination of CED of TSPO-targeted ^10^B compounds with BPA may complement the effect of BNCT on cells that were not destroyed by BPA-BNCT.

## O081 -

### Ultra-small, luminescence, boron-based nanoparticles for boron neutron capture therapy

#### Chen Wei Chiang^1^, Yun Chen Chien^1^, Chih Yi Wang^1^, Wei-Jui Yu^2^, Liang Cheng Chien^1^, Chou Chio Lao^1^, Chi-Shiun Chiang^2^, Pei Yuin Keng^1^

##### ^1^National Tsing Hua University, Department of Materials Science and Engineering, Hsinchu, Taiwan ^2^National Tsing Hua University, Department of Biomedical Engineering and Environmental Sciences, Hsinchu, Taiwan

Herein, we demonstrated the self-assembly of ultra-small boron carbon oxy-nitride (BCNO) nanoparticles with transformable surface properties as a potential new boron drug for boron neutron capture therapy (BNCT). The nanoparticle surface is functionalized with a positively charge polymer followed by a stealthy layer of polyethylene glycol on the outer layer for enhanced tumor accumulation. Upon reaching the tumor microenvironment, the engineered polymer coating with an acid sensitive bond will break, thus releasing the toxic, and positive charge nanoparticles for effective cell penetration. Previously, our group have successfully demonstrated the synthesis and functionalization of 5 nm BCNO nanoparticles with polyethyleneimine (PEI), a positively charged polymer layer. Here we will investigate the self-assembly of ultra-small 5 nm PEI@BCNO nanoparticles with stimuli responsive polyethylene glycol outer layer for enhanced tumor accumulation via EPR effect while masking the toxicity of the cationic nanoparticles prior to entering the tumor microenvironment. We will report on our preliminary in vitro cell studies to confirm cell viability, cell uptake of BCNO with different polymer coatings, and in vitro neutron flux experiment of the new boron nanomedicine for BNCT. With the functionalization of a specific ligand for tumor targeting, this new boron drug has the potential of enhancing BNCT treatment efficacy through enhanced tumor accumulation, targeting, and solid tumor penetration.

## O082 -

### A novel BNCT compound, polymer-conjugated glucosamine complexed with boric acid shows tumor-selective accumulation and simultaneous inhibition of glycolysis

#### Yoshitaka Matsumoto^1^, Waliul Islam^2^, Yu Sugawara^3^, Tomohiro Sawa^2^, Nobusyohi Fukumitsu^4^, Hideyuki Sakurai^1^, Hiroshi Maeda^2^

##### ^1^University of Tsukuba, Faculty of Medicine Proton Medical Research Center, Tsukuba, Japan ^2^Kumamoto University, School of Medicine, Kumamoto, Japan ^3^University of Tsukuba Hospital, Proton Medical Research Center, Tsukuba, Japan ^4^Kobe Proton Center, Department of Radiation Oncology, Kobe, Japan

A unique water-soluble synthetic polymer, styrene-maleic acid copolymer (SMA) conjugated glucosamine (SG), was synthesized. It formed a stable complex with boric acid (BA). The complex had an average particle size of 15 nm by light scattering and a single peak by gel permeation chromatography. The particles were taken up by tumor cells five times faster than free BA *in vitro*, and BA was liberated at acidic tumor pH. The liberated BA inhibited glycolysis and resulted in tumor suppression *in vivo*. Intravenously injected SGB complexes bound albumin, had a plasma half-life of about 8 hours in mice, and accumulated in tumor tissue about 10 times more than in normal organs. The IC_50_ of SGB complexes in HeLa cells under 6-9% pO_2_ was about 20 μg/ml (equivalent to free BA), 150 times more potent than free BA. Neutron irradiation of human oral cancer cells with SGB complexes resulted in 16-fold more cell death than without SGB complexes. *In vivo* anti-tumor effects were evaluated after one-time neutron irradiation in SCCVII-bearing mice, and significant tumor suppression was observed. No visible sign of neutron irradiation was observed nor other systemic toxicity. These results indicate that the SGB complex is a unique multifunctional anticancer agent with much more potent activity under low pO_2_ conditions, as in large advanced cancers.

## O083 -

### Evaluation of the therapeutic efficacy of boron neutron capture therapy (BNCT) for primary central nervous system lymphoma (PCNSL)

#### Kohei Yoshimura^1^, Hideki Kashiwagi^1^, Kawabata Shinji^1^, Yusuke Fukuo^1^, Takeuchi Koji^1^, Futamura gen^1^, Hiramatsu Ryo^1^, Miyatake Shin-ichi^2^, Wanibuchi Masahiko^1^

##### ^1^Osaka Medical College, Neurosurgery, Osaka, Japan ^2^Kansai BNCT Medical Center, Neurosurgery, Osaka, Japan

**Background**: Boron neutron capture therapy (BNCT) is a nuclear reaction-based tumor cell-selective particle irradiation that occurs when nonradioactive boron-10 is irradiated with low-energy neutrons to produce high-energy α particles. Primary central nervous system lymphoma (PCNSL) accounts for 5% of all brain tumors and is classified as a WHO Grade IV. Treatment for PCNSL is based on a high dose methotrexate (HD-MTX) chemotherapy with whole brain irradiation. While the initial response rate is relatively good, the recurrence rate is high, and there is no useful treatment for PCNSL at relapse after irradiation because of toxicity. Therefore, BNCT, which is highly cell-selective, is considered to be an effective treatment for recurrent PCNSL. In order to expand the therapeutic indications, we conducted basic BNCT experiments.

**Methods**: We evaluated boron concentrations after exposure to boronophenylalanine (BPA).Also, the bio distribution study had done using lymphoma cell bearing mouse brain tumor model .These results were compared with our glioma studies.

**Results**: The boron concentrations in glioma cells and lymphoma cells after exposure to BPA were 3.27 μgB/10^7^ cells and 2.96 μgB/10^7^ cells, indicating that the lymphoma had the same boron uptake capacity as glioma. bio distribution in the mouse brain tumor model using Raji showed a tumor/normal brain ratio of 1.8 and a tumor/blood ratio of 0.3. Since lymphoma have good BPA uptake and bio distribution, we will conduct neutron irradiation experiments to expand the application of BNCT for PCNSL.

**Conclusions**: BNCT is expected to be effective for refractory PCNSL. A protocol for future clinical application is planned.

## O084 -

### BNCT mediated by BPA+GB-10 using Oligo-Fucoidan and Glutamine as adjuncts to improve efficacy and reduce radiotoxicity in colon cancer model

#### Debora N Benitez Frydryk^1^, Mónica A. Palmieri^2^, Emiliano C.C. Pozzi^1^, Silvia I. Thorp^1^, Marcela A. Garabalino^1^, Andrea Monti Hughes^1,3^, Paula Curotto^1^, Iara S. Santa Cruz^1^, Paula S. Ramos^1^, María E. Itoiz^1,4^, Amanda E. Schwint^1,3^, Veronica Andrea Trivillin^1,3^

##### ^1^Comisión Nacional de Energía Atómica, Radiobiology, San Martín, Argentina ^2^Facultad de Ciencias Exactas y Naturales- Universidad de Buenos Aires UBA- Argentina, Departamento de Biodiversidad y Biología Experimental, Ciudad Autónoma de Buenos Aires, Argentina ^3^CONICET, Ciencias Médicas, Ciudad Autónoma de Buenos Aires, Argentina ^4^Facultad de Odontología- Universidad de Buenos Aires UBA- Argentina., Cátedra de Anatomía Patológica, Ciudad Autónoma de Buenos Aires, Argentina

Boron Neutron Capture Therapy (BNCT) combines selective tumor uptake of 10B compounds and neutron irradiation. Oligo-Fucoidan, a seaweed extract, has anti-inflammatory and anticancer activities. Glutamine is an amino acid with many functions in the body. The aim was to evaluate the therapeutic efficacy and radiotoxic effects of (BPA+GB-10)-BNCT alone or combined with Oligo-Fucoidan or Glutamine. BDIX rats with flank tumors induced by syngeneic colon cancer cells were treated locally with (BPA+GB-10)-BNCT at the RA-3 Nuclear Reactor.

a-(BPA+GB-10)-BNCT: borophenylalanine (BPA) 31 mg 10B /kg + Decahydrodecaborate (GB-10) 34 mg 10B/kg, i.v.
b-(BPA+GB-10)-BNCT + Oligo-Fucoidan: (a) + Oligo-Fucoidan (200 mg/ml) once a week for 7 weeks, joint oral-topical administration.
c-(BPA+GB-10)-BNCT + Glutamine: (a) + Glutamine (40 mg/ml) once a week for 7 weeks, with wet compresses.
d-Sham: same manipulation, untreated.

The post/pre-BNCT ratio of tumor volume at 7 weeks post-treatment was significantly lower for all the groups treated with BNCT vs SHAM (p <0.05). Using the end-point “incidence of tumors that underwent a reduction to ≤50% of initial tumor volume” to further assess therapeutic response, results were 57% for (BPA+GB-10)-BNCT alone, 80% for (BPA+GB-10)-BNCT + Glutamine and 100% for (BPA+GB-10)-BNCT + Oligo-Fucoidan. The incidence of severe dermatitis at two weeks was 100% for (BPA+GB-10)-BNCT alone, while Oligo-Fucoidan reduced incidence to 80 % and Glutamine reduced incidence to 40%, the latter reduction being statistically significant vs. (BPA+GB-10)-BNCT alone (p<0.05). (BPA + GB-10)-BNCT is therapeutically effective. Oligo-Fucoidan and Glutamine used as adjuvants would improve therapeutic efficacy and reduce radiotoxicity. **Acknowledgement**: HI-Q Marine Biotech International LTD.

## O085 -

### Fragmentation contributions to dose and relative biological effectiveness (RBE) across common RBE models in carbon radiotherapy

#### Shannon Hartzell^1^, Fada Guan^1^, Paige Taylor^1^, Rebecca Howell^1^, Christine Peterson^2^, Stephen Kry^1^

##### ^1^The University of Texas MD Anderson Cancer Center, Radiation Physics, Houston, USA ^2^The University of Texas MD Anderson Cancer Center, Biostatistics, Houston, USA

Variable RBE in carbon radiotherapy may be calculated using several models, including the microdosimetric kinetic model (MKM), stochastic MKM (SMKM), and Local Effect Model I (LEM), which have not been thoroughly compared. This work compares how models handle carbon beam fragmentation, providing insight into where model differences arise. Monoenergetic and SOBP carbon beams incident on a water phantom were simulated with Geant4 Monte Carlo. From these, input parameters for each RBE model (microdosimetric spectra, double strand break yield, kinetic energy spectra, dose fragment contributions) were calculated for each contributing fragment of a carbon beam (H, He, Li, Be, B, and C). Spectra for each fragment were used to calculate linear (α) and quadratic (β) portions by RBE model, which were combined with reference α and β values and physical dose (1 Gy) to calculate RBE. Calculations found that secondary fragment contributions could exceed 20% of total physical dose (Figure 1). When calculated using identical beam parameters, RBE magnitude varied greatly across models and was typically lowest using MKM. When compared across fragments, RBE decreased with atomic number when Z<3 and increased when Z≥3 for RBE_MKM_ and RBE_SMKM_ (Table 1). RBE_LEM_ increased with Z, until dropping sharply at Z=6. Trends of RBE by fragment varied by LET region for microdosimetric models only. This study demonstrated that secondary fragments could contribute notably to physical dose, indicating that fragmentation is an important factor in treatment delivery. Similar trends were seen in RBE fluctuations by atomic number for microdosimetric models, which differed from those of RBE_LEM_.

## O086 -

### Incorporating target volume uncertainties in the optimization process using a novel worst-case approach

#### Gregory Buti^1^, Kevin Souris^1^, Ana Maria Barragan-Montero^1^, John Aldo Lee^1^, Edmond Sterpin^1^

##### ^1^UC Louvain, Institute of Experimental and Clinical Research, Brussels, Belgium

**Objective**: This study proposes a worst-case optimization method that combines geometric uncertainties with target volume uncertainties, in a statistically-consistent way.

**Methods**: Two steps are performed: (1) a k-means clustering algorithm partitions the error space of uncertainties in k clusters of equal probability (k=60). k optimization scenarios are then defined as the center of each cluster. This error space includes a dimension with tumor infiltration errors to take into account the target volume uncertainty. Infiltration errors are modeled by isotropically dilating the GTV, followed by a correction for anatomical barriers. (2) Minimax optimization is performed with the 90% best scenarios (re-evaluated at each iteration). Discarding the 10% worst scenarios ensures that 90% confidence interval in dosimetric space. A lung tumor patient, treated with the IMPT modality (60 Gy prescription), is used as test case. The proposed method is compared to conventional CTV-based worst-case optimization. Treatment plan robustness is evaluated with the Monte Carlo dose engine MCsquare. The MCsquare robustness test recomputes the dose for 250 scenarios, sampling the following errors: tumor infiltration errors, setup errors, range errors and breathing motion.

**Results**: Fig. 1 compares the dose-volume histogram (DVH) bands and reports dosimetric metrics for the target and OARs for each method. The proposed method (right) reduces exposure to the esophagus (-7.9 Gy of D2) whilst maintaining similar coverage of the target (D95 > 57 Gy in the worst case).

**Conclusion**: A worst-case optimization method is proposed that considers target volume uncertainties explicitly in the treatment planning process.

## O087 -

### Scripted spot removal in critical structures for PBS proton therapy planning

#### Samantha Hedrick^1^, Valerie Coffman^1^, Scott Petro^1^, Bart Morris^1^, Khai Lai^1^, Lauren Rigsby^1^, Michael Leyva^1^

##### ^1^Provision CARES Proton Therapy Center, Medical Physics, Knoxville, USA

It's well known that the biological impact of a proton beam is greater than 1.1 at the Bragg peak and, traditionally, beams were chosen to avoid placing distal layers on critical structures. With multiple-field optimization planning, high weighted spots aren't necessarily in the distal layers anymore. To potentially improve OAR sparing, our clinic has developed a process to remove spots from critical structures. Within our treatment planning system (TPS), we have a script that allows the user to choose one or more ROIs in which to delete spots. The planner will start an optimization, during which the TPS will place spots within a margin around the target. The planner will delete spots via scripting, then continue the optimization, which will redistribute spot weight without changing spot position. Our clinic is currently focusing on skin sparing with this technique, so the planner will delete spots in the 5mm skin rind around the patient. We are evaluating the effect on breast, chestwall, and H&N plans. Areas of concern are lateral neck and medial breast, likely due to tangential skimming and spots ending past the target on the skin. Compared to planning without the script, nominal target coverage is unchanged. Nominal skin sparing is often improved. On average, deleting spots takes 1-2 hours with current hardware. With this technique, we hope to reduce skin reactions and improve patients' quality of life during treatment. Physics is attending patient OTVs each week to evaluate the effect of this technique on skin reactions.

## O088 -

### A novel energy layer optimization framework for spot-scanning proton arc therapy

#### Wenbo Gu^1,2^, Dan Ruan^1^, Qihui Lyu^1^, Wei Zou^2^, Lei Dong^2^, Ke Sheng^1^

##### ^1^University of California—Los Angeles, Radiation Oncology, Los Angeles, USA ^2^University of Pennsylvania, Radiation Oncology, Philadelphia, USA

Spot-scanning proton arc therapy (SPAT) improves proton plan dosimetry and delivery efficiency. However, existing greedy and heuristic algorithms do not promise dosimetry or efficiency optimality. To improve the dosimetry, planning and delivery efficiency of SPAT, we develop an optimization framework with integrated energy layer selection. Energy Layer Optimization for SPAT(ELO-SPAT) includes dose fidelity, group-sparsity regularization, log barrier function, and energy-sequencing (ES) penalty. Group-sparsity and log barrier function allow one layer selected per control point. Since delivery efficiency is most affected by the energy-layer switching-time (ELST) for switching from low to high energies, ES regularization is devised to penalize energy switch-up more heavily than switch-down, creating an efficient yet flexible energy layer sequence. Four cases including frontal base-of-skull, chordoma, head-and-neck and lung were tested. We compared ELO-SPAT with IMPT and SPArc by Ding et al. For the two arc algorithms, both plans with and without energy-sequencing were created. ELO-SPAT reduced optimization runtime by 80-90% compared with SPArc. In ELO-SPAT plans, one energy layer per control point was selected. By ES regularization, the number of energy switch-up was reduced to under 20 from 40-60 without ES. Compared with sequenced SPArc, ELO-SPAT with ES reduced total ELST by 15-20%. ELO-SPAT and SPArc achieved better sparing than IMPT. Without ES, ELO-SPAT achieved further OAR improvement than SPArc. Adding ES regularization degraded plan quality, but ELO-SPAT still had comparable or better sparing than SPArc. In conclusion, the proposed computation-efficient SPAT algorithm can generate plans with further improved dosimetry and delivery efficiency.

## O089 -

### SDDRO: Simultaneous Dose and Dose Rate Optimization for FLASH proton therapy

#### Hao Gao^1^, Bowen Lin^2^, Yuting Lin^1^, Shujun Fu^2^, Katja Langen^1^, Tian Liu^1^, Jeffrey Bradley^1^

##### ^1^Emory University, Radiation Oncology, Atlanta, USA ^2^Shandong University, Mathematics, Jinan, China

FLASH-RT can potentially reduce normal tissue toxicity while preserving tumoricidal effectiveness to improve the therapeutic ratio. The key for FLASH is to irradiate tissues with an ultra-high dose rate (i.e., ≥40Gy/s), for which isochronous cyclotron based proton systems are currently the only commercially available devices capable of delivering such high dose rate for clinical FLASH-RT. However, currently available treatment plan optimization method only optimizes the dose and does not directly optimize the dose rate. The contribution of this work to proton FLASH-RT is the development of a novel treatment optimization method, i.e., simultaneous dose and dose rate optimization (SDDRO). That is, SDDRO also accounts for dose rate constraint and optimizes dose rate distribution. In terms of mathematical formulation, SDDRO is a constrained optimization problem with dose-volume constraint on dose distribution, minimum dose rate constraint on dose-averaged tissue-receiving dose rates, minimum monitor-unit constraint on spot weight, and maximum intensity constraint on beam intensity. In terms of optimization algorithm, SDDRO is solved by iterative convex relaxation and alternating direction method of multipliers. SDDRO was compared with intensity modulated proton therapy (IMPT) (dose optimization alone, and no dose rate optimization) for 3 lung SBRT patients. SDDRO substantially improved the dose rate distribution compared to IMPT with preserved dose distribution, e.g., increasing of the region-of-interest (ROI) volume (ROI=CTV_10mm: the ring sandwiched by 10mm outer and inner expansion of CTV boundary) receiving at least 40Gy/s from ∼30-50% to at least 98%, and the lung volume receiving at least 40Gy/s from ∼30-40% to ∼70-90%.

## O090 -

### Dose and dose rate quantification for liver FLASH treatment planning using proton PBS transmission beams

#### Shouyi Wei^1^, Haibo Lin^1^, Carla Hajj^2^, Robert Press^3^, Arpit Chhabra^3^, Isabelle Choi^3^, Shaakir Hasan^3^, Charles Simone II^3^, Minglei Kang^1^

##### ^1^New York Proton Center, Medical Physics, New York, USA ^2^Memorial Sloan Kettering Cancer Center, Radiation Oncology, New York, USA ^3^New York Proton Center, Radiation Oncology, New York, USA

**Introduction:** This work aims to study transmission proton pencil beam scanning (PBS) FLASH radiotherapy (RT) planning for standard-of-care liver cancer cases based on Varian ProBeam parameters in FLASH mode.

**Methods:** First, transmission plans of 4.5 Gy x 15 fractions for 5 hepatocellular carcinoma patients were optimized with an in-house tool, using 2, 3, 4 and 5 fields combined with the highest minimal MU/spot from 100, 150, 200, 250, 300, 350, 400 that achieve acceptable plan quality and meet organ-at-risk (OAR) dose constraints. Then, 3D average dose rate (ADR) distribution was calculated (Fig.1). Liver-CTV mean dose, major OAR dose constraints and 40 Gy/s volume coverage (V_40Gy/s_) were characterized to evaluate the planning quality.

**Results:** All plans achieve reasonably good uniformities with CTV D_max_ for 2, 3, 4 and 5 fields of 116.0±1.2%, 118.0±4.1%, 117.5±3.4%, and 117.5±4.5%, respectively. The liver-CTV mean dose for 2, 3, 4 and 5 fields are 17.0±12.0 Gy, 16.1±10.6 Gy, 17.7±10.4 Gy, and 17.1±10.2 Gy. The V_40Gy/s_ of liver-CTV for 2, 3, 4 and 5 fields are 90.3±2.8%, 80.2±5.0%, 64.6±9.8% and 56.7±8.8%, with around 60% increase between the 2- and 5-field plans. The V_40Gy/s_ of other OARs have large variations due to the selection of beam angles and the distance to target (Fig.2).

**Conclusion:** In conclusion, FLASH planning for hypofractionation with smaller fractional dose (4.5Gy/fraction) is challenging. Using fewer fields can allow higher minimal MU/spot, leading to higher ADR coverages in OARs while achieving similar plan quality compared to plans with more fields.

## O091 -

### FLASH proton therapy at TRIUMF: demonstration and plans

#### Camille Bélanger-Champagne^1^, Crystal Penner^1^, Ewart Blackmore^1^, Michael Trinczek^1^, Stan Yen^1^, Cornelia Hoehr^1^

##### ^1^TRIUMF, Proton therapy group, Vancouver, Canada

The TRIUMF proton-therapy centre treated over 200 ocular melanoma patients between 1995-2018 with a 70/74 MeV proton beam. It operates off a high current 520-MeV maximum energy research cyclotron which can theoretically deliver > 2,500 Gy/s, a much higher dose rate than typically available at clinical accelerators. The proton beam energies available make it possible to deliver these high dose rates while the Bragg peak is positioned in the tumor. The facility has enormous potential for systematic studies of the FLASH effect in proton therapy with in-vitro and preclinical models. Here we demonstrate that operating within the existing clinical facility safety limits, TRIUMF can deliver FLASH dose rate proton beams over a wide energy range, see Figure 1. The irradiation field is sufficiently large (> 1cm) to enable early preclinical work, see inset in Figure 1. Furthermore, the potential of fluorescent PMMA optical fibres for dosimetry up to FLASH dose rates was established, see Figure 2. Planned upgrades to the beamline, dosimetry and safety systems to enable an increase of an order of magnitude or more in beam current are discussed.

## O092 -

### Optimizing design and commissioning of a synchrotron proton beamline for FLASH preclinical experiments

#### Uwe Titt^1^, Ming Yang^1^, Xiaochun Wang^1^, Steven Lin^2^, Xiaorong Ronald Zhu^1^, Iga Kiminori^3^, Narayan Sahoo^1^, Albert Koong^2^, Xiaodong Zhang^1^, Radhe Mohan^1^

##### ^1^University of Texas- M. D. Anderson Cancer Center, Radiation Physics, Houston, USA ^2^University of Texas- M. D. Anderson Cancer Center, Radiation Oncology, Houston, USA ^3^University of Texas- M. D. Anderson Cancer Center, Proton Therapy Center, Houston, USA

The purpose of this work was to optimize beam shaping devices to produce small volumes (up to ∼8 cm^3^) of uniform dose in a spread-out Bragg peak to achieve ultrahigh dose rates appropriate for FLASH experiments. We modified the characteristics of our ocular beamline to spill the accelerated protons in times as short as 2 milliseconds. Monte Carlo simulations were employed to design custom beam modifiers. These included a scattering foil and a conical flattening filter to maximize the flux of protons into the region of interest, ridge filters, range compensators, and apertures. Shapes, sizes and positions of the last three components were optimized to provide various field sizes and SOBPs in preparation of experiments to be performed. The stability and reliability of the beamline was ascertained, and dose distributions computed with Monte Carlo simulations for each configuration have been validated experimentally. With the modified devices we produced circular field sizes of 10, 15 and 20 mm diameter and SOBP modulation widths of 10, 15 and 20 mm. System tests revealed excellent stability and flatness of lateral dose profiles at the center of the SOBP was within ± 3%. Assessment of systematic uncertainties, such as impact of misalignments and positioning uncertainties was performed using simulations and the results used to make appropriate adjustments. As a result of this project, the modified beamline is now capable of delivering proton flash beams for in vitro and in vivo experiments.

## O093 -

### The first investigation of spot-scanning proton arc (SPArc) delivery time and accuracy with different delivery tolerance window settings

#### Gang Liu^1,2^, Lewei Zhao^1^, Di Yan^1^, Guillaume Janssen^3^, Xiaoqiang Li^1^, Xuanfeng Ding^1^

##### ^1^Beaumont Health System, Radiation Oncology, Royal oak, USA ^2^Union Hospital Tongji Medical College Huazhong University of Science and Technology, Cancer Cenrter, Wuhan, China ^3^Ion Beam Applications SA- Belgium, Advanced Technology Group, Louvain-la-Neuve, Belgium

**Purpose**: To investigate the Spot-scanning proton arc(SPArc) treatment delivery time and accuracy with various delivery tolerance window settings.

**Methods and materials**: SPArc plans were generated for four representative disease sites (lung, brain, head neck, liver cancer) with an angle sampling frequency of 2.5 degrees. An in-house dynamic arc controller was used to simulate the arc treatment delivery with various tolerance windows(±0.25, ±0.5, ±1, and ±1.25 degrees). The controller generates machine mechanical and irradiation information (logfile_simulation_) with one degree of sampling frequency during the arc delivery simulation, such as gantry speed, acceleration and deceleration, spot position, and delivery sequence similar to the actual machine logfiles. The logfile_simulation_ was then imported to the treatment planning system (TPS) to reconstruct the delivered dose distribution and compared to the initial SPArc plan. A three-dimensional gamma index with criteria 3mm/3% was used to assess delivery accuracy quantitatively. The dynamic arc delivery time(T_arc_) and relative lost time(RLT) [RLT=(T_arc_–fix beam delivery time)/fix beam delivery time×100] were reported.

**Result**: The 3D gamma pass ratio was great than 97% for all the cases(Fig 1and2). T_arc_ increased with the decreasing of delivery tolerance windows length(Fig 1). The average delivery time and the RLT(%) were 1024±516s(235%±31%),527±262s(73%±13%), 375±185s(23%±7%), 322±149s(7%±1%), 311±141s(5%±2%) for tolerance windows as ±0.25, ±0.5, ±1, and ±1.25 degrees respectively.

**Conclusion**: This is the first investigation of SPArc delivery time and accuracy with different delivery tolerance window settings. The simulated result indicated that the SPArc plan with 2.5deg sampling frequency can be delivered efficiently and accurately with ±1 or ±1.25degrees tolerance window.

## O094 -

### Assessing the interplay effect based on the cyclotron accelerator proton therapy system machine-specific delivery sequence model

#### Lewei Zhao^1^, Gang Liu^1^, Weili Zheng^1^, Jiajian Shen^2^, Andrew Lee^3^, Di Yan^1^, Rohan Deraniyagala^1^, Craig Stevens^1^, Xiaoqiang Li^1^, Shikui Tang^3^, Xuanfeng Ding^1^

##### ^1^Beaumont Health System, Radiation Oncology, Royal Oak, USA ^2^Mayo Clinic Arizona, Radiation Onclogy, Phonix, USA ^3^Texas center for Proton Therapy, Radiation Oncology, Irving, USA

**Purpose:** We proposed an experimental approach to build a precise machine-specific model for standard, volumetric, layer repainting delivery based on a cyclotron accelerator system. Then, we assessed the interplay effect using a 4D mobile lung target phantom compared to a generic delivery sequence model from West German Proton Therapy Essen (WPE) with the same type of proton system.

**Methods:** Test fields and clinical treatment plans were used to drive each parameter that impacted beam delivery time, including spot scanning, energy layer switching. To quantitatively evaluate the interplay effect, a series of digital thoracic 4DCT image sets were used. The interplay effect was assessed based on the 4D dynamic dose accumulation method. Different delivery techniques such as standard delivery, volumetric (n=2,3,4) and layer repainting delivery (n=2,3,5,25) were simulated based on the machine-specific delivery sequence model and WPE model. D99 (Dose received by 99% of target volume) of the target is used to estimate the delivery accuracy.

**Results:** The results showed that the WPE model's spot delivery sequence deviated from the log file significantly compared to the machine-specific model (Figure 1). The WPE model leads to an enormous difference in delivery time prediction compared to the machine-specific model in layer repainting technique. Such a difference also resulted in different interplay effects estimation (Figure 2) between the two models even though both institutions used the same proton system and calculated using the same 4DCT imaging set.

**Conclusion:** A precise machine-specific delivery sequence is highly recommended to ensure an accurate estimation of mobile target treatment's interplay effect.

## O095 -

### State-of-the art fixed field permanent magnet proton therapy gantry

#### Dejan Trbojevic ^1^

##### ^1^Brookhaven National Laboratory, Collider Accelerator Depratment, Upton, USA

We present the top-notch design of the proton therapy gantry made of permanent magnets with very strong focusing. This represents a superb solution fulfilling all cancer treatment requirements for all energies without changing any parameters. The proton energy range is between 60-250 MeV. The beam arrives to the patient focused on each required treatment energy. The scanning system is place between the end of the gantry and the patient. There are multiple advantages of this design: easy operation, no significant electrical power - just for the correction system, low weight, low cost. The design is based on the recent very successful commissioning of the permanent magnet ERL 'CBETA' at Cornell University.

## O096 -

### High-gradient magnetically focused system for preclinical proton minibeam experiments

#### Anthony Teran^1^, Andrew Wroe^2^, Jerry Slater^1^, Grant Mcauley^1^

##### ^1^Loma Linda University, Radiation Medicine, Loma Linda, USA ^2^Miami Cancer Institute, Radiation Oncology, Miami, USA

Numerous studies have shown a favorable differential response between normal and tumor tissues to spatially fractionated proton and photon irradiation. However, the underlying radiobiology responsible for this response remains to be fully elucidated, thus necessitating diverse preclinical experiments. The purpose of the present project was to create a system to allow such experiments to be performed in an existing clinical proton facility. Planar beamlets were produced by focusing 127 MeV protons (range in water 10 cm) with high-gradient (250 T/m) quadrupole Halbach cylinders. Magnets with a 10 mm bore diameter and 68 mm length were mounted on a breadboard (fitted as an instant replacement for the treatment table) and placed inline with 10 mm diameter passively scattered collimated pencil beams in a clinical treatment room (Fig 1). Dose distributions were measured in a water tank mounted to a stage also attached to the breadboard. Spatially fractionated composite dose distributions consisting of 3 beamlets were created by shifting the stage (water tank) with respect to the delivered beamlet. Preliminary results show composite beams with high proximal spatial fractionation (PVDR_11mmWED=9.9, FWHM_11mmWED=1.9 mm (Fig2)) and homogeneous dose at target. However, Monte Carlo simulations suggest larger PVDR and smaller FWHM values are possible (eg, PVDR_10mmWED=35, FWHM_11mmWED=1.6 mm for 127 MeV beams) when magnets with larger lens powers are used and experiments are ongoing. The system described will deliver focused proton minibeams using single or multiple magnets for biology and physics experiments and can be mounted to a standard kVue treatment table.

## O097 -

### Optimizing the model-based selection of head and neck cancer patients for proton therapy using a delineation-based preselection tool

#### Makbule Tambas^1^, Hans Paul van der Laan^1^, Arjen van der Schaaf^1^, Johannes A. Langendijk^1^

##### ^1^University of Groningen- University Medical Center Groningen, Department of Radiation Oncology, Groningen, Netherlands

**Purpose:** In the Netherlands, patients with HNC are selected for proton therapy using model-based approach, requiring plan comparison (VMAT vs. IMPT) for each patient. Our aim was to develop a preselection tool to select patient for plan comparison.

**Methods:** 151 HNC patients included. Separate linear regression models for individual OARs were created to predict the VMAT and IMPT OARs Dmeans, where the predictors were OARs volume percentages overlapping with target volumes. Then, actual and predicted plan comparison outcomes were compared. The endpoint was a positive selection outcome, (being selected for proton). A post-hoc sensitivity analysis was performed on the first and the second half of the patients based on their treatment initiation date.

**Results:** 106 (70%) patients were selected for proton after plan comparison. Actual and predicted OAR Dmeans (VMAT R2=0.953, IMPT R2=0.975) and NTCP values (VMAT R2=0.986, IMPT R2=0.992) were highly correlated (Table 1). The sensitivity, specificity, positive and negative predictive values (PPV, NPV) of the new preselection tool were 46%, 93%, 94% and 42%, respectively. It was consistent between the first and second half of the cohort (first vs. second half, proton indication: 67% vs. 74%, sensitivity: 46% vs. 46%, specificity: 92% vs. 95%, PPV: 92% vs. 96%, NPV: 46% vs. 39%).

**Conclusion:** The model-based selection outcome can be predicted using only the delineation data. The probability of qualifying for proton is >90% when the tool indicates a positive outcome for protons. This tool will contribute significantly to a more effective selection of HNC patients for proton therapy.

## O098 -

### Seed spots analysis to characterize linear-energy-transfer (LET) effect in the adverse event regions of head and neck in intensity-modulated-proton-therapy (IMPT)

#### Yunze Yang^1^, Samir Patel^1^, Jidapa Bridhikitti^2^, William Wong^1^, Michele Halyard^1^, Lisa McGee^1^, Jean-Claude Rwigema^1^, Steven Schild^1^, Sujay Vora^1^, Tianming Liu^3^, Martin Bues^1^, Mirek Fatyga^1^, Robert Foote^2^, Wei Liu^1^

##### ^1^Mayo Clinic Arizona, Department of Radiation Oncology, Phoenix, USA ^2^Mayo Clinic Rochester, Department of Radiation Oncology, Rochester, USA ^3^University of Georgia, Department of Computer Science, Athens, USA

**Purpose**: We propose to investigate the effects of linear energy transfer (LET) upon adverse events (AEs) in intensity-modulated proton therapy (IMPT) using dose linear-energy-transfer volume histograms (DLVHs) and seed spots analysis.

**Methods**: We included 14 H&N patients with unanticipated CTCAEv4.0 grade≥3 AEs. The AE regions were contoured and the corresponding DLVHs of the AE regions were generated (**Fig.1a**). DLVH was constructed with physical dose (Gy) and LET (keV/μm) as independent variables. The normalized volume of the structure was contoured as iso-volume lines in the dose-LET plane. All voxels in the structure were mapped into the dose-LET plane as dots. For seed spot analysis, we selected voxels at the top edge of the DLVH plots as critical voxels. Individually clustered critical voxels that are geometrically apart were considered as independent seed spots (**Fig.1b**). Median dose/LET from seed spots were extracted (**Fig.2a**). Bivariate-linear-regression models were established. The dose-LET-product (xBD) volume constraint of osteoradionecrosis was obtained using the receiver operating characteristic curve of additional independent 4 osteoradionecrosis patients and 15 control patients.

**Results**: Dose played a dominant role for in-field AEs, while LET played an important role in out-of-field AEs. Intercept-free linear models between the reciprocal of dose and LET were established (**Fig.2b**). The xBD volume constraint of osteoradionecrosis was derived, *V*(xBD≥275.18 Gy·keV/μm)<0.0612cc, with an area under curve of 0.85 (**Fig.2c**).

**Conclusion**: A potentially important LET-enhancing effect to induce AEs in H&N cancer treated with IMPT was observed. Voxel-based DLVH and seed spots analysis are powerful tools for the AE study in IMPT.

## O099 -

### Carbon ion radiotherapy for locally advanced head and neck mucosal melanoma: clinical results in 40 consecutive patients treated at CNAO

#### Sara Ronchi^1^, Maria Bonora^1^, Rossana Ingargiola^1^, Eleonora Rossi^1^, Stefania Russo^1^, Alessandro Cicchetti^2^, Ester Orlandi^1^, Barbara Vischioni^1^

##### ^1^National Center for Oncological Hadrontherapy CNAO, Radiation Oncology Clinical Department, Pavia, Italy ^2^Fondazione IRCCS Istituto Nazionale dei Tumori di Milano, Prostate Cancer Program, Milan, Italy

**Aim**: To retrospectively analyze toxicity and outcome of carbon ion radiotherapy (CIRT) in locally advanced head and neck mucosal melanoma (LAHNMM) patients treated at CNAO.

**Materials and methods:** LAHNMM patients receiving curative CIRT from June 2013 to June 2020 were included. Toxicity was scored using CTCAE v.5.0. Local control (LC), overall survival (OS) and progression free survival (PFS) were calculated via Kaplan-Meier Method.

**Results:** Overall, 40 patients were included. Median age was 70 years(39-87). Tumor site was nasal-paranasal in 90%, tumor status naïve/recurrent in 77,5%/22,5%, stage T3/T4 in 17(42,5%)/23(57,5%). Mutational status was unknown in 12(30%), wild type in 23(57,5%), NRAS/BRAF/c-KIT-mutated in 3(7,5%)/1(2,5%)/1(2,5%) patients. 28(70%) patients were treated after surgery, 10(25%) with exclusive CIRT, 2(5%) received systemic therapy before and after CIRT. Mean GTV volume (in R2 and definitive cases) was 137 cc(5-276 cc). CIRT total dose was 65.6 or 68.8 Gy(RBE) in 22(55%) and 18(45%) patients, respectively. 17 patients (44%) received immunotherapy after CIRT. Median follow-up was 11 months. Acute toxicity at the end of CIRT was ≤G2/G3 in 95%/5%, with no toxicities >G2 at 3 months. Late toxicity was ≤G2 in 81%; 2 patients (5%) had respectively G3 unilateral hearing loss and G4 unilateral visual loss (expected toxicities). At last follow-up, LC was maintained in 33 patients (84,6%). 2 year-LC, OS and PFS were 73%, 62% and 27%, respectively.

**Conclusions:** CIRT in LAHNMM is promising and safe. Impact of mutational status and immunotherapy are still under investigation. Prospective trials and further multidisciplinary efforts are required to improve prognosis and survival.

## O100 -

### Proton beam therapy achieves favorable organ at risk sparing and minimal acute toxicity in patients with major salivary gland tumors

#### Michael Chuong^1^, Siddharth Chundru^1^, William Hartsell^2^, John Chang^3^, George Laramore^4^, Sanford Katz^5^, Henry Tsai^6^, Craig Stevens^7^, Carlos Vargas^8^

##### ^1^Miami Cancer Institute, Radiation Oncology, Miami, USA ^2^Northwestern Medicine, Radiation Oncology, Chicago, USA ^3^Oklahoma Proton Center, Radiation Oncology, Oklahoma City, USA ^4^University of Washington, Radiation Oncology, Seattle, USA ^5^Willis Knighton Cancer Center, Radiation Oncology, Shreveport, USA ^6^ProCure Proton Therapy Center, Radiation Oncology, Somerset, USA ^7^Beaumont Cancer Center, Radiation Oncology, Royal Oak, USA ^8^Mayo Clinic, Radiation Oncology, Scottsdale, USA

**Background**: Because many head and neck organs at risk (OARs) are adversely affected by even low dose, proton beam therapy (PBT) may significantly reduce toxicity over XRT although supporting clinical evidence is limited.

**Methods**: A retrospective analysis of prospectively recorded PBT outcomes was performed for patients with major salivary glands enrolled on a multi-institutional registry trial (NCT01255748). Our hypothesis was that PBT is associated with a significantly lower incidence of clinically meaningful (grade 2+) acute toxicity compared to historical XRT outcomes.

**Results**: One hundred twenty-five patients were analyzed either primary or metastatic cancer involving the parotid (N=105) or submandibular gland (N=20). Median age was 61 years. The most common histologies were mucoepidermoid (19.2%) and squamous cell carcinoma (15.2%). Most were T1/T2 (54.4%) and N0 (57%). PBT was typically given postoperatively (68%) with median prescription dose of 66 GyE in 33 fractions. The ipsilateral neck rarely treated (32%). Uniform scanning was the predominant delivery technique (54%). Concurrent chemotherapy was uncommon (20%). Median follow-up was 20.4 months (range 2.0-95.1). Acute grade 2+ nausea (2.4%), dysgeusia (4.8%), xerostomia (6.4%), mucositis (18.4%), and dysphagia (10.4%) were rare. Median OAR doses were low: (mean oral cavity: 0.78 GyE, mean contralateral parotid gland: 0 GyE, mean contralateral submandibular gland: 1.28 GyE, mean larynx: 11.8 GyE, maximum brainstem: 0.89 GyE).

**Conclusions**: PBT achieves a substantial reduction in acute toxicity for major salivary gland patients compared to historical XRT outcomes. These data support the use of PBT with curative intent for salivary gland patients in the absence of randomized data.

### O101 - Proton therapy outcomes for the treatment of head and neck melanomas: Prospective analysis from the Proton Collaborative Group

#### James Han^1^, Henry Tsai^2^, Nasiruddin Mohammed^3^, Carlos Vargas^4^, Lane Rosen^5^, John Chang^6^, Charles Simone^7^, Robert Press^7^

##### ^1^UCLA Health, Radiation Oncology, Los Angeles, USA ^2^ProCure Proton Therapy Center, Radiation Oncology, Somerset, USA ^3^Northwestern Medicine, Radiation Oncology, Chicago, USA ^4^Mayo Clinic, Radiation Oncology, Scottsdale, USA ^5^Willis-Knighton Cancer Center, Radiation Oncology, Shreveport, USA ^6^OKC Proton Center, Radiation Oncology, Oklahoma City, USA ^7^New York Proton Center, Radiation Oncology, New York, USA

**Purpose/Objectives:** This study reports on the efficacy and toxicities of proton beam therapy (PBT) for primary melanomas of the head and neck (HN) region.

**Materials/Methods:** We queried the prospectively collected, multi-institutional Proton Collaborative Group registry for all consecutive patients with HN melanoma receiving PBT from 5/2010-12/2019. Kaplan-Meier methods were used to estimate overall survival (OS), progression free survival (PFS), and locoregional recurrence free survival (LRFS). Toxicity was reported per CTCAE v4.0.

**Results:** Sixteen patients were identified. Median age was 70 years (range 37-88). Primary disease included mucosal melanoma (n=8) and cutaneous melanoma involving salivary glands and/or neck lymph node metastases (n=8). Most (n=14) were treated post-operatively. Four patients had T3 disease and 7 patients had T4 disease. In addition, 7 patients were also node positive. The median radiation dose was 58 CGE (27-70) and median dose per fraction was 2.5 CGE (1.8-10). At a median follow-up of 48 months, the 1- and 3-year OS rates were 93% and 73%, respectively. The PFS at 1 and 3 years were 67% and 38%, respectively. Median PFS was 25 months. LRFS was 92% at both 1 and 3 years. Nine patients developed distant metastases. Acute grade(G)2+ and G3+ toxicities occurred in 16/16 patients and 5/16 patients, respectively. G3 toxicities included mucositis/pain (n=2), radiation dermatitis (n=2), and immunotherapy-related rash (n=1). No G4+ toxicities were reported.

**Conclusions:** Single modality PBT for HN melanomas in the definitive setting provides effective and durable LC rates with tolerable acute toxicity. Distant failure remains the primary pattern of failure.

## O102 -

### Experimental determination of the modulation power for lung tissue in ion beams

#### Jan Michael Burg^1,2^, Veronika Flatten^1,2,3^, Uli Weber^4^, Hilke Vorwerk^2,3^, Klemens Zink^1,2,3^, Kilian-Simon Baumann^1,2,3^

##### ^1^University of Applied Sciences, Institute of Medical Physics and Radiation Protection, Giessen, Germany ^2^University Medical Center Giessen-Marburg, Department of Radiotherapy and Radiooncology, Marburg, Germany ^3^MIT, Marburg Ion-Beam Therapy Center, Marburg, Germany ^4^GSI Helmholtzzentrum für Schwerionenforschung, Biophysics Division, Darmstadt, Germany

For the treatment of lung tumors using particle therapy, modulating effects on the particle beam may occur due to the structure of the lung tissue. These effects are caused by the heterogeneous nature of lung tissue and cannot be fully accounted during treatment planning, as these microstructures are too small to be fully resolved in planning CTs. In recent publications, a material parameter called modulation power was introduced in order to describe the effect and investigate the influence of lung modulation on dose distributions during irradiation using this parameter. For various artificial lung surrogates, this parameter was measured and is in the range of 100 to 800 μm. However, measurements to determine the modulation power of real lung tissue have not been published so far. In this work, the modulation power of real lung tissue was measured using porcine lungs as a model for human lung tissue. For this purpose, ex-vivo porcine lungs were frozen in a ventilated state depth dose curves were measured in a particle beam. From the obtained depth dose profiles with and without lung sample in the beam, the modulation power can be determined. For this purpose, a Gaussian distribution was optimized so that, when convolved with the reference curve, it reproduces the modulated curve. The modulation power can be calculated from the parameters σ and μ of this Gaussian distribution. For a total of 15 positions in 5 different lung samples the modulation power was determined.

## O103 -

### Analytical method to optimize the shape of conformal energy filters for Bragg peak FLASH proton therapy

#### Sylvain Deffet^1^, Kevin Souris^1^, Rudi Labarbe^2^, Lucian Hotoiu^2^, Arnaud Pin^2^, Edmond Sterpin^1,3^

##### ^1^Université catholique de Louvain, Molecular Imaging- Radiotherapy & Oncology, Woluwe-Saint-Lambert, Belgium ^2^Ion Beam Applications S.A., Research and development, Louvain-la-Neuve, Belgium ^3^Katholieke Universiteit Leuven, Laboratory of Experimental Radiotherapy- Department of Oncology, Leuven, Belgium

In order to maximize the dose rate to achieve a FLASH effect in proton therapy, one approach could be to use mono-energetic pencil beams to avoid delays caused by switching between energy layers. To obtain Spread-Out-Bragg-Peak from such beams, we consider the use of a ridge shifter of complex geometry also named Conformal Energy Filter (CEF). It is typically composed of many peaks to degrade the initial energy into those required for dose conformity. With such a geometry, scattering has a major impact on the dose distribution and must be taken into account to optimize the CEF. To perform the optimization in acceptable computational time, we have developed an analytical model which relies on the use of successive convolutions for scattering and straggling and which takes into account the fact that the CEF is made of a unique material, to maximize computation efficiency. The optimization can then be carried out either with a prior on the shape of the peaks (square, hexagonal, ...) or directly on a voxelized geometry as shown in Fig. 1. For this optimization, a conventional IMPT treatment plan (multi-energy) is first determined and is then used to simulate the dose in a water tank (Fig. 2). The difference between the dose map in water and that obtained with the CEF is then iteratively minimized considering an initial fluence map estimated from the conventional treatment plan.

## O104 -

### FLASH effect enhancement with a single proton-beam pulse?

#### Sergey Akulinichev ^1,2^

##### ^1^Institute for nuclear research of RAS, Medical physics laboratory, Troitsk, Russian Federation ^2^Hospital of RAS, oncology, Troitsk, Russian Federation

The experiments on proton flash therapy have been conducted so far at mean dose rate _m_ <200Gy/s. Does the flash effect in proton therapy change at significantly higher _m_ ? Unlike clinical proton accelerators, our linear accelerator can deliver the therapeutic dose of 50 Gy to a tumor (up to 1 kg) in a single pulse lasting 100μs. In this case, _m_ can reach values of 10^6^Gy/s. We used three possible irradiation modes: the conventional mode, _m_ < 3Gy/s, the “ordinary” flash mode, _m_ ∼ 60Gy/s, and the new single-pulse flash (*splash*) mode, _m_ > 30000Gy/s. With 209MeV protons, we irradiated plates with tumor and normal cells in the SOBP region. Lines HCT116 and HT29 were used as tumor cells and human fibroblasts (ADSC) as normal cells. The flow cytofluorimetry served to determine the level of apoptosis and other cell characteristics. The statistics of the first experiment are small, but the preliminary results indicate that in the “ordinary” flash mode the flash effect is manifested for radiosensitive HCT116 cells (SF∼1.7), but not for HT29 cells. In the *splash* mode, the flash effect is observed for both types of tumor cells, with SF about 6 for HCT116. The preliminary conclusion is that the flash effect increases with the significant growth of _m_ and the *splash* mode of proton irradiation _m_ can reduce radiation damage to normal tissues.

## O105 -

### Interlaced proton minibeam radiotherapy with heterogeneous tumor dose

#### Matthias Sammer^1^, Stefanie Girst^1^, Günther Dollinger^1^

##### ^1^Universität der Bundeswehr München, Institut für Angewandte Physik und Messtechnik, Neubiberg, Germany

Proton minibeam radiotherapy is a spatial fractionation method that spares healthy tissue better than conventional homogeneous irradiation schemes. The tissue sparing effect increases the smaller the minibeams and the larger the center-to-center (ctc) distances. We calculate dose distributions for unidirectional and interlaced proton minibeams. Pencil and planar minibeams are interlaced from two (opposing) directions as well as planar beams from four orthogonal directions. A tumor located at 10–15 cm depth within a 25 cm thick water phantom is considered. An initial beam size of σ_0_=0.2 mm (standard deviation) is assumed in all cases. Tissue sparing potential is evaluated by comparing calculated clonogenic cell survival using the linear-quadratic model. Interlacing proton minibeams for a homogeneous tumor irradiation had only minor benefits for cell survival compared to unidirectional minibeam irradiation. To improve the tissue sparing of interlaced minibeams, heterogeneous irradiation of the tumor was considered. A minimum dose criterion at each location within the tumor is applied to keep tumor control at least as high as for homogeneous irradiation. This leads to increased mean doses but nevertheless improves the cell survival up to the tumor edges due to the larger ctc-distances. The results suggest the application of a heterogeneous tumor irradiation with large ctc-distances. Furthermore, the calculations show optimum enhancement for large dose fractions pointing to hypofractionated or even single dose fractions to be the best option for minibeam irradiation scenarios. Similar benefits would result for heavy ion minibeams with the advantage of smaller minibeams in deep tissue thus even increased tissue sparing potential.

## O106 -

### A fast dose calculation approach for proton beams in magnetic fields

#### Alisha Duetschler^1,2^, Carla Winterhalter^1^, Sairos Safai^1^, Damien C Weber^1,3,4^, Antony J Lomax^1,2^, Ye Zhang^1^

##### ^1^Paul Scherrer Institute, Center for Proton Therapy, Villigen PSI, Switzerland ^2^ETH Zurich, Department of Physics, Zurich, Switzerland ^3^University Hospital of Zurich, Department of Radiation Oncology, Zurich, Switzerland ^4^University of Bern, Department of Radiation Oncology, Bern, Switzerland

**Purpose:** Although Monte-Carlo (MC) methods are considered the gold standard to simulate proton dose distributions in magnetic fields, a comparably accurate but more time-efficient analytical dose calculation approach is advantageous for exploiting the potentials of onboard MR guided proton therapy (MRgPT). We developed a fast dose calculation approach for MRgPT based on a deforming-dose-grid algorithm.

**Methods:** Dose distributions of proton beams (70-230 MeV) under varied magnetic fields (0/0.6/1.5/3.0 T) were simulated by TOPAS-MC. The beam-center-lines were extracted and corresponding dose grid points with and without magnetic field established by stepping along these in equal water-equivalent-lengths (fig1). Lateral grid points were correlated afterwards. The displacements of corresponding dose grid points were then used to warp the 0 T beam to other magnetic field scenarios. This approach was validated for single beams and applied for a plan of a sphere phantom in water. The dose distributions were directly compared to corresponding ground truth (GT) MC results.

**Results:** The example beams in fig2, show comparable dose distributions (absolute point-to-point difference less than 1.2%) for single proton beams for all low energy scenarios independent of the magnetic field. Pronounced dose differences were only observed for the highest beam energy (228.9 MeV) under 3 T, with a 1%/1mm-gamma index of 96.24%. For the sphere plan, a gamma index between the GT MC and the deformed dose distribution of 96.07% was obtained.

**Conclusion:** The fast dose calculation approach can accurately simulate proton beam distributions under magnetic fields. The technique will be further extended to inhomogeneous patient geometries.

## O107 -

### Experimental benchmarking of a MC beam model for Carbon ions in magnetic fields

#### Fatima Padilla Cabal^1^, Brad Oborn^2^, Dietmar Georg^1^, Hermann Fuchs^1^

##### ^1^Medical University of Vienna and Christian Doppler Laboratory for Medical Radiation Research for Radiation Oncology, Department of Radiation Oncology, Vienna, Austria ^2^University of Wollongong and Illawarra Cancer Care Centre- Wollongong Hospital, Centre for Medical Radiation Physics, Wollongong, Australia

**Introduction**: Dose calculations and treatment planning (TP) for magnetic resonance guided particle therapy will require accurate models describing the impact of magnetic fields on clinical beams. Dedicated Monte Carlo (MC) beam models will support dose calculations and generate calibration datasets for TP.

**Material and Methods**: A GATE9.0/Geant4 MC beam model was experimentally validated using ^12^C ion beams at five different clinical energies (120 – 402.8 MeV/u) at the MedAustron ion therapy center. Simulations included the clinical nozzle and a dipole magnet (Danfysik, Taastrup, Sweden) (B=0-1T) positioned at the treatment isocenter. Homogenous and fringe fields maps were calculated using COMSOL. For benchmarking, in-depth integrated radial dose profiles (IRDPs) at 0 and 1T were measured in a water phantom (810×125×400mm^3^). For beam optics, measurements were conducted using a Lynx detector (IBA, Schwarzenbruck, Germany) after the IC at different magnetic fields strengths.

**Results**: Range differences lower than 0.7% were obtained between simulated and measured IRPDs for all energies and magnetic fields, see Fig.1. Simulated in-air lateral beam deflection were found to be within 5% with respect to experimental measurements, see Fig.2. At the isocenter, beam deflections are expected to agree within 0.5mm for all configurations.

**Conclusions**: A MC model, allowing to generate basic beam data for clinical ^12^C ion beams in magnetics fields up to 1T, was successfully validated against experimental measurements. Further benchmarking against more complex irradiation fields is foreseen.

## O108 -

### Building driven data for AI with open platform to collect experiences in PT

#### Faiza Bourhaleb^1^, Claudia Pardi^1^, Mattia Cretoni^1^, Ludovic DeMarzi^2^, alexandru Dasu^3^

##### ^1^I-See Computing Ltd, I-See Computing Ltd, Turin, Italy ^2^Institut Curie- Research center, Hospital- Orsay Proton Therapy center, Orsay, France ^3^Skandionkliniken, Skandion, upsala, Sweden

We propose a cloud app solution for sharing and publishing investigations and providing training tool in proton therapy. This is a project partially financed by EU project INSPIRE. we are participating with a tool for commissioning, structured to embed a shared applications running distributed calculation for Monte carlo simulations for different beam lines, where different members can run different simulations. Relative analysis on the same private cloud app are shown after few minutes, and then stored. The cloud application is allowing different research institutes to publish their results in dynamic and interactive way. Moreover, the relative results analyses can be done online and downloaded in standard formats. This kind of software applications are a prototype of what could be in the near future results of an open research where we would share not simply graphs but the full set of tools in the experiment with the community. from another side such platform provides data input for creating an expert AI system where we could use the experience to feed deep learning algorithms in the PT field.

## O109 -

### Shield design cost matters

#### Joern Meissner ^1^

##### ^1^Meissner Consulting GmbH, Radiation Safety, Neubiberg, Germany

Proton therapy facilities shall be designed for as low as reasonably achievable (ALARA) exposure of persons. This can easily be achieved by making more and more conservative assumptions. But what is “reasonable”? To overcome this it may be a good idea to define key performance indicators (KPI's). KPIs can then be used to balance concrete mix design against concrete cost, shielding material cost against foot print constraints, quantify FLASH treatment capacity vs construction cost. Also other parameters like time to first patient, prioritization of shielding calculation results can play a vital role in the facility's budget. Worst case assumptions vs realistic disease site mix assumptions can cause substantial wall thickness variations. This presentation will provide examples of decision trees and techniques to identify KPI's and provide for some KPI's examples how the “reasonable” balance can be achieved.

## O110 -

### Impact of new delivery techniques (PMAT, flash-therapy) in the commissioning of operational radiation protection in compact proton therapy centers (CPTC)

#### Gonzalo Garcia^1^, Alejandro Carabe-Fernandez^2^, Alejandro Bertolet-Reina^3^, Hector Rene Vega-Carrillo^4^, Lenin Estuardo Cevallos Robalino^5^, Karen Arlet Guzman Garcia^4^, Marisa Ogando^6^, Jose Maria Gomez-Ros^7^, Eduardo Gallego^1^

##### ^1^Universidad Politecnica de Madrid UPM, Industrial Engineers Faculty - Energy Engineering, Madrid, Spain ^2^Hampton University Proton Therapy Institute, Medical Physics, Hampton, USA ^3^Massachusetts General Hospital, Physics Research, Boston, USA ^4^Universidad Autonoma de Zacatecas UAZ, Unidad Academica de Estudios Nucleares, Zacatecas, Mexico ^5^Universidad Politecnica Salesiana UPS, Automatization and Electronic, Guayaquil, Ecuador ^6^Bioterra SL, Direction, Madrid, Spain ^7^Ciemat, Radiation Protection, Madrid, Spain

Proton therapy is in continuous ever evolving to improve its performance. Some prominent current trends involve cutting-edge delivery methods or building compact proton centers. New developments have direct impact in radiation protection of facilities. Compact centers have specific features to reduce their size while achieving more affordable facilities: usually have one single room (sometimes two) and small footprint, higher radiation density, or mix of professional exposed workers (clinical and technical staff). These characteristics make compact centers face significant challenges, from the point of view of operational radiation protection. This work is framed into the research project *Contributions to operational radiation protection and neutron dosimetry in compact proton therapy centers (CPTC)*, which is focused on assessing the impact of these innovations on the operational radiation protection and commissioning of the compact facilities. Thus, several tasks have been carried out over the last three years in fields as checking shielding, comparing ambient dose yielded by neutrons in several CPTC, analyzing activation in shielding with different concretes, characterizing wide range Rem-meters to measure neutron fields, studying new proton delivery techniques and their neutron fields yielded, or assessing personal dosemeters suitable for CPTC. The aim of the work is to present those different activities developed from 2018 until now, in designing the operational radiological protection of compact proton therapy centers (CPTC), collecting outcomes achieved in the fields aforementioned. The current and new proton delivery methods compared in the yielding of neutron fields were IMPT (experimental and simulation), PMAT (experimental and simulation) and flash-.therapy (just simulation).

## O111 -

### A shielding evaluation paradigm for conventionally shielded proton facility as used for FLASH Radiotherapy (RT)

#### Zhiyan Xiao^1^, Yongbin Zhang^1^, Joe speth^1^, Eunsin Lee^1^, Anthony Mascia^1^, Mike Lamba^2^

##### ^1^Cincinnati Children's Hospital/University of Cincinnati Medical Center, Proton Therapy Center, Cincinnati, USA ^2^University of Cincinnati- Medical Center, Radiation Oncology, Cincinnati, USA

**Purpose:** This study proposes a shielding evaluation paradigm for conventionally shielded proton rooms as used for FLASH.

**Methods and Results:** We evaluated the current conventional shielding as used for FLASH radiotherapy. FLASH plan irradiations are typically in a range of a few hundred milliseconds, which is out of WENDI-2 response time. The dose-rate (beam current) dependence of detector response shows WENDI-2 detector stabilizes with dose rate no more than 2Gy/s (Fig.1). This corresponds to a beam current of no more than 3nA. The predefined FLASH plan (7x20 cm) was then tuned to be delivered at 3nA and 244MeV (maximum energy for conventional beam currents) for shielding evaluation at locations indicated in Fig.2. The proton beam was fully stopped in 30×30×40cm solid water slabs at isocenter.The results shown in below Table 1.

**Conclusion:** The ambient dose rates (including both photon and neutron) scaled to FLASH beam currents outside of the treatment room yielded results greater than 2 mR/hr, with a maximum outside the treatment room door. Due to the very short duration of the FLASH fields and the limited number of fields that can be delivered per hour, the cumulative doses delivered are <0.01 mR in an hour assuming 25 flash beams delivered. These results indicate that these conventionally shielded treatment rooms result in acceptable occupational and public doses when the FLASH beams are stopped at isocenter in solid water with the beam delivered at 0° gantry.

**Acknowledgements:** This project was funded by Varian.

## O112 -

### Characterization of Varian's first ocular proton therapy beamline and an interinstitutional comparison

#### Emmanuelle Fleury^1^, Petra Trnková^2^, Kees Spruijt^1^, Joël Herault^3^, Franciska Lebbink^1^, Jens Heufelder^4^, Jan Hrbacek^5^, Tomasz Horwacik^6^, Tomasz Kajdrowicz^6^, Anais Gerard^3^, Petter Hofverberg^3^, Maria Mamalui^7^, Roelf Slopsema^8^, Jean-Philippe Pignol^9^, Mischa Hoogeman^1^

##### ^1^HollandPTC, Radiation Oncology, Delft, Netherlands ^2^Medizinische Universitat Wien, Radiation Oncology, Vienna, Austria ^3^Centre Antoine Lacassagne, Radiation Oncology, Nice, France ^4^Charité - Universitätsmedizin, Department of Ophthalmology, Berlin, Germany ^5^Paul Scherrer Institute, Radiation Oncology, Villigen, Switzerland ^6^Institute of Nuclear Physics Polish Academy of Sciences, Radiation Oncology, Kraków, Poland ^7^University of Florida, Radiation Oncology, Florida, USA ^8^Emory Proton Therapy Center, Radiation Oncology, Atlanta, USA ^9^Dalhousie University, Radiation Oncology, Halifax, Canada

**Purpose/Objective:** The aim is to characterize the Varian's first ocular proton therapy facility by reporting its physical and dosimetrical properties as part of a multicentric comparison, and to address a clinical interpretation of the technical differences by simulating uveal melanoma specific case scenarios.

**Material/Methods:** The Varian Medical Systems proton eyeline was recently installed in the Netherlands, and clinical activity started early 2020. For commissioning and characterization of Varian eyeline, measurements of distal, proximal and lateral regions of the dose distributions were performed and compared to five ocular proton facilities with different technologies and initial beam energies. To interpret a clinical impact of different eyeline characteristics, irradiation of tumor case scenarios with different beam properties were simulated and analyzed: Small, medium and large uveal melanomas, located either anteriorly, at equator or posteriorly within the eye.

**Results**: The initial nozzle energy of 75 MeV and energy spread of 1.10 MeV of Varian eyeline result in an average lateral penumbrae of 2 mm and distal fall-offs of 0.30 g/cm^2^. It is in agreement with currently existing centers. Among institutions, the largest difference in lateral and distal penumbrae are 0.80 mm and 0.25 g/cm^2^, respectively. The largest 20% difference in the proximal region occurs for a simulated small deep-seated tumor, resulting in a potential increase of ocular toxicities in the anterior segment of the eye.

**Conclusion:** The HollandPTC Varian eyeline has been compared to other eyelines. The clinical interpretation of the physical and dosimetrical parameters among institutions may result in differences in ocular radiation-related toxicities.

## O113 -

### Quantitative Study on Enhancement of Patient Throughputs to Particle Centers by Adding an Inexpensive Imaging Room

#### Robabeh Rahimi Darabad^1^, Kiet Huynh^1^, Jiajin Fan^1^, Peng Wang^1^

##### ^1^Inova Schar Cancer Institute, Medical Physics, Fairfax, USA

Particle therapy offers clinical advantages compared to conventional radiation therapy for different types of cancers and patients populations, however because of its higher cost, the use of particles is not yet fully justified nor been implemented to its full capacity. There is a demand to increase the treatment efficiency of particles and to lower the cost. The expensive part is the “beam on time” and we want to consume it wisely. Nonetheless, a big part of the “room use time” is in fact drawn to patient pre-treatment positioning, which is indeed critical for treatment final outcome, but does not have anything to do with the use of the beam. In this work, an algorithm is developed to simulate the patient treatment schedule with imaging room(s) being added to a single or a multiple-gantry center. Figure 1 shows the schematic of the procedure. The algorithm is flexible in terms of input parameters, e.g. disease sites vs positioning times, the number of gantries/particle sources. The efficiency improvement is shown to be better than 16% for our center, INOVA Schar Cancer Institute that is an IBA two-gantry proton system with a single cyclotron. It is also shown that a better than 28% efficiency is achieved for a typical single-gantry center with only one imaging room being added, see figure 2. Our algorithm is an open source, will be accessible to public in our website, and in its new version will include a scheduling optimizer, as the next step of this work.

## O114 -

### Mutation induction from particle beams: modelling through systematic data collection and MKM

#### Andrea Attili^1^, Emanuele Scifoni^2^, Francesco Tommasino^3^

##### ^1^INFN, Roma 3, Roma, Italy ^2^INFN, Trento Insitute for Fundamental Physics and Applications TIFPA, Trento, Italy ^3^University of Trento, Department of Physics, Povo- Trento, Italy

**Purpose:** Since the early years, particle therapy treatments have been associated with concerns for late toxicities, especially secondary cancer risk (SCR). Nowadays, this concern is related especially to patients for whom long-term survival is expected (e.g. breast cancer, paediatrics). We present a dedicated statistical and modelling analysis aiming at improving our understanding of the RBE for mutation induction (RBE_M_) for different particle species.

**Methods:** We built a new database collecting all available RBE data for mutation induction (i.e. HPRT mutation assay) from literature (101 entries, distributed among 4 cell lines and 14 particle species). The data were employed to perform statistical and modelling analysis. The latter was performed by applying the microdosimetric kinetic model (MKM) to describe the mutagenesis in analogy to the lethal lesion induction.

**Results:** When considering all data available, correlation analysis between RBE for survival (RBE_S_) and RBE_M_ reveals significant correlation between these two quantities (Figure 1, ρ=0.79, p<0.01). The correlation gets even stronger when looking at a subset of data based on e.g. cell line, particle species, maximum RBE. We also show that the MKM can be successfully employed to describe RBE_M_, obtaining at the same time comparably good agreement with the experimental data and realistic model parameters (Figure 2).

**Conclusions:** We showRBE_S_ and RBE_M_ are strongly related. Together with the successful application of the MKM, in analogy to the RBE_S_, RBE_M_ can be readily implemented into TPS evaluations. This might contribute to a more accurate estimation of secondary cancer risk in particle therapy treatments.

## O115 -

### Microdosimetry of alpha particles correlates with induction of direct damage to DNA

#### Alejandro Bertolet^1^, Jan Schuemann^1^, José Ramos Méndez^2^, Harald Paganetti^1^

##### ^1^Massachusetts General Hospital, Radiation Oncology, Boston, USA ^2^University of California San Francisco, Radiation Oncology, San Francisco- CA, USA

**Purpose:** To elucidate the relationship between microdosimetric quantities scored on spherical sites of liquid water and the yield of double strand breaks (DSB) produced on DNA structures by alpha particles.

**Methods:** Using the Monte Carlo tool TOPAS and its extension for cellular and subcellular applications TOPAS-nBio, we scored microdosimetric distributions of short-tracks segments of alpha particles with initial kinetic energies from 0.5 MeV to 15 MeV. Spheres with radius ranging from 50 nm to 750 nm were employed as microdosimetric sites. Sites were uniformly sampled in a water box large enough to include all secondary electrons. Independently, linear plasmids of DNA were placed inside a sphere of 250 nm radius irradiated by an isotropic source of alpha particles with the same kinetic energies. We registered DNA direct damage by quantifying the number of double strand breaks (DSBs) produced directly by each track.

**Results:** A relation between alpha particle kinetic energy and dose-mean lineal energy (yD) was obtained for each site size, compared to the analytical model from Bertolet et al. (Radiat Res 2020;194:403-10) fitted to data for the 250 nm-radius sphere, shown in Figure 1. The yield of DSBs per Gy per giga base pair (Gbp) for each particle energy was found to correlate linearly with the corresponding for the 250 nm-radius site, as shown in Figure 2.

**Conclusions**: Dose-averaged microdosimetric quantities, such as , can be effectively used to predict the yield of DSBs produced by a given alpha particle, with a correlation factor of 0.0063 DSB/Gy/Gbp/(keV/μm).

## O116 -

### From analytic to machine learning: predictive performance of NTCP models for radiation-induced esophagitis in NSCLC cancer patients receiving proton radiotherapy

#### Mei Chen^1,2^, Zeming Wang^2^, Sun Jian^3^, Jiang Shengpeng^2^, Li Wang^4^, Narayan Sahoo^2^, Quynh-Nhu Nguyen^3^, Zhongxing Liao^3^, Joe Y Chang^3^, Xiaorong Ronald Zhu^2^, Xiaodong Zhang^2^

##### ^1^Ruijin Hospital- Shanghai Jiao Tong University School of Medicine, Department of Radiation Oncology, Shanghai, China ^2^The University of Texas MD Anderson Cancer Center, Department of Radiation Physics, Houston, USA ^3^The University of Texas MD Anderson Cancer Center, Department of Radiation Oncology, Houston, USA ^4^The University of Texas MD Anderson Cancer Center, Department of Experimental Radiation Oncology, Houston, USA

**Purpose**: This study aimed to compare the predictive performance of different modeling methods in developing normal tissue complication probability (NTCP) models for predicting radiation-induced esophagitis (RE) in non–small cell lung cancer patients receiving proton radiotherapy.

**Methods and Materials**: Four modeling methods were used to build NTCP models: Lyman-Kutcher-Burman (LKB), multivariable logistic regression using two variable selection procedures-stepwise forward selection (Stepwise-MLR) and least absolute shrinkage and selection operator (LASSO-MLR), and support vector machines (SVM). Predictive performance was validated by a bootstrap approach for a fair evaluation of each modeling method. The overall performance, discriminative ability, and calibration were assessed using the Negelkerke R^2^, area under the receiver operator curve (AUC), and Hosmer–Lemeshow (HL) test, respectively.

**Results**: Of all the 328 patients receiving passive-scattering proton therapy, 136 patients experienced grade 2 or higher RE within 6 months from the start of treatment. The LASSO-MLR model showed better overall performance with a mean validated Negelkerke R^2^ value of 0.332. All models had an AUC value higher than 0.7, with the highest value for the LASSO-MLR model. The *p* values of the HL test were 0.24, 0.79, 0.92, 0.85 for the LKB, Stepwise-MLR, LASSO-MLR and SVM models, respectively. LKB model had the smallest optimism in the model variation and discriminative ability.

**Conclusion**: The prediction of complication rates could be improved by incorporating clinical and dosimetric parameters. Models with fewer parameters showed less model uncertainties. Advanced machine learning approach might have limited applicability in clinical settings with a relatively small amount of data.

## O117 -

### Evaluating normal tissue damage after hadrontherapy by chromosome aberration prediction

#### Alessia Embriaco^1^, Mario P. Carante^1^, Grazia Brazzorotto^2^, Alfredo Ferrari^3^, Andrea Mairani^4^, Ricardo L. Ramos^1^, Paola Sala^5^, Francesca Ballarini^2^

##### ^1^INFN sezione di Pavia, Dipartimento di Fisica, Pavia, Italy ^2^University of Pavia, Dipartimento di Fisica, Pavia, Italy ^3^University Hospital Heidelberg, University Hospital Heidelberg, Heidelberg, Germany ^4^HIT Heidelberg Ion-beam Therapy center, HIT Heidelberg Ion-beam Therapy center, Heidelberg, Germany ^5^INFN sezione di Milano, Dipartimento di Fisica, Milan, Italy

In cancer hadrontherapy, the Relative Biological Effectiveness (RBE) is generally calculated by considering cell survival as the endpoint of interest. Although this is a good estimator of the beam effectiveness in eliminating the tumour cells, late damage in healthy tissues, including secondary tumours, seems to be better correlated with chromosomal aberrations. In particular, dicentrics in peripheral blood lymphocytes are considered good biomarkers of normal tissue damage, since the blood circulating in all tissues is inevitably irradiated during the treatment. In this study, lymphocyte dicentrics were used as an ad hoc endpoint to evaluate (late) normal tissue damage by means of BIANCA (BIophysical ANalysis of Cell death and chromosome Aberrations), a biophysical model that simulates radiation-induced chromosome aberrations and cell death. The dicentric yields predicted by BIANCA were compared with experimental data available in literature on lymphocyte irradiation with different ions, as well as photons as a reference. This allowed producing a radiobiological database that predicts dicentric induction as a function of dose, particle type and LET. Afterwards, an interface with the FLUKA MC transport code allowed predicting dicentric RBE along a C-ion SOBP, and finally the RBE predictions for dicentrics were compared with those for cell survival calculated in a previous work. This study showed that BIANCA can predict RBE not only for ion effectiveness in tumor cell killing, but also for normal tissue damage. Furthermore, preliminary results suggest that considering only the RBE for cell survival might lead to an underestimation of normal tissue damage.

## O118 -

### Can we optimise proton PBS delivery to give sufficient oxygen depletion for FLASH?

#### Bethany Rothwell^1^, Matthew Lowe^1,2^, Norman Kirkby^1^, Michael Merchant^1^, Amy Chadwick^1^, Ranald Mackay^1,2^, Karen Kirkby^1^

##### ^1^University of Manchester, Division of Cancer Sciences, Manchester, United Kingdom ^2^The Christie NHS Foundation Trust, Christie Medical Physics and Engineering-, Manchester, United Kingdom

FLASH radiotherapy is a rapidly developing field which promises improved normal tissue protection compared to conventional irradiation and no compromise on tumour control. The transient hypoxic state induced by depletion of oxygen at high dose rates is currently the most well-accepted explanation. The potential to deliver protons at FLASH dose rates and reach deep seated tumours means proton beam therapy provides a promising modality for FLASH. A number of studies have investigated FLASH effects using uniform fields of dose, however there is a lack investigation into the spatial and temporal variation of dose from proton pencil beam scanning (PBS). We have extended our model of oxygen reaction and diffusion in tissue to incorporate parameters which model the delivery of proton spots at high dose rates and their impact on oxygen levels. The model acts as a tool to predict the magnitude of oxygen depletion, and change in radiosensitivity, during a typical PBS treatment for different regions in the treatment field. We can use the model to explore potential FLASH treatment plans and various delivery strategies to enhance oxygen depletion effects in an optimised way. Results so far suggest that reordering the delivery of spots in a plan may have a significant impact on the variation in radiosensitivity during the treatment (Fig 1). Further work will continue to investigate delivery strategies which could achieve more optimised FLASH sparing in various treatment plans, and work towards FLASH-augmented planning with the capability to improve patient outcome.

## O119 -

### Evaluation of cell survival and DNA-repair performance for HSG/NB1 cells using Geant4-DNA

#### Dousatsu Sakata^1^, Masao Suzuki^2^, Ryoichi Hirayama^3^, Yasushi Abe^1^, Wook-Geun Shin^4^, Oleg Belov^5^, Ioanna Kyriakou^6^, Sinji Sato^1^, Masayuki Muramatsu^1^, Dimitris Emfietzoglou^6^, Susanna Guatelli^7^, Sebastien Incerti^4^, Taku Inaniwa^1^

##### ^1^National Institute of Radiological Sciences, Department of Accelerator and Medical Physics, Chiba, Japan ^2^National Institute of Radiological Sciences, Department of Basic Medical Sciences for Radiation Damages, Chiba, Japan ^3^National Institute of Radiological Sciences, Department of Charged Particle Therapy Research, Chiba, Japan ^4^Univ. Bordeaux, Cnrs/Cenbg, Bordeaux, France ^5^Joint Institute for Nuclear Research, Laboratory of Radiation Biology, Dubna, Russian Federation ^6^University of Ioannina, Medical Physics Laboratory, Ioannina, Greece ^7^University of Wollongong, Centre for Medical Radiation Physics, Wollongong, Australia

One current challenge that still remains as an issue to be solved is predicting biological endpoints bridging a gap between simulated initial damage and radiobiological mechanism (such as characteristics DNA repair process of specific cell). The aim of the study is to provide a model to predict cell survival fraction and DNA repair performance after proton irradiation for HSG/NB1 cells with initial DNA damage as inputs of the model. To evaluate cell survival fraction and DNA repair speed, we performed biological experiments (colony assay and FAR assay) irradiated by 70 MeV protons at NIRS-cyclotron. As same as the previous work, initial DNA damage have been estimated using Geant4-DNA simulations (as shown Fig1). The absorbed dose and the initial energies of protons at the cell entrance are also evaluated by means of Monte Carlo simulations; Geant4 (left top/bottom panel of Fig2). Two-lesion kinetics model is performed to predict survival fraction induced by residual DNA damage. The model parameters of TLK model have been adequately optimized to predict cell survival fraction (right top panel of Fig2) and FAR (which represent residual DSBs in naive) as a function of time up to 24 hours(right bottom panel of Fig2).

## O120 -

### Feasibility of proton therapy Set Up in developing country like India: An initiation of new era in radiation treatment

#### Maitrik Mehta ^1^

##### ^1^The Gujarat Cancer & Research Institute, Radiotherapy Department, Ahmedabad, India

Radiotherapy treatment acts with the goal of balance between long term survival and less side effects, which is currently achieved with IMRT and Stereotactic treatments. Protons with its physical properties like almost no dose to normal structures beneath the target volume has shown significant interest in experimental research and clinics from last 20 years in Pediatric patients, Skull base tumors, Spinal tumors, Prostate, Breast etc. But, still most of the data are not of randomized controlled trials, to justify its use in routine clinical treatment. Proton therapy installation comes with huge cost and regarding set up in developing countries, if we see, number of patients diagnosed and treated are in quite huge number (almost one million cases every year), to be potential source of enrollment in clinical trials. In my State Cancer Centre in western part of India, we are treating almost 200-250 new pediatric patients every year with latest technologies and giving results almost in the range of 55-60%. In other Regional or State cancer centers across India, the scenario is almost same. If we consider cost as a factor, with the help of State Government and Central Government, Proton therapy Centre can judiciously be installed in Regional/State cancer Centre, and these centers can provide treatment at cheaper rates too. As in forthcoming years, cancer incidence will increase tremendously, so, to look at cost versus treatment results, cost will have minimal impact as everybody are connected digitally in current world and we can have guidance from masters all over the world.

## O121 -

### A structured programme template to create a sustainable workforce in a proton therapy centre in a developing country

#### Michael Penniment ^1^

##### ^1^RAH, Radiation Oncology, Adelaide, Australia

The Australian Bragg Centre for Proton Therapy (ABC) will commence treatment in late 2024. We aim to staff the centre with proton therapy (PT) trained health care professionals and providing opportunity to young Australians and world leading experts. Our initial strategy includes:

Phase 1: 2020-2021

- PT specific coursework in dosimetrist and physics undergraduate and postgraduate University courses .
- Train current Royal Adelaide Hospital Radiation Oncology staff, utilising comparative proton/photon planning
- Enhance our existing national clinical teleconference to discuss complex treatment plans

Phase 2: 2021->operations

- Extend our current work in artificial intelligence with the Australian Institute for Machine Learning. Auto-contouring is part of our current RT planning process and machine learning moves us from atlas based to continually refined personalised and adaptive patient care.
- Hold an online and attended training symposium with invited faculty both to consider models of remote learning and to develop a Bragg Centre Academic Faculty
- Embed our staff in positions overseas, working as clinicians and commissioning physicists in major particle therapy centres.

Phase3: 2023 onward: Complete operational team recruitment and training. This is also intended to assist training needs into the expanding Asian PT market.

This presentation will develop strategies for online, remote flexible training systems to meet the future professional staff requirements of our region and provide a template to assess countries developing PT facilities to build a sustainable local workforce. It is intended to foster specific discussions on how the Asian region builds a sustainable workforce for the next decade of treatment facility expansion.

## O122 -

### Molecularly aggressive ependymomas treated with Image guided pencil beam proton therapy: consecutive patient Indian experience

#### Rishan Thimma Sudarsan^1^, Srinivas Chilukuri^1^, Nagarjuna Burela^1^, Sham Sundar^1^, Utpal Gaikwad^1^, Rajesh Thiyagarajan^2^, Noufal Manthala Padannayil^2^, Adhithyan Rajendran^3^, Pankaj Kumar Panda^1^, Dayananda Sharma^2^, Roopesh Kumar^4^, Rakesh Jalali^1^

##### ^1^Apollo proton cancer centre, Radiation Oncology, Chennai, India ^2^Apollo proton cancer centre, Medical Physics, Chennai, India ^3^Apollo proton cancer centre, Radiology, Chennai, India ^4^Apollo proton cancer centre, Neurosurgery, Chennai, India

**Aim:** To assess the toxicities and early clinical outcomes in patients of primary and recurrent ependymomas treated with image guided pencil beam scanning proton beam therapy (PBS-PBT).

**Materials and Methods:** Between January 2019-2021, we analyzed consecutive patients of ependymomas treated with image guided PBS-PBT. They were also analyzed molecularly, and all recurrent/re-irradiated patients were considered for craniospinal irradiation (CSI). Acute toxicities were assessed based on NCI CTC v5.0, local control and radiological response by 3-monthly MRI post therapy.

**Results:** Fifteen consecutive patients with ependymoma (11 Grade III and 4 Grade II) (median age-9 years) were analyzed. 7 were treated at first recurrence and 1 at second recurrence. Majority had posterior fossa (PF) tumors (11) followed by supratentorial (ST) (2) and spine (2). Among PF ependymomas, 9 had global loss of H3K27me3, whereas amongst ST, 1 had YAP1 fusion and other, negative L1CAM and p65/RELA on immunohistochemistry. Gross/near-total resection was achieved in 87% patients. Among 8 patients treated for recurrence, 7 received CSI followed by primary site boost to a total median dose of 55CGE (50.2-55.8CGE). Grade 2 dermatitis, grade 2 and 3 hematological toxicities (CSI) were noted in 3,2 and 2 patients respectively. Grade 2 fatigue noted in 53% of all and 71% receiving CSI. With a median follow-up of 10 months (2-24 months), local control was 73% (upfront-100%, recurrent-50%).

**Conclusion:** Our experience of treating patients of ependymoma with complete resection followed by PBS-PBT including CSI is encouraging with low acute toxicities. Recurrent/molecularly aggressive cases may be considered for CSI.

## O123 -

### Cyst wall dynamics during and after image guided pencil beam scanning proton therapy in craniopharyngiomas

#### Nagarjuna Burela^1^, Srinivas Chilukuri^1^, Rajesh Thiyagarajan^2^, Adhithyan Rajendran^3^, Noufal Mp^2^, Dayananda S Sharma^2^, Rakesh Jalali^1^

##### ^1^Apollo Proton Cancer Centre, Radiation Oncology, Chennai, India ^2^Apollo Proton Cancer Centre, Department of Medical Physics, Chennai, India ^3^Apollo Proton Cancer Centre, Radiology, Chennai, India

**Purpose:** Craniopharyngioma cyst wall changes potentially perturb pencil beam scanning proton therapy (PBS-PBT) dose distributions. This study evaluates cyst wall dynamics and its dosimetric impact during the PBS-PBT.

**Material and Methods:** Eleven consecutive patients with craniopharyngioma treated with image guided PBS-PBT were analyzed. PBS-PBT plans were generated after target delineation to dose of 54CGyE/30fractions with robustness upto 3mm setup errors and +3.5% range uncertainty. Weekly quality assurance (QA)CT and/or MRI scans were performed to assess the cyst wall dynamics and PBT dose perturbations.

**Results:** Total of 34 QA scans were performed with median of 3 scans per patient. Four patients (36%) showed cyst deformation. Three patients showed an increase in the volume (median 0.52cc; range 0.15-1.16cc) and one showed a reduction in the volume (0.8cc). Largest changes to the cyst wall volumes were noted after 3 weeks in 2 patients and at 4 weeks in 2 patients. None required adaptive re-planning as no significant dose perturbation to GTV was noted. Median PTV dose perturbation was 0.32% (range 0.16 to 0.8%). Mean percentage change from the nominal plan for temporal lobes, hippocampus and optic chiasma were 1.2%, 1.3% and 0.5% respectively. With a median follow-up of 14 months(3-24 months), 5 patients showed shrinkage of residual tumor/cyst and rest showed stable disease.

**Conclusion:** We have demonstrated craniopharyngioma cyst wall deformations during PBS-PBT in more than one third of our patient cohort without significant dose perturbations to targets and OARs, reinforcing a need for stringent QA imaging during treatment of these patients.

## O124 -

### The HRH Princess Maha Chakri Sirindhorn Proton Center: Thailand's first proton center

#### Napapat Amornwichet^1^, Chonlakiet Khorprasert^1^

##### ^1^Faculty of Medicine- Chulalongkorn Universisty, HRH Princess Maha Chakri Sirindhorn Proton Center- King Chulalongkorn Memorial Hospital, Bangkok, Thailand

Thailand is a country of 70 million people. There are about 120,000 new cancer cases per year. Almost half of these patients need radiotherapy as a part of cancer treatment. Regarding ASTRO model policies, approximately 12,000 patients are indicated for PBT. With an in-patient capacity of 1,435 beds, King Chulalongkorn Memorial Hospital is one of Bangkok's largest university hospitals, operating under Chulalongkorn University and Thai Red Cross Society. Our center has been planning to use proton particles to treat patients since 2014, consisting of a feasibility study, technical, physical, cost-effectiveness, and personnel preparation. The Government budget of 1,200-million-baht was provided for one compact gantry, proton equipment, and new building. The construction began in 2017. This building is an underground building with a garden rooftop, depth of 15 meters, or equal to 3 floors located in the hospital center directly connected to the radiotherapy department. The full-featured ProBeam® Compact system is installed, including a cyclotron, a 360-degree gantry from Varian. This facility has received royal grace, granting the name of the Proton Center “the HRH Princess Maha Chakri Sirindhorn Proton Center” (HPSP). HRH Princess Maha Chakri Sirindhorn graciously presided over the cyclotron rigging ceremony on June 20, 2019. Due to the COVID situation, the planned first patient treatment is delaying to September 2021. For policy and reimbursement model establishment, data will be collected and conducted in systematic research. HPSP will be one of the prototypes in the compact proton therapy system integrated into the hospital.

## O125 -

### The importance of developing education and research programs in the emerging particle therapy landscape in Latin America

#### Marcelo Vazquez^1^, Gustavo Santa Cruz^2^

##### ^1^Loma Linda University Medical Center, Radiation Medicine, Loma Linda, USA ^2^CNEA, Gerente Investigación y Desarrollo en Aplicaciones Nucleares a la Salud, Buenos Aires, Argentina

The construction of the first proton center in Argentina, represent the dawn of particle Beam therapy in Latin America (LATAM) as well as the creation of new opportunities for education and research in radiation oncology and other disciplines. Education and research are of key importance for the future professional role and development of radiation oncologists in general and for particle therapy in LATAM. It is critical that radiation oncology departments must professionalize and expand their research laboratories and scientific staff to contribute efficiently to the growing demands of translational and clinical research, which are both indispensable for personalized cancer care and particle therapy. Research plays an essential role in informing evidence-based practice to ensure high-quality treatment and care is provided to patients. The Argentine Center of Proton Therapy (CEARP) and its Research and Development Proton Therapy Laboratory (LAIDEP) will provide a local and regional hub for the development and utilization of particle therapy in LATAM. The CEARP/LAIDEP will be the first center in Central and South Americas with advanced technologies in proton therapy, research, development, training, and technology transfer providing cutting edge research capabilities. Also, it will promote public and private initiatives to support research in particle therapy with the final goal of creating and expand a user community interested in the use of protons for scientific research and establish national and international partnerships to promote translational research, technology R&D and educational initiatives. The challenges and requirements to develop a local and regional research hub in LATAM will be discussed.

## O126 -

### 3D dose verification for carbon ion beams with an EBT3 Gafchromic film stack

#### Timo Steinsberger^1,2^, Michelle Lis^1,3^, Athena Paz^1^, Moritz Wolf^1^, Christian Graeff^1^

##### ^1^GSI Helmholtzzentrum für Schwerionenforschung GmbH, Biophysics, Darmstadt, Germany ^2^Technical University of Darmstadt, Institute of Condensed Matter Physics, Darmstadt, Germany ^3^Louisiana State University, Department of Physics and Astronomy, Baton Rouge, USA

**Purpose:** When treating moving tumors with scanned ion beams, the interplay effect can cause steep dose gradients inside the tumor volume. Therefore, high resolution absolute dosimetry is needed in order to quantify the performance of motion mitigation techniques. EBT3 Gafchromic films offer high resolution, but doses are distorted by the LET quenching effect. We present a method to compensate for this effect.

**Methods:** Carbon ion treatment plans were optimized for homogeneous absorbed dose distributions in a static and moving ellipsoidal target with and without a wedge in the beam path. They were delivered to a stack of EBT3 Gafchromic films and first converted to non-corrected dose distributions using calibration curves measured in the plateau region of the Bragg curve. Dose and dose averaged LET distributions were calculated using a dose reconstruction. A correction curve relating the LET quenching effect to the dose averaged LET was optimized for a static delivery with the wedge and then applied to the other treatments. Measured doses were compared to simulations using 3D gamma analysis.

**Results:** The correction was able to compensate the longitudinal (Figure 1) and lateral (Figure 2) dose fall-off in non-corrected films caused by the LET distribution. 3%/3mm gamma pass rates of (86.8±8.9)% for 16 films in the target volume and (94.3±4.7)% for 23 films in a volume with ≥5% target dose were achieved.

**Conclusions:** Film stacks are a viable tool for high resolution 3D dose verification and can improve the measurement of the treatment quality achieved with upcoming motion mitigation techniques.

## O127 -

### Full 2π field-of-view proton tracking and LET measurement with a compact Timepix-based detector

#### Racell Nabha^1,2^, Olivier Van Hoey^2^, Carlos Granja^3^, Alessio Parisi^2^, Marijke De Saint-Hubert^2^, Lara Struelens^2^, Filip Vanhavere^1,2^, Edmond Sterpin^1^

##### ^1^KU Leuven, Laboratory of Experimental Radiotherapy- Department of Oncology, Leuven, Belgium ^2^Belgian Nuclear Research Center SCK CEN, Research in Dosimetric Applications, Mol, Belgium ^3^ADVACAM, Space Radiation Research- Nuclear Physics, Prague, Czech Republic

High-sensitivity hybrid semiconductor pixel detectors can provide wide-range spectrometric and directional information of energetic charged particles. The high-granularity and small pixel size enable single-particle tracking with a high spatial resolution. The miniaturized radiation camera has proven to be particularly useful for characterizing primary and secondary radiation in particle therapy including measurements in-situ/inside phantoms. By analyzing the pixelated clusters created by single particles, a measurement of the deposited energy, linear energy transfer (LET) spectra and angular distribution can be obtained. However, the accuracy of LET calculation and directional detection using a single Timepix chip was generally limited to charged particles with an incidence angle higher than 20^0^. In this work, we present a new model to derive the proton's incident angle based on morphological cluster parameters. Using a single Timepix chip, we have extended the angular sensitivity down to 0^0^ (normal incidence) for 300 and 500 μm thick Silicon sensor detectors. This enables the reconstruction of the particle's incident angle with improved resolution over the full solid angle (2π). Consequently, the calculation of the track length across the sensor is extended and further improved. By using this method, the LET spectra of a wide-range of proton energies (10 to 200 MeV) were measured, and a very good agreement was found with Monte Carlo simulations. Our method shows that precise LET calculation and extended directional response with high angular resolution can be obtained even with a single layer Timepix detector. In future work, a similar approach will be applied to other particles.

## O128 -

### Response homogeneity of large area ionization chambers

#### Peter Kuess^1,2^, Ana Lourenço^3,4^, Severine Rossomme^5^, Viktoria Moser^1^, Dietmar Georg^1^, Hugo Palmans^2,3^

##### ^1^Medical University of Vienna, Radiation Oncology, Vienna, Austria ^2^MedAustron Iontherapy Center, Medical Physics, Wiener Neustadt, Austria ^3^National Physical Laboratory, Medical Radiation Science, Teddington TW 11 0LW, United Kingdom ^4^University College London, Department of Medical Physics and Biomedical Engineering, London, United Kingdom ^5^IBA Dosimetry, Medical Physics, Schwarzenbruck, Germany

The use of large area ionization chambers (LAICs) for scanned particle dosimetry requires detailed information on the uniformity of the response over the sensitive area. The method to determine such response was controversially discussed in recent literature [1–3]. In this study, narrow proton beams were applied to determine the lateral response variation of LAICs from different vendors. A collimated 80 MeV proton beam was used to map the response for four PTW-34080, four PTW-34070, and one PTW-34089. Furthermore, similar measurements were conducted with an IBA StingRay using an uncollimated proton beam (E=256.7 MeV). A collimated 200 kV x-ray beam was used as reference beam quality. Using Monte Carlo (MC) simulations (FLUKA), the secondary electron transport within the chambers was investigated for proton beams. All PTW-34070 and the StingRay showed a response decrease (2–5%) towards the chambers' edges. Three PTW-34080 showed an increase (up to 17%) and one chamber a decrease (8%). The response of the PTW-34089 increased by up to 5%. Response behavior of all chambers is summarized in Figure 1. The Fano test passed with an accuracy of 0.1%. The investigations showed that the response of LAICs is chamber dependent. Furthermore, the results indicate a similar response for protons and x-rays. The general heterogenous response of LAICs along the sensitive area needs to be carefully considered in particle dosimetry. Further MC studies will be performed to better understand the variations of the lateral response uniformity of LAICs.

## O129 -

### A Geant4 Fano test for high dose-per-pulse very high energy electrons

#### Michael Mcmanus^1^, Francesco Romano^2^, Gary Royle^3^, Hugo Palmans^1,4^, Anna Subiel^1^

##### ^1^National Physical Laboratory, Radiation Science, London, United Kingdom ^2^Istituto Nazionale di Fisica Nucleare, Sezione di Catania, Catania, Italy ^3^University College London, Medical Physics and Biomedical Engineering, London, United Kingdom ^4^MedAustron, Medical Physics, Wiener Neustadt, Austria

Ultra-short pulsed Very High Energy Electrons (VHEEs) provide multiple benefits over current clinical modalities including beam scanning, focusing for deep-seated tumours and increased dose conformity. VHEEs have also been previously investigated in regimes which are known to produce the FLASH biological effect. Dose-to-water determination using secondary standard ionization chamber measurements in high rose-rate VHEE beams relies on accurate correction of ion recombination and detailed Monte Carlo calculation of fluence perturbation factors and beam quality corrections. Confidence in the particle transport of Monte Carlo codes is necessary for the calculation of ionization chamber beam quality correction factors in high dose-rate VHEE beams but currently no studies exist. The Geant4 general purpose Monte Carlo code was chosen for this work as it allows for the simulation of possible hadronic interactions and neutron production at VHEE energies, unlike standard clinical simulation toolkits such as EGSnrc. However, Geant4 has never been previously used for these types of calculations. To demonstrate its performance, the Fano consistency test for the PTW-34001 Roos-Type plane-parallel ionization chamber has been conducted thoroughly using a Geant4 example code. Multiple particle transport configurations, including different ionization models, multiple-scattering models and boundary crossing algorithms have been tested and their efficacy determined. Geant4 has been found to pass the Fano test for a number of physics and particle transport configurations within 0.05% (± 0.06%). These configurations will be used to calculate perturbation and beam quality correction factors contributing to future development of dosimetry protocols and traceable dosimetry to a primary standard for VHEEs.

## O130 -

### Absolute and relative dosimetry for high dose-rate proton beams

#### Roberto Catalano^1^, Antonino Amato^1^, Giacomo Cuttone^1^, Giuliana Milluzzo^1^, Giada Petringa^1^, Giuseppe Antonio Pablo Cirrone^1^

##### ^1^Istituto Nazionale di Fisica Nucleare, Laboratori Nazionali del Sud, Catania, Italy

It is becoming evident, supported by many in-vivo and in-vitro experiments, that very high dose rate (>40 Gy/sec) ionizing radiation beams can strongly improve the efficiency in radiotherapeutic tumors treatment. Currently, however, no well-established absolute calibration protocols for the dosimetry of extremely high dose-rate charged particle beams exist, thus requiring each facility to establish its own dosimetric procedure. The challenges concerning the possibility to perform an accurate and reliable dosimetry of ultra-intense proton/ion beams, able to meet the specific requirements and tolerances of clinical applications, in particular, require the development of innovative detectors, methods and procedures. In this work, we will discuss the innovative dosimetric system for real-time absolute and relative dosimetry under FLASH irradiation conditions successfully developed and tested at the INFN-LNS (Catania, Italy) with pulsed proton beams up to 230 Gy/sec. The proposed approach is based on the use of dose-rate independent detectors (Faraday Cup and Radiochromic Films) coupled to a specifically designed dual-gaps in transmission ionization chamber, able to correct for any recombination effects, and a secondary electron emission monitor. The overall absolute dose estimation accuracy in terms of relative mean difference between the dose measured with the different detectors was found to be less than 5%.

## O131 -

### Dosimetry measurements for FLASH irradiation with 280 MeV/u carbon ion beams

#### Uli Weber^1^, Christoph Schuy^1^, Felix Horst^1^, Leon Baack^2^, Walter Tinganelli^1^, Yuri Simeonov^3^, Daria Boscolo^1^, Bernd Voss^1^, Marco Durante^1^, Thomas Haberer^4^, Stephan Brons^4^

##### ^1^GSI Helmholtzzentrum für Schwerionenforschung, Biopyhsics, Darmstadt, Germany ^2^Technical University of Darmstadt, Festkörperphysik, Darmstadt, Germany ^3^Technische Hochschule Mittelhessen University of Applied Sciences, LSE - Medizinische Physik, Darmstadt, Germany ^4^Heidelberger Ionenstrahl-Therapiezentrum HIT, Medical Physics, Heidelberg, Germany

In order to prepare radiobiological experiments with very high dose rates, we performed different dosimetry tests with a 280 MeV/u carbon beam at the Heidelberg Ion-Beam Therapy Center (HIT) and at GSI Darmstadt. Recent studies showed that a beneficial (FLASH) effect in the normal tissue is only expected at dose rates exceeding 40 Gy/s and doses larger than 8 Gy. Therefore, a particle rate of at least 5x10^9^ carbon ions/s is needed even for small tumour volumes.Especially for a scanned ion beam, this leads to an extremely high local ionization density in the detector gas for the beam monitors as well as for the dosimetry chambers. Thus, we found that air and ArCO_2_-filled beam monitors show strong saturation effects, resulting in an overdosage of 10% or more. Generally, ion beams from a synchrotron show strong beam intensity fluctuations, well known as ‘beam ripple'. Therefore, the aforementioned saturation effects cannot simply be compensated by a correction factor. Fluctuations of the beam intensity and the resulting fluctuations of the saturation level are unpredictable and would induce an inhomogeneous dose from beam scanning. Our approach is to install a dose monitoring system that shows only negligible saturation effects at FLASH beam intensities. In this work we will present the (voltage dependent) gas saturation effects of beam monitors and ionization chambers (see figure). Additionally, we tested a monitor and dosimetry system that minimizes these effects to an acceptable level <3%. We also present dosimetry results from FLASH SOBP irradiations (in-vivo + in-vitro) with carbon beams.

## O132 -

### Bridging the gap between the photons and particles by joining the carbon-ions and ultra-fractionated SBRT partial tumor irradiation

#### Slavisa Tubin^1^, Piero Fossati^1^, Antonio Carlino^2^, Markus Stock^2^, Eugen Hug^1^

##### ^1^EBG MedAustron GmbH, Medical department, Wiener Neustadt, Austria ^2^EBG MedAustron GmbH, Department of medical physics, Wiener Neustadt, Austria

In order to improve radiotherapy (RT) outcomes by exploiting immune-stimulatory radiation effects, an unconventional **SBRT**-based **PA**rtial **T**umor irradiation of **HY**poxic segment (SBRT-PATHY) was developed in 2016. Recently, relying on LET- and RBE-advantages of carbon-ions, a novel CARBON-PATHY concept has been clinically explored.

SBRT-PATHY has three main components determining its higher immunogenic potential compared to conventional RT: partial tumor irradiation targeting hypoxic cells, sparing of peritumoral immune microenvironment (PIM) and its time-synchronization with most reactive anti-tumor immune response phase. The analysis of 86 patients treated with SBRT-PATHY showed that adding to direct radiation tumor cell killing also the immune-stimulatory radiation effects in terms of bystander (BE) and abscopal effects (AE), has the potential to improve RT therapeutic ratio. Immunohistochemistry (IHC) and gene-expression analyses of surgically removed abscopal-tumor sites (ATS), suggested that the sparing of PIM at the time of an effective tumor-antigen release following the high-dose radiation of massive tumors is capable of inducing immunomodulatory effects of SBRT-PATHY. Although IHC showed very dense lymphocyte infiltration in radiation-spared PIM region, at ATS immune reaction was absent. However, an abundance of cell death related signaling molecules has been found not only in the partially irradiated tumors but even more so at ATS. We assessed the feasibility of CARBON-PATHY by treating 6 patients with highly complex and previously irradiated unresectable bulky tumors, which were unsuitable for further conventional RT or particle therapy. Translation of SBRT-PATHY to CARBON-PATHY was feasible and safe, showing promising potential in terms of local tumor control.

## O133 -

### Acute toxicity and patient reported outcomes after proton based stereotactic body radiotherapy

#### Naba Ali^1^, Bree Eaton^1^, Jun Zhou^1^, William Stokes^1^, Pretesh Patel^1^, Katja Langen^1^, Roelf Slopsema^1^, Jeffrey Bradley^1^, Mark McDonald^1^

##### ^1^Winship Cancer Institute of Emory University, Department of Radiation Oncology, Atlanta, USA

**Purpose:** To report acute toxicity and patient reported outcomes (PROs) following proton-based stereotactic body radiotherapy (SBRT) for palliation or ablation.

**Materials/Methods:** From Sep. 2019 to Dec. 2020, 52 patients were treated with proton SBRT to 62 lesions. Fractionation varied by indication and site with a median of 5 fractions and median fractional dose of 8 Gy. For immobile lesions, a target equivalent to a standard photon SBRT PTV was defined and optimized with range uncertainty (RU). For mobile targets, 4DCT was used to define an ITV for robust optimization, typically with 5 mm positional uncertainty + RU. Acute toxicities were prospectively recorded using CTCAE grading without attribution. PROs were assessed prior to and at completion of treatment using the MD Anderson Symptom Inventory (MDASI) and EQ-5D5L visual analogue score (VAS), where a 7-point change is considered clinically relevant.

**Results:** Twenty-one patients (40%) had no observable acute toxicity. Maximum recorded toxicities, related or unrelated to SBRT treatment, were grade 1 in 25 patients (48%), grade 2 (fatigue, depression, anorexia, proctitis) in 5 patients (10%), and grade 3 (dysphagia and hoarseness) in 1 patient (2%). Comparing pretreatment to end of treatment timepoints, there was a significant improvement in the mean VAS (68 to 76, p = 0.027, n=33), with no significant change in the mean MDASI symptom (1.8, 1.8) or interference (2.2, 2.3) scores.

**Conclusions:** Proton-based SBRT was well-tolerated with no decrement in patient reported outcomes and a mean 8-point improvement in VAS at the conclusion of SBRT. Further follow-up is necessary for tumor control and late effects analysis.

## O134 -

### Sacral nerve roots and ischial nerve as organs at risk in definitive carbon ion irradiation of sacral sarcoma

#### Piero Fossati^1^, Petra Georg^1^, Joana Gora^2^, Giovanna Martino^2^, Markus Stock^2^, Carola Luetgendorf-Caucig^1^, Eugen Hug^1^

##### ^1^MedAustron Ion Therapy Center-, Radiation Oncology, Wiener Neustadt, Austria ^2^MedAustron Ion Therapy Center-, Medical Physics, Wiener Neustadt, Austria

Radical surgery of pelvic sarcoma is technically challenging and can be mutilating. Carbon ion radiotherapy has been successfully employed in the definitive treatment of non-operated sacral sarcoma. Peripheral nerves neuropathy is a late unwanted side effect of high dose radiotherapy whose real incidence and pathophysiology are not yet completely understood. In proton and carbon ion series peripheral nerves toxicity is reported when dose to the cauda equine/nerves exceeds 70 Gy RBE. We defined an internal protocol for peripheral nerve sparing in high dose carbon ion radiotherapy of pelvic sarcoma. Individual nerve roots are contoured on axial CT slices. Ischial nerve is contoured dorsal to piriform muscle and ventral to gluteus maximus. Coronal reconstructions are employed to interpolate the contours in dubious slices. Prescription doses used for definitive carbon ion irradiation of pelvic sarcoma at MedAustron range from 76.8 Gy RBE in 16 fractions of 4.6 Gy RBE to 73.6 Gy RBE in 16 fractions of 4.8 Gy RBE. Dose constraints for peripheral nerves are set at 70 Gy RBE over 5 cm length and at 74 Gy RBE in a 0.01cc volume. Thanks to the favorable physical properties of carbon ions (small spot size and sharp lateral penumbra) it is possible to selectively spare peripheral nerve and maintain adequate target coverage. We have implemented a nerve sparing high dose carbon ion radiotherapy concept for pelvic sarcoma. Long term follow up is needed to confirm the efficacy of this approach in reducing clinically relevant radiation induced neuropathy.

## O135 -

### Safety of hypofractionated proton therapy: comparison of acute toxicity for normo- and hypofractionated irradiation

#### Maciej Pelak^1^, Petra Georg^1^, Piero Fossati^1^, Carola Lütgendorf-Caucig^1^, Birgit Flechl^1^, Konstantinovic Rastko^1^, Slavisa Tubin^1^, Galalae Razvan^1^, Mock Ulrike^1^, Joanna Gora^1^, Eugen Hug^1^

##### ^1^MedAustron, Ion Therapy Center, Wiener Neustadt, Austria

**Background and aim**: Data on the safety of hypofractionated proton therapy (PT) is limited. This study compares the early toxicity of normo-and hypofractionated PT using pencil-beam scanning technology.

**Material and methods**: We prospectively assessed acute toxicities in 90 patients treated within our registry study with PT for following locations: head and neck (n = 69), abdomen and pelvis (n = 18) and other soft tissue tumors (n = 3). Twenty-nine patients (32.2%) underwent PT following prior radiation treatment. Toxicities were scored according to CTCAE v 4.0 criteria. All patients were evaluated weekly during treatment and 3 months after PT completion. Comparisons between normo- (n = 73, dose per fraction [D/Fx]: 1.8-2.3, total dose [TD]: 60-79.2 Gy RBE) and hypofractionated (n = 17, D/Fx: 2.6-3.1, TD: 51-69 Gy RBE) PT were performed for each location and toxicity group.

**Results**: The median prescription EQD2 dose (a/b of 10 used) did not vary between normo- and hypofractionated treatments except the locations in pelvis (76 vs 65 Gy RBE, p = 0.005) and the percentage of re-irradiations was greater in the hypofractionated treatments (79% vs 19.7% p < 0.001). Despite these variabilities, there were no statistically significant differences between acute toxicity patterns in any of the analyzed locations except the skin toxicities in H&N location being less prominent in the hypofractionated group (Table 1). The delayed, prolonged and non-resolving events were statistically similarly frequent (Table 2).

**Conclusion**: Hypofractionated proton therapy appears to offer non-inferior early safety as compared to normofractionated irradiation.

## O136 -

### Toxicity outcomes of hypofractionated image guided pencil beam scanning proton beam therapy for spinal chordomas

#### Nagarjuna Burela^1^, Srinivas Chilukuri^1^, Kartikeswar Patro^2^, Sapna Nangia^1^, Adhithyan Rajendran^3^, Utpal Gaikwad^1^, Sham Sundar^1^, Rishan Thimma^1^, Dayananda S Sharma^2^, Pankaj Kumar Panda^4^, Rakesh Jalali^1^

##### ^1^Apollo Proton Cancer Centre, Radiation Oncology, Chennai, India ^2^Apollo Proton Cancer Centre, Medical Physics, Chennai, India ^3^Apollo Proton Cancer Centre, Radiology, Chennai, India ^4^Apollo Proton Cancer Centre, Clinical Research Unit, Chennai, India

**Objective:** To evaluate acute and late toxicities in patients of chordoma treated with hypofractionated, image guided pencil beam scanning proton beam therapy(PBS-PBT).

**Material and Methods:** Consecutive 23 patients diagnosed with spinal chordomas treated with PBS-PBT were included in this study. Among all patients, high risk CTV(CTV-HR) received dose of 70.4CGE/32fractions and intermediate risk CTV(CTV-IR) to 57.6-64CGE/32fractions. Patients underwent clinical and radiological(including metabolic- DCE MRI imaging) evaluations at baseline and 6 monthly thereafter. Acute and late toxicities were recorded as per CTCAE-V4.0 and LENT SOMA scales respectively.

**Results:** 17% patients(2clival and 2sacral) underwent prior radiation with photons (IMRT technique) with 13% receiving more than once. Mean volumes of CTV-HR for skull base chordomas was 32.4cc(0.83-132cc) and for sacral chordomas was 855cc(96.1-1740cc). Two patients had acute grade-3 dermatitis, one acute grade-3 oropharyngeal mucositis and one acute grade-3 gastrointestinal mucositis. 28.5% of patients with Clival and 50% of patients with Sacral lesions had grade-2 acute toxicities. 1 patient had late grade-3 toxicity requiring tracheostomy. None had any late grade2/3 gastrointestinal or genitourinary toxicities. With a median follow up of 17 months(9-23), 21 patients(91%) remained radiologically stable with favorable metabolic response in all the 10 patients (43%) where metabolic imaging was available. Only 1 patient had in-field radiological progression and one had distant bone metastases.

**Conclusion:** Pencil beam scanning proton beam therapy is feasible in patients with spinal chordomas. Despite high doses, large treatment volumes and large number of patients receiving prior treatments, the acute and late toxicities were relatively low in this cohort.

## O137 -

### Preclinical proton minibeam radiotherapy: Biomedical aspects

#### Judith Reindl^1^, Gerd Datzmann^2^, Stefanie Girst^1^, Matthias Sammer^1^, Günther Dollinger^1^

##### ^1^Universität der Bundeswehr München, Institute for applied physics and measurement technology, München, Germany ^2^Datzmann Interact & Innovate, Proton Therapy, München, Germany

The concept of spatial fractionation in radiotherapy was developed for better sparing of normal tissue in the entrance channel of radiation. Spatial fractionation utilizing proton minibeam radiotherapy (pMBRT) promises to be advantageous compared to X-ray minibeams due to higher dose conformity at the tumor. Preclinical in-vivo experiments conducted with pMBRT in mouse ear models or in rat brains support the prospects. However, the research on radiobiological mechanisms and the search for adequate application parameters delivering the most beneficial minibeam therapy is still in its infancy. Progressing towards clinical usage, pMBRT research should overcome technical and biomedical limitations of the current irradiation test stages and animal models. This work discusses results achieved so far in human skin tissue, in-vivo mouse ear and rat brain models. It further gives insight in the next steps and provides suggestions for biomedical research , which in our opinion has to be conducted for bringing pMBRT into clinical use. We consider glioma, non-small cell lung cancer and hepatocellular carcinoma as the most promising targets for preclinical and later clinical use. We furthermore propose an in-depth investigation of proton minibeams on healthy tissue. Especially neuronal cells and abdominal organs – both in in-vitro studies using artificial organoids as well as in in-vivo animal studies – need further testing. Taking together this gives a possible roadmap for bringing pMBRT into clinic.

## O138 -

### Effect of heavy ion beam on metastatic potential and survival of intermittently hypoxic cultured cancer cells

#### Yoshitaka Matsumoto^1^, Huizu Keiko Li^2^, Ryoichi Hirayama^2^, Koichi Ando^3^, Yoshiya Furusawa^4^, Hideyuki Sakurai^1^

##### ^1^University of Tsukuba, Faculty of Medicine Proton Medical Research Center, Tsukuba, Japan ^2^National Institute of Radiological Sciences- National Institutes for Quantum and Radiological Science and Technology, Department of Charged Particle Therapy Research, Chiba, Japan ^3^Gunma University, Heavy Ion Medical Center, Mebashi, Japan ^4^National Institute of Radiological Sciences- National Institutes for Quantum and Radiological Science and Technology, Department of Basic Medical Sciences for Radiation Damages, Chiba, Japan

The tumor microenvironment influences the behavior and prognosis of malignant tumors. The irregular vasculature of solid tumors creates regions of chronic hypoxia and regions of transient hypoxia, which are characterized by intermittent periods of hypoxia and reoxygenation. The effect of irradiation to the tumor cells experiencing intermittent hypoxia is not clear. The aim of study is to clarify the effect of irradiation to the cells selected by the intermittent hypoxic treatment in respect of cell survival and metastatic potentials of tumor cells. Cells were cultured in 0.1% O2 condition for 24 h and then cells were reoxygenated by normal culture medium for 2 days (1 cycle). The cells were cultured in this cycle maximum 15 cycles. Cells were irradiated with X-rays or carbon beams (C-ions) at the center of SOBP. Cells cultured under the intermittent-hypoxia showed low mitochondrial mass and membrane potential, and high discharge amount of lactic acid compared with oxic group. Additionally, the intermittent-hypoxia cells showed the resistance to apoptosis induced by hypoxic and irradiation, and these cells were more resistant to irradiation than oxic cells. The resistance ratio of X-rays is higher than C-ions. Further clarified were that these cells showed high metastatic potentials, migration and invasion abilities compared with oxic condition, and that C-ions were also effective to suppress the high metastatic potentials of these cells. It is suggested that intermittent hypoxia enhances the dependence on the anaerobic glycolytic system of tumor cells, and the enhancement contributes the radioresistance and metastatic potential.

## O139 -

### Brain organoids: A new tool to characterize mechanisms and biological differences of particle radiation-induced neurotoxicity

#### Foluwasomi Oyefeso^1^, Timothy Simon^2^, Marcelo Vazquez^3^, Antonella Bertucci^3^, Michael Pecaut^1^

##### ^1^Loma Linda University, Biomedical Engineering Sciences, Loma Linda, USA ^2^Loma Linda University, Basic Sciences, Loma Linda, USA ^3^Loma Linda University, Radiation Medicine, Loma Linda, USA

Particle therapy (PT) is becoming more widely available for clinical use. For patients with favorable prognoses, particularly childhood brain tumors, proton therapy is increasingly used for treatment. There is also interest in using carbon ion therapy for the treatment of aggressive brain tumors. However, the biological effects of PT (proton and carbon ions), may differ substantially from those of photon radiation. Therefore, there is a need to study the potential mechanisms of particle radiation (PR) neurotoxicity. Three-dimensional (3D) brain organoid cell culture systems have emerged as novel models to simulate the organization and cell diversity of human CNS in vitro. Here, we report the use of human brain organoids to study the cell-specific effects of PR on normal neural tissue as these models recapitulate features of the human brain, such as structural and functional integrity. In this study, we used brain organoids to characterize proton-induced changes to neural cells in vitro. Organoids were exposed to 0, 0.5 and 2 Gy of 250 MeV protons to evaluate changes in cell architecture (i.e., MAP2, GFAP), proliferation (Ki67), apoptosis (Caspase-3) and DNA damage (γ-H2AX) using immunofluorescence and confocal microscopy. Preliminary results from this study support the use of brain organoids as a translational tool to investigate particle-induced cellular and molecular changes as well as identify potential biological differences between photon and particle toxicity in the CNS.

## O140 -

### Cellular redox capacity as potential predictor for effectiveness of carbon ion radiotherapy

#### Ankita Nachankar^1^, Takahiro Oike^2^, Hiroshi Hanaoka^3^, Hiro Sato^2^, Yukari Yoshida^4^, Akihisa Takahashi^4^, Tatsuya Ohno^2^

##### ^1^Gunma University Graduate School of Medicine, Department of Radiation Oncology, Maebashi City, Japan ^2^Gunma University Graduate School of Medicine, Department of Radiation Oncology and Gunma University Heavy Ion Medical Center, Maebashi City, Japan ^3^Gunma University Graduate School of Medicine, Department of Radiotheranostics, Maebashi City, Japan ^4^Gunma University Graduate School of Medicine, Gunma University Heavy Ion Medical Center, Maebashi City, Japan

**Introduction:** Carbon ion radiotherapy (CIRT) offers superior biological and physical properties and better clinical results compared to X-rays. Few CIRT institutes worldwide highlight the emergent need to establish predictive biomarkers to stratify tumors most beneficial to CIRT over X-rays. To address this, we focused on tumor redox potential and investigated ^64^Cu-ATSM (Copper(II)- diacetylbis(4-methylthiosemicarbazonato), a redox imaging molecule, as potential predictive biomarker of relative biological effectiveness (RBE) of carbon ions over X-rays.

**Methods:** Ten human cancer cell lines were used. Sensitivities to X-rays (MX-160Labo, 160 kV, 3 mA) and carbon-ions (Gunma University Heavy Ion Medical Center, 290 MeV/nucleon, center of 6 cm-SOBP, average LET 50 KeV/μm) were assessed by clonogenic assays. *In vitro* ^64^Cu-ATSM uptake were assessed by radioactivity assay using gamma-counter. Cellular redox state was assessed by western blotting and by flow cytometry.

**Results:** *In vitro* ^64^Cu-ATSM uptake correlated positively with RBE. ^64^Cu-ATSM uptake-high cell lines showed greater upregulation of cellular antioxidant system in response to X-ray irradiation compared with ^64^Cu-ATSM uptake-low cell lines, indicating that these cell lines have high reactive oxygen species (ROS) scavenging capacity. Consistently, RBE of these cell lines showed strong positive correlation with their ROS scavenging capacity. Furthermore, inhibition of the ROS scavenging pathways by Brusatol (Nrf2 inhibitor) led to greater sensitization in X-rays than in carbon-ions, as well as for RBE-high cell lines than RBE-low counterparts.

**Conclusions:** Carbon ion RBE is associated with high ROS scavenging capacity of cancer cells, which can be targeted by ^64^Cu-ATSM imaging. *In vivo* validation is ongoing.

## O141 -

### Identification of an E6 inhibitor that sensitizes HPV-positive HNSCC cells to radiation

#### Lennox Chitsike^1^, Marcelo Vazquez^2^, Antonella Bertucci^2^, Penelope Duerksen-Hughes^1^

##### ^1^Loma Linda University, Basic Sciences, Loma Linda, USA ^2^Loma Linda University, Radiation Medicine, Loma Linda, USA

Photon-based radiotherapy in combination with cisplatin is the standard of care for patients with locally advanced HNSCC and it causes significant treatment-related sequelae. The HPV-associated HNSCC patient cohort has favorable survival outcomes and this has prompted clinicians to consider de-escalating this intensive regimen, particularly for patients with HPV^+^-oropharyngeal SCC. De-intensification through cisplatin replacement with cetuximab (an EGFR-targeted antibody) has therefore been pursued recently in various clinical trials. Unfortunately, early results show inferior survival outcomes when cetuximab is used in place of cisplatin in combination with radiation. This indicates that there is an urgent need for novel targeted agents that can safely enhance the effective dose of radiation. To this end, we investigated the effects of our recently discovered, novel HPV E6 inhibitor on radio-sensitizing HPV^+^ and HPV^-^ HNSCC cell lines to photon and proton radiation. Radiosensitivity was assessed using various assays such as apoptosis, cell cycle and survival. Dose enhancement ratios (DERs) were calculated using clonogenic survival as the endpoint. Our E6 inhibitor reduced colony formation and increased apoptosis induction in an HPV-dependent manner. Significantly higher DER values were obtained for HPV^+^ cells compared to their HPV^-^ counterparts. The relative biological effectiveness (RBE) values of HPV^+^ cells were also generally higher compared to HPV^-^ cells upon treatment with proton radiation. More work in the future is warranted to further characterize the interaction of the E6 inhibitor with radiation and its potential to improve the safety and effectiveness of radiation treatment for patients with oropharyngeal cancer.

## O142 -

### Influence of alpha-particle radiation on intercellular communication networks of tunneling nanotubes in U87 glioblastoma cells

#### Nicole Matejka^1^, Judith Reindl^1^

##### ^1^Universität der Bundeswehr München, Institut für angewandte Physik und Messtechnik, Neubiberg, Germany

The aggressive nature of glioblastoma, a common brain tumor, is composed of several features like uncontrolled cell-growth, high infiltration rates and their strong ability to develop therapy resistance. For their organization, effective cell-to-cell communication among the cancerous cells is essential. One remarkable communication mechanism of cells is tunneling nanotubes (TNTs). These ultra-fine membrane connections with a diameter from 50 to 1500 nm enable cells to network very strongly with each other and thus ensure their survival. Here, we study the response of TNT communication networks in glioblastoma cells on radiative stress induced by α-particle radiation. The aim was to figure out whether cellular TNT-networks are influenced by radiation and if cellular communication is enhanced upon radiation treatment. U87 glioblastoma cells were homogeneously irradiated with high-LET α-particles to a dose of 1.2 Gy. After post-irradiation incubation times up to 72 h, the cell membrane was labeled and the TNT-network was examined using live-cell confocal microscopy. In our study, we suggest an evaluation method to characterize these communication networks and describe the development of TNT-networks after radiation treatment. Our results show that irradiated cells establish their network faster and have more cell-to-cell connections with a high TNT content than sham irradiated controls within the first 24 h. These findings indicate that cancer cells respond with a fast and intensive TNT-network formation to radiation and the development of such a resistant communication network could be a responsible cellular mechanisms for therapy resistance.

## Poster Abstracts P001 -

### Laser-hybrid accelerator for radiobiological applications (LhARA)

#### Titus-Stefan Dascalu ^1^

##### ^1^Imperial College London, Department of Physics, London, United Kingdom

The ‘Laser-hybrid Accelerator for Radiobiological Applications', LhARA, is conceived as a novel, uniquely flexible facility dedicated to the study of the biological response to ionising radiation. With the potential to deliver multiple ion species in beams with a wide range of temporal and spatial profiles, and at ultra-high dose rates, LhARA will enable the exploration of a completely new regime of particle-beam therapy. The high flux, short bunch duration, and high repetition rate are well-suited to study the radiobiological mechanisms by which the therapeutic benefit is generated. The new approach is based on a high-power laser which creates a large flux of protons or light ions from a foil target. The particles are captured and focused using electron plasma lenses, thus evading the current limits on the maximum instantaneous dose rate. LhARA will be developed in two stages. In the first stage, a programme of in vitro experiments will be served with proton beams with energies between 10 MeV and 15 MeV. In stage two, rapid acceleration will be performed using a fixed-field alternating-gradient (FFA) accelerator. A high-energy in vitro end station and an in vivio end station will be served by proton beams with energy up to 127 MeV. In addition, ion beams, with energies up to 30 MeV per nucleon for carbon, will be available for in vitro and in vivo experiments. This paper presents the conceptual design for LhARA and the R&D programme that is required to demonstrate the feasibility of critical LhARA components.

## P002 -

### A new preclinical proton minibeam facility: physics and technological challenges

#### G. Datzmann^1,2^, G. Dollinger^2^, M. Mayerhofer^2^, J. Reindl^2^

##### ^1^Datzmann interact & innovate, Proton Therapy, München, Germany ^2^Universität der Bundeswehr München, Institute for Applied Physics and Measurement Technology, München, Germany

Proton minibeam radiotherapy (pMBRT) has shown several promising results with regards to tissue sparing effects and tumor control capability in the past decade. However, the preclinical experiments conducted up to now addressed only a small set of specific tumor targets and types of healthy tissues. Progressing towards clinical usage, pMBRT research should overcome technological and biomedical limitations of the current irradiation test stages. Therefore, we aim for a new facility that is feasible to perform small animal pMBRT offering a large flexibility concerning the most important beam characteristics i.e. beam energy, beam sizes and peak to valley dose ratios. Using such a versatile tool enables to understand radiobiological mechanisms and to fully exploit the therapeutic potential of pMBRT. In addition, dealing with other new opportunities and challenges in modern radiotherapy such as FLASH-Irradiation, hypofractionation or compensation of moving targets, the treatment system technology shall allow the dose rate to be adaptable over many orders of magnitude. This work discusses the achievable beam parameters with respect to the technology used for accelerating and forming proton minibeams. In particular, an accelerator concept based on an RF linac system will be presented that is generating a magnetically focused proton minibeam at 70 MeV. Comprehensive beam-transport simulations demonstrate beam sizes of approx. 0.1 mm at the skin of a small animal in combination with high peak to valley dose ratios and high dose rates.

## P003 -

### The first modeling of the spot-scanning proton arc (SPArc) delivery sequence and investigating its efficiency improvement in the clinical treatment workflow

#### G. Liu^1,2^, L. Zhao^1^, D. Yan^1^, R. Deraniyagala^1^, C. Stevens^1^, X. Li^1^, X. Ding^1^

##### ^1^Beaumont Health System, Radiation Oncology, Royal oak, USA ^2^Union Hospital Tongji Medical College- Huazhong University of Science and Technology, Cancer Center, Wuhan, China

**Purpose:** To quantitatively model a precise spot-scanning proton arc(SPArc) delivery sequence and assess its efficiency improvement in the routine proton clinical operation.

**Method and material:** The SPArc delivery sequence model(DSM_SPArc_) includes two kinds of parameters:(1) mechanical parameters (gantry velocity and acceleration speed). (2) irradiation parameters (tolerance window and buffer, spot and energy layer scanning sequence). An independent gantry inclinometer was used to measure mechanical parameters. Logfiles were used to derive the irradiation parameters through test plans. The DSM_SPArc_ was established by fitting both mechanical and irradiation parameters. Eight SPArc plans from different disease sites were used to validate the DSM_SPArc_'s accuracy. To quantitatively assess the treatment efficiency improvement, a random clinical operation date of our proton center(total 21 cases on Jan 6^th^ 2021) was selected, and SPArc plans were generated for all the cases. The DSM_SPArc_ simulated the SPArc treatment delivery and compared it to the clinical IMPT logfiles.

**Result:**The relative difference of treatment time between log files and DSM_SPArc_'s prediction was 6.1%±3.9%, and the arc delivery sequence showed a good agreement between the DSM_SPArc_ and logfile(Fig 1). Additionally, the SPArc plan could effectively save two hours from a 10-hour clinical operation day by simplifying the treatment workflow for a single room proton therapy center. The average treatment delivery time per patient was reduced to 226±149s using SPArc compared to 665±407s using IMPT (p<0.01)(Fig 2).

**Conclusion:** This is the first modeling of the SPArc delivery sequence. Additionally, SPArc can offer a superior delivery efficiency to improve clinical treatment throughput, compared to IMPT.

## P004 -

### Development of an adjustable collimator for microbeam irradiations in proton beams for radiobiological and dosimetric studies

#### Sam Flynn^1,2^, Philip Allport^1^, Oscar Ayress^1^, Harry Bennion^1^, Geoffrey Niu^1^, Ben Phoenix^1^, Edward Taylor^1^, Stuart Green^3^, Ileana Silvestre Patallo^4^, Anna Subiel^2^, Russell Thomas^2^, Tony Price^1,2^

##### ^1^University of Birmingham, Particle Physics, Birmingham, United Kingdom ^2^National Physical Laboratory, Medical Radiation Science, Teddington, United Kingdom ^3^University Hospital Birmingham NHS Trust, Department of Medical Physics, Birmingham, United Kingdom ^4^National Physical Laboratory, Medical Radiation Physics, Teddington, United Kingdom

Spatial Fractionation in radiotherapy is an emerging technique which is indicated to have preferential normal tissue sparing relative to conventional radiotherapy. Preclinical studies have indicated that this dose pattern has a greater efficacy than that of a single uniform field but a substantial amount of research is still required before translation into routine clinical practices. Spatial fractionation in protons is theorised to have additional benefits as multiple Coulomb scattering can be exploited to maintain a uniform dose distribution in the tumour volume. This project is developing an adjustable microbeam collimator for low energy proton microbeam irradiations with an ambition of establishing a test facility for cellular irradiations and the development of novel dosimetry techniques. Using the University of Birmingham's MC40 cyclotron and a Monte Carlo optimised tantalum collimator with 100 μm slits we present a novel system capable of delivering a variety of Peak-to-Valley Dose Ratio (PVDR) configurations. By installing two tantalum collimators at variable separation along a fixed rail, as shown in Figure 1, it becomes possible to repeatably modify the proton microbeams generated. Furthermore, by rotating one collimator such that it is orthogonal to the other, we can generate grid-microbeams with 100 μm nominal square sides. Assembly of the collimator system is expected to occur in March 2021, with commissioning completed by end of May 2021 in anticipation of making cell irradiations for preclinical research.

## P005 -

### Solute ion heavy ion beam cancer therapy via CO2 in H2O forming H+ and HCO3-ion beams

#### A.N. Fresco ^1^

##### ^1^Anthony N FRESCO, Solute Ion Linear Alignment Acceleration, Port Jefferson Station, USA

Linear alignment of ions results in vector alignment of Coulomb forces to create an ion jet for particle acceleration. An Electric Force Example provides a very dramatic illustration of Coulomb forces: Hyperphysics.phy-astr.gsu.edu/hbase/electric/elefor.html. SILA should provide a very inexpensive ion beam method that may replace expensive proton accelerators in proton therapy centers and thus even rural hospitals globally may be able to use ion beams for cancer therapy. An abstract accepted for the PTCOG57-2018 Conference in Cincinnati, Ohio, offered the idea that sodium and chlorine ion beams could separate CO2 into carbon and oxygen ions and subsequently into linearly aligned ion beams. Dissolving CO2 into plain water H2O could create protons H+ ion beams and HCO3- ion beams which would be far heavier (HCO3- molecular weight 61, proton 1 gm/gm-atomic weight) than protons and about 4 to 5 times heavier than carbon or oxygen alone. Bragg Peak would be even greater thereby beneficially depositing even more energy in the tumor and not surrounding tissue. Since SILA produces one positive beam and one negative beam, the beams can be merged for a generally neutral beam if advantageous. SILA beam technology may significantly facilitate applications for FLASH, mini-beams and ultra-hypofractionation and studying the effects of oxygen. With a magnetic Wiggler, it should be possible to create intense UV and other light for COVID-19 and mutation sterilization and potential new light-based and ion beam cancer therapies with new compounds accumulating in tumors similar to BNCT.

## P006 -

### Fast energy modulation within the beamline momentum acceptance: a clinical feasibility study

#### A.C. Giovannelli^1,2^, D. Meer^1^, S. Safai^1^, B. Christian^1^, V. Maradia^1^, S. Psoroulas^1^, T. Michele^1^, D.C. Weber^1,3^, A.J. Lomax^1,2^, G. Fattori^1^

##### ^1^Paul Scherrer Institute, Center for proton therapy CPT, Villigen, Switzerland ^2^ETH Zürich, Physics, Zürich, Switzerland ^3^University Hospital Bern, Radio-Onkologie, Bern, Switzerland

Pencil beam scanning proton therapy is delivered as a sequence of iso-energy dose layers, changing progressively while scanning across the target. Changing energy requires changing magnetic fields in slow-settling beamline magnets; to reduce the number of changes, we investigated into modulating proton energy within the beamline momentum acceptance of a clinical gantry. We analysed clinically significant parameters (beam position, shape, range, transmission) for a relevant set of particles off-momentum. After introducing dedicated correction models, the measured beam data was within 1.2% beam momentum acceptance of the institutional reference data (depth-dose γ:1%,1mm>90%) with clinically acceptable beam position accuracy and intensity at isocenter. The concept has been clinically tested on a single treatment field (cranial glioma) optimised to deliver 1.8 GyRBE using 48 energy layers. An additional range shifter was used in order to operate high beam energy where the beam momentum spread is smaller than the acceptance of the beamline. We found negligible distortions in the delivered dose, verified at three depths with a cross-calibrated array of ionisation chambers. Largest discrepancies with respect to clinical settings were measured for a proximal layer located in a region of steep dose gradients, albeit still within the clinical tolerance (γ:3%,3mm>99%). Overall, 16 energy changes were skipped, resulting in a 12% reduction of delivery time on a machine with nominal energy switching times about 100ms. Exploiting beam momentum acceptance is a technical option for reducing energy change dead-times and could be an interesting upgrade for facilities with slow energy switching time without ad-hoc beamline modifications.

## P007 -

### A scanning dynamic pinhole aperture to reduce spot sizes in proton therapy

#### Jason Holmes^1^, Samir Patel^1^, William Wong^1^, Robert Foote^2^, Wei Liu^1^

##### ^1^Mayo Clinic, Department of Radiation Oncology, Phoenix, USA ^2^Mayo Clinic, Department of Radiation Oncology, Rochester, USA

**Purpose:** To improve nearby normal tissue protection in proton therapy, the spot size must be carefully controlled and reduced if possible. We propose the concept of dynamic pinhole aperture (DPA) (Figure 1). In this work, we investigate the clinical viability of the DPA based on its ability to reduce spot sizes while retaining a reasonable dose rate.

**Methods:** The DPA, attached to a range shifter, has a single cylindrical opening that follows and trims each spot. The DPA has been modelled in MCsquare. The resulting spot sizes are compared between the DPA, the optimal thickness range shifter, and the extended range shifter (ERS). The beam transmission ratio is characterized for the DPA, on- and off-axis, to indicates the reduction of the dose rate.

**Results:** Compared to ERS, spot sizes are reduced 50% when the DPA is placed 5 cm from the patient at depths less than 50 mm (Figure 2). For a DPA of 3 mm radius positioned 50 mm from the patient, the spot size is reduced by 30% compared to an optimal range shifter placed at the same position. Depending on the beam energy and DPA positioning, the beam transmission ratio is between 10-50% for a 3 mm radius DPA.

**Conclusions:** The device is simple, compact, and effective, especially for shallow tumors. Since the DPA adds treatment time for each spot, its use should be limited to a subset of spots of the treatment plan where the reduction in spot sizes is most beneficial.

## P008 -

### Application of lung-substitute material as a ripple filter for multi-ion therapy with helium-, carbon-, oxygen-, and neon-ion beams

#### Taku Inaniwa^1^, Yasushi Abe^1^, Masao Suzuki^2^, Sung Hyun Lee^1^, Kota Mizushima^1^, Taku Nakaji^3^, Shinji Sato^1^, Yoshiyuki Iwata^1^, Nobuyuki Kanematsu^1^, Toshiyuki Shirai^1^

##### ^1^National Institute of Radiological Sciences- QST, Department of Accelerator and Medical Physics, Chiba, Japan ^2^National Institute of Radiological Sciences- QST, Department of Basic Medical Sciences for Radiation Damages, Chiba, Japan ^3^QST Hospital- National Institute of Radiological Sciences- QST, Quality Assurance Division, Chiba, Japan

A development project for hypo-fractionated multi-ion therapy has been initiated at NIRS. In the treatment, helium, carbon, oxygen, and neon ions will be used as primary beams with pencil beam scanning. A ripple filter (RiFi), consisting of a thin plastic or aluminum plate with a fine periodic ridge and groove structure, has been used to broaden the Bragg peak of heavy-ion beams in the beam direction. To sufficiently broaden the Bragg peak of the beams with suppressed lateral scattering and surface dose inhomogeneity, we tested a plate made of a lung substitute material, Gammex LN300, as the RiFi. The planar integrated dose distribution of a 183.5-MeV/u neon-ion beam was measured behind a 3-cm-thick LN300 plate in water. The Bragg peak of the pristine beam was broadened following the normal distribution with the standard deviation value of 1.29 mm, while the range of the beam was reduced by 8.8 mm by the plate. To verify the LN300 performance as the RiFi in multi-ion therapy, we measured the pencil beam data of helium-, carbon-, oxygen, and neon-ion beams penetrating the 3-cm-thick LN300 plate. The data were then modeled and used in a treatment planning system to achieve a uniform 10% survival of HSGc-C5 cells within a cuboid target by the beam for each of the different ion species. The measured survival fractions were reasonably reproduced by the planned ones for all the ion species. The LN300 plate is applicable as the RiFi in multi-ion therapy with helium-, carbon-, oxygen, and neon-ion beams.

## P009 -

### New gantry beam optics solution for minimizing treatment time in cyclotron-based proton therapy facilities

#### Vivek Maradia^1,2^, David Meer^1^, Anna Chiara Giovannelli^1,2^, Antony John Lomax^1,2^, Damien Charles Weber^1,3,4^, Jacobus Maarten Schippers^1^, Serena Psoroulas^1^

##### ^1^Paul Scherrer Institut, Center for Proton Therapy, Villigen, Switzerland ^2^ETH Zurich, Physics, Zurich, Switzerland ^3^University Hospital Bern, Medical, Bern, Switzerland ^4^University Hospital Zurich, Medical, Zurich, Switzerland

Treatment delivery time in proton therapy depends on beam-on time and the time required to change energy layers and/or lateral position. For cyclotron-based facilities, low energy beams (100-70 MeV) are inefficiently transported through beamlines due to their large emittance after the degrader (∼400 pi*mm*mrad 2-sigma emittance), whereas the beamline and Gantry can only transport small emittances (e.g. 30 pi*mm*mrad for PSI Gantry 2) resulting in a low dose rate at the patient and increased beam-on time. In this work, we aim to maximize the emittance transported through the gantry for low energy beams. By choosing a small divergence, but large beam size, at the gantry entrance, it is possible to transport higher emittances through the gantry without compromising transmission. Additionally, in order to retrieve small beam sizes at the patient, we propose a 2:1 imaging of the gantry beam optics between the gantry coupling point and the patient. This concept has been experimentally validated on Gantry 2 at PSI. A beam with an emittance of 90 pi*mm*mrad and ∼60% transmission was transported through the gantry. As we are transporting only a narrow part of the large Gaussian beam after the degrader, this 3 times higher emittance corresponds to ∼3 times more transported particles. With this, treatment times for example cases (lung and liver) have been estimated to reduce by a factor of 2 to 3. Such a beam optic could therefore have substantial potential for reducing treatment times, and be of particular advantage for the treatment of moving targets.

## P010 -

### Design of heavy ion radiotherapy system configuration considering the interface to the facility

#### Ryotaro Masuda ^1^

##### ^1^Toshiba Energy Systems & Solutions Corporation, Third Advanced System Design & Engineering Group, Kawasaki, Japan

This presentation shows the outline of Toshiba's heavy ion radiotherapy system and the interface requirements between the system and the facility. Toshiba has been providing heavy ion radiotherapy system and contributing to cancer therapy. And Toshiba has developed and incorporated new technologies in the system configuration, such as 3D high-speed scanning, compact superconducting iso-centric rotating gantry, and so on. They will bring some benefits like shorter treatment time and load reduction of patient. In order to supply the customers with high-quality radiotherapy system, the interface in various utilities with the facility and the facility environment are essential as well as the equipment. As the beam control is affected by the facility environment, the completion of all the requirements in the interface conveys the best performance of the equipment as initially planned. Toshiba also pays attention to the interface to the facility and optimize the system design under customer cooperation for providing smooth and continual treatment.

## P011 -

### Comparison of three acceleration methods for GATE Monte Carlo simulation of passive scattering delivery

#### Elham Piruzan^1^, Naser Vosoughi^1^, Hojjat Mahani^2^

##### ^1^Sharif University of Technology, Department of Energy Engineering, Tehran, Iran Islamic Republic of ^2^Nuclear Science and Technology Research Institute, Radiation Applications Research School, Tehran, Iran Islamic Republic of

While the GATE Monte Carlo simulator provides promising features in proton beam therapy, its flexibility comes at a cost as it is highly computationally intensive. Therefore, improving GATE simulation efficiency is of importance to keep the computing time at an acceptable level. To do so, Mevion S250 passively scattered beam delivery nozzle was completely modeled within the GATE toolkit. The investigated speed-up methods were (1) secondary particles range cutoff, (2) virtual beam modulation using the user-defined source energy spectrum, and (3) virtual beam collimation using the built-in kill track actor. Then, the acceleration factor associated with each technique was compared considering the same statistical uncertainty of less than 1%. A reference GATE simulation was also performed for a range cutoff of 1 μm. Furthermore, the GATE model was validated against published measurements. Among them, the 1 mm range cutoff results in a superior performance by exhibiting an acceleration factor of 18.85. The virtual collimation and modulation approaches lead to the 2.65 and 1.80 acceleration factors, respectively, for a modulation width of 3.5 cm at 6.0 cm depth in a water phantom. The maximum difference between reference dose profiles and that of the cutoff technique was less than 7%. In contrast, the virtual collimation and modulation techniques offer approximately the same dose distribution in the water phantom compared to the reference GATE. Acceleration techniques significantly shorten computation time while the applicability of the GATE platform is retained. A suitable acceleration method can be chosen depending on the required speed and accuracy.

## P012 -

### Development of the Low Intensity Extraction Beam Control System for Proton Radiography Purposes at Protom Synchrotron

#### Alexander Pryanichnikov^1,2,3^, Pavel Zhogolev^1,2^, Alexander Shemyakov^1,2^, Michail Belikhin^1,2^, Alexander Chernyaev^3^, Victor Rykalin^4^

##### ^1^Protom Ltd., Research and Development Department, Protvino, Russian Federation ^2^Lebedev Physical Institute RAS, Physical-Technical Center, Protvino, Russian Federation ^3^Lomonosov Moscow State University, Accelerator Physics and Radiation Medicine Department, Moscow, Russian Federation ^4^ProtonVDA, Research and Development Department, Chicago, USA

Protom synchrotron is a medical accelerator that was specially designed for proton therapy. The synchrotron has an opportunity to accelerate protons to 330 MeV. This fact makes proton imaging of the entire human body available without any restrictions. Using of proton imaging will allow us to escape of proton range uncertainties in the patient body and will make the treatment process more accurate. Moreover, proton radiography can be used as a patient position verification tool instead of standard CBCT systems. The proton imaging system has a lower equivalent dose that is received by the patient in comparison with similar X-ray imaging systems. However, proton imaging systems can't work with beam intensity that is used in standard proton therapy. So, there is a need to decrease the proton intensity for this purpose. This study demonstrates the current version of the new beam control system for low proton intensity extraction. The system is based on automatic removable unit with special luminescence film and sensitive photoreceptor. Using of the removable module allows us to save initial parameters of the therapy beam. Remote automatic control of this unit will provide switch therapy and imaging modes between synchrotron cycles. The work describes algorithms of low flux beam control, calibration procedures and experimental measurements. Measurements and calibration procedures were performed with certified Protom Faraday Cup, PTW Bragg Peak Chamber and specially designed experimental external detector based on plastic scintillator and PhM. The developed system can be implemented in any proton therapy complexes based on the Protom synchrotron.

## P013 -

### Commissioning of a clinical pencil beam scanning proton therapy unit for ultrahigh dose rates (FLASH)

#### Serena Psoroulas^1^, Konrad P. Nesteruk^1^, Michele Togno^1^, Jeppe B. Christensen^2^, Martin Grossmann^1^, Marco Schippers^3^, Antony J. Lomax^1,4^, Damien C. Weber^1,5,6^, Sairos Safai^1^, David Meer^1^

##### ^1^Paul Scherrer Institut, Centre for Proton Therapy, Villigen PSI, Switzerland ^2^Paul Scherrer Institut, Department of Radiation Safety and Security, Villigen PSI, Switzerland ^3^Paul Scherrer Institut, Department Large Research Facilities, Villigen PSI, Switzerland ^4^ETH, Department of Physics, Zürich, Switzerland ^5^University Hospital Bern, Department of Radiation Oncology, Bern, Switzerland ^6^University Hospital Zurich, Department of Radiation Oncology, Zurich, Switzerland

**Aims**: To convert the former clinical treatment unit Gantry 1 into a proton FLASH delivery unit.

**Methods**: PSI Gantry 1 treated patients until December 2018. We optimized the beamline parameters to transport the 250 MeV beam extracted from the accelerator to the treatment room, maximizing beam intensity transmission. We characterized the dose monitors on the gantry to ensure high dose accuracy in spot-scanning delivery.

**Results**: We achieved 86% beam transmission to the sample position ('isocenter', Fig. 1). In FLASH irradiations, we reached a peak dose rate of 9000 Gy/s along the central axis of a single pencil beam (Fig.2). The clinical dose monitor showed large recombination effects (> 2%) at dose rates higher than 100 Gy/s, which we characterized against dose-rate-independent dose measurements, performed with a Faraday cup, achieving a 2% dose accuracy in FLASH irradiations. To validate the dose fields delivered to the samples, we performed dose measurements at the maximum dose-rate points in a water phantom using passive dosimeters. Additionally, we tested fast 2D pencil beam scanning aiming at the delivery of small fields in FLASH conditions.

**Conclusions**: We have realized a proton FLASH irradiation setup able to investigate continuously a wide dose rate spectrum, from below 1 to 9000 Gy/s in a single spot and in pencil beam scanning mode. As such, we have developed a versatile test bench for FLASH research.

**Acknowledgements**: we thank our collaborators at CHUV (M.-C. Vozenin). KPN's work was funded by the Swiss National Science Foundation (grant n. 190663).

## P014 -

### Monte Carlo evaluation of neutron dose equivalent to patients treated with Vertex Beams in IMPT and PSPT

#### Uwe Titt^1^, David Grosshans^2^, Narayan Sahoo^1^, Xiaorong Ronald Zhu^1^, Radhe Mohan^1^

##### ^1^University of Texas- M. D. Anderson Cancer Center, Radiation Physics, Houston, USA ^2^University of Texas- M. D. Anderson Cancer Center, Radiation Oncology, Houston, USA

Patients treated with protons are subjected to secondary neutrons originating from the beam delivery system as well as in the patient. Application of Vertex beams, i.e., proton beams oriented in the superior-inferior direction raised concerns of increased neutron exposure to the patients due to the penetrating nature of these particles. A study was performed to assess the neutron dose equivalent to patients treated with such beam configurations. A Monte Carlo model of the complete treatment room, including all relevant beam line equipment was produced for IMPT and PSPT treatments, each equipped with a simplified representation of an adult and a 10 year old pediatric patient. Proton beams of about 15 cm range in water were simulated to the heads of the phantoms in the Vertex Beam orientation and from a lateral direction. The neutron fluence in the phantom's arms, legs, torso, as well as head and neck were scored as a function of neutron energy and converted to neutron dose equivalent. The IMPT treatments yielded moderately reduced neutron dose equivalent values for Vertex Beams, mostly to the neck of the phantoms. Using PSPT, there was a noticeable systematic reduction in neutron dose equivalent in the Vertex Beam configuration. We found that the neutron dose equivalent to the phantoms was more dependent on the distance to the beam line/nozzle than on beam direction, and that the application of Vertex Beams does not pose additional hazards to a proton therapy patient due to neutron exposure.

## P015 -

### Ultra-sensitive non-interceptive monitors for beam current and position measurements at the proton therapy facility at PSI

#### Sudharsan Srinivasan^1^, Pierre-André Duperrex^1^, Jacobus Maarten Schippers^1^

##### ^1^Paul Scherrer Institute, GFA, Villigen, Switzerland

A superconducting cyclotron “COMET” at PSI (Paul Scherrer Institute) delivers a pulsed proton beam of 250 MeV at 72.85 MHz for proton radiation therapy. Measurement of proton beam current (0.1-40 nA) and its position are of crucial importance and are performed regularly with invasive monitors such as ionization chambers. We have developed two types of non-interceptive monitors. A new macor-filled cavity resonator working on TM010 mode resonance for beam current measurement and an alumina-filled quadrated cavity resonator working on TM110 mode resonance for beam position measurement has been developed to replace ionization chambers thus preventing associated scattering issues and preserving delivered beam quality. To prevent interference of the RF signal, both resonators are tuned to the 2^nd^ harmonic of the beam repetition rate i.e. 145.7 MHz. The beam current monitor has been validated with intensity sweeps for energies of 79-231 MeV with an existing measurement chain. There is good agreement between the expected and measured resonator calibration factor for the beam current monitor as a function of energy. Similarly, the beam position monitor has been validated with intensity and position sweeps for energies of 138 and 200 MeV with a spectrum analyzer. Also, the beam position monitor behaves as expected with respect to simulation. We will summarize the achievements, such as the lowest measurable beam intensity with the beam current monitor and the position resolution of the beam position monitor.

## P016 -

### Adjustment of the lateral size of line-scanned proton beam using multiple thickness of range shifter

#### Y. Sugama^1^, A. Masayuki^1^, H. Fujimoto^1^, Y. Seki^1^, T. Yamaguchi^2^, G. Shibagaki^2^, T. Nishio^3^, H. Onishi^4^

##### ^1^Aizawa Hospital, Proton Therapy Center, Matsumoto, Japan ^2^Sumitomo Heavy Industries- Ltd., Industrial Equipment Division, Shinagawa, Japan ^3^Osaka University Graduate School of Medicine, Division of Health Sciences, Suita, Japan ^4^Yamanashi University, Department of Radiology, Chuo, Japan

In Aizawa Hospital, 40 mm-thick range shifter (RS) is used for treating shallow targets with line scanning technique. It has been reported that the depth at which the beam size is minimized depend on the thickness of RS. Therefore, there is a possibility that the beam size can be optimized by using multiple thickness of RSs. In this study, we investigated a simple method of combining RSs of two different thicknesses. The purpose of this study is to evaluate the beam size variation due to the difference in RS thickness and to determine the optimal RS thickness for use in combination with 40 mm RS. We used Monte Carlo simulation (MCS) to predict the beam size variation. The beam size at the depth of Bragg Peak was simulated by changing the beam energy and the RS thickness. In some RS thickness, Eclipse was used to create a treatment plans for a cubic target which were centered at a depth of 20 mm and 170 mm. Furthermore, the penumbra widths at the depth of target center were compared. Figure 1 shows the result of MCS. We think that the combination of 40 mm RS and 100 mm RS is optimal for use of small size beams in wide range depth. Table 1 shows the result of comparing the penumbra widths. From this result, we consider that beam size can be optimized by using 40 mm RS and 100 mm RS in combination. The usefulness of using multiple thickness of RSs was shown.

## P017 -

### Development status of the SHI superconducting cyclotron (sc230) for proton therapy

#### Takehisa Tsurudome^1^, Masayuki Hirabayashi^2^, Hiroshi Tsutsui^3^, Yuta Ebara^1^, Jun Yoshida^1^, Hirohiko Murata^1^, Nobuaki Takahashi^1^, Yoshihiko Arakawa^1^, Nagaaki Kamiguchi^1^, Takaaki Morie^1^, Takuya Miyashita^2^, Kazuya Taki^2^, Toshiki Tachikawa^2^, Yukio Mikami^1^, Yukio Kumata^1^

##### ^1^Sumitomo Heavy Industries- Ltd., Technology Research Center, Yokosuka, Japan ^2^Sumitomo Heavy Industries- Ltd., Industrial Equipment Division, Niihama, Japan ^3^Sumitomo Heavy Industries- Ltd., Industrial Equipment Division, Tokyo, Japan

A superconducting cyclotron for proton therapy is being developed at Sumitomo Heavy Industries (SHI). The cyclotron weight is about 65 tons, and a diameter is 2.8 meters. It was designed to meet various clinical needs with a beam energy of 230 MeV and a maximum current of 1000 nA. In particular, it can be combined with high-speed scanning to enable very short irradiation times. Magnetic fields of the cyclotron have been measured in 2019. The magnetic field distribution and parameters such as horizontal and vertical tunes agreed well with the original design. After the magnetic field distribution measurement, all components of the cyclotron were assembled, including two RF cavities, ion source, central region, electrostatic deflector, two magnetic channels, eight harmonic coils, beam monitoring system, and vacuum pumping system. A new solid-state RF amplifier was fabricated in 2020 and the RF system was confirmed to be at its design value of 95.2 MHz frequency and 50 kV DEE voltage. Based on the results, beam tests have been started. In this paper, the status of the development and the results of beam tests are presented.

## P018 -

### Development of scanning magnet with distributed winding coils for particle beam therapy

#### T. YAMADA^1^, K. Nagashima^1^, T. Nomura^2^, S. Soeda^2^, N. Hino^1^

##### ^1^Hitachi- Ltd., Research & Development Group, Hitachi-shi- Ibaraki, Japan ^2^Hitachi- Ltd., Smart Life Business Management Division, Kashiwa-shi- Chiba, Japan

To reduce the size of a rotating gantry for a particle therapy system, it is desirable to reduce the size of the scanning system by substituting separated scanning magnets with a combined-function scanning magnet. We propose a new type of combined-function scanning magnet with distributed winding coils commonly used in electric motors (DW-SCM). The DW-SCM can generate a rotating dipole magnetic field using a three-phase sinusoidal current (Fig. 1). The two-dimensional static magnetic field distributions of the DW-SCM were calculated using Poisson Superfish code (Fig. 2) and compared with those of an octupole scanning magnet (CW-SCM) and cos-theta-type scanning magnet (Cos θ-SCM) with the same bore diameter of 98 mm. The calculated two-dimensional magnetic field showed that the DW-SCM had the highest magnetic field strength of the three types of scanning magnets, and compared with the CW-SCM, the DW-SCM was 229% higher and the Cos θ-SCM was 172% higher. The good field region (radius) of field homogeneity within ± 0.5% of the DW-SCM had a symmetrical shape centered on a current phase of 30°, and the minimum value was 24 mm. Also compared with the CW-SCM, the minimum good field region was 111% higher for the DW-SCM and 165% higher for the Cos θ-SCM. The results indicate that the proposed scanning magnet could generate a high-strength rotating dipole magnetic field, and it might be possible to reduce the size of a scanning system.

## P019 -

### Precise magnetic field measurements of high-gradient permanent quadrupole focusing magnets

#### Elijah Yap^1^, Anthony Teran^2^, Andrew Wroe^3^, Jerry Slater^2^, Grant Mcauley^2^

##### ^1^Loma Linda University, School of Medicine, Loma Linda, USA ^2^Loma Linda University, Radiation Medicine, Loma Linda, USA ^3^Miami Cancer Institute, Radiation Oncology, Miami, USA

Work in our laboratory has shown that focusing protons with quadrupole Sm2Co17 permanent magnetic material adhered into Halbach cylinders could allow irradiation of small targets with fewer beams, lower entrance dose, and shorter treatment times. These magnets can also focus protons into planar beamlets to form spatially fractionated minibeams. While previous Monte Carlo simulations have used idealized mathematic equations to describe the quadrupole field, the purpose of this project is to develop a system to precisely map the actual fields of our magnets. The strong fields and large gradients inside the cylindrical magnet bore require the precise placement of a probe with a small active element for accurate and reproducible measurements. Three linear tracks (micro step-size ∼0.124 μm) were bolted together to create a computer-controlled precision 3D positioning system. Python code was developed to synchronize measurements by a teslameter with automatic, rapid positioning of a Hall probe, while simultaneously preventing collisions with the magnet surface. The program employs a menu-driven interface allowing selection of teslameter settings, magnet dimensions, measurement volume, separate resolutions for measurement points internal and external to the magnet bore (Fig 2), creation of a 3D visualization of measurement points (Fig 2) and output of field measurement data as a CSV file. Ongoing work includes design and fabrication of a suitable field probe holder and thorough end-to-end testing. Once in service, the accurate field maps will help produce more accurate computer simulations, inform potential experiments with our magnets, and provide insight regarding associated patient safety.

## P020 -

### Development of a Monte Carlo simulation model of the Clatterbridge Cancer Centre 60 MeV ocular proton therapy beamline in TOPAS

#### J.S.L. Yap^1,2^, A. Kacperek^3^, M. Brooke^4^, S. Jolly^5^, C. Welsch^1,2^

##### ^1^University of Liverpool, Physics, Liverpool, United Kingdom ^2^Cockcroft Institute, Physics, Warrington, United Kingdom ^3^Clatterbridge Cancer Centre, Douglas Cyclotron, Wirral, United Kingdom ^4^Oxford University, Oncology, Oxford, United Kingdom ^5^University College London, Dept of Physics & Astronomy, London, United Kingdom

The Clatterbridge Cancer Centre (CCC) in the United Kingdom is the world's first hospital proton beam therapy facility, having successfully provided treatment for ocular cancers over the past 32 years. A 60 MeV proton beam with a sharp penumbra and fall off is delivered by a passive delivery system, shaping the beam specifically for the needs of each treatment. A model of the beamline has been built using TOPAS, a specialised simulation platform for proton therapy based on the Monte Carlo toolkit, Geant4. The treatment line is constructed using the exact physical descriptions of each component as based on CAD imports and user defined geometry, representing the most comprehensive and realistic model of the CCC facility to date. This includes precise definitions of the vacuum beam tube containing the double scattering foils, modulator and dosimetry boxes, ion chambers and the patient nozzle. The parameters of the particle source are determined by optical modelling of the beam transported from the cyclotron through to the scattering system. Measurements were also carried out to validate the accuracy of the simulation model including depth dose and transverse profiles. The source code and documentation will be made available for wide use, as a verified standard simulation model for all related work performed with the Clatterbridge proton therapy beamline. Further developments with this TOPAS CCC model also explore its application in radiobiological studies and additional capabilities for end-to-end modelling.

## P021 -

### Assessment of a treatment planning system for carbon-ion radiotherapy using Monte Carlo simulation: a preliminary study

#### Yongdo Yun^1^, Hyo Kyeong Kang^1^, Min Cheol Han^1^, Sung Hyun Lee^2^, Takeo Iwai^2^, Jin Sung Kim^1^

##### ^1^Yonsei University College of Medicine, Department of Radiation Oncology, Seoul, Korea Republic of ^2^Yamagata University Graduate School of Medical Science, Department of Heavy Particle Medical Science, Yamagata, Japan

**Purpose:** The objective of this project is to assess a commercial treatment planning system (TPS) used at Yonsei Cancer Center and Yamagata University Hospital compared with a general-purpose Monte Carlo (MC) simulation in terms of both physical and biological aspects. In this study, as a preliminary study, the TPS and MC-based models were commissioned based on measurement data obtained from carbon-ion radiotherapy (CIRT), and implemented RBE models in the TPS and MC code were validated.

**Materials and Methods:** For physical commissioning, required beam data were measured at Yamagata University Hospital. Regarding biological calculation, a human submandibular gland (HSG) cell was defined as a type of the microdosimetric kinetic model (MKM) in both TPS and MC code. The physical and biological doses were calculated in water, and the results were compared with each other. In this study, RayStation 10A and the TOol for PArticle Simulation (TOPAS) were used.

**Results:** The measured beam data were successfully implemented in TPS and TOPAS (Figure 1). Figure 2 shows integrated depth dose (IDD) curves which were commissioned to match the measurement data within up to 1% uncertainty for the Bragg Peak range. For biological dose calculation, physics models used in our MC simulation were successfully validated by comparing them with previous literature data.

**Conclusions:** In this study, the TPS and MC code were validated by comparing with measurement data of CIRT. Our results showed well in agreement. Detailed quantitative figures will be presented at PTCOG59 conference.

## P022 -

### Investigating the delivery efficiency and accuracy of Spot-Scanning Proton Arc (SPArc) in comparison with conventional IMPT, automatic step-and-shoot multifield IMPT

#### Lewei Zhao^1^, Gang Liu^1^, Di Yan^1^, Xiaoqiang Li^1^, Xuanfeng Ding^1^

##### ^1^Beaumont Health System, Radiation Oncology, Royal Oak, USA

**Purpose:** To quantitatively investigate the treatment delivery efficiency improvement using spot-scanning proton arc (SPArc) in comparison with automated step and shoot multi-field IMPT (IMPTauto) treatment delivery and clinical IMPT treatment .

**Methods:**Total twelve cases from the three disease sites such as head neck, lung and brain cancer were retrospectively selected. SPArc and IMPTauto plans were generated with the same plan objectives and robustness parameters like clinical IMPT plans. A dynamic arc controller is designed to connect the velocity between control points or beam angles during SPArc and IMPTauto treatment delivery with the following machine configurations. Total treatment delivery time of each case was compared among SPArc, IMPTauto and clinical treatment log files. 3D gamma index was used to evaluate the treatment delivery accuracy between SPArc and IMPTauto from the simulated dose reconstruction.

**Results:** The SPArc and IMPTauto delivery accuracy (Figure 1) is clinical acceptable with ±1 degree delivery tolerance window. The average 3D gamma index comparison showed 97.85± 2.77% passing rate for SPArc and 99.07+ 0.73% for IMPTauto. The SPArc could reduce the average treatment time to 175+74 s compared to the clinical IMPT (641±428 s) and IMPTauto (595±254s). In comparison with clinical IMPT, SPArc simplified the clinical treatment workflow by eliminating the time for each beam request, preparation, transfer time from OIS to delivery system and manually gantry rotation between each treatment field. In comparison with IMPTauto, SPArc effectively reduced the energy layers and spot number (Table 1).

**Conclusions:** SPArc technique could significantly simplify and improve the proton treatment workflow.

## P023 -

### A novel device and treatment fields to irradiate tumors in mouse legs in high LET regions of proton beams

#### Narayan Sahoo^1^, Ronald Zhu^1^, Matthew kerr^1^, Paul Wisdom^1^, Ramesh Tailor^1^, Gabriel Sawakuchi^1^, Xiaodong Zhang^1^, Lakshmi Mahadevan^2^, Bhanu Venkatesulu^3^, Li Wang^4^, Steven Frank^5^, Sunil Krishnan^6^

##### ^1^UT MD Anderson Cancer Center, Department of Radiation Physics-Pt. Care, Houston, USA ^2^Georgetown University Hospital, Department of Anatomical and Clinical Pathology, Washington DC, USA ^3^Loyola University, Department of Radiation Oncology, Chicago, USA ^4^UT MD Anderson Cancer Center, Department of Radiation Oncology-Research, Houston, USA ^5^UT MD Anderson Cancer Center, Department of Radiation Oncology, Houston, USA ^6^Mayo Clinic Florida, Department of Radiation Oncology, Jacksonville, USA

**Purpose:** Irradiation of thick tumors in high LET proton beams is challenging due to sharp dose gradient in its distal dose fall off region. This work describes a device and treatment fields designed to irradiate tumors in mouse legs in high LET regions of proton beams.

**Material and Methods:** The device (Figure 1) consists of a rectangular water tank with two pieces of a movable acrylic platform attached to its walls. This tank has two measuring tapes on its walls. A semi cylindrical animal holder with an opening on the bottom to allow the mouse leg to be extended out, is placed on the two acrylic platforms. A large half beam blocked field is used to irradiate the tumor in the leg immersed in water with minimal radiation dose to mouse body. Two parallel opposed fields of a 250 MeV passively scattered proton beam are used to create a 6 mm section with a dose distribution with +/- 10% variation in the distal fall off region of the Bragg peak as given in Table 1.

**Results:** It has been possible to irradiate six mice together using the device and fields described above. The sliding platform allows for the placement of the tumors in locations with relatively high LET and uniform dose distribution of parallel opposed fields.

**Conclusion:** We have designed a novel device and proton treatment fields for radiobiology experiments to irradiate tumors in mouse legs in high LET regions of proton beams with dose variations within 10% in the irradiated region.

## P024 -

### Commissioning of pencil beam dose calculation algorithm of RayStation v8BSP1 for the MedAustron scanned carbon ion beam delivery system

#### Antonio Giuseppe Amico^1^, Mansure Schafasand^1^, Michaela Daniel^1^, Giovanna Martino^1^, Ralf Dreindl^1^, Loic Grevillot^1^, Alessio Elia^1^, Jhonnatan Moreno Osorio^1^, Marta Bolsa^1^, Stanislav Vatnitskiy^1^, Markus Stock^1^, Antonio Carlino^1^

##### ^1^MedAustron Ion Therapy Centre, Medical Physics, Wiener Neustadt, Austria

In this work we report the results of dosimetric commissioning of the carbon pencil beam algorithm (PBv3.0) available in the TPS RayStation v8BSP1 (RaySearch Laboratories, Sweden) for the scanned carbon ion beam delivery system installed at the fixed vertical beam line (VBL) at MedAustron. The validation of PBv3.0 algorithm was done stepwise by comparing TPS calculated lateral spot profiles in air, integrated radial dose profiles as function of depth in water and absorbed dose to water values in reference conditions with the measured data. In a second step 3D dose distributions with increasing complexity from box-shape to clinical cases were included. Measurements were performed at different air gaps between the nozzle and the in-room isocenter including cases with range shifter (RaShi). The TPS predicted physical range (R_80%_) in water and the measured one are in agreement within ±0.2mm at isocenter. For lateral spot profiles in air the computed FWHMs were within 0.2mm or 2.0% of the measured one at isocenter. Larger deviations within ±5.0% were found at a reduced air gap. Measured and computed absorbed doses to water in reference conditions agree within ±0.1% (Figure1). The results for box-shaped dose distributions in water at isocenter show an average global dose deviation within ±1.0% for isocentric and non-isocentric setups that increase to ±3.0% for non-isocentric setups with RaShi. Regarding the clinical cases, for all 6 different cases the mean delivered global dose deviation was 0.1 ± 0.4% for beams without RaShi and -1.6 ± 0.9% with RaShi (Figure 2).

## P025 -

### Proton and carbon range verification for anatomy-like objects with the use of animal tissue samples

#### Joanna GORA^1^, Marta Bolsa-Ferruz^1^, Stanislav Vatnitsky^1^, Gabriele Kragl^2^, Antonio Carlino^1^, Alessio Elia^1^, Markus Stock^1^

##### ^1^MedAustron Ion Therapy Center, Medical Department, Wiener Neustadt, Austria ^2^Ordensklinikum Linz GmbH- Barmherzige Schwestern, Strahlentherapie, Linz, Austria

Proton and carbon dose calculation in the treatment planning system(TPS) is based on the relationship between CT numbers(HU) and the relative stopping powers(RSP). Previously^*)^, validation of RSP for various animal tissues in the proton field has been performed. As a next step of HU-to-RSP conversion validation, the irradiation of a box-like plan in presence of different tissue interfaces was performed and dose calculation accuracy with proton Monte Carlo (MCv4.2) and carbon pencil beam (PBv3.0) was evaluated. Three different tissue samples(fig.1), were placed in the PMMA phantom, scanned in the CT scanner, acquired images were imported into the RayStationV8bBSP1 (RaySearch Laboratories) and 3d dose distribution for 4x4x4cm^3^ targets were generated for protons and carbons, with and without range shifter (Rashi). Subsequently, the phantom was attached to the MP3 water phantom, each plan was irradiated and measured with a set of 24 pinpoint chambers (PTW, Freiburg), in multiple positions (1-3mm spacing). The differences between measured and computed ranges were evaluated. Independently on the algorithm, particle type and anatomy setup, ranges at R_80_ were within 1mm(Fig2). Larger deviations were observed in the fragmentation tail for the carbon plans. With the increased anatomy complexity (air/bone/tissue interfaces), especially for carbon Rashi beam, the PB algorithm struggled to model the tail of dose distribution, which was not the case for the proton MC algorithm, or in general for homogeneous anatomy. This deficiency of PB algorithm in calculating the dose behind the target (even 8mm) needs to be taken into the account in the treatment planning process.

## P026 -

### Measurement of dose reduction due to markers for RGPT with using Gafchromic film on carbon ion therapy

#### Noriaki Hamatani^1^, Toshiro Tsubouchi^1^, Masaaki Takashina^1^, Masashi Yagi^2^, Tatsuaki Kanai^1^

##### ^1^Osaka Heavy Ion Therapy Center, Radiation physics, Osaka-shi, Japan ^2^Osaka University Graduate School of Medicine, Department of Carbon Ion Radiotherapy, Suita-shi, Japan

**Background**: In our facility, a Real-time Image Gating Particle Therapy (RGPT) system is installed on treatment room 1 and 2. To use this system, some kind of marker that the system can trace with X-ray imagry would be indispensable. However, the combination of RGPT and carbon ion therapy is the first time. Therefore, as commissioning of RGPT system, the effect of markers was confirmed before the start of treatment.

**Purpose**: To confirm the dose reduction ratio due to markers.

**Material and Method**: Eight kinds of markers were used to confirm dose reduction ratios at their downstream. The simple irradiation pattern was made, 10 × 10 × 8 cm SOBP. It was flat with physical dose and the maximum range was 20 cm. And acrylic phantoms were used as the solid phantom. Markers were set to the proximal region of SOBP, and Gafchromic films (EBT3) were set to just after markers, 2.0, 4.0, 5.0 cm and so on behind with the acrylic thickness. After scan of films with scanner, the net optical density (netOD) was calculated using red channel. As the reference netOD of the region without markers, dose reduction ratios were calculated.

**Results and Conclusion**: The reduction ratio became largest with the gold marker (gold sphere type). And the reduction ratio became large as the residual range was close to the distal end region. Gold anchors and VISICOIL with small radius did not so much effect on dose distribution. About other markers, it has to be taken care of the detention places.

## P027 -

### Updated Status of East Japan Heavy Ion Center, Faculty of Medicine, Yamagata University

#### Takeo Iwai^1^, Hikaru Souda^1^, Takayuki Kanai^1^, Yuya Miyasaka^1^, Sung Hyun Lee^1^, Katsumata Masashi^2^, Hiraku Sato^1^, Shinya Sato^3^, Yoshiyuki Ueno^4^, Kenji Nemoto^1^

##### ^1^Yamagata University, East Japan Heavy Ion Center- Faculty of Medicine, Yamagata, Japan ^2^Accelerator Engineering Corporation, Yamagata Office, Yamagata, Japan ^3^Yamagata University, Hosipital, Yamagata, Japan ^4^Yamagata University, Faculty of Medicine, Yamagata, Japan

A carbon-ion therapy project of the Faculty of Medicine, Yamagata University called “East Japan Heavy Ion Center” will be under operation soon. It has two treatment rooms: a fixed horizontal room and a 360-degree rotating gantry room. The horizontal port is used for treatment of prostate cancer with 12 fractions. The rotating gantry is still under preparation, and it will start treatment of other cancer types in the summer of 2021. The main accelerator is a carbon-dedicated compact synchrotron based on the design of Gunma University Heavy Ion Medical Center. The energy range is 55.6 to 430 MeV/u. Beam delivery system is full energy scanning of 600 steps with interval of 0.5 mm, without any physical range shifter. A rotating gantry with superconducting magnets is even smaller than NIRS gantry thanks to the new short-length scanning delivery system developed by NIRS and TOSHIBA Energy Systems. Clinical commissioning including installation of the beam data of integral depth dose and lateral beam profile to treatment planning system (RayStation^TM^ 10.0) was successfully completed in February 2021. Modified microdosimetric kinetic model was applied as RBE model of the TPS. After the fixed horizontal room is open for treatment of prostate cancer on Feb 25, 2021, acceptance test and clinical commissioning of the rotating gantry have been ongoing in almost every evening. Updated status of our facility will be presented.

## P028 -

### Current development status of iBNCT device, the demonstrator of a linac-based neutron source for BNCT

#### Hiroaki Kumada^1^, Yinuo Li^1^, Kenta Takada^2^, Susumu Tanaka^1^, Yoshitaka Matsumoto^1^, Fujio Naito^3^, Toshikazu Kurihara^3^, Takashi Sugimura^3^, Masaharu Sato^3^, Kei Nakai^1^, Akira Matsumura^1^, Hideyuki Sakurai^1^, Takeji Sakae^1^

##### ^1^University of Tsukuba, Faculty of Medicine, Tsukuba, Japan ^2^Gunma Prefectural College of Health Sciences, Department of Radiological Technology, Maebashi, Japan ^3^High Energy Accelerator Research Organization, Accelerator Laboratory, Tsukuba, Japan

The iBNCT project, which consists of industry-academia-government collaboration teams such as the University of Tsukuba and KEK, is being developed accelerator-based neutron source applicable to boron neutron capture therapy (BNCT). iBNCT001 as the demonstration device of an accelerator-based BNCT device has adopted a linac consisted of an RFQ and a DTL and has been combined with beryllium as neutron target material. The linac has designed to accelerate protons of 5 mA or more to 8 MeV. At present, the iBNCT001 has succeeded to drive with an average proton current of 2.1 mA. To confirm the applicability of the iBNCT001 to BNCT, we are conducting several characteristic measurements. In the experiments with a water phantom, distributions for thermal neutron flux and gamma-ray dose, which are the fundamental elements of BNCT dosimetry, were measured. When the device emitted epithermal neutrons while operating the linac with an average current of 2.1 mA, the maximum thermal neutron flux in a water phantom was approximately 1.4 × 10^9^ (n/cm^2^/s) at 2 cm in depth. The neutron intensity has sufficient performance to complete irradiation within 30 minutes with BNCT for head and neck cancer and malignant brain tumors. To confirm the safety and the practicability in the irradiation with actual patients, we have also evaluated radiation exposure outside of the irradiation field by the irradiation with a whole-body phantom. Based on the results for the characteristic measurement, we have planned to implement non-clinical studies with the irradiations for cells and mice with the iBNCT001.

## P029 -

### FAST-01: A medical physics review of the first in-human clinical trial for proton FLASH radiotherapy

#### Anthony Mascia^1,2^, Yongbin Zhang^1,2^, Zhiyan Xiao^1,2^, Eunsin Lee^1,2^, Joseph Speth^1,2^, Mathieu Sertorio^2^, Eric Abel^3^, Michael Folkerts^3^, Ana Lourenco^4^, Nigel Lee^5^, Russell Thomas^5^, Richard Amos^4^, John Perentesis^2^, John Breneman^1^

##### ^1^University of Cincinnati, Department of Radiation Oncology, Cincinnati, USA ^2^Cincinnati Children's Hospital, Cancer and Blood Disease Institute, Cincinnati, USA ^3^Varian Medical Systems, Varian Medical Systems, Palo Alto, USA ^4^University College London, Medical Physics, London, United Kingdom ^5^National Physics Laboratory, National Physics Laboratory, London, United Kingdom

**Introduction and Methods:** Ultra-high dose rate radiotherapy, or FLASH radiotherapy (FRT), delivers therapeutic doses at dose rates exceeding 40Gy/s. With small modifications, some modern proton therapy systems are capable of treating deep-seated tumors with FRT. FAST-01, the first in-human proton therapy FLASH clinical trial, delivers pre-defined FLASH treatment plans using a 250MeV transmission beam at a minimum of 40Gy/s. Commissioning and characterization of the detectors and delivery system was performed, and a quality assurance program and clinical workflow was established. FAST-01 began accruing patients in late 2020.

**Results:** Radiochromic film was calibrated for use in a FLASH environment, and parallel plate ion chambers were validated against a graphite calorimeter, with agreement within 1.0%. The treatment planning beam model was created and transmission-field dose calculations were validated for quality assurance of the pre-defined FRT library. Measurement and calculations agree generally within 2.0%. Quality assurance baselines and trends show absolute dose variation within 1% and dose rate variation within 5%. Safety and workflow testing, such as end-to-end tests and shielding evaluations, demonstrated a safe and efficient FRT environment. Patient QA results meet a 90% passing rate at gamma criteria of 3%/3mm. In vivo dosimetry and log file analysis tools were used as independent monitors and checks following FRT treatment delivery.

**Conclusion:** By adopting an accepted methodology for commissioning while incorporating additional considerations for dose and dose rate delivery, the proton therapy system was technically readied, in a safe and effective manner, for the first in-human proton FLASH clinical trial.

## P030 -

### Feasibility and first year operation of single-room non-hospital-based Quironsalud-Madrid protontherapy center: Workflow, state of the art and research are possible

#### Alejandro Mazal^1^, Juan Castro^1^, Juan Antonio Vera^1^, Juan Maria Perez^1^, Isabel Lorenzo^1^, Enrique Pascual^1^, Elisabet Casas^1^, Lina Mayorga^2^, Amaia Ilundain^2^, Carme Ares^2^, Marta Cremades^3^, Raymond Miralbell^2^

##### ^1^Centro de Protonterapia Quironsalud, Medical Physics, Pozuelo- Madrid, Spain ^2^Centro de Protonterapia Quironsalud, Radiotherapy, Pozuelo- Madrid, Spain ^3^Centro de Protonterapia Quironsalud, Direction, Pozuelo- Madrid, Spain

Our center is based on a Proteus-One-IBA-230MeV synchrocyclotron (“open” arm, robotic positioning, oblique-X-rays and ConeBeamCT), a GE-Revolution dual energy CT (with spectral imaging, artefact correction, and extended Hounsfield), a RaySearch treatment planning system, an Elekta-Mosaiq Oncological Information System, IBA-Dosimetry detectors (scintillators, planes and multilayers ionisation chambers, special chambers), and positioning and organ-movement management by VisionRT surface-based and DynR spirometer systems. The clinical staff is 17 full-equivalent-times evolving from a ratio clinical-staff:patients of 6:1 to better than 10:1 after ramp-up. Thirty-four months were required between contract signature and first patient, 10 months between rigging and acceptance, including 6 months training abroad and 3 months between acceptance and 1^st^ patient (including a calorimetric calibration and international inter-comparisons and audits with CAL-Nice-France, NPL-UK and IROC-USA). As a pioneer facility, we are progressively opening the financial support from the public system (17 autonomic regions), insurances (more than 10) and private circuits, including international patients following national recommendations.

In the first year of operation, starting in December 2019 and regardless of the Covid-19 pandemia, we have succeeded to accomplish our initial goal of treating the first hundred patients. 60% of them are paediatric patients (40% with general anaesthesia). A backup circuit with preplanning has been set with 2 close hospitals with different linacs, TPS and OIS, keeping a single immobilisation system. We have treated complex targets as cranio-spinal, headandneck and base-of-skull, moving organs, and regions with different metallic implants (Ti, Co-Cr, Pt). In addition, we have expanded our indications to hypo-fractionation schemes.

## P031 -

### New transport and dosimetry beamline for Eye Proton Therapy

#### Giuliana Milluzzo^1^, Roberto Catalano^2^, Giada Petringa^2^, Cirrone Pablo^2^, Cuttone Giacomo^2^, Piazza Leandro^3^

##### ^1^LNS-INFN, LNS-INFN, Catania, Italy ^2^INFN-LNS, Infn-Lns, Catania, Italy ^3^BEST Company, BEST Cyclotron, Vancouver, Canada

It is well known that Hadron Therapy, based on the use of protons and ions for cancer treatment, shows many physical and biological advantages with respect to the conventional radiotherapy with X- and gamma rays, such as the higher ballistic precision in the radiation release which allows maximizing the damage to the cancer volume while sparing the surrounding healthy tissues. Recently, a collaboration between the INFN-LNS and the BEST Cyclotron company has been established for the development and the commercialization of a new Proton Therapy beamline for the eye treatment with the 70 MeV protons accelerated from a BEST Cyclotron. The beamline component will be designed by the LNS-INFN also providing a complete Monte Carlo Geant4 simulation of the beam transport. The Monte Carlo simulation will serve to choose the beam line element characteristics in terms of material, thickness and shape in order to respect the clinical tolerances of the beam parameters for protontherapy. New solutions are currently under investigation for making the beamline as compact and automatic as possible as for instance for what concern the modulation and the degradation section of the beam line. Moreover, in order to open to the possibility to use the beam line with high-dose rate proton beams (>40 Gy/s in the so-called flash regime) the implementation of an innovative ionization monitor chamber for the relative dosimetry along the beam line which would allow correcting for the ion recombination effect due to the high-dose rate, is currently under discussion.

## P032 -

### First 18 months experience operating a network of single-room proton therapy centres across the UK

#### John Pettingell^1^, Kate Evans^2^

##### ^1^Rutherford Cancer Centres, Physics & Radiotherapy, London, United Kingdom ^2^Rutherford Cancer Centres, Radiotherapy, South Wales, United Kingdom

Our company built, commissioned and now operates a network of proton therapy centres across the UK. The first three centres started proton treatments in April 2018, June 2019, August 2019, and a fourth centre is under construction. Each centre is equipped with IBA Proteus ONE single-room PBT, as well as linac, CT and MR scanners. All sites are on the same IT network with centralised OMS server (Elekta Mosaiq) and centralised TPS (RayStation and Pinnacle). Beam data from our PBT machines match well enough (J.Lambert et al PTCOG58) that we can transfer patients between sites for a proportion of treatment fractions without the need to re-plan, and without data transfer since all sites use the same Mosaiq server. Previously we presented our first year's experience of running a network of three proton therapy centres (J.Pettingell et al PTCOG2020) including transferring patients between sites. During the first year:

- 44% patients experienced disruption due to downtime
- 3% only had their first fraction delayed
- 41% had disruption after starting treatment
- 0% (no patients) chose (backup) linac treatment at their 'home' centre
- 29% patients chose to travel to another one of our centres for proton therapy the same day or continuing next day
- 12% chose to miss one scheduled treatment fraction (which was made up later)
- No patients had their overall treatment duration increased by more than one day.
- Only 0.12% of the time were all 3 sites/machines down at the same time.

This data will be updated to include the first 18 months.

## P033 -

### Current status of the accelerator-based Boron Neutron Capture Therapy facility at the Helsinki University Hospital

#### Liisa Porra^1^, Tiina Seppälä^1^, Lauri Wendland^1^, Heikki Joensuu^1^, Leila Vaalavirta^1^, Paul Eide^2^, Hanna Koivunoro^2^, William Park^2^, Noah Smick^2^, Ted Smick^2^, Mikko Tenhunen^1^

##### ^1^Helsinki University Hospital, Comprehensive Cancer Center, Helsinki, Finland ^2^Neutron Therapeutics Inc., Neutron Therapeutics Inc., Danvers, USA

Boron Neutron Capture Therapy (BNCT) is a biologically targeted radiotherapy method. Helsinki University Hospital will start BNCT treatments in near future using a compact accelerator-based neutron source, which can be installed in a hospital environment. The safety and efficacy of the L-boronophenylalanine-fructose (BPA-F)-mediated BNCT, have previously been evaluated in the clinical trials in patients with head and neck carcinomas or malignant glioma at the Finnish research reactor FiR 1[1]. Commissioning of the accelerator-based BNCT facility manufactured by Neutron Therapeutics Inc. started mid 2018. The 2.6MV electrostatic proton accelerator is designed to operate at 30mA, and the neutrons are produced by a rotating lithium target. The Radiation Safety Authority of Finland has measured the radiation levels during and after the beam operation and have approved the usage of the facility for commissioning. Neutron beam characteristics in air, the beam profiles and depth dose curves in water and PMMA phantoms have been measured using neutron activation analysis and ionization chambers. The results are consistent with the design goals that also fulfill the recommendations of the IAEA TECDOC-1223[2]. Due to the low residual radiation, the personnel is allowed to enter in the room shortly after the termination of the irradiation. After the commissioning of the neutron beam and the patient positioning system, and approval by the authorities, the first clinical trial will be initiated on patients within inoperable locally recurrent head and neck cancer.

## P034 -

### A new proton beam research facility for radiobiology, animal and medical physics experiments

#### Marta Rovituso^1^, Atia Ibrahimi^2^, Henar Rituerto Prieto^2^, Ersnt van der Wal^3^, Marjan Dwarswaard^4^, Marco Lavagno^5^, Mattia Fontana^5^, Mischa Hoogeman^4^, Danny Lathouwers^6^, Uli Weber^7^, Yuri Simeonov^8^, Ellen Schenk^9^, Marco van Vulpen^10^

##### ^1^HollandPTC, R&D, Delft, Netherlands ^2^TUDelft, Biomedical engineer, Delft, Netherlands ^3^TUDelft, Demo, Delft, Netherlands ^4^HollandPTC, Medical physics, Delft, Netherlands ^5^DE.TEC.TOR, Research and development, Turin, Italy ^6^TUDelft, Radiation Science and Technology, Delft, Netherlands ^7^GSI, Biophysics, Darmstadt, Germany ^8^University of Applied Sciense, Institute of Medical physics and radiation protection, Giessen, Germany ^9^HollandPTC, Research and development, Delft, Netherlands ^10^HollandPTC, Physician, Delft, Netherlands

HollandPTC is the proton therapy centre of Delft, Netherlands, that was born as consortium of TU Delft, Leiden University Medical Centre and Erasmus Medical Centre. Starting to treat patients in 2018, this facility provides a unique opportunity to introduce technological innovations. A Varian cyclotron serves two rotating gantries, an eye beam line and an experimental room. In this frame, the R&D proton beam line is supported by three laboratories for biology, physics and chemistry activities and a short staying animal facility. Inside a well-constructed research program, the scientists of the consortium can perform radiobiological and advanced technology experiments. For this purpose, the experimental room is equipped with different type of detectors to measure the beam characteristics even during irradiation, an innovative concept of removable and modular target station has been customised and built. Motorised target supports of different type, camera and laser system defining two room isocenters are available. In this work, we present the data of the single pencil beam characterisation. Measurements of particle fluence, beam spot size, beam positioning, energy and range have been performed. Moreover, a passive beam line has been designed for radiobiology research in order to have different field size and a spread out Bragg peak. The field characterisation in terms of lateral and depth-dose profile will be shown. A specific work is conducted to measure high dose rate in order to perform FLASH experiment. In this context, data on FLASH dose rate will be presented.

## P035 -

### Status and evolution of the TOP-IMPLART Project

#### Monia Vadrucci^1^, Alessandro Ampollini^2^, Giulia Bazzano^2^, Evaristo Cisbani^3^, Cinzia De Angelis^3^, Sara Della Monaca^3^, Francesco Ghio^3^, Valeria Landoni^4^, Paolo Nenzi^2^, Enrico Nichelatti^5^, Luigi Picardi^2^, Massimo Piccinini^6^, Ronsivalle Concetta^2^, Fabio Santavenere^3^, Vincenzo Surrenti^2^

##### ^1^Enea, Particle Accelerator for Medical Application Laboratory- Application of Radiations Technical Unit- ENEA- Frascati r.c.- Via E. Fermi 45- 00044- Frascati- Rome- Italy, Frascati RM- Italy, Italy ^2^ENEA, Particle Accelerator for Medical Application Laboratory- Application of Radiations Technical Unit- ENEA- Frascati r.c.- Via E. Fermi 45- 00044- Frascati- Rome- Italy, Frascati, Italy ^3^ISS, Istituto Superiore di Sanità ISS- Viale Regina Elena 299- 00161 Rome- Italy, Rome, Italy ^4^IFO, Istituto Nazionale Tumori Regina Elena- IFO- via Elio Chianesi- 53- 00144- Rome- Italy, Rome, Italy ^5^ENEA, Photonics Micro and Nanostructures Laboratory- Application of Radiations Technical Unit- ENEA- Casaccia r.c.- Via Anguillarese 301- 00123 Rome- Italy, Rome, Italy ^6^ENEA, Photonics Micro and Nanostructures Laboratory- Application of Radiations Technical Unit- ENEA- Frascati r.c.- Via E. Fermi 45- 00044- Frascati RM- Italy, Frascati, Italy

TOP IMPLART (Terapia Oncologica con Protoni-Intensity Modulated Proton Linear Accelerator for RadioTherapy) is a proton linear accelerator dedicated to cancer therapy. The project, funded by the Innovation Department of Regione Lazio (Italy) is led by ENEA in collaboration with the Italian Institute of Health (ISS) and the Oncological Hospital Regina Elena-IFO. The accelerator up to 150 MeV, under construction and test at ENEA-Frascati laboratories, employs a commercial 425 MHz 7 MeV injector followed by a sequence of 3 GHz accelerating modules consisting of SCDTL (Side Coupled Drift Tube Linac) structures up to 71 MeV and CCL structures for higher energies, grouped in 4 modules - sections each one powered by a 10 MW klystron. The temporal beam structure consists of a sequence of 3 μsec long pulses at a maximum repetition frequency of 50 Hz. Currently the segment consisting of the first six accelerating modules up to 55 MeV is under commissioning, this energy value is very close to those of clinical interest for the treatment of superficial tumours. Measurements aimed to the optimization and characterization of the output beam in terms of reproducibility and stability are in progress.

## P036 -

### Biological validation using tissue samples of three CT scanners commissioned for proton therapy

#### Alberto Viñals^1^, Borja Aguilar^1^, Pablo Cabello^1^, José Miguel Delgado^1^, Juan Diego Azcona Armendáriz^2^

##### ^1^Clínica Universidad de Navarra, Radiation Physics, Madrid, Spain ^2^Clínica Universidad de Navarra, Radiation Physics, Pamplona, Spain

**Purpose/Objective:** To verify the accuracy of a CT stoichiometric calibration for proton therapy by comparing predicted SPR values with measured ones for a variety of biological tissue samples.

**Material/Methods:** Three Siemens CT scanners (Somatom Drive, Emotion and Biograph PET/CT) were calibrated using the stoichiometric method proposed by Schneider et al. in 1996. A series of 12 samples including fat, cortical bone, brain, blood, round, loin, liver, kidney, and skin were packed in plastic boxes of 5 × 5 × 8 cm^3^, and inserted on a water equivalent phantom and scanned in the CT to get the HU. Additionally, the samples packed in the boxes were used to measure the SPR using a proton beam. They were placed on the side of the water tank. An IDD was measured with the sample located between the beam and the tank, and repeated without the sample. The relationships between the R80 metrics for both IDD was used to determine the SPR.

**Results:** Agreement between the SPR determined by calibration and by measurement was found to be similar for the three CT. We present the differences between predicted and measured SPR for the Drive in the following table. For our clinical practice and CT calibration curve introduced in our TPS, we tweaked the table for the HU corresponding to fat to further reduce the uncertainty in this area.

**Conclusion:** Our CT calibration was experimentally validated ensuring the reliability of its determined SPR values for dose calculation in nthe presence of biological tissue inhomogeneities.

## P037 -

### Proton pencil beam scanning (PBS) small field dosimetry and commissioning using IAEA TRS-398 protocol

#### Hossein Dadkhah^1^, Hisham Kamal Sayed Abdalghaffar^1^, Peng Wang^1^

##### ^1^Inova Schar Cancer Institute, Radiation Oncology, Fairfax, USA

**Purpose:** Proton PBS small field dosimetry and patient-specific QA is challenging due to factors including treatment planning system (TPS) model's limitations, detector size, scattering, charged particle disequilibrium, and linear energy transfer (LET) response of some dosimeters, e.g. radiochromic film (RFC). In this work, we commissioned small-sized proton PBS fields delivered with IBA Proteus PLUS PBS proton system for a clinical TPS (Raystation 10A clinical Monte Carlo), following TRS-398.

**Methods:** The variables being considered include field size (FWHM), target's central depth, presence of the range shifter (RS) in the beam path, air gap between the RS and phantom surface, and energy layer plus spot spacing of the PBS field. The FWHM are designed to be larger than the size of the IBA Razor IC (CC01) plus the range of secondary electrons. The dose in the area covering CC01 is uniform in all directions. Dose measured by CC01 is compared to TPS calculation. Gamma analysis between RFC measurements and TPS calculation is performed.

**Results:** Fields measured without RS showed agreement within 0.5% of the TPS values. However those fields with RS showed deviation in the range of 2-6% depending on FWHM, depth, and air gap. The dose under-response of EBT3 RFC is observed as expected due to LET effects. However the passing rate of relative Gamma analysis with 3%/3mm criteria are over 90%.

**Conclusions:** The TPS tested underestimates dose of the fields with RS with 1.3 cm≤ FWHM≤3, on average by 3%. The degree of underestimation should be quantified and accounted for in the planning and QA procedure.

## P038 -

### A low-budget transmission ionization chamber suitable for Flash-irradiations up to 1000 Gy/s

#### S. Gerke^1,2^, A. Denker^1,2^, T. Fanselow^2^, G. Kourkafas^2^, A. Weber^3^, J. Heufelder^3^

##### ^1^Beuth Hochschule für Technik, Fachbereich II, Berlin, Germany ^2^Helmholtz-Zentrum Berlin, Proton Therapy, Berlin, Germany ^3^Charite - Universitätsmedizin Berlin, Berlinprotonen, Berlin, Germany

A low-budget transmission ionization chamber was developed for Flash irradiations. The functionality and characteristic properties of the designed chamber were investigated with a 68 MeV proton beam. The electrodes were made of metal-coated plastic films. The housing is constructed like a shell structure into which the electrodes were inserted with spacers. The support is made from a 3D printer and shielded by an additional Aluminum housing. The outer dimensions of the housing as well as the value for the operating voltage are based on the values of our conventionally used monitor chambers Type 7861 (PTW-Freiburg, Germany). For the tests, read out and voltage supply, a conventional therapy electrometer (Unidos 10001, PTW-Freiburg, Germany) was used. The measurements were carried out under the aspects of leakage current, water equivalent thickness of the chamber, response and dose linearity. The leakage current was less than 0.2 pA. The signal stability over time is about ± 0.2%. Investigations with varying beam diameter and beam position on the chamber showed variations of up to 4.7%. The dose linearity was the same as the dose linearity of an Advanced Markus chamber (34045, PTW-Freiburg, Germany). Using a chamber voltage of 300 V a 99% saturation will be observed at a dose rate of about 380 Gy/s.

## P039 -

### Real-time measurements to evaluate delivered uncertainty per burst in a pulsed proton pencil-beam-scanning system

#### Wen Chien Hsi^1^, Brett Nelson^2^

##### ^1^University Florida Health Proton Therapy Institute, Assistant Professor- Clinical Medical Physicist, Jacksonville, USA ^2^Logos Systems Int'l, Logos Systems Int'l, Scotts Valley, USA

Each energy-layer of modulated pencil-beam-scanning (PBS) fields was delivered over 3 bursts in our single-room Proteus®One pulsed proton beam system. The dose delivery per burst in this pulsed system can result in a larger uncertainty in comparison with the macro-scale continuous delivery of our Proteus®Plus system. Therefore, 2-dimensional distributions of each burst and each energy-layer were measured in real-time to compare with the planned spot-pattern calculated in the treatment planning system. The commissioning of our pulsed system was expedited by using the data acquired during the validation and verification procedure performed during the tuning of the proton beam optics. The quality-control (QC) program, including the routine machine and patient-specific quality-assurance, was established using various dosimeters. An XRV-124 optical metrology phantom by Logos Systems provided a tool for measuring the coincidence between isocenters of proton-beam and patient positioning in our QC program. The XRV-124 with a capacity of 30 image samples per second was further utilized to measure the 2D distribution of each burst delivery. This phantom and software allowed reconstructing the actual delivered energy-layers (Fig. 1) according to the planned spot-pattern for a uniform cubic dose target 4.0 cm on a side. Because the XRV measured the planar transmissive fluence at <5mm thickness, the normalized gray-scale of mean values over the reconstructed 2D distribution for each energy-layer agrees within 2% to either normalized MU or dose-in-air over 10 planned layers (Fig. 2A). Two XRV-124 measurement techniques with different layer Region of Interest sizes agree well with each other (Fig. 2B).

## P040 -

### Method of Proton FLASH Dosimetry with Advanced Markus Ion Chamber is Verified Using Monte Carlo Simulation of Custom-Made Beamline Components

#### Benjamin Insley^1^, Kiminori Iga^1^, Uwe Titt^1^

##### ^1^MD Anderson Cancer Center, Radiation Physics, Houston, USA

Ion chambers remain the most convenient dosimeter for repeated experiments and quantitative analysis. Markus chambers can achieve acceptable accuracy for dose rates up to 375 Gy/s, but FLASH proton experiments may require rates much larger. To address this, we introduce a custom-made tungsten scatterer into our beamline. TOPAS was used to evaluate its ability to reproducibly reduce the energy deposition to a Markus chamber in a water phantom for various scatterer placements and thicknesses. The beamline is displayed in figure 1. The scatterer was confined to 3.5-cm beyond the reference monitor to avoid interfering with additional beam-modulating components needed elsewhere. For each location and thickness, a correction factor at 3 cm depth (well within the plateau region of the Bragg curve) was calculated as E_dep(No Scatterer)/E_dep(Scatterer). Figure 2a displays correction factors for a 0.538-mm-thick scatterer at distances between 36.2 and 39.7-cm from the phantom surface in intervals of 5-mm. 2b gives the corrections for the scatterer at 39.7 cm using thicknesses between 0.538 and 2.69-mm in intervals of 0.538-mm. Misaligning the chamber by one chamber radius resulted in an average deposition error of 0.6%. This error jumped to 6.6% for 3 radii. Given a 1.614-mm scatterer placed 39.7-cm from the phantom surface, the 31-fold energy deposition reduction would allow measurements of dose rates up to 11,625 Gy/s. This provides a basis for future experimental validation using a Markus chamber to measure a given dose at low and high dose rates.

## P041 -

### Method to measure linear energy transfer for proton therapy beam using thin film solar cell coated with scintillating powder

#### S. Jeong^1^, C. Kim^1^, Y.C. Kwon^2^, S.I. Park^2^, D. Shin^1^, Y.K. Lim^1^, J.H. Jeong^1^, H. Kim^1^, W. Cheon^1^, S.B. Lee^1^

##### ^1^National Cancer Center, Proton Therapy Center, Goyang, Korea Republic of ^2^Kyungpook National University, Department of Physics, Daegu, Korea Republic of

**Purpose:** The present study evaluated the feasibility of the amorphous silicon (a-Si) based flexible thin film solar cell coated with scintillating powder for simultaneously measuring linear energy transfer (LET) and dose for proton therapy beam.

**Materials and Methods:** Solar cell coated with scintillating powder (SC-SP) was composed by attaching scintillating powder to the thin film solar cell using an optical adhesive. Quenching factor of SC-SP was calculated by comparing relative signals by measuring pristine Bragg peak of 145 MeV proton beam using Markus chamber and SC-SP. Proton beam of single Bragg peak with 14.07 g/cm^2^ range was measured with Markus chamber and SC-SP to observe the quenching effect. Then, LET was obtained by inversely using Birks equation and quenching factor. LET measured using SC-SP was compared with the results of Monte Carlo simulation.

**Results:** Quenching correction factor of SC-SP was 0.042 mm/MeV. LETs measured using SC-SP at Bragg peak, - 1.0, - 0.5, and + 0.5 mm of mid-SOBP were 7.289, 2.993, 5.099, and 8.512 MeV/mm, while those calculated by Monte Carlo simulation were 7.264, 2.824, 5.037, and 8.763, respectively.

**Conclusion:** Feasibility of the a-Si flexible thin film solar cell based LET measuring system for proton beam therapy was evaluated by comparing the LETs measured using this system with those calculated by Monte Carlo simulation. The results of this study suggest that this system cost-effectively provides a real-time measurement LET of a therapeutic proton beam.

## P042 -

### Evaluation of neutron ambient dose equivalent in energy scanning carbon-ion radiotherapy

#### Shinnosuke Matsumoto ^1^

##### ^1^National Institute of Radiological Sciences- QST, Department of Accelerator and Medical Physics, Chiba, Japan

**Introductions**: In carbon-ion radiotherapy (CIRT), secondary neutrons occur as the result of interaction between primary beam and beam-line devises or patient's body. The neutron exposure may increase risk for second neoplasm. In scanning-beam CIRT, energy scan(ES), hybrid scan(HS), and range-sifter scan(RS) were used to get depth dose distribution at PTV. Previous research shown neutron ambient dose equivalent (H*(10)) in HS-CIRT and RS-CIRT. On the other hand, It has yet to be shown that H*(10) in ES-CIRT.

**Purpose**: The purpose of this study was to evaluate H*(10) in ES-CIRT so as to develop a understanding of neutron dose in CIRT.

**Materials and Methods**: Using neutron detector WENDI-II and water phantom simulating a patient's body, we measured H*(10) and evaluated distance and angle dependence. And then, we compared H*(10) in ES-CIRT with that of HS- and RS-CIRT.

**Results**: The farther out from water phantom, the lower H*(10) will be. And, The greater the angle of beam axis, the lower H*(10) will be. In this study, we define the distance between phantom and detector as “d” and, the angle of beam axis as “theta”. H*(10) in d=200 was about 10% of that of d=50. And H*(10) in theta=120 was about 42% of that of theta=40. The results of this study also find that H*(10) in ES-CIRT is lower than that of HS- and RS-CIRT.

**Conclusion**: This study evaluated H*(10) in ES-CIRT. The present result suggested that H*(10) can be reduced by using ES-CIRT.

## P043 -

### Predicting proton stopping power ratio for biological tissues using MRI-measured material hydrogen density

#### S. Mossahebi^1^, P. Sabouri^2^, K. Nkenge^1^, B. Yi^1^

##### ^1^University of Maryland School of Medicine, Radiation Oncology, Baltimore, USA ^2^Miami Cancer Institute, Radiation Oncology, Miami, USA

**Purpose**: MRI benefits from exquisite soft-tissue contrasts. However, the need for stopping power ratios (SPR) in proton dose calculation hinders their direct use in dose calculation and requires their fusion and registration with CT. Uncertainties associated with CT-derived proton stopping power ratios (SPR), and image-registration are limiting factors in exploiting the full benefits of MRI in proton therapy. We investigate the feasibility of a model that predicts SPR using MRI-measured material hydrogen densities.

**Methods**: Twenty-two materials taken from the National Institute of Standards and Technology (NIST) database were analyzed and a model (SPR-H) that related the medium's hydrogen density with its SPR was constructed. The SPR-H model was initially validated by using proton-density and T2-weighted MRI scans of twelve salt-water solutions with known salt concentrations (hydrogen densities). For further validation, three tissue samples (two muscle and a muscle-fat mixture) proton-density and T2-weighted MRI scans were used by SPR-H model, and the model-predicted SPRs were compared with measurement using multi-layer ionization chamber (MLIC).

**Results**: MRI pixel values of both scans correlated well with the measured SPRs. For all salt-water solutions and tissues samples, model predicted SPR and WET were within 2% and 2.5% of the measurements.

**Conclusion**: SPR predicted by SPR-H model facilitates dose calculation using MR images while reducing range uncertainty in proton therapy.

## P044 -

### Proton computed tomography algorithm and instrument

#### Paul Mulqueen ^1^

##### ^1^Bragg Peak Systems, Hartselle, AL, USA

We have developed an engineering approach and computer algorithm for proton computed tomography(pCT). The algorithm allows the reconstruction of slices up to 36 cm by 36 cm. The relative stopping power in each voxel can be determined to within 0.0001% of truth. The algorithm allows one cubic mm resolution and 3D reconstruction. As a result, treatment margins can be significantly reduced. The algorithm was tested using simulated tissue slices as per NIST data and simulated proton beams. Each voxel randomly assigned a rsp for air, adipose, water, muscle, bone, or beryllium (simulate metal implant). Further, Gaussian noise is added to each voxel to model clinical variability. The algorithm requires an absorbed dose less than 0.4 Gray. Both beam energy and beam scattering are used to minimize signal to noise. The proposed instrument will rotate in and out of the beam path without needing to be physically attached to either the beam nozzle or patient chair/table. The instrument consists of a robotic aperture, detector, and computer. The aperture avoids the need for a low intensity mode for the beam. The aperture allows only selected protons to exit towards the patient. The selected paths change with time to optimize the image. The detector acquires single proton beam energy and direction information for input into the algorithm.

## P045 -

### In-water fast-time-sensitive measurements with Timepix3 detector for dosimetry and tracking characterization of stray radiation fields produced by FLASH proton beams

#### Cristina Oancea^1^, Lukas Marek^1^, Jiri Pivec^1^, Stepan Polansky^1^, Vladimir Linhart^1^, Carlos Granja^1^, Jan Jakubek^1^, Elisabeth Bodenstein^2^, Jörg Pawelke^2,3^, Jaroslav Solc^4^

##### ^1^ADVACAM, Research, Prague, Czech Republic ^2^OncoRay - National Center for Radiation Research in Oncology- Faculty of Medicine and University Hospital Carl Gustav Carus- Technische Universität Dresden- Helmholtz-Zentrum Dresden-Rossendorf, Research, Dresden, Germany ^3^Helmholtz-Zentrum Dresden-Rossendorf- Institute of Radiooncology - OncoRay, Research, Dresden, Germany ^4^Czech Metrology Institute, Research, Prague, Czech Republic

The purpose of this work is to characterize the stray radiation produced in a water-phantom irradiated with “FLASH” proton beams (PB). The data are valuable to develop traceable and validated dosimetric methods for characterization of ultra-high-pulse-dose-rates (UHPDR) PB. A customized detector based on Timepix3 ASIC chip was immersed inside the water-phantom. For data acquisition, the detector was moved laterally at different depths during irradiation. Using a compact semiconductor pixel detector with electronics placed on a flexible cable at a distance from the sensor (Fig. 1a), we measured the composition, spatial, time, and spectral characteristics of mixed radiation fields. The measurements were performed at the University Proton Therapy Dresden, Germany. Dose rates (DR) exceeding 160 Gy/s were delivered by a pencil PB of 220 MeV energy. Minipix-Timepix3-Flex (Fig. 1b) provides ns-scale timing resolution at the pixel level together with spectral tracking response of individual particles. Results are presented in terms of 3D directional sensitive tracks (Fig. 1b), linear energy transfer spectra with identification of particle type, energy deposition, flux, and DR. In the case of UHPDR PB, the integrated deposited energy (Fig. 2) and DR were measured.

## P046 -

### PRAGUE: the first detector prototype to measure the proton beam range

#### Giada Petringa^1,2^, Pablo Cirrone^1,3,4^, Mariacristina Guarrera^1^, Daniele Margarone^2,5^, Salvatore Tudisco^1^

##### ^1^Istituto Nazionale di Fisica Nucleare INFN, Laboratori Nazionali del Sud LNS, Catania, Italy ^2^Eli-beamlines, Institute of Physics FZU, Prague, Czech Republic ^3^Centro Siciliano di Fisica Nucleare e Struttura della Materia, csfnsm, Catania, Italy ^4^Università di Catania, Dipartimento di Fisica e Astronomia Ettore Majorana, Catania, Italy ^5^Centre for Plasma Physics, Queen's University of Belfast, Belfast, United Kingdom

Measuring and verifying the reliability and stability of the dosimetric properties of a radiotherapeutic beam is the most important task of any external-beam radiotherapy quality assurance program. The beam characteristics can be assessed in terms of several different parameters, such as the percentage depth-dose distribution, flatness, symmetry, and absolute dose output. The depth-dose-distribution measure is today performed, adopting commercial systems whose main advantage is the short operational time. The aim of PRAGUE (Proton RAnGe measure Using silicon carbidE) project is to design and construct a detector, based on a new generation of Silicon Carbide (SiC) devices, to measure proton depth dose distributions in real-time and with high spatial resolution (10 μm). The extreme radiation hardness of such devices and the independence of their response with the proton beam energy makes them capable to operate with clinical hadrontherapy beams and laser-driven ion beams, where extremely high dose rates are delivered. The detector will be composed of a stack of new generation, large area SiC devices with an active thickness of 10 μm. A first detector prototype was already designed and tested. The obtained results indicate the SiC detector as a suitable detector for relative dosimetry with charged particles. It showed, in fact, stable and reproducible response and extremely good behaviour in terms of linearity with respect to absorbed dose was found. The negligible dependence of its response against energy and dose-rate and the high radiation hardness, represent advantageous features with respect to other commercial solid-state detectors for ion beams dosimetry.

## P047 -

### Reconstruction of lateral dose distributions with optimized filtering process for fine carbon-ion beams toward ‘carbon-knife'

#### Mutsumi Tashiro^1^, Hikaru Souda^2^, Hiroshi Sakurai^3^

##### ^1^Gunma University, Heavy Ion Medical Center, Maebashi, Japan ^2^Yamagata University, Department of Heavy Particle Medical Science- Graduate School of Medical Science, Yamagata, Japan ^3^Gunma University, Graduate School of Science and Technology- Division of Electronics and Informatics, Kiryu, Japan

Carbon-Knife with fine carbon-ion beams is expected to be an efficacious treatment for intracranial cancerous and/or non-cancerous diseases with mm sizes, because of its physical advantages such as high linear energy transfer (LET) around Bragg peak and consequent dose concentration with sharper lateral penumbra. For application of such fine beams, dosimetry and/or dose distribution determination are quite essential but often difficult even using well calibrated dosimeters, because of the smaller beam size than the detector sensitive area. We proposed a method to estimate lateral dose distributions for fine beams using iterative reconstruction from the measured dose-area-product (DAP) distributions. DAP distributions were measured with a diode dosimeter (sensitive area of 1 mm^2^) for fine carbon-ion beams with the beam size of 1 mm (FWHM) at surface and near the Bragg peak depth. Lateral dose distributions at respective depths were reconstructed from the measured DAP distributions using the proposed iterative reconstruction method. Errors of the reconstructed dose distributions were reduced using low-pass filtering process with optimized cut-off frequencies. The errors of the reconstructed dose distributions were estimated within 3% accuracy on average with respect to the peak value using the filtering process. In the reconstructed dose distributions, high dose rate of ∼90 Gy/s and sharp penumbra of P_80-20_∼0.2 mm were obtained at the Bragg peak and surface depths within the 1 mm beam size, respectively. These results show a potential of fine carbon-ion beams toward the application to ‘carbon knife' treatment and/or biological research for small targets.

## P048 -

### Dosimetric effects of different ocular prosthesis materials for spot scanning proton therapy

#### Pingfang Tsai^1^, Chin-Cheng Chen^1^, Sheng Huang^1^, Haibo Lin^1^, Ackerman Christopher^1^, Minglei Kang^1^

##### ^1^New York Proton Center, Physics, New York, USA

**Purpose:** To present an efficient way of investigating the stopping power ratio and dosimetric effect of PMMA and silicone ocular prosthesis.

**Methods:** A mini water tank consists of four compartments: one filled with water for calibration; one PMMA and two silicone ocular prostheses (FCI Ophthalmics, and Jardon Eye Prosthetics) were placed in each of the other three with the same water level. A proton Spread-Out-Bragg-Peak (SOBP: 3 cm in width and 7 cm in range) beam was created in TPS for delivery. EBT3 film and MatriXXPT were used for measurement at various depths of the SOBP and at the distal fall-off. The RSP was studied by comparing the measurements against the doses from PCS V15.6 and Monte Carlo (MC) (MCsquare and AcurosPT 13.7) models. The materials selected for AcurosPT calculation were based on the closest available RSP.

**Results:** Hounsfield units (HUs) for the ocular prostheses were assigned based on the suitable RSP (PMMA1.154, silicone1.0298) for dose calculation of different algorithms. The measured R90 for silicones of different brands agree within 1mm. The R90 of PCS and MCsquare of all prostheses are within 2mm agreement with measurement in R90. AcurosPT has a discrepancy ≥3mm in all scenarios when compared to PCS.

**Conclusion:** The difference in R90 for different ocular prostheses is within 2mm between PCS and MCsquare with the RSPs used in this study. However, the AcurosPT shows more range discrepancy due to limited material selections.

## P049 -

### Short-lived radioactive 8Li beam for hadrontherapy

#### Marie Vanstalle^1^, Emil Traykov^1^, Nicolas Arbor^1^

##### ^1^Université de Strasbourg- CNRS- IPHC, Department of Subatomic Research, Strasbourg, France

Although charged particle therapy (CPT) for cancer treatment has grown these past years, the use of protons and carbon ions for therapy remains controversial compared to X-ray therapy. While a biological advantage of protons is not clearly demonstrated, therapy using carbon ions is often pointed out for its high cost. Furthermore, the nuclear interactions undergone by carbons inside the patient are responsible for an additional dose delivered after the Bragg peak, which deteriorates the ballistic advantage of CPT. Therefore, a renewed interest for lighter ions with higher biological efficiency than protons has been recently observed. In this context, lithium ions can represent a good compromise between protons and carbons, as they exhibit a higher LET than protons in the Bragg peak and can be accelerated by cyclotrons. The possibility of accelerating radioactive ^8^Li, decaying in 2 α-particles, is particularly interesting. This work aims to assess the interest of the use of ^8^Li ions for therapy. It was calculated that the ^8^Li decay results in an increase of the LET of almost a factor 2 in the Bragg peak compared to stable ^7^Li. This results also in a higher dose deposited in the Bragg peak without an increase of the dose in the plateau region, as shown on Figure 1 (obtained by Geant4 simulations). The feasibility of accelerating facilities delivering ^8^Li will also be discussed.

## P050 -

### A lateral penumbra studio in protontherapy with pencil beam scanning delivery technique

#### Juan Antonio Vera Sánchez^1^, Juan Maria Perez^1^, Juan Castro^1^, Isabel Lorenzo^1^, Elisabeth Casas^1^, Enrique Pascual^1^, Carme Ares^2^, Amaia Ilundain^2^, Lina Mayorga^2^, Marta Cremades^3^, Raymond Miralbell^2^, Alejandro Mazal^1^

##### ^1^Centro de Protonterapia Quironsalud, Medical Physics, Pozuelo- Madrid, Spain ^2^Centro de Protonterapia Quironsalud, Radiotherapy, Pozuelo- Madrid, Spain ^3^Centro de Protonterapia Quironsalud, Direction, Pozuelo- Madrid, Spain

The lateral penumbra of the clinical beams is straightforward related to the maximum gradients that can be achieved in clinical dose distributions. We compare the lateral penumbra obtained with uniformly distributed spot spacing single layers to those obtained by an inverse optimization of a 10x10x10cm3 cube employing the planning system. We quantify the penumbra reduction that can be achieved without and external collimator and we verify the agreement between dose calculations and experimental measurements. The calculations of the RayStation10ATPS (RaySearch) for both, single layers with uniformly spot spacing and optimized cubic dose distributions at different depths with and without Range Shifter are compared. The beam model employed is a clinical valid model of a Proteus One (IBA). Also, transversal dose distributions are measured for every beam employing a RW3 phantom and analyzed with Matlab. For optimized cubic dose distributions, we find lateral penumbra ranging from 8.7 mm for a cube with the isocenter at a depth of 10cm to 9.7mm for a cube with the isocenter at a depth of 25cm. The use of the RS increases the lateral penumbra up to 12.1 mm for a cubic field with the isocenter at a depth of 5cm. In conclusion, inverse optimization may be employed to minimize the lateral penumbra in PBS as demonstrated by the close values found in this work between optimized dose distributions at different depths. However, further reduction of the lateral penumbra should be accomplished via external collimation.

## P051 -

### Faraday cup – revival of a traditional dosimetry device for measurements up to ultra-high dose-rates for proton therapy

#### Carla Winterhalter^1^, Michele Togno^1^, Konrad Pawel Nesteruk^1^, Frank Emert^1^, Serena Psoroulas^1^, Marie Vidal^2^, David Meer^1^, Damien Charles Weber^1^, Antony John Lomax^1^, Sairos Safai^1^

##### ^1^Paul Scherrer Institut, Center for Proton Therapy, Villigen PSI, Switzerland ^2^Centre Antoine Lacassagne, Institut Mediterraneen de Protontherapie, Nice, France

Faraday cups (FC), which “count protons” by measuring the charge of proton beams, have been used for proton dosimetry for over 40 years (Figure 1). Interest has recently been renewed however as they are, by design, dose-rate independent and able to operate up to the ultra-high dose-rates required for FLASH type therapies. In this study, we analyse FC determined currents using varying dose-rates, and investigate best magnetic and electric field settings of the device. First, FC currents have been determined as a function of cyclotron current (red stars in Figure 2a, dose rates up to 1000 Gy/s). The linear relation between FC and cyclotron current (residuals <5%, dotted line Figure 2a) shows that the FC is indeed independent of the applied dose-rate. Next, a magnetic and/or electric field has been applied in the FC, aiming to minimize contributions of secondary electrons. With a magnetic field (240 Gauss, lines in Figure 2b), the FC signal does not depend on the applied voltage (-1000V to +1000V). This indicates that secondary electrons do not influence the measured charge, and that this setting represents 100% efficiency. Without magnetic field and an electric field only, the measured signal is reduced by an energy dependent factor of up to 1.3% (stars in Figure 2b). In conclusion, the FC is a dose-rate independent dosimetry device which will be invaluable for high and ultra-high dose-rate experiments. Best FC efficiency is obtained when using the FC with a magnetic field only or a combination of magnetic and electric fields.

## P052 -

### Preliminary research on dose-area-product calculation for the fixed beam of the Shanghai Advanced Proton Therapy facility

#### L. Zhu^1^, M. Zhang^2^, X. Xiang^3^, X. Wang^3^

##### ^1^Tsinghua University, Institute of Nuclear Energy and New Energy Technology, Beijing, China ^2^Chinese Academy of Science, Shanghai Advanced Research Institute-, Shanghai, China ^3^Tsinghua University, Institute of Nuclear and New Energy Technology, Beijing, China

During the commissioning stage of China's first domestically-made proton radiotherapy facility, the Shanghai Advanced Proton Therapy facility (SAPT), we chose two methods to derive dose-area-product (DAP) for absolute dosimetry. One is to measure the dose in the center of a 10×10 cm^2^ uniform field with a Markus chamber and Farmer chamber. Another is to measure the dose of one single spot with a Bragg peak chamber. DAP at different counts was calculated ranging from 600 to 15000 counts. Calibration factor in terms of absorbed dose to water of Bragg peak chamber, N_D,w._^BPC^ is cross-calibrated with both Markus chamber and Farmer chamber. The deviation between two calibrations is 1.93 %. Mean DAP at 235 MeV is 69.86, 71.07 and 68.18 Gy*mm^2^/MU for three chambers. Each measurement was repeated ten times for analysing Type A uncertainty. DAP percent difference compared with the average value is 1.25%, 3.00% and -1.19% for three chambers respectively.

## P053 -

### Monte Carlo calculated perturbation correction factors for ionization chambers in clinical proton beams using TOPAS/Geant4

#### Kilian-Simon Baumann^1,2,3^, Sina Kaupa^2^, Constantin Bach^2^, Rita Engenhart-Cabillic^1,3^, Klemens Zink^1,2,3^

##### ^1^University Medical Center Giessen-Marburg, Department of Radiotherapy and Radiooncology, Marburg, Germany ^2^University of Applied Sciences, Institute of Medical Physics and Radiation Protection, Giessen, Germany ^3^Marburg Ion-Beam Therapy Center, Marburg Ion-Beam Therapy Center, Marburg, Germany

**Purpose/Objective**: TOPAS/Geant4 was used to calculate perturbation correction factors for different plane-parallel and cylindrical ionization chambers in clinical proton beams.

**Material/Methods**: The absorbed dose-to-water in a reference volume in a water phantom was scored. Subsequently, the absorbed doses in the sensitive volumes of the ionization chambers were scored while different parts of the chamber were absent (e.g. the central electrode, wall, sleeve and stem). By comparing these dose values the different perturbation correction factors were derived. The chambers were positioned in the water phantom following the reference conditions of the upcoming TRS-398 Code of Practice (CoP).

**Results**: In Figure 1 the perturbation correction factors for the cylindrical chamber NE2571 are shown: the factors for the central electrode, stem and sleeve are independent on the proton energy and are not significantly different than unity. The factor for the cavity and displacement is dependent on the initial energy and significantly different than unity. The factor for the wall does not depend on the initial energy and is significantly different than unity. The total perturbation correction factor as the product of all single factors differs from unity by up to 1.7%. In Figure 2 the perturbation correction factors for the plane-parallel PTW Roos chamber are shown. The total perturbation correction factor differs from unity by up to 1.0%.

**Conclusion**: Perturbation correction factors of ionization chambers in clinical proton beams were calculated. In contrast to the assumption of the TRS-398 CoP, perturbation correction factors can be significantly different than unity.

## P054 -

### New features in Geant4 based particle therapy simulation framework

#### Tsukasa Aso^1^, Chihiro Omachi^2^, Toshiyuki Toshito^2^, Shogo Okada^3^, Koichi Murakami^3^, Takashi Sasaki^3^

##### ^1^National Institute of Technology- Toyama College, Electronics and Computer Engineering, Imizu, Japan ^2^Nagoya City West Medical Center, Nagoya Proton Therapy Center, Nagoya, Japan ^3^High Energy Accelerator Research Organization, Computing Research Center, Tsukuba, Japan

The endeavor to develop the Geant4 based particle therapy simulation framework (PTSIM) began in Japan in 2003. Now the PTSIM is used at many facilities for quality assurance, verification of treatment plans, and advanced research. The PTSIM has been continuously updated to include facility requirements and new treatment techniques. It has been adopted as a primary application software for Geant4 tutorial in Japan to facilitate the understanding of Geant4 beginners in medical physics. PTSIM tutorials were also held in Taiwan with the support of Chang Gung University and the medical physics society in Taiwan. Toward precision medicine, the use-case of the simulation was extended from a simple dose calculation to various research such as development of imaging devices for non-invasive dose monitoring and the study of track structure for calculating biological dose. This research requires a flexible geometry description, scoring functions, and efficient simulation performance. In this paper, we summarize the PTSIM features and example applications. Then we discuss the prospects of the interface of PTSIM to MPEXS-h. The MPEXS-h is a GPU-based Monte Carlo simulation code for electromagnetic process and the extension of the hadronic process of protons developed by the MPEXS project. While the PTSIM is currently used for preparing phase space data of the beam delivery system as an input data file of MPEXS-h, it also provides sophisticated interfaces for data exchange and the migration of code from the PTSIM to the MPEXS-h. We report on the status of our development of the new features.

## P055 -

### Repeatibility and reproducibility of microdosimetry with a mini-TEPC

#### Anna Bianchi^1,2,3^, Anna Selva^2^, Paolo Colautti^2^, Giada Petringa^4^, G.A. Pablo Cirrone^4^, Brigitte Reniers^3^, Alessio Parisi^1^, Lara Struelens^1^, Filip Vanhavere^1^, Valeria Conte^2^

##### ^1^Belgian Nuclear Research Centre SCK CEN, Radiation Protection, Mol, Belgium ^2^Laboratori Nazionali di Legnaro - Istituto Nazionale di Fisica Nucleare, Fisica interdisciplinare, Legnaro, Italy ^3^UHasselt, Faculty of Engineering Technology- Centre for Environmental Sciences- Nuclear Technology Center, Diepenbeek, Belgium ^4^Laboratori Nazionali del Sud - Istituto Nazionale di Fisica Nucleare, Fisica interdisciplinare, Catania, Italy

In proton-therapy, a constant RBE of 1.1 is assumed all along the proton penetration depth, despite the biological evidence of an increased RBE at the distal edge where LET augments. This increase becomes significant in the last millimeters of the proton range but advanced TPS based on LET and RBE variations could result in higher therapeutic gain. TPS would therefore benefit from the availability of detectors that enable to validate the implemented simulations. With this purpose, microdosimeters could be useful tools. At the LNL-INFN a miniaturized TEPC was developed. The aim of this work is studying repeatability on short term and reproducibility on measurements of four campaigns at the 62 MeV therapeutic proton beam of CATANA (Catania, Italy) over a period of one year with a sealed mini-TEPC without gas refurbishing. To enter the clinical practice repeatability and reproducibility must be guaranteed. The dose–mean lineal energy was compared to the total dose–mean LET simulated with the Geant4 code, results reported in Figure 1. RBE assessed with microdosimetric measurements, RBE_μ_, is compared with biological measurements performed by other authors in the same radiation field (Figure 2). The Reproducibility is determined as the standard deviation of repeated measurement, all measurements are consistent within one standard deviation.

## P056 -

### Augmented reality application for innovative data access in particle therapy facilities

#### Faiza Bourhaleb^1^, claudia pardi^1^, Mattia Cretoni^1^, Fabrizio Bello^1^

##### ^1^I-See Computing Ltd, I-See Computing Ltd, Turin, Italy

Progress in particle therapy technology requires a more complex workflow, accordingly the amount of data increases too. Challenge lays in making such data accessible, in a fast and simple way, to all the professional figures involved within the process. The main obstacle is in the distribution of data among several workstations dislocated in different dedicated structures. As main player, augmented reality (AR) is supposed to speed up data access without losing consistency. The AR app allows the user to point specific trackers for objects of interest with a dedicated mobile device (tablet). Trackers trigger the app to pop-up precise information related to each object in real time, using the potentiality of a private cloud dedicated and structured for that purpose. The app is protected by different level of privileges and the host is protected by proper authentication. Another important feature is the possibility to experiment the patient positioning using a 3D sample model inside the scene simulating the treatment room. We present a prototype of AR application for particle therapy centres that improves and speeds up the whole work flow, making the access to information easier and more centralized. We are presenting three different levels of access in this AR application: medical doctor, medical physicists and technical engineer. Augmented Reality is the perfect candidate to help healthcare organizations make their existing processes more precise and efficient. Using AR tools, useful information can be provided and related in real-time to the specific need of the different systematic tasks that are daily checked accurately in PT facilities.

## P057 -

### Proton Radiosurgery: Patient Specific Dosimetric PBS QA

#### M. Bussiere^1^, J. Daartz^1^, J. Verburg M.^1^, N. Depauw^1^, T. Ruggieri^1^, J.S. Loeffler^1^, P.H. Chapman^2^, H.A. Shih^1^

##### ^1^Masachusetts General Hospital, Radiation Oncology, Boston, USA ^2^Masachusetts General Hospital, Neurosurgery, Boston, USA

Proton radiosurgery has been an integral part of our clinical programs with over 5,000 cases treated using passive scattering (PS) since 1961. Although pencil beam scanning (PBS) has been available on one of our two gantries since 2010, it has primarily been used to treat large targets which can benefit from the additional beam shaping offered by this modality. A recent upgrade of our second gantry from PS to PBS necessitated the validation of PBS for small fields with FWHM < 4 cm. When centers replace PS with PBS, they are challenged to adapt QA techniques for radiosurgery which combines multiple small collimated fields to deliver doses of 8-22 Gy(RBE) within stringent alignment requirements. Our standard PBS QA uses a 2D ion chamber array with 7.6 mm detector spacing. Measurements are done at a fixed gantry angle. Field-specific output and profiles are measured at one or more depths, while maintaining the detector at isocenter to streamline workflow. Agreement with the treatment planning system is assessed by comparing absolute dose and performing a 3D gamma-analysis with in-house software. We describe a technique which combines a micro-diamond detector to measure absolute dose and calibrated gafchromic film to compare high-resolution cross-sectional dose profiles. This approach has been clinically validated for field diameters from 9 mm with isocenter depths to 17.5 cm. A hybrid 2/3D gamma-analysis pass-criteria requires 90% of points be within 3% of the global maximum dose with 1 mm distance to agreement and 10% threshold.

## P058 -

### Enhancing gamma production for online dose verification in proton therapy

#### Giorgio Cartechini^1^, Luna Pellegri^2^, Michela Maraffini^3^, Franco Camera^4^, Francesco Tommasino^1^, Emanuele Scifoni^5^, Chiara La Tessa^1^

##### ^1^University of Trento, Physics, Trento, Italy ^2^University of the Witwatersrand- South Africa, Physics, Witwatersrand, South Africa ^3^Museo Storico della Fisica e Centro Studi e Ricerche E.Fermi, Physics, Rome, Italy ^4^Università degli Studi di Milano Statale, Physics, Milano, Italy ^5^Trento Institute for Fundamental Physics and Application TIFPA-INFN, Physics, Trento, Italy

The main rationale for using protons in cancer treatment is based on the depth-dose distribution, which translates into a superior sparing of normal tissue compared to conventional radiotherapy. This advantage can be fully exploited only if it is possible to accurately verify the match between the Spread-Out-Bragg-Peak and the tumor position. A mispositioning potentially translates into an under-dosage of the tumor as well as an over-dosage of the normal tissue, which can significantly hinder the treatment efficacy. In this study, we investigated a novel strategy for real time dose verification exploiting prompt gammas (PG). The methodology is based on the detection of PG, whose production is artificially enhanced using a non-radioactive element transported selectively to the tumor with a drug carrier. Nuclear interactions of this element with the beam generate a signature PG spectrum, from which the tumor position can be reconstructed. To identify potential candidate elements, we performed Monte Carlo calculations with Geant4, simulating the interaction of a proton beam at energies relevant to the clinic with different targets: 19F, 45Sc, 63Cu and 89Y. The elements were chosen due to their low abundance in human body and the existence of a compatible drug carrier which guarantees the applicability in clinic. From this study we identified 63Cu and 89Y as promising tumor-label candidates due to the production of high energetic gammas, above the characteristic gamma emission of normal tissue. We also compared the simulations with experimental measurements, and we investigated the minimum element concentration in tumor to obtain a detectable PG enhancement.

## P059 -

### A novel component method to delineate surgical spine implants for proton Monte Carlo dose calculation

#### Chih-Wei Chang ^1^

##### ^1^Emory University, Radiation Oncology, Atlanta, USA

**Purpose:** Spine implants has been shown to decrease local control for spinal sarcoma and chordoma patients because of artifact-induced dose calculation inaccuracies. Artificial intelligence (AI)-based methods have shown promises for metal artifact reduction (MAR) assuming full prior knowledge of implant material maps. We propose a novel component method to delineate implants from CT images that can generate accurate sinogram data to train the AI-based models.

**Methods:** We propose a novel method to identify implant components (tulip, screw and rod), utilizing various levels of prior knowledge: CT-scanned implant components and implant characteristics from medical records. The method was applied to CT images with extended Hounsfield units (HU) and fine resolution. A spine phantom and *in vivo* for one patient were selected to demonstrate the method.

**Results:** It was found that mischaracterization of 8 typical implants in a patient can cause under coverage of a clinical target volume (CTV) around 20 cm^3^, consistent with excessive proton range uncertainties of 10 mm reported in the literature. Using our method, tulip, the most important implant component, can be characterized with proper material and volume as well as other components. Intended CTV coverage can be maintained within tolerance.

**Conclusion:** Material inference using our component method provides accurate implant characterization for proton dosimetry and can potentially enhance the accuracy of AI-based MAR models. The current study primarily focuses on the implant characterization for lumbar spine. Future work include cervical spine and dental implants for head-and-neck patients where tighter margins are required due to proximity of organs-at-risk.

## P060 -

### Acoustic detection of injectable superheated nanodroplet for in situ range verification: Methods for localization of single vaporization events

#### Gonzalo Collado-Lara^1^, Sophie Heymans^2^, Marta Rovituso^3^, Bram Carlier^4^, Yosra Toumia^5^, Martin Verweij^6^, Gaio Paradossi^5^, Edmond Sterpin^4^, Hendrik Vos^1^, Jan D'hooge^7^, Nico de Jong^1^, Koen Van Den Abeele^2^, Verya Deichin^6^

##### ^1^Erasmus MC, Biomedical Engineering- Department of Cardiology, Rotterdam, Netherlands ^2^KU Leuven Kortrijk, Department of Physics, Kortrijk, Belgium ^3^Holland PTC, Research, Delft, Netherlands ^4^KU Leuven, Department of Oncology, Leuven, Belgium ^5^University of Rome Tor Vergata, Department of Chemical Sciences and Technology, Rome, Italy ^6^TU Delft, Imaging Physics, Delft, Netherlands ^7^KU Leuven, Department of Cardiovascular Sciences, Leuven, Belgium

Superheated nanodroplets (100-5000 nanometers), currently investigated in the ultrasound imaging field, can circulate in the vascular system after injection. Recently, it has been shown that the homogeneous nucleation theory developed for bubble chambers and superheated detectors also holds for these small droplets [1]. Charged particles, upon deposition of high energy densities at their individual Bragg peak, can trigger the transition of the droplet to a more stable gaseous phase. Thus, the vaporization distribution of these nanodroplets could be used to monitor the beam range. Here, we have implemented two different methods: active and passive, to localize the forementioned vaporizations using an ultrasound imaging platform. Nanodroplets suspended in a gel matrix were irradiated with a pencil proton beam (Holland PTC, the Netherlands). In the active method, ultrasound snapshots were taken during irradiation. Since the formed gas bubbles have a much better echogenicity than the droplets, only the former appear distinctly in the ultrasound images. In the passive method, the ultrasound system was used to listen to the acoustic waves that the droplets produced during phase change. As the acoustic probe had several sensing elements, the difference in time of arrival was used to locate the vaporizing droplet. The distributions of vaporization events were compared with an independent measurement of the beam with an ionization chamber, showing a submillimeter agreement in both the beam range and the spot sigma. Thus, these methods appear promising for real-time range verification.

## P061 -

### Detection of inter-fraction morphological variations in carbon therapy: results of a clinical trial performed at CNAO

#### Marta Fischetti^1^, Micol De Simoni^2^, Yunsheng Dong^3^, Alessia Embriaco^4^, Veronica Ferrero^5^, Carlo Mancini-Terracciano^2^, Michela Marafini^6^, Riccardo Mirabelli^2^, Matteo Morrocchi^7^, Silvia Muraro^8^, Vincenzo Patera^1^, Elena Solfaroli Camillocci^2^, Marco Toppi^1^, Giacomo Traini^9^, Alessio Sarti^1^

##### ^1^La Sapienza- Università di Roma, Dipartimento di Scienze di Base e Applicate per l'Ingegneria, Rome, Italy ^2^La Sapienza- Università di Roma, Dipartimento di Fisica, Rome, Italy ^3^Università degli studi di Milano, Dipartimento di Fisica, Milan, Italy ^4^INFN, Section of Pavia, Pavia, Italy ^5^INFN, Sezione di Torino, Turin, Italy ^6^Centro Ricerche Enrico Fermi, Centro Ricerche Enrico Fermi, rome, Italy ^7^Università di Pisa, Dipartimento di Fisica, Rome, Italy ^8^INFN, Section of Milan, Milan, Italy ^9^INFN, Section of Rome, Rome, Italy

in particle therapy, morphological variations occurring within the treatment delivery may significantly modify the actual deposed dose with respect to that calculated at planning stage, affecting the treatment efficacy and the enhancing Normal Tissue Complication Probability. The Dose Profiler, developed in the framework of the INSIDE collaboration, is a scintillating fiber-based tracker designed to spot morphological changes in 12C treatments reconstructing the large-angle charged particles produced in the fragmentation of the primary beam. As the fragments yield is correlated to the tissue density, morphological variations are identified comparing the reconstructed 3D spatial distribution in different fractions. The Dose Profiler is currently operating at CNAO (Centro Nazionale di Adroterapia Oncologica, Pavia, Italy) in the context of a clinical trial (ClinicalTrials.gov Identifier: NCT03662373). started in 2019 to evaluate the capability and sensitivity in detecting morphological changes arising in pathologies of the neck-head district. Analysing the first set (10) of patients involving Adenoid Cystic Carcinoma (ACC) , Clival Chordoma and Intestinal-type adenocarcinoma (*ITAC*) pathologies, fragment map differences correlated to morphological variations has been observed in some patients that underwent to a control CT during the treatment. In this contribution the results obtained investigating the all the patient sample will be reviewed and the technique potential in assessing the insurgence of morphological changes in the patient that require a treatment plan re-evaluation will be discussed.

## P062 -

### Real-time positron emission imaging for range verification in helium beam radiotherapy.

#### Peter Dendooven^1,2^, Ikechi Ozoemelam^1^, Emiel van der Graaf^1,2^, Marc-Jan van Goethem^1,2^, Maciej Kapusta^3^, Nan Zhang^3^, Sytze Brandenburg^1,2^

##### ^1^University of Groningen, KVI-Center for Advanced Radiation Technology, Groningen, Netherlands ^2^University Medical Center Groningen, Department of Radiation Oncology, Groningen, Netherlands ^3^Siemens Healthineers, Siemens Medical Solutions USA, Knoxville, USA

Interest in helium beam radiotherapy is driven by advantages over protons and carbon ions: smaller lateral penumbra and larger radiobiological effects than protons; less fragmentation and lower implementation costs than carbon ions. Real-time range verification potentially enables smaller safety margins during treatment planning, reducing side effects and increasing quality of life. Experiments were performed at the PARTREC Facility of the University Medical Center Groningen. We established that nitrogen-12 (half-life 11 ms) is the most produced very short-lived positron emitter in the Bragg peak region of helium-4 and helium-3 beams in water and carbon [1]. Helium-3 produces 3-4 times more nitrogen-12 than helium-4. The positron emitter activity induced by a 90 MeV/u helium-4 beam stopped in PMMA was imaged using a dual panel PET scanner [2]. After a 10 ms pulse containing 6.6×10^7^ helium-4 ions, positron imaging can determine the edge of the nitrogen-12 profile with a precision of 4.1 mm (1 standard deviation). This precision scales with the inverse square root of the number of helium-4 ions, indicating that it is essentially determined by counting statistics. Extrapolation to an optimized imaging situation gives a clinically relevant precision of 0.9 mm (1 standard deviation) when summing data over 10 typical distal layer spots of 4×10^7^ helium-4 ions each. Under the same dose conditions, the precision reached in a proton irradiation is almost 3 times better [3].

## P063 -

### An intuitive tool for detection of mechanical collisions during treatment planning in RayStation

#### F. Hueso-González^1,2^, P. Wohlfahrt^1,3^, D. Craft^1^, K. Remillard^1^, N. Depauw^1^

##### ^1^Massachusetts General Hospital and Harvard Medical School, Department of Radiation Oncology, Boston, USA ^2^Now with IFIC - Instituto de Física Corpuscular, CSIC / Universitat de València, Paterna, Spain ^3^Now with Siemens Healthineers, Cancer Therapy, Forchheim, Germany

In particle therapy treatment planning, it is common to encounter beam incidence directions that are attractive from the dosimetric point of view, but that might pose a risk of collision between the nozzle and the robotic couch or patient. The planner has either to avoid this gantry angle, or to verify it during a dry run in the treatment room. To address this shortcoming, we have implemented a software tool [1] that allows for interactive collision verification within the treatment planning system. The 3D models of the machine and couch, stored in common STL format, are visualized together with the patient surface (Fig. 1) and are translated and rotated to match the angles selected in the active treatment plan or in the interactive sliders (Fig. 2). The risk of collision is calculated in real-time based on the overlap between the volumes of interest. The planner can profit from this feedback to guide the choice of beam angles and prevent collisions at a later stage, which otherwise delay patient treatment and affect the clinical workflow. The proposed software has been implemented and tested for the RayStation planning system, and is available on GitHub with an open-source license.

## P064 -

### Evaluation of Carbon Fiber and Titanium surgical implants for proton therapy.

#### N. Depauw^1^, J. Pursley^1^, S. Lozano-Calderon^2^, C. Patel^1^

##### ^1^Massachusetts General Hospital, Radiation Oncology, Boston, USA ^2^Massachusetts General Hospital, Orthopaedics Surgery, Boston, USA

Carbon fiber (CF) is an attractive alternative to titanium alloy (TA) for orthopedic osseous fixation implants in patients receiving radiation therapy due to its physical properties. We compared the two materials in terms of imaging quality and proton dosimetric properties. We analyzed 5 × 5 × 1.1 cm slabs of TA and CF. CT scans with and without metal artifact reduction (MARS) were acquired of each slab placed in a Lucite holder. The apparent thickness, average Hounsfield units (HU), and corresponding proton relative stopping power ratio (RSP) were evaluated. Actual RSP values were obtained for each slab from range pull back measurements and exact physical thickness evaluation with a caliper. Physical thicknesses were 11.05 +/-0.03 and 11.1 +/-0.1 mm for TA and CF, respectively. Pull back measurements yielded results consistent with RSP of 3.204 and 1.414, respectively. CT-based thicknesses were 12.8 and 10.7 mm for TA before and after MARS. CF measured 10.9 mm both with and without MARS. Titanium resulted in CT HU saturation (3071) which yields a 3.20 RSP value based on our clinical HU-RSP mapping. CF resulted in a mean 364 HU value, consistent with a RSP value of 1.203. Compared to actual RSP values, these correspond to -0.1% and -14.9% errors. MARS improved the accuracy of CT-based thickness assessment of TA; CF CT-based thickness was accurate regardless of MARS. CT-based RSP determination was more accurate for TA than for CF. These results will provide guidance in order to best handle CF and TA implants for proton treatment planning purposes.

## P065 -

### Quantitative and statistical analyses of monthly OBI QA on Varian ProBeams

#### Zachary Diamond^1^, Alex Stanforth^1^, Roelf Slopsema^1^, Katja Langen^1^

##### ^1^Emory Proton Therapy Center, Physics, Atlanta, USA

Routine quality assurance of onboard imaging systems (OBI) for proton therapy is a standard practice performed monthly according to the recommendations given in the American Association of Medical Physics Task Group Report No. 224 (AAPM TG #224). Methods utilized to carry out such tests are nearly identical to those in standard photon radiotherapy (AAPM TG #142) for both planar-kV imaging and CBCT imaging, where all measurements taken in subsequent months are with respect to a pre-determined baseline measurement. This study examines the variability of obtained image quality parameters across four ProBeam treatment rooms and varying imaging techniques. Furthermore, this study examines the effects of possible artifacts and routine imager recalibration specific to ProBeam systems.

## P066 -

### Dosimetric commissioning of an independent dose calculation system for proton therapy

#### Ralf Dreindl^1^, Roser Fayos-Solá Capilla^1,2^, Alessio Elia^1^, Marta Bolsa-Ferruz^1^, Lukas Scheuchenpflug^1,3^, Ruben Gonzalo Gleyzes^1^, Antonio Carlino^1^, Markus Stock^1^, Loïc Grevillot^1^

##### ^1^MedAustron Ion Therapy Center, Medical Physics, Wiener Neustadt, Austria ^2^Hospital Universitario La Princesa, Department of Medical Physics and Radiation Protection, Madrid, Spain ^3^University of Vienna, Technical Physics, Vienna, Austria

**Introduction**: Independent Dose Calculation (IDC) is partly replacing measurement-based patient-specific QA (PSQA) at the MedAustron Ion Therapy Center since February 2021. This work presents the results of the dosimetric commissioning of the myQA iON (IBA Dosimetry) IDC system for a horizontal proton beam line (HBLp).

**Methods**: The agreement between Monte Carlo simulations and the measured baseline-data was checked with respect to beam ranges and beam optics for isocentric/non-isocentric conditions, for 20 energies within the clinical proton energy range 62.4 - 252.7 MeV. The isocenter to detector surface distance (ISD) was increased towards the nozzle, leaving air gaps between 64.8 cm (ISD0) and 6.8cm (ISD58). The influence of range shifter was investigated in addition. The beam model (BM) was calibrated in mono-energy scanned fields (reference conditions) and verified for various 3D targets in water.

**Results**: Simulated ranges were found to agree with the baseline measurements within ±0.2 mm, Bragg-Peak width within ±0.1 mm. Simulated spot sizes were within ±5% of the baseline (Figure 1). Dose in reference conditions was on average -0.3%± 0.5% compared to measurements. Point dose comparisons of 3D targets resulted in an average dose difference between IDC and measurements of +1.8%, independently of the target size, energy used or range shifter. Consequently, a 1.8% scaling factor in terms of number of particles (NP) was applied to the BM (Figure 2).

**Conclusion**: Agreement between simulations and measured baseline data was found to be well within clinical tolerances. The dosimetric commissioning of the HBLp BM was a pre-requisite to start clinical commissioning in patient CT geometry.

## P067 -

### Range verification in proton therapy using oxygen-18 enriched water: in vivo proof of principle

#### Samuel España^1^, Daniel Sánchez-Parcerisa^1^, Paloma Bragado^2^, Alvaro Gutierrez-Uzquiza^2^, Almudena Porras^2^, Carolina Gutiérrez-Neira^3^, Andrea Espinosa^1^, Víctor V Onecha^1^, Paula Ibáñez^1^, Víctor Sánchez-Tembleque^1^, José M Udías^1^, Luis M Fraile^1^

##### ^1^Universidad Complutense de Madrid, Estructura de la Materia- Física Térmica y Electrónica, Madrid, Spain ^2^Universidad Complutense de Madrid, Departamento de Bioquímica y Biología Molecular, Madrid, Spain ^3^Universidad Autonoma de Madrid, Centro de Microanálisis de Materiales, Madrid, Spain

PET imaging has been proposed for in-vivo proton range verification using radiotracers produced in naturally-occurring isotopes. Other isotopes such as 18O, 68Zn or 63Cu, with a high proton-induced reaction cross-section and a low reaction threshold, may be used as possible contrast media for proton PET range verification. However, no in-vivo experiments using such contrast agents have been performed to date. In this study, 18O-enriched water (18-W) was evaluated as a suitable contrast agent on a chicken-embryo chorioallantoic membrane tumor model of head and neck cancer. Several eggs inoculated with tumor cells were infused with 18-W and irradiated with 8-MeV protons. Activation was recorded using a preclinical PET-CT scanner and further evaluated ex-vivo on excised tumors using gamma radiation detectors. The production of 18F and its biological entrapment was determined. PET images showed retained activation of the contrast agent within the tumor up to several hours after irradiation, demonstrating that the produced 18F has minimal biological washout. 18-W appears as an innocuous contrast agent which produces PET activity entrapped inside the tumor and provide proton activation in the last-mm of the proton path. Further steps are required to evaluate its possible clinical implementation.

## P068 -

### Scanning beam visualization in a clinic-like carbon-ion treatment delivery

#### Renato Felix-Bautista^1,2,3^, Laura Marie Helen Ghesquière-Diérickx^1,3,4^, Marcus Winter^3,5^, Tim Gehrke^1,3,5^, Mária Martišíková^1,3^

##### ^1^German Cancer Research Center DKFZ, Medical Physics in Radiation Oncology, Heidelberg, Germany ^2^Heidelberg University, Faculty of Physics and Astronomy, Heidelberg, Germany ^3^National Center for Research in Radiation Oncology NCRO, Heidelberg Institute for Radiation Oncology HIRO, Heidelberg, Germany ^4^Heidelberg University, Medical Faculty, Heidelberg, Germany ^5^Heidelberg University Hospital, Radiation Oncology, Heidelberg, Germany

Pencil beam delivery at synchrotron-based facilities is more prone to uncertainties than at facilities with cyclotrons. The sensitive fine-tuning of elements of the beam delivery system can introduce uncertainties in the control of the lateral positions of carbon-ion beams. The expression of such fluctuations is enhanced by the usually much larger nozzle-to-isocenter distance. New QA strategies capable of providing information on the actual lateral beam position at the isocenter during the irradiation could lead to valuable improvements. An independent non-invasive methodology for lateral beam positions monitoring is presented. This methodology, based on single secondary fragment tracking, is developed and verified using clinic-like carbon-ion treatments with typical clinical doses per fraction of 3 Gy (RBE), using an anthropomorphic head phantom in a treatment position. Treatment deliveries were performed at the HIT facility in Heidelberg, Germany. A mini-tracker based on the Timepix3 technology, developed at CERN, was used to track secondary fragments leaving the irradiated phantom in the beam direction. Secondary-fragment tracks were used to derive the measured lateral pencil beam positions to visualize and quantify the intra-fractional scanning beam movement (Fig.1a). Precision and accuracy of the method, calculated from differences between measured and reference beam positions in each beam energy, were found to be in line to clinical uncertainties of ±1 mm (Fig.1b) over the entire treatment delivery. From the findings, the presented methodology has shown clinically relevant capabilities of lateral beam position monitoring during treatment deliveries and it is ready to be assessed in an observational clinical study in future.

## P069 -

### Development of a PBS dose rate QA tool to support the first in-human FLASH proton therapy trial

#### Michael Folkerts^1^, Sylvie Spiessens^2^, Michiko Rossi^3^, Pierre Lansonneur^3^, Matti Ropo^3^, Viljo Petäjä^3^, Irakli Kalichava^4^, Amin Douiri^4^, Adam Harrington^1^, Vidhya Krishnamurthi^1^, Eric Abel^1^

##### ^1^Varian Medical Systems- Inc, Proton Solutions, Palo Alto, USA ^2^Varian Medical Systems Belgium NV, Proton Solutions, Machelen, Belgium ^3^Varian Medical Systems Finland Oy, Proton Planning, Helsinki, Finland ^4^Varian Medical Systems Particle Therapy GmbH, PT Software Quality Engineering, Troisdorf, Germany

**Purpose:** FLASH therapy is an experimental treatment modality delivering radiotherapy at ultra-high dose rates to treatment volumes in typically <1 second. With some modifications to ProBeam® clinical hardware, FLASH proton therapy using pencil beam scanning (PBS) delivers dose rates (DR) greater than 40 Gy/s. Such hardware is used for the first in-human FLASH proton therapy trial (FAST-01). Due to high beam currents and unique spatiotemporal characteristics of PBS FLASH delivery, direct DR measurement techniques are challenging and time consuming. This work presents a logfile-based PBSDR [1] verification tool developed for the trial.

**Methods:** A 250 MeV single-energy-layer proton transmission plan with a field size of 7.5 × 7.5 cm^2^ was designed. A prototype build of the Eclipse™ treatment planning system (TPS) predicted the 10th percentile PBSDR in the isocenter plane based on modeling the hardware delivery characteristics. The field was delivered 180 times and logfiles were collected. The developed FLASH QA tool reconstructed dose delivery as a function of time based on measurements and data recorded in the logfiles. The tool calculated and reported the 10th percentile PBSDR for each field. The delivered PBSDR was compared to the value predicted by the TPS.

**Results:** Figure 1 shows the distribution of delivered PBSDR. The predicted TPS value of 52.5 Gy/s matched the mean of the delivered values of 52.5 Gy/s (having a standard deviation of 1.5 Gy/s). Figure 2 shows an example screenshot from the tool.

**Conclusion:** We successfully developed an independent FLASH QA tool to verify delivered PBS dose rates for FAST-01.

## P070 -

### Spot characteristic stability for utilizing the XRV-2000 scintillation detector in proton pencil beam scanning

#### Shun Fujiwara^1^, Yuki Tominaga^1,2^, Takayasu Haruna^3,4^, Masataka Oita^1^

##### ^1^Okayama Universitey, Graduate School Of Interdisciplinary Science And Engineering In Health Systems, Okayama, Japan ^2^Medeical Co. Hakuhokai Osaka Proton Therapy Clinic, Radiotherapy, Osaka, Japan ^3^Tsuyama Chuo Hospital, Department of Radiological Technology, Tsuyama, Japan ^4^Okayama Universitey, Graduate School of Health Sciences, Okayama, Japan

**Purpose**: The study aims to evaluate the long-term stabilities for the spot position and beam size of our pencil beam scanning proton therapy system with the XRV-2000 (XRV). We analyzed the 17-month measurements from November 2018 to March 2020.

**Methods**: The 25 beam spot positions were measured continuously for 92 energies (235.0-70.7 MeV) with a simple grid pattern of 160 × 160 mm2 at 40 mm intervals in air. The beam spot was measured only in the center for all energies. The measurement angles consist of 0, 90, 180, and 270 degrees both measurements. The spot positions in the X and Y direction and the spot sizes were analyzed by the Gaussian fitting with the Sample Picture (HIBMS).

**Results**: The average spot position deviations in X-direction were -0.66 ± 0.37 mm for the 0-degree, -0.83 ± 0.80 mm for the 90-degree, 0.04 ± 0.47 mm for the 180-degree, and -0.44 ± 0.59 mm for the 270-degree. The average spot position deviations in Y-direction were 0.06 ± 0.58 mm for the 0-degree, 0.35 ± 0.34 mm for the 90-degree, 0.35 ± 0.63 mm for the 180-degree, and 0.89 ± 0.31 mm for the 270-degree. All spot size deviations were within ± 6.4% for all energies at four angles.

**Conclusions**: The spot size deviations were satisfied our tolerance of ± 10%. However, the spot position deviations were exceeded of -3.39 mm (within ± 2 mm) as it was affected by the rotation of XRV.

## P071 -

### Detection of 2 mm air cavities in a head phantom using secondary-ion tracking during clinic-like carbon-ion irradiations

#### Laura Marie Helene Ghesquiere-Dierickx^1,2,3^, Annika Schechter^1,2,4^, Renato Felix-Bautista^1,2,4^, Laurent Kelleter^1,2^, Gernot Echner^1,2^, Marcus Winter^2,5^, Tim Gehrke^1,2,6^, Mária Martišíková^1,2^

##### ^1^German Cancer Research Center DKFZ, Department of Medical Physics in Radiation Oncology, Heidelberg, Germany ^2^Heidelberg Institute for Radiation Oncology HIRO, National Center for Radiation Research in Oncology NCRO, Heidelberg, Germany ^3^Heidelberg Medical Faculty, University of Heidelberg, Heidelberg, Germany ^4^Faculty of Physics and Astronomy, University of Heidelberg, Heidelberg, Germany ^5^Heidelberg Ion-Beam Therapy Center HIT - Heidelberg University Hospital, Department of Radiation Oncology, Heidelberg, Germany ^6^Heidelberg University Hospital, Department of Radiation Oncology, Heidelberg, Germany

Monitoring of the radiation field within a patient during carbon-ion radiotherapy (CIRT) is of great interest to detect inter-fractional changes and potentially reduce CIRT tumour safety margins. To do so, a carbon-ion beam monitoring method using secondary-ion tracking has been developed in our group and showed promising results. In this contribution, the performance of the method was investigated to detect inter-fractional changes in a head model for different air cavity positions and detection angles of the monitoring device. Clinic-like carbon-ion irradiations (3Gy(RBE)) of a 70-cm^3^ spherical tumour located in a head-sized PMMA cylinder were performed at the HIT in Germany. To mimic inter-fractional variations, a 2-mm-thick air inhomogeneity was inserted in the head model before, at the entrance, in the middle, and at the distal end of the tumour volume. Emerging secondary ions were measured at 10°,20°,30°,40°,50° relative to the beam axis, using a mini-tracker composed of two pixelated silicon detectors (Timepix3). Data analysis was developed to quantify the capability of the method to detect the air cavities in each measurement. With a mini-tracker positioned at 30° with respect to the beam axis, the 2-mm air cavity could be detected at all positions between the entrance and end of the tumour with a significance of at least 5 sigma. For an air cavity positioned before the tumour, it was detectable from 10° on with a significance of at least 10 sigma. This work demonstrates the potential of tracking secondary charged particles for detecting small inter-fractional variations in a clinic-like carbon-ion treatment.

## P072 -

### Concept of a device for measurement of intensity of medical charged particle beam distribution

#### A. Grigorieva^1^, A. Bulavskaya^1^, Y. Cherepennikov^2^, I. Miloichikova^2,3^, S. Stuchebrov^1^

##### ^1^National Research Tomsk Polytechnic University, Research School of High-Energy Physics, Tomsk, Russian Federation ^2^National Research Tomsk Polytechnic University, School of Nuclear Science & Engineering, Tomsk, Russian Federation ^3^Cancer Research Institute of Tomsk National Research Medical Center of the Russian Academy of Sciences, Radiotherapy Department, Tomsk, Russian Federation

This study describes the concept of a method for measurement of heavy charged particle beams' flux density distribution in the transverse plane. Currently, various approaches are used to modulate a radiation field most closely following the tumor shape. However, all of these methods require continuous monitoring of the dose and spatial parameters of the beams. Therefore, the development of new methods for determining the beam's spatial parameters during creation and modernization of installations for hadron radiation therapy is a vital task. One of the main requirements to detectors intended to determine parameters of therapeutic hadron beams is the possibility of real-time measurement without beam disturbance. Detectors like dosimetry films, thermoluminescent dosimeters and dosimetry gels require post-processing and do not meet these requirements. Real-time measurements of the beam flux density distribution in the transverse plane are possible with the use of matrix detectors where diodes or ionization chambers are distributed in the examined plane. As a rule, such detectors provide low resolution that becomes insufficient as hadron therapy and the approaches to monitoring parameters of beams in medical applications develop.

This study describes the concept of the measurement method based on mathematical reconstruction of beam profiles obtained with multiple scans at different angles with a fixed angle step. This approach will provide for determining the full beam density distribution in the transverse plane and ensuring the continuous monitoring of charged particle beam parameters in both plan verification and treatment. This work is supported by the Russian Science Foundation, project No. 19-79-10014.

## P073 -

### Incorporating a block aperture into MCsquare, a Monte-Carlo dose simulator for proton therapy

#### Jason Holmes^1^, Jie Shan^1^, Jiajian Shen^1^, William Wong^1^, Robert Foote^2^, Samir Patel^1^, Martin Bues^1^, Wei Liu^1^

##### ^1^Mayo Clinic, Department of Radiation Oncology, Phoenix, USA ^2^Mayo Clinic, Department of Radiation Oncology, Rochester, USA

**Purpose:** The RayStation^TM^ treatment planning system (TPS) is capable of modelling block apertures for proton therapy. MCsquare is an open-source Monte Carlo (MC) dose engine for proton therapy, however it currently does not support block apertures. To use MCsquare as a second dose calculation check software for proton therapy, MCsquare needs to be enhanced to support block apertures.

**Methods:** The block aperture has been modeled within MCsquare in a manner consistent with DICOM. During the MC simulation, primary particles are determined to be within the aperture opening using the *crossing-number algorithm*. For validation, circular brass apertures with diameters of 1, 2, 3, 4, and 5 cm were constructed. Mono-energetic plans were delivered through the apertures into a water phantom and measured at various depths. The measurements were compared to the doses calculated by MCsquare and RayStation^TM^ respectively (see Figure 1). Additionally, MCsquare and Raystation^TM^ were directly compared for 10 patient plans with block apertures.

**Results:** Comparing to the absolute point-dose water measurements, MCsquare differed by 1.8% ± 3.6% while RayStation^TM^ differed by 1.2% ± 1.0%. Comparing to the film measurements in water, MCsquare and RayStation^TM^ both performed well with an average 2D Gamma pass-rate of 99.7% and 99.4% (3%/3mm) respectively. A t-test of the film results suggests MCsquare and RayStation^TM^ are statistically indistinguishable. Comparing dose calculations between MCsquare and RayStation^TM^ over 10 patients resulted in 3D Gamma pass-rates of 98.5% (3%/3mm) and 94.1% (2%/2mm).

**Conclusions:** MCsquare, as modified, performed similarly to that of RayStation^TM^ with respect to film measurements and in patients.

## P074 -

### Feasibility of delivering exchanged PBS treatment plans in two gantries of multiroom proton therapy system: a preliminary study

#### Yu-Hua Huang^1,2,3^, Chun-Feng Fang^3^, Tao Yang^3,4^, Lin Cao^3^, Gao-Long Zhang^2,5^, Bao-Lin Qu^4,5^, Yi-Hang Zhang^2^, Zi-Shen Wang^3^, Shou-Ping Xu^3,4,5^

##### ^1^The Hong Kong Polytechnic University, Department of Health Technology and Informatics, Kowloon, Hong Kong ^2^Beihang University, School of Physics, Beijing, China ^3^Hebei YiZhou Cancer Hospital, Department of Radiation Oncology, Zhuozhou, China ^4^The First Medical Center of PLA General Hospital, Department of Radiation Oncology, Beijing, China ^5^Beihang University, Beijing Advanced Innovation Center for Big Data-Based Precision Medicine, Beijing, China

**Purpose**: Inspired by the beam-matching concept in Linacs, the feasibility of delivering exchanged PBS treatment plans without replanning and recalculations in gantries of multiroom proton system is studied.

**Methods**: Beam characteristics for 33 energy levels (70∼226.09MeV) of two gantries (GTR2/4 in YiZhou Cancer Hospital) were measured and established beam-model. 36 cases of nasopharyngeal-carcinoma, lung and prostate cancer were retrospectively selected to generate patient-specific QA plans based on the beam-model of both gantries. Each QA plan was respectively delivered in both gantries. Planar dose measurements were done and were statistically analyzed.

**Results**: Through comparison between two beam-models, their IDD are nearly identical. The spot profiles at each energy level had slight differences. Independent of delivering the QA plans in GTR2/4, the average passing rates of measured doses had not changed much, reaching a high level of 95% when using a 3mm/3% criterion. While the t-test shows these differences were not statistically significant, the Pearson analysis proved a certain correlation between the passing rates of two gantries. Few abnormal cases could be seen when the stringent criterion of 2mm/3% was used, but when using the less rigorous 3mm/3% criterion which was widely used in clinic, there's no exception.

**Conclusions**: The beam characteristics in different rooms showed high consistency and the tolerance was kept within a reasonable range while the difference of the same treatment plan delivered in two gantries met the requirement of clinical treatment. It's recognized that the treatment plan could be delivered directly in different rooms.

## P075 -

### To evaluate the effects of spot position error on the dose distribution of proton pencil beam scanning (PBS) plans

#### Jhih Kai Jhang^1^, Yuki Tominaga^2^, Takahiro Waki^3^, Mitsutoshi Tada^1^

##### ^1^Tsuyama Chuo Hospital, Radiation technology, Tsuyama, Japan ^2^Okayama University, Graduate School of Health Sciences, Okayama, Japan ^3^Tsuyama Chuo Hospital, Radiology, Tsuyama, Japan

**Purpose**: To evaluate the effects of spot position error on the dose distribution of proton pencil beam scanning (PBS) plans.

**Methods**: This study involved 10 consecutive prostate cancer patients underwent treatment with PBS at our hospital. All patients were selected two opposing beam angles (90 degree and 270 degree), and the all energy ranges were from 191.7 MeV to 159.9 MeV. The spot profiles at center coordinate in air were measured using XRV-2000 (Logos systems). The measurement gantry angles were 90 degree and 270 degree. The offset of the spot positions were defined by the displacement vectors between the center of analyzed Gaussian distributions and the center coordinate. With the use of in-house scripts, the post-shifted coordinates were applied to the initial plans as replacements and recalculated by the RayStation 6.2. Further, we compared the dose volume histograms for the contours of CTV, PTV, Rectal wall, and Bladder wall between the initial plans and the modified plans.

**Results**: At the modified plans, the target coverage of D_95_ at CTV and PTV were decreased the average of 0.1% and 1.15%, respectively. In the critical organs, the average dose of Rectum wall decreased by an average of 3.44% while that of Bladder wall increased by an average of 3.13%.

**Conclusion**: We could evaluate the dose distributions that applied XRV measurement results using the in-house scripts. Although the dose distribution of the each contours changes depending on the direction and the amount of the spot shifts, these results were clinically acceptable.

## P076 -

### Localisation of air cavities from charged-fragment track distribution in carbon-ion radiotherapy

#### Laurent Kelleter^1,2^, Laura Marie Hélène Ghesquiere-Dierickx^1,2,3^, Annika Schlechter^1,2,3^, Renato Félix-Bautista^1,2,4^, Gernot Echner^1,2^, Tim Gehrke^1,2,5^, Marcus Winter^2,3,6^, Maria Martišíková^1,2^

##### ^1^German Cancer Research Centre DKFZ, Department of Medical Physics in Radiation Oncology, Heidelberg, Germany ^2^Heidelberg Institute for Radiation Oncology HIRO, National Centre for Research in Radiation Oncology NCRO, Heidelberg, Germany ^3^University of Heidelberg, Heidelberg Medical Faculty, Heidelberg, Germany ^4^University of Heidelberg, Department of Physics and Astronomy, Heidelberg, Germany ^5^Heidelberg University Hospital, Department of Radiation Oncology, Heidelberg, Germany ^6^Heidelberg University Hospital, Heidelberg Ion-Beam Therapy Center HIT Department of Radiation Oncology, Heidelberg, Germany

The dose distribution in carbon-ion radiotherapy is susceptible to anatomical changes in the patient. Charged fragments produced in nuclear reactions of the carbon-ion beam with the patient tissue have been proposed to potentially enable online treatment monitoring. A model of a human head (homogenous PMMA cylinder) was irradiated with a realistic carbon-ion treatment plan (3 Gy RBE) for a spherical tumour (70 cm^3^ volume) at the centre of the phantom. The emerging fragment tracks were measured using a mini-tracker based on Timepix3 sensors at various angles relative to the beam axis. Anatomical changes were simulated by introducing an air cavity of 2 mm thickness at different positions along the beam path. The measurements were reproduced in a Monte Carlo simulation using Fluka. The analysis focused on the capability of the method to determine the position of the air cavity along the beam axis. The variation of the detection angle leads to a trade-off between the number of detected fragments and the achievable spatial resolution. Changes in the measured fragment distribution were more significant for smaller angles, whereas the position of the air cavity could be determined with a precision of 5 mm for detection angles larger than 30 degrees. The localisation of small air cavities in geometrical phantoms was found to be feasible and the method could be used with a future clinical prototype. Moreover, different scenarios for the combination of the available information from multiple trackers are being discussed.

## P077 -

### A novel clinical workflow to assess the dosimetric errors of compensator manufacture defects in double scattering proton therapy

#### Josh Kilian-Meneghin^1^, Ke Nie^1^, Yin Zhang^1^, Xiao Wang^1^, Bo Liu^1^, Ning Yue^1^, Taoran Cui^1^

##### ^1^Rutgers Cancer Institute of New Jersey, Department of Radiaiton Oncology, New Brunswick- NJ, USA

Range compensators are used in double scattering proton therapy (DSPT) to conform the distal end of dose distribution to treatment target. The accurate manufacture of compensator is therefore essential to ensure target coverage and OARs dose. The QA of compensator is usually performed by comparing its planned and measured height-maps with 2D gamma analysis (1mm/1%), which fails to reflect the dosimetric errors due to manufacture defects. This study aims to design a novel workflow to quantify the dosimetric impacts for compensator QA. Patients treated with DSPT at a single institution were retrospectively reviewed. 12 patients with at least one compensator which failed the original QA were selected. A measured height-map of the compensator was extracted from its CT scan used for the original QA. The RT-Plan DICOM file of the original plan was then modified by replacing the planned height-map with the measured one, and imported back to TPS for dose calculation. Using the original beamline parameters, a plan representing the actual treatment compensator was created. The coverage of PTVs in the plans using the actual compensator were comparable with the original plans with no statistically significant difference between the V100% (Wilcoxon; p=0.89) of the PTVs. The tested compensators that failed the gamma analysis were therefore clinically viable and the physician can now assess the impact within the TPS. The proposed workflow directly quantifies the dosimetric impact of manufacture defects in compensator with higher specificity than gamma analysis method, and should be adopted for compensator QA in DSPT.

## P078 -

### Deep learning prediction of proton range and SOBP for passive scattering proton therapy

#### Young Kyu Lee^1^, Sang Hee Ahn^2^, Chankyu Kim^1^, Dongho Shin^1^, Se Byeong Lee^1^, Young Kyung Lim^1^, Jong Hwi Jeong^1^, Haksoo Kim^1^

##### ^1^National Cancer Center, Proton Therapy Center, Goyang-si- Gyeonggi-do, Korea Republic of ^2^Yonsei University College of Medicine, Radiation Oncology, Seoul, Korea Republic of

**Purpose**: In the proton therapy with passive scattering mode, the range (C-range) and spread-out Bragg peak (C-SOBP) values using the conversion algorithm from IBA (CONVALGO) are different with those of the actual measurements (M-range, M-SOBP) in the water. So, C_range and C_SOBP should be measured to find the desired M_range and M_SOBP for every quality assurance (QA). The aim of this study was to develop a predictive model of proton range and SOBP using deep learning and reduce the time spent on QA.

**Methods**: Twenty-four beam range options in the double scattering mode at the National Cancer Center in Korea were used in this analysis. A total of 5,475 QA measurement data were collected, and a prediction model was developed using deep neural network. To predict C-range and C-SOBP, the algorithm build a training model using six QA parameters (proton_main_option, proton_sub_option, C-range, C-SOBP, M-range, M-SOBP). Additional 110 test sets were used to verify the training model.

**Results**: In the analysis of range, the maximum, mean absolute error (MAE) and root mean square error (RMSE) were 0.18 cm, 0.056 cm and 0.075 cm, respectively. In SOBP analysis, the maximum, MAE and RMSE were 0.8 cm, 0.217 cm and 0.288 cm, respectively.

**Conclusions**: An acceptable QA criteria is based on a difference of less than 0.1 cm. The MAE of 0.056 cm for the range is considered a positive result. The maximum error for range is 0.18 cm, but it can be a tool in setting the initial C-range value before QA.

## P079 -

### High speed detector for Flash proton therapy QA

#### C.H. Lin^1^, F.X. Chang^2^, H.T. Chang^2^, C.H. Hsing^3^, Y.C. Tsai^4^, H.C. Huang^4^

##### ^1^Academia Sinica, Institute of Physics, Taipei, Taiwan ^2^Liverage Biomedical Inc, Technology, Taipei, Taiwan ^3^Chang Gung University, Institute for Radiological Research, Taoyuan, Taiwan ^4^Chang-Geng Memorial Hospital- Linko, Particle Physics and Beam Delivery Core Laboratory, Taoyuan, Taiwan

Flash proton therapy has the potential to revolutionize radiation therapy, which treats the target tumor with an ultra-high dose rate (> 40,000 cGy/second) and a very short exposure time (< 1 second). This new cancer treatment technology also brings new requirements to equipment used in the machine quality and assurance (QA) control. Here we present a novel detector system, CROSSflash, with an ultra-high measuring rate up to 40,000 Hz. With such a system, one can measure the time evolution of all beam parameters required by the Flash proton therapy QA down to 25 μsec precision. In this report, we provide an overview of the design of CROSSflash detector and its preliminary test results with large proton beam current.

## P080 -

### Clinical experience of quality assurance for beam-specific apertures used in pencil beam scanning proton therapy

#### Chin-Cheng Chen^1^, Haoyang Liu^2^, Scott Luckman^2^, Emily Aunio^2^, Niek Schreuder^3^, Dennis Mah^2^

##### ^1^New York Proton Center, Medical Physics, New York, USA ^2^ProCure Proton Therapy Center, Medical Physics, Somerset, USA ^3^Leo Cancer Care, Medical Physics, Middleton, USA

**Purpose:** Clinical experience of quality assurance (QA) program for beam-specific apertures used in pencil beam scanning (PBS) proton therapy is reported.

**Methods:** A uniform PBS QA field with a single central spot was trimmed by a square aperture. The spot provided the position of the PBS isocenter which should correspond to the center of the aperture shape (Figure 1). Deviations between the two centers were recorded in both commissioning and monthly QA. Additionally, the results of patient-specific quality assurance (psQA) measured as 2D planar doses with beam-specific apertures mounted at the planned snout positions were reviewed.

**Results:** The averaged isocenter offsets of the collimated PBS QA fields were 0.84, 1.05, and 0.74 mm for 10, 18, and 25-cm snout respectively observed in 2 treatment rooms from January 2019 to January 2021. The psQA results of total 62 collimated PBS fields for patient treatments were reviewed. The averaged snout position of the collimated PBS fields was 18.6 cm, comparable with the 20-cm snout position in monthly QA. The averaged passing rate in Gamma evaluation (3%, 3 mm) were 97.6% for all 2D dose comparisons, and no significant dependence was found over the range of snout sizes and positions (Figure 2).

**Conclusion:** The isocenter offsets were all ∼1 mm without systemic drift in 25 months. To have the best dosimetric benefits from collimated PBS fields, only snouts with small air gaps (snout positions < 20 cm) should be used for patient treatments.

## P081 -

### New perspectives in the development of a Compton telescope for treatment monitoring

#### Gabriela Llosa^1^, Luis Barrientos^1^, José Bernabéu^1^, Marina Borja-Lloret^1^, José Vicente Casaña^1^, Fernando Hueso-Gonzalez^1^, Carlos Lacasta^1^, Enrique Muñoz^1^, Ana Ros^1^, Jorge Roser^1^, César Senra^1^, Carles Solaz^1^, Rita Viegas^1^

##### ^1^Instituto de Física Corpuscular, CSIC-U. Valencia, Paterna, Spain

The IRIS group of IFIC has made significant progress in the development of a multilayer Compton camera for treatment monitoring based on prompt gamma detection. The system is composed of three planes of LaBr3 crystals coupled to SiPMs. The new version employs the readout board AliVATA, which can handle the three detectors with a single board. An alternative readout system based on PETsys electronics with improved timing resolution has also been evaluated. The system incorporates a spectral image reconstruction code that can estimate position and energy of the incoming photons both in two- or three-interaction events, and also combines all types in a joint reconstruction. Furthermore, the group is addressing the effect of the background events on the reconstructed images. After exploring the degradation caused by the different background types, a twofold reduction strategy is followed. First, with the incorporation of silicon detectors that can reject the charged particles coming from the beam or generated in secondary interactions. Second, through the use of convolutional neural networks to separate signal and background events. Both methods yield a substantial improvement of the images. As a result, the system has detected 3 mm Bragg peak shifts with 150 MeV proton beams impinging a PMMA target, and also 1 mm variations in the position of a graphite target impinged by a 18 MeV proton beam. The system is now ready for new tests in clinical beams in collaboration with Quirónsalud protontherapy centre in spring 2021.

## P082 -

### GPU-accelerated Monte Carlo simulation program for PET-based proton range verification

#### L. Ma^1^, Y. Zhong^1^, M. Chen^1^, X. Jia^1^, X. Gu^1^, Y. Shao^1^, W. Lu^1^

##### ^1^University of Texas Southwestern Medical Center, Radiation Oncology, Dallas, USA

Range verification is critical in proton therapy to ensure accurate treatment delivery. In-beam positron emission tomography (PET), combined with mid-range probing, are promising for in-vivo range measurement. To translate in-beam range verification from the benchtop to bedside, an ultra-fast Monte Carlo (MC) tool is demanded for simulating both proton-induced positron activity and radiation dose. We built a GPU-based MC proton program, gPMC v3.0, in which sampling for the non-elastic nuclear process is implemented via a lookup table (LUT). This new sampling method can generate a full spectrum of products for the non-elastic process, enabling the scoring of positron emitters for the study of PET-based range verification. The inputs of gPMC v3.0 are a proton treatment plan, voxelized patient geometry data and physics data, which includes stopping powers, cross sections, and LUTs generated by the benchmark algorithm for sampling the phase-space of the products of the non-elastic process. Simulation results include dose distribution and spatial distribution of positron emitting isotopes including C10, C11, N13, and O15. We benchmarked gPMC v3.0 and validated it with GATE. gPMC v3.0 achieved sub-millimeter accuracy: ranges (R50) of benchmark results versus gPMC results are 255.4/255.6 mm, 247.6/247.7 mm and 244.5/244.9 mm for dose, O15 and C11 respectively, of 200MeV protons in homogeneous soft tissue. gPMC v3.0 can finish a simulation of 10^7 protons with energy of 0.5-240MeV in 1-4 seconds, which makes it feasible for in-beam PET-based range verification of a clinical proton plan associated with patient-specific geometry.

## P083 -

### LET signature in laser CT of polymer gel dosimeters exposed to PBS proton-therapy fields; potential enhancement of proton-therapy QA

#### Marek Maryanski^1,2^, Alireza Kassaee^3^, Stephen Avery^3^

##### ^1^Faculty of Applied Physics and Mathematics / Gdansk University of Technology, Institute of Nanotechnology and Materials Engineering, Gdansk, Poland ^2^MGS Research Inc, dba 3D Dosimetry, Madison CT, USA ^3^University of Pennsylvania / Perelman School of Medicine, Radiation Oncology, Philadelphia PA, USA

**Background:** Laser CT (LCT) of polymer gel dosimeters (PGD) is known to generate accurate 3D maps of physical dose in proton-therapy fields. Also, effects of PGDs' chemical modifications on LET-enhanced response have been reported. This may enable new measurement protocols to generate high-definition 3D maps of LET in PGD phantoms. If sufficiently streamlined, such protocols could help optimize the clinical utility of QA measurements, enhance treatment outcomes and minimize side effects. Here we present experimental evidence for a practical alternative to multiple chemical modifications, potentially allowing for a single-PGD phantom irradiation protocol.

**Methods:** The new optical signature of LET utilizes Mie-scattering on radiation-induced polymer nanoparticles' clusters. A PBS plan (250 cGy maximum dose; nominal proton energies up to 140 MeV) was delivered to a CrystalBall™ PGD phantom: a thin-wall Pyrex glass sphere of 166 mm OD with a cylindrical neck for reproducible positioning and scanning in the fast-scanning OCTOPUS™ LCT. Both the PGD phantom and the scanner were manufactured by MGS Research, Inc., Madison, CT. An experimental variable-aperture diffuser was mounted in front of the LCT's photodetector.

**Conclusions:** Feasibility of a practical new approach to 3D mapping of both dose and LET using a single-PGD phantom irradiation has been demonstrated.

## P084 -

### A method for improving the spatial resolution of helium-beam radiography and its consequences for calibrations regarding water-equivalent thickness

#### Margareta Metzner^1,2^, Carlo Amato^1,2^, Gernot Echner^1,2^, Oliver Jäkel^1,2,3^, Mária Martišíková^1,2^, Tim Gehrke^1,2,4^

##### ^1^German Cancer Research Center DKFZ, Department of Medical Physics in Radiation Oncology, Heidelberg, Germany ^2^National Center for Radiation Research in Oncology NCRO, Heidelberg Institute for Radiation Oncology HIRO, Heidelberg, Germany ^3^University Hospital Heidelberg, Heidelberg Ion-Beam Therapy Center HIT, Heidelberg, Germany ^4^University Hospital Heidelberg, Department of Radiation Oncology, Heidelberg, Germany

To fully use the potential of ion-beam radiotherapy, an exact target positioning and accurate knowledge about the relative stopping power distribution in the patient are crucial. Using the same kind of particles for treatment and imaging, helium-beam radiography (αRad) offers a promising alternative to the clinically used X-ray radiography for positioning. In addition to less dose being deposited, a helium-beam radiograph shows the integrated stopping power along the beam axis directly. This renders an additional validation of the stopping power calculated on basis of the planning CT feasible. This contribution presents a technique which improves the spatial resolution (SR) of αRad. Using higher beam energies than required for the ions to traverse the object, multiple Coulomb scattering inside the object is reduced. Since our radiography method is based on a thin energy deposition detector, an energy degrader compensates for the increased energies behind the object and ensures that the rising edge of the Bragg peak still lays in the energy deposition detector.

The influence of the copper energy degrader on quantitative calibrations regarding water-equivalent thickness (WET) is calculated and discussed. The SR could be increased from (0.54 ± 0.01) lp/mm to (0.69 ± 0.02) lp/mm, corresponding to an improvement of (29 ± 5) %. The single-ion WET precision increased from 1.2 % to only 1.5 %. Hence, a choice between better SR or CNR becomes feasible, offering the possibility to adjust image characteristics to their corresponding application.

## P085 -

### Test of a particle counting silicon detector with therapeutic proton beams

#### Vincenzo Monaco^1,2^, Mohammed Abujami^1,2^, Roberto Cirio^1,2^, Cosimo Galeone^1^, Sara Garbolino^2^, Simona Giordanengo^2^, Omar Hammad Ali^3^, Felix Mas Milian^1,2,4^, Giovanni Mazza^2^, Marco Mignone^2^, Richard James Wheadon^2^, Matteo Centis Vignali^3^, Anna Vignati^1,2^, Oscar Ariel Martì Villarreal^1,2^, Roberto Sacchi^1,2^

##### ^1^Università degli Studi di Torino, Physics Department, Turin, Italy ^2^Istituto Nazionale di Fisica Nucleare, Sezione di Torino, Torino, Italy ^3^Fondazione Bruno Kessler, Fondazione Bruno Kessler, Trento, Italy ^4^Universidade Estadual de Santa Cruz, Departamento de Ciencias Exatas e Tecnologicas, Bahia, Brazil

**Purpose:** Developments and tests of a prototype of an innovative system for the monitoring of therapeutic proton beams are presented. The device is operated in particle counting mode, allowing to enhance the sensitivity and readout speed with respect to gas detectors.

**Materials and Methods**: Several silicon sensors based on the LGAD technology were designed and produced at Fondazione Bruno Kessler (Trento, Italy), with the final ones covering an area of 2,7x2,7 cm^2^, segmented in 146 strips and with 50 μm active thickness. External amplifiers and a digitizer were used for preliminary tests. The readout of the final prototype employs custom frontend ASIC chips to amplify and discriminate the signals from 24 strips in a wide charge range with a maximum dead-time of 10 ns. Non linearity effects due to signal overlapping are mitigated with algorithms, based on logical combinations of signals from neighboring strips, implemented in the FPGA used for data acquisition.

**Results:** Tests performed on synchrotron and cyclotron clinical proton beams, using a pin-hole chamber for the independent measurement of the particle flux, show the possibility to keep the counting error < 1 % up to beam fluxes of 5·10^8^ p/(cm^2^·s) (Fig.1).

**Conclusions:** Tests of LGAD silicon sensors on therapeutic proton beams demonstrate their capabilities as monitoring detectors. The use of this technology in the clinical practice requires further studies to improve their radiation resistance and a finer segmentation to operate at therapeutic rates.

## P086 -

### Proton range verification with prompt gamma-ray timing and on-line proton bunch monitoring

#### F.F. Permatasari^1^, B. Lutz^2^, G. Pausch^3,4^, K. Roemer^2^, S. Schellhammer^1,5^, A. Wagner^2^, D. Weinberger^2^, R.D. Werner^1,6^, T. Werner^1,5,7^, T. Kögler^1,5^

##### ^1^OncoRay – National Center for Radiation Research in Oncology, Faculty of Medicine and University Hospital Carl Gustav Carus- Technische Universität Dresden- Helmholtz-Zentrum Dresden - Rossendorf, Dresden, Germany ^2^Helmholtz-Zentrum Dresden – Rossendorf, Institute of Radiation Physics, Dresden, Germany ^3^OncoRay – National Center for Radiation Research in Oncology , Faculty of Medicine and University Hospital Carl Gustav Carus- Technische Universität Dresden- Helmholtz-Zentrum Dresden - Rossendorf, Dresden, Germany ^4^Helmholtz-Zentrum Dresden – Rossendorf , Institute of Radiooncology – OncoRay, Dresden, Germany ^5^Helmholtz-Zentrum Dresden – Rossendorf, Institute of Radiooncology – OncoRay, Dresden, Germany ^6^Martin-Luther-Universität Halle, Institute for Physics, Halle, Germany ^7^Currently with: Technische Universität Dresden, Institute of Nuclear and Particle Physics, Dresden, Germany

Range verification is an important prerequisite to unfold the full potential of the finite range of proton beams and to improve treatment precision. The prompt gamma-ray timing (PGT) technique offers a non-invasive approach for range verification using the measured time distribution of the prompt gamma rays produced in the patient. PGT dispenses with a heavy collimator and can be integrated into existing treatment gantries. However, the high sensitivity of this technique to any instabilities in the proton bunch periodicity is a major challenge and demands online monitoring of the proton bunch arrival time. Therefore, we have developed a proton bunch monitor (PBM) comprising fast-scintillating fibers with a double-sided silicon photomultiplier readout. Placing the PBM in the beam halo allows the direct measurement of the proton arrival time at clinical beam intensities while maintaining a processable trigger rate. In a proof-of-principle experiment with a thick acrylic glass target and defined cylindrical air cavities as well as tissue equivalent inserts, a direct monitoring of proton bunches was carried out together with the PGT measurement. With the use of the PBM, another important step towards the clinical translation of the PGT method was taken. **Acknowledgement**: This work has been generously supported by StrahlenSchutzSeminar in Thüringen e.V.

## P087 -

### Hybrid Compton-PET Imaging for Range Verification in Proton Therapy

#### Mohammad Javad Safari^1^, Andreas Zoglauer^2^, Giulio Lovatti^1^, Vasiliki Anagnostatou^1^, Munetaka Nitta^1^, Hideaki Tashima^3^, Akram Mohammadi^3^, Taiga Yamaya^3^, Peter Thirolf^1^, Georgios Dedes^1^, Katia Parodi^1^

##### ^1^Ludwig Maximilians Universität München LMU, Department for Medical Physics, Munich, Germany ^2^University of California, Space Sciences Laboratory, Berkeley- California, USA ^3^National Institute of Radiological Sciences NIRS, Department of Advanced Nuclear Medicine Sciences- National Institutes for Quantum and Radiological Science and Technology QST, Chiba, Japan

One of the main challenges in proton therapy is the uncertainty in the exact positioning of the proton beam distal dose fall-off (Bragg-peak) in the patient. Currently, prompt gamma-ray (PG) imaging and positron emission tomography (PET) as independent tools for non-invasive proton beam range verification are regarded as the most promising and investigated approaches, but as there is no clear demonstration yet on which method provides the best results, we propose to provide a detector that can acquire all signals. Therefore, in the scope of an ongoing collaboration between the LMU with NIRS-QST the concept of a so-called Whole-Gamma-Imaging (WGI) as a combined Compton camera ring array and a PET scanner in one imaging scheme has been further extended for proton range verification. In this project, the performance of a small animal hybrid-scanner, comprising two scatter and four absorber rings [Fig1c], for the detection of the proton range was investigated using an enhanced version of MEGAlib (Medium-Energy Gamma-ray Astronomy library) as a dedicated simulation, data analysis, and image reconstruction toolkit for Compton, PET, and Compton-PET imaging. The initial simulation results for a low energy proton beam (50MeV) using the proposed pre-clinical hybrid-scanner showed that this imaging system provides the opportunity to detect and utilize all prompt/delayed gamma emissions to reconstruct the proton range [Fig2a-2d]. **Acknowledgement**: This research is supported by the Alexander von Humboldt Foundation, the Bavaria-California-Technology-Center (BaCaTeC), and the QST International-Research-Initiative (IRI).

## P088 -

### Toward log-data based patient-specific quality assurance in Osaka-HIMAK

#### Masaaki Takashina^1^, Asuka Komatsu^2^, Noriaki Hamatani^1^, Toshiro Tsubouchi^1^, Masashi Yagi^3^, Tatsuaki Kanai^1^

##### ^1^Osaka Heavy Ion Therapy Center, Department of Medical Physics, Osaka, Japan ^2^Osaka University Graduate School of Medicine, Division of Health Sciences, Osaka, Japan ^3^Osaka University Graduate School of Medicine, Department of Heavy Ion Radiotherapy, Osaka, Japan

Purpose: To establish a method of patient specific quality assurance (PSQA) for carbon ion radiotherapy utilizing the irradiation log data to detect errors of dose distribution related to scanning machine performance.

**Materials and Methods**: As a first step, we investigated the relationship between dose error and spot position error, the latter of which is detected by spot position monitor (SPM). A 6x6x6 cm^3 cubic target in water was considered, and constant physical or clinical dose distribution was made by optimization. We simulated influence of random position error on the flatness, where the errors from planned positions were supposed to have a Gaussian distribution, and its standard deviation (SD) was varied. The dose calculation was performed by treatment planning system. We also investigate whether the dose deviation stem from the position error made above could be detected by measurement using 2D-array detector.

**Results and discussions**: The results of the simulation show that the flatness increases 2 percent when SD is 0.42 mm for both the physical and clinical dose cases. Although the measurement is in progress, this result could be a basis to determine the criteria for the log-data based PSQA. Since the spot position recorded as log data includes error of the SPM as well as beam position error itself, evaluation of SPM accuracy is required.

**Conclusion**: The SD value of spot position error making the flatness worse by 2 percent was derived by simulation, which will be connected to the criteria for the log-data based PSQA.

## P089 -

### Dose perturbation effect of titanium implant in post-operative proton therapy of head and neck cancer.

#### M. Tomida^1^, T. Kamomae^2^, T. Nishio^3^, T. Yanagi^4^

##### ^1^Narita Memorial Proton Center, Medical Physics, Toyohashi, Japan ^2^Nagoya University Hospital, Department of Radiology, Nagoya, Japan ^3^Osaka University Graduate School of Medicine, Medical Physics Laboratory- Division of Health Science, Osaka, Japan ^4^Narita Memorial Proton Center, Department of Proton, Toyohashi, Japan

In the post-operative proton therapy for head and neck cancer, there is a possibility that the patients have titanium implants around the site where the tumor existed. Although it is well known that implants perturb the dose distribution of the proton therapy, there are many different types of implants. The purpose of this study is to evaluate the fundamental properties of the titanium implant on the dose distribution for the case that we have actually experienced in pencil beam scanning proton therapy. The thickness of the titanium implant used in this study was 0.7 mm (Figure 1). The treatment plan was created by pencil beam scanning on the slab phantom with the cubic target. The phantoms, which inserted the radiochromic films with 1 mm interval around distal fall-off region with or without the implant, were irradiated. The dose profiles obtained from the irradiated films were compared with the calculated profiles from the treatment planning system. The measured proton range of R_90_ on beam-axis with the implant was 1.0 mm shorter than that without the implant. Meanwhile, the calculated proton range difference, with or without the implant, in the treatment planning system was 2.0 mm. The calculation result from the treatment planning system reacted to this implant; however, from a clinical perspective, there is need to carefully consider the slight overestimation of this effect in the treatment planning system.

## P090 -

### Flatness calibration of large area XY strip parallel-plate ionization chamber (PPIC)

#### Thien Tran^1^, Chi-wen Hsieh^2^, Ei-fong Chen^1^, Kun-Jong Chiang^1^, Trang Hoang^3^, Lin Chih-Hsun^4^

##### ^1^Natinal Central University, Physics, Taoyuan, Taiwan ^2^National Chia-Yi University, Department of Electrical Engineering, Chia-Yi, Taiwan ^3^University of Science- VNU-HCM, Department of Nuclear Physics- Nuclear Engineering- and Medical Physics, Ho Chi Minh city, Viet Nam ^4^Academia Sinica, Institute of Physics, Taipei, Taiwan

Recent advanced pencil beam scan in proton therapy deploys non-uniform beam profile which scans across a large radiation field and complete high conformity treatment. High dose gradient in beam profile is one of its characters. 2D array of ICs or XY strip PPIC (Parallel Plate Ionization Chamber) occupying large area, high spatial resolution and large dynamical range, are potential candidates for such purpose. XY strip PPIC uses less readout channel and renders better spatial resolution in comparison to 2D array of ICs. Calibration of large area XY strip IC consists two parts. Electronics can be calibrated by standard pulse generators. In this study, uniformity of PPIC, characterized by a large area of 345.44 × 345.44 mm2, will be calibrated by point-like mini X-ray source which provides stable and small localized radiation. The stability and compact size of the mini X-ray introduce the operational simplicity and variation to be less than 1% for flatness calibration of XY strip PPIC.

## P091 -

### Validation of the accuracy of a spot position monitor in carbon ion therapy for patient-specific QA based on log-file

#### Toshiro Tsubouchi^1^, Noriaki Hamatani^1^, Masaaki Takashina^1^, Masashi Yagi^2^, Tatsuaki Kanai^1^

##### ^1^Osaka heavy Ion Center, Physics, Osaka, Japan ^2^Osaka University Graduate School of Medicine, Carbon Ion Radiotherapy, Suita, Japan

**Background**: Carbon ion therapy with raster scanning is performed at Osaka Heavy Ion Therapy Center. During irradiation, delivered spot positions and MU values are measured by a spot position monitor (SPM) and dose monitor, installed in the nozzle, and recorded in a log-file. Now we are developing a new patient-specific QA (PSQA) method based on the log file. To start the new PSQA, Verifying the precision of these monitors is important. This study reports on SPM accuracy was evaluated.

**Methods**: To begin with, 9 spots were irradiated and measured by an EBT3 film, aligned at Isocenter. After the irradiation, spot positions recorded in the log file were compared with those measured with the film.

**Results**: Figure 1 shows spot positions measured by the film (film data), recorded in the log file (SPM data), and planned ones. The maximum deviation between film data and SPM data (SPM data – film data) was 1.78 mm.

**Conclusion**: A large difference between SPM data and film measurements was observed. It is assumed that this difference is due to different reference points (center positions) of SPM and the film, different spatial resolutions of both detectors, and the systematic error inherent in the SPM. Revealing the systematic error of SPM quantitatively will lead to conducting the new PSQA with high accuracy.

## P092 -

### Dictionary based MLEM-algorithm for real-time proton range verification from PET data: submilimretic precision in clinical dose.

#### Victor Valladolid Onecha^1^, Jose Manuel Udías^1,2^, Luis Mario Fraile^1,2^, Pablo Galve^1^, Daniel Sánchez-Parcerisa^1,2^, Samuel España^1,2^, Paula Ibañez^1^, Andrea Espinosa^1^

##### ^1^Complutense University of Madrid UCM-, Faculty of Physical Science- Nuclear Physics Group and IPARCOS- EMFTEL- CEI Moncloa, Madrid, Spain ^2^Instituto de Investigación Sanitaria del Hospital Clínico San Carlos, IdISSC, Madrid, Spain

Proton therapy can not reach its full potential due to uncertainties in proton range calculation. A method to solve this problem is in-vivo proton range verification using PET signal. In this work we propose a fast and accurate dose reconstruction algorithm based in Monte Carlo (MC) simulation from activation maps. Prior to irradiation, we precalculate the dose deposition and activity distribution produced on the patient by each individual pencil beam (PB) in the irradiation plan. During or right after irradiation, with an MLEM algorithm, we calculate the linear combination of the pre-calculated PB activities that best fits the observed PET activation data. This method can incorporate the complete physics in the precalculation phase, employing for instance MC packages for proton therapy and PET, making it possible dose reconstruction in real time since the matching linear combination of PBs is obtained within seconds in a common GPU. To analyze the accuracy of the program we have reconstructed the activation of a 1Gy SOBP over a prostate CT (Fig1.). This activation has been deviated from the reference position 1,3,and5 mm and reconstructed using the same initial conditions. R50 deviation results obtained from this study (Fig 2) show that the program is able to detect minimal deviation from the plan (precision <1mm) using clinical doses. In addition, the program only takes less than 10 seconds to do the reconstruction. Its really high precision and its velocity show the potential of this method for in-vivo proton and dose verification.

## P093 -

### Monthly QA from different gantry angles using an ionization chamber array and a rotating unit

#### Gloria Vilches-Freixas^1^, Ilaria Rinaldi^1^, Esther Decabooter^1^, Erik Roijen^1^, Bas Nijsten^1^, Mirko Unipan^1^, Geert Bosmans^1^

##### ^1^Department of Radiotherapy MAASTRO- GROW-School for Oncology and Developmental Biology- Maastricht University Medical Centre- Maastricht- The Netherlands, Maastro, Maastricht, Netherlands

**Purpose**: To ensure the quality of the delivered proton treatment plans, the constancy and accuracy of the beam characteristics need to be checked periodically from different gantry angles.

**Materials and Methods**: To monitor the output, field symmetry and flatness, range, and spot size and position at different gantry angles (AAPM TG 224), we developed a monthly QA procedure using a 2D array detector (PTW Octavius XDR 1500) embedded in a rotation unit (PTW Octavius 4D). A modular 32 cm diameter top is used to check the range consistency. We position the device on the couch and with kV imaging we ensure a proper alignment. Using an inclinometer mounted on the gantry nozzle, we measure 18 × 18 cm2 homogeneous fields, SOBP, spot patterns for different energies, and a 2D field for the range verification, at five gantry angles. We perform gamma analysis to compare experimental and TPS dose distributions, and we also extract all beam parameters for each angle.

**Results**: We developed a consistent monthly QA procedure. Despite the resolution of the ionization chamber array (7.1 mm detector spacing), this setup is sensitive to 1 mm shifts in spot positioning and spacing and to errors manually introduced in a 2D field (Fig.1). The difference between consecutive energies (1.1 MeV gap) can be easily detectable (Fig.2).

**Conclusion**: With one setup the monthly QA at different gantry angles can be performed with a significant time gain. We are planning to extend the current procedure to check other beam parameters.

## P094 -

### Patient specific QA of scanning beam carbon ion radiotherapy with rotating gantry for choroidal melanoma in clinical trial

#### S. Yonai^1^, N. Saotome^1^, M. Sakama^2^, N. Kanematsu^1^

##### ^1^National Institutes for Quantum and Radiological Science and Technology, National Institute of Radiological Sciences NIRS- Department of Accelerator and Medical Physics, Chiba, Japan ^2^National Institutes for Quantum and Radiological Science and Technology, QST Hospital, Chiba, Japan

The QST hospital has successfully treated more than 200 patients with choroidal melanoma by broad beam carbon ion radiotherapy (CIRT) with a compensator and a patient-specific collimator since 2001. We decided in 2017 to use scanning carbon ion beam with a rotating gantry for this treatment owing to its flexibilities of dose conformation and beam direction, and then its clinical trial started in April, 2018. In our facility, a commercial 2D ionization chamber array had been employed to verify the 2D dose distribution for any patient specific QA. [1] However, the target volume in choroidal melanoma treatment is typically so small that the 2D array is not appropriate to the verification tool of 2D dose distribution due to its low spatial resolution. Thus, in this clinical trial, we applied new method to the patient specific QA. The method consists of two dosimetric verifications by measurements of depth dose distribution with the Bragg peak chamber and lateral dose distributions at three different depths with the pinpoint chambers, and a verification of beam position at each beam spot with the existing beam position monitor (multi-wire proportional chamber). This method was successfully applied to patient specific QAs for 44 treatment beams of all 22 patients received scanning CIRT for choroidal melanoma in this clinical trial until July, 2019. Here, we provide the summary of the QA results and introduce the current QA procedure based on this clinical trial.

## P095 -

### Identification of HU window for accurate contouring of metal implants

#### Jen Yu^1^, Andrew Wroe^1^, Alonso Gutierrez^1^

##### ^1^Miami Cancer Institute, Radiation Oncology, Miami, USA

**Purpose**: To identify an appropriate HU window in extended-HU CT to accurately contour the metal implants encountered in proton therapy.

**Materials**: CT scans were acquired with a phantom and three known-size and -composition plugs of SS, Ti and Al. Each plug was scanned with the two clinical protocols (pelvis and brain) in the center and at the periphery of the phantom. Each scan was reconstructed with 4 configurations: standard reconstruction, reconstruction with iMAR, with iMAR and extended HU, and with iMAR, extended HU and small FOV (100 cm). The standard FOV is 500 cm. Total 48 CT data sets were used for data analysis. Different HU windows were experimented for contouring, with the resultant contour compared in dimension to the measured plug size to determine the most accurate window. The average, minimum, maximum, and coefficient of variations (CV) of the HU numbers for each metal were also reported.

**Results**: The HU numbers of the three metals are substantially different. Table 1 lists the HU values per metal and per protocol, as well as per metal only. Table 2 summarizes the HU window for accurate contouring of the 3 metals. These defined windows depend on the type of the metals, and they apply for both protocols, different locations and FOV.

**Conclusions**: Metal-specific HU windows can be defined in extended HU CT scans for accurate contouring of different metals. The defined windows are protocol- and CT scanner-specific. We recommend this method be applied in the radiation oncology community to support accurate planning involving metal implants.

## P096 -

### Evaluating the usefulness and uniqueness of a recurring physics and dosimetry “lessons learned” meeting

#### Mark Zakhary^1^, Mingyao Zhu^2^, Katja Langen^2^, Sina Mossahebi^1^, Sastry Vedam^1^, Byongyong Yi^1^

##### ^1^University of Maryland School of Medicine, Radiation Oncology, Baltimore, USA ^2^Winship Cancer Institute of Emory University, Radiation Oncology, Atlanta, USA

**Background**: A regular “Lessons Learned” meeting is held between the physics and dosimetry groups at our proton center, in order to discuss planning-related issues, share experience, and improve communication between the two groups. The purpose of this study is to categorize discussion topics from the Lessons Learned meeting and identify trends, in order to assess the scope and usefulness of the meeting.

**Methods**: Topics discussed during Lessons Learned meetings taking place between January 2019 and February 2021 were compiled and classified into four categories: procedural errors, new procedures and procedural clarifications, planning discussions, and troubleshooting. Frequently recurring topics were identified, and overlap with other quality assurance or safety meetings was evaluated.

**Results**: A total of 225 topics were discussed over 31 meetings during the time period reviewed. 87 topics were categorized as procedural errors, 82 as new procedures or procedural clarifications, 32 as planning discussions, and 24 as troubleshooting. 106 topics (47%) are not germane to any other quality assurance or safety meeting at our institution. Frequently occurring topics include: material override issues (28 topics), CT calibration curve issues (12 topic), nomenclature (12 topics), beam angle selection (12 topics), improving communication within and between the physics and dosimetry groups (10 topics), and field weighting (9 topics).

**Conclusions**: The Lessons Learned meeting has consistently been a productive and informative space promoting quality improvement, particularly for delving deeply into technical topics which would not be routinely shared elsewhere. Material override issues were the most prevalent topic, and communication issues were surprisingly common.

## P097 -

### Evaluation of proton pencil beam scanning (PBS) plan quality and delivery time as a function of spot and layer spacing

#### J. Alshakhi^1^, G. Royle^2^, D. D'Souza^3^, R. Amos^2^

##### ^1^Saudi Proton Therapy Center, Medical Physics, Riyadh, Saudi Arabia ^2^Unversity College London, Medical Physics & Biomedical Engineering, London, United Kingdom ^3^University College London Hospitals NHS Foundation Trust, Radiotherapy Physics, London, United Kingdom

**Purpose/Objective:** To evaluate variable spot- and layer-spacing on proton PBS plan quality and delivery time.

**Materials and Methods:** Plans to 10x10x10cm homogeneous target calculated using Eclipse v13.5, commissioned with IBA beam data. All plans used 2.5mm calculation grid and optimized for a *Rx* of 100% ≤ *Rx* ≤ 102% to entire volume. Layer-spacing (Bragg peak 90-90% of most proximal target region) varied 3.7mm-10mm with 5mm spot-spacing. Spot-spacing varied 3mm-10mm with constant layer-spacing of 7.6mm (4 x σ of next highest energy). Numbers of spots and layers were extracted from DICOM files using a Matlab script.

For all plans, times taken for calculation and optimization were recorded, and dose homogeneity was evaluated (D_1_-D_99_, D_2_-D_98_, and D_5_-D_95_). Treatment delivery times for each plan were estimated using Eq.1: *t_i_* = (*n_i_* – 1) x *t_E_* + *n_s_* x *t_s_* [Eq.1] where: *t_i_* is estimated delivery time; *n_l_* is the number of layers; *t_E_* is layer switching time, (approx 1.4 s (averaged across all energies)); *n_s_* is number of spots; *t_s_* time to deliver a single spot.

**Conclusions:** Reducing spot- and layer-spacing in proton PBS plans increases number of spots to be delivered, resulting in improved homogeneity but increased delivery time. Layer-spacing ≥ 5mm resulted in dose inhomogeneity. Ideally, spot-spacing would vary as a function of depth to account for multiple Coulomb scattering, however, where a constant spot-spacing is required, 5mm was found to maintain homogeneity.

As compromise between plan quality and delivery time, both spot- and layer-spacing of 5mm is recommended for proton PBS plans.

## P098 -

### Evaluation of daily and accumulated proton dose for sinonasal cancer demonstrates adequate target coverage for most patients

#### R. Argota Perez^1^, M.B. Sharma^1^, U.V. Elstroem^2^, D.S. Moeller^1^, C. Grau^1,2,3^, K. Jensen^2^, A.I.S. Holm^1^, S.S. Korreman^1,2,3^

##### ^1^Aarhus University Hospital, Department of Oncology, Aarhus, Denmark ^2^Aarhus University Hospital, Danish Center for Particle Therapy, Aarhus, Denmark ^3^Aarhus University, Department of Clinical Medicine, Aarhus, Denmark

**Aim:** To evaluate accumulated dose over a full proton therapy course for sinonasal cancer (SNC) patients using daily CBCT scans.

**Materials and methods:** Twenty-four SNC patients were retrospectively replanned with IMPT. Dose was 66-68Gy/60-66Gy for primary/postoperative radiotherapy. Plans were made in Eclipse v.15.6 using 3-5 beam angles, pencil beam scanning, multi-field optimization, and 5 cm range shifter. Robustness parameters included ± 3 mm setup uncertainty in all cardinal directions and ± 3.5 % range uncertainty. To calculate daily anatomical variations, synthetic CTs (SynCT) were generated by deforming the planning CT (pCT) to the daily CBCTs in MIM v.7.0.2. Nominal plans were recalculated on the SynCTs and the resulting dose distributions were accumulated on the pCT. Acceptable target coverage was defined as V95%>99% for the high-dose CTV.

**Results:** Large variations (up to 8.8 cc) were observed for the ipsilateral maxillary filling throughout the treatment. Variations resulted in CTV underdosage for 13/24 patients for one or more of the recalculated daily doses. However, the accumulated doses resulted in clinically acceptable target coverage for 22/24 patients (Figure 1). The delivered OAR dose was very robust with respect to anatomical variations and no statistically significant differences were observed between nominal and accumulated dose. (Table 1).

**Conclusions:** Accumulated dose coverage was clinically acceptable for most patients, even when target was undercovered in daily doses. However, for the individual patient this will not be known before start of treatment. Therefore, an individualized strategy to ensure robustness towards anatomical variations occurring during the treatment of SNC is warranted.

## P099 -

### Energy layer reduction strategies for proton arc therapy

#### Cecilia Battinelli^1^, Rasmus Bokrantz^1^, Björn Andersson^1^, Erik Traneus^1^

##### ^1^RaySearch Laboratories AB, Research, Stockholm, Sweden

**Purpose**: Proton arc therapy where spots are delivered from a large number or continuum of gantry directions has been proposed as an evolution of IMPT. Because energy switching constitutes a major component of the treatment delivery time, a central problem is selecting energy layers over the arc. The study explores proton arc optimization strategies for energy layer selection.

**Methods**: We suggest four methods for energy layer selection: progressive addition of layers based on a gradient score, successive removal of layers based on their spot weights, a hybrid method that combines the previous two, and inclusion of a layer-sparsity promoting term in the objective function of the treatment plan optimization problem.

**Results**: The methods were compared to three-field IMPT pancreatic cancer cases. A comparable number of energy layers were used for arcs and fixed-field IMPT. Delivery times were computed with respect to a gantry speed of 6°/s and an energy switching time of 1 s/layer. Arc therapy led to improved sparing of OARs (Figure 1), improved objective function values (19% better on average), equivalent delivery times and similar dose-averaged LET to the PTV and OARs (Figure 2). Overall performance was best for the hybrid method.

**Conclusions**: All four methods can generate proton arc plans of equal or better quality with different computational effort. Proton arc therapy has the potential to reduce dose to OARs. We observe no noteworthy LETd differences.

## P100 -

### Develop a rotational robust optimized Spot-scanning Proton Arc (SPArc) algorithm

#### S. Chang^1^, G. Liu^1^, L. Zhao^1^, W. Zheng^1^, A. Qin^1^, D. Yan^1^, X. Li^1^, X. Ding^1^

##### ^1^Beaumont Health, Radiation Oncology Proton Therapy Center, Royal Oak, USA

**Purpose:** Develop a new rotational robust optimization SPArc algorithm (SPArc_rot_) to mitigate the dosimetric impact from patients' rotational setup error.

**Method:** The SPArc_rot_ incorporates a multi-CT robust optimization framework. Compared to the standard SPArc algorithm, the SPArc_rot_ takes into account two more CT image sets by rotating +5°, and -5° of the planning CT. Five cases from different disease sites were selected to evaluate the effectiveness of SPArc_rot_. Both SPArc and SPArc_rot_ plans were generated using the same translational robust optimization parameters. Then, both SPArc and SPArc_rot_ plans were recalculated using a series of pseudo-CT introducing different rotational setup errors (±1°,±2°,±3°,and ±5°). Dosimetric metrics such as D98%, D95% of CTV, and 3D gamma analysis were used to assess the dose distribution changes.

**Results:** The magnitudes of dosimetric perturbation in the targets due to rotational errors were significantly reduced by the SPArc_rot_ in all the cases (Figure 1, 2). The max dose uncertainties to the brainstem, optic nerves, spinal cord, and esophagus were reduced using SPArc_rot_ for brain and lung cases, respectively(Figure 1). The uncertainties of the mean dose to the OARs such as the liver and oral cavity, parotid is comparable between the two planning techniques. The gamma pass-rate (3%/3mm) was significantly improved for CTV of all the disease sites in SPArc_rot_ plan group.

**Conclusion:** SPArc_rot_ could effectively mitigate the max dose perturbations in the adjunct OARs which is critical to the series organ. This new algorithm could be implemented through the multi-CT robust optimization framework using the existing commercial TPS.

## P101 -

### Dual Energy CT Imaging from Single Energy CT using Deep Learning for Proton Radiation Therapy

#### Serdar Charyyev^1^, Tonghe Wang^1^, Yang Lei^1^, Beth Ghavidel^1^, Liyong Lin^1^, Jonathan Beitler^1^, Mark McDonald^1^, Jeffrey Bradley^1^, Jun Zhou^1^, Tian Liu^1^, Xiaofeng Yang^1^

##### ^1^Emory University, Radiation Oncology, Atlanta, USA

Purpose: Dual-energy CT (DECT) has been shown to derive a stopping-power-ratio (SPR) map with higher accuracy than conventional single-energy CT (SECT) by obtaining energy dependence of photon interactions. However, DECT is not as widely implemented as SECT in proton radiotherapy simulation. This work presents a learning-based method to synthesize DECT images from SECT for proton radiotherapy. Methods: Proposed method uses a residual attention cycle-consistent generative adversarial network. Residual blocks with attention gates were used to force the model to focus on the difference between DECT maps and SECT images. Cycle-consistent generative adversarial networks were used to let the SECT-to-DECT mapping be close to a one-to-one mapping by introducing an inverse DECT-to-SECT mapping. To evaluate the accuracy of the method, we retrospectively investigated 30 head-and-neck cancer patients with both DECT and SECT scans available. The high and low energy CT images acquired from DECT acted as learning targets in the training process and were evaluated against results from the proposed method using a leave-one-out cross-validation strategy. Results: The synthetic DECT images showed an average mean absolute error around 30 Hounsfield Units across the whole-body volume. The corresponding SPR maps generated from synthetic DECT showed an average normalized mean square error of about 1% and have significantly reduced noise levels and artifacts than those from the original DECT. Conclusion: We proposed a novel deep-learning-based approach to synthesize DECT from SECT. These results strongly indicate the high accuracy of synthetic DECT image by our method and show its potential feasibility for proton radiotherapy.

## P102 -

### Energy layer and spot spacing for pencil beam scanning proton treatment planning

#### Yu Chen ^1^

##### ^1^MedStar Georgetown University Hospital, Radiation Medicine, Washington, USA

**Purpose:** To investigate how to optimally set “Energy layer spacing” and “Spot spacing” parameters in RayStation treatment planning system for Hyperscan pencil beam scanning proton treatment plan.

**Method:** Hyperscan machine can produce proton beams of 160 discrete energies with a range step size of 0.2cm, resulting in possible energy layers with spacing of 0.4, 0.6, 0.8 cm, etc. Integral depth doses and spot size data, and the QA results for patient treatment plans were analyzed.

**Results:** The treatment plans passed QA have the number of energy layers < 30, the number of spots < 2500, and the average MU/spot > 1 for each field. Hyperscan machine demonstrated an unchanged Bragg peak width of 0.53cm at 90%-90%, indicating an adequate energy layer spacing of 0.4 or 0.6 cm by setting “constant 0.45-0.8 cm in water” (corresponding to “automatic with scale 0.53-0.94”). Such settings can be adjusted if required one more distal layer to cover 3.5% range uncertainty (Fig. 1). The spot spacing by setting “automatic with scale” varies with energy and air gap and is approximately proportional to spot sigma with scale 0.95 equivalent to 1 sigma (Fig. 2). Therefore, spot spacing scale 0.87 shall be adequate such that two 0.92 sigma away Gaussians would overlap at 90%. Due to a large amount of spots filtered out by a low MU limit, actual spot spacing scale 0.5-0.8 is used.

**Conclusion:** The optimal settings of energy layer and spot spacing are suggested in planning with adequate quality and high possibility to pass QA.

## P103 -

### Beam angle optimization for double-scattering proton delivery techniques using Eclipse API and convolutional neural network

#### Wonjoong Cheon^1^, Sang Hee Ahn^1^, Seonghoon Jeong^1^, Se Byeong Lee^1^, Dongho Shin^1^, Young Kyung Lim^1^, Jong Hwi Jeong^1^, Sang Hee Youn^1^, Sung Uk Lee^1^, Sung Ho Moon^1^, Tae Hyun Kim^1^, Haksoo Kim^1^

##### ^1^National cancer cetnter, Proton Therapy Center, Goyang-si, Korea Republic of

To automatically identify optimal beam angles for proton therapy configured with the double-scattering technique, a beam angle optimization network based on a convolutional-neural-network (CNN): BAODS-Net is proposed. Fifty liver plans were used for training BAODS-Net. An in-house program based on a treatment planning system API (Eclipse) was developed. Twenty-five rays on the beam's eye view were determined per angle to generate input data. A ray collects nine features: a normalized Hounsfield unit and position information of eight structures from 0° to 359° with a step size of two-degrees (Fig.-1). The outputs were a set of beam angle scores (S_beam_) ranging from 0° to 359°. The score of an angle was a continuous value from 0 to 1 point, which was ranking information. In the training process, the layers of BAODS-Net were optimized to suggest an optimal S_beam._ Finally, we compared the performances of three types of loss functions and performed *K*-fold cross-validation (*K*=5) to evaluate the plan qualities of deep-learning, equi-spaced, and clinic plans. The smooth-L1 loss showed the best optimization performance among L1, L2, and smooth-L1 loss. In terms of the plan quality, the average relative differences of the mean dose to the clinic plan for organs at risk were −2.18% and 96.86% for the proposed deep-learning method, equi-spaced method, respectively (Fig.-2). A deep-learning-based BAO method for proton double-scattering treatments was developed and verified. Using Eclipse API and BAODS-Net, a plan with clinically acceptable quality was created within 5min. **Acknowledgement**: This research was supported by National-Research-Foundation of Korean-National-Cancer-Center Fund:(2010400-1).

## P104 -

### Proton therapy X-ray CT calibration by proton tomography

#### C. Civinini^1^, M. Bruzzi^1,2^, F. Fracchiolla^3,4^, S. Lorentini^3,4^, R. Righetto^3,4^, M. Scaringella^1^, M. Scarpa^4,5^, F. Tommasino^4,5^, P. Farace^3,4^

##### ^1^INFN, Florence, Sesto Fiorentino, Italy ^2^Università degli Studi di Firenze, Dipartimento di Fisica e Astronomia, Florence, Italy ^3^Azienda Provinciale per i Servizi Sanitari APSS, Protontherapy Unit- Hospital of Trento, Trento, Italy ^4^INFN, Trento Institute for Fundamental Physics and Applications, Trento, Italy ^5^Università di Trento, Dipartimento di Fisica, Trento, Italy

In the recent past, INFN research projects have studied the feasibility of proton Computed Tomography (pCT) and a prototype has been successfully built and tested with a proton beam at the Trento Proton Therapy Centre. Proton tomographies of test phantoms (Fig.1) have been reconstructed using this apparatus allowing a direct measurement of their relative stopping power (SPR) with an accuracy of about 1% (2020, *Phys. Med. Biol*. 65 225012).

The INFN pCT system will be then used for a possible clinic application as a tool to improve treatment accuracy in proton therapy. In particular, we aim to introduce a new calibration method for the x-ray CT systems used in proton treatment planning procedure. This calibration has been recently described (2021, *Med. Phys*. https://doi.org/10.1002/mp.14698) and will be investigated within an INFN funded project. A set of biological phantoms will be prepared and the pCT apparatus used to extract their SPR maps then associated to the x-CT measured Hounsfield Units (HU) of the same objects. The calibration function, a SPR-HUs look-up table, could then be derived. Once a stable biological phantom will be available, this calibration procedure might be extended to PT centres not equipped with a pCT system. This could be done by shipping the phantom to the remote centre for an acquisition with the x-CT systems to be calibrated, while having the corresponding SPR maps already reconstructed from pCT. The SPR map and the x-CT HU will be then processed and a calibration for the remote x-CT system can be extracted.

## P105 -

### Different planning considerations for breast cancer in proton therapy for free and breath hold anatomies. Is there any golden rule?

#### Katarzyna Czerska^1^, Piotr Winczura^2^, Julianna Wejs-Maternik^2^, Pawel Olko^3^, Renata Kopec^1^

##### ^1^Institute of Nuclear Physics PAN, Cyclotron Centre Bronowice, Krakow, Poland ^2^Radiotherapy Center Elblag, Radiotherapy, Elblag, Poland ^3^Institute of Nuclear Physics PAN, Applied Physics Division, Krakow, Poland

**Purpose**: Moving targets remain one of the most challenging problems in proton radiotherapy. Although breast cancer is not considered as a classical 4D indication, special attention must be paid to methodology of treatment planning. In this work comparative treatment plans using different approaches were performed in order to study their sensitivity against the motion.

**Materials and methods**: Five left-sided breast cancer patients were planned with an Intensity Modulated Proton Technique (IMPT) on free (FB) and breath-hold (BH) anatomies. We incorporated five beam arrangements, that might be possibly chosen for such cases: one beam only, three beams, three layouts for two beams (en face + anterior or lateral beam). Finally the robustness analysis was performed (2mm/3.5%).

**Results**: We have chosen for comparison CTV, left lung, heart and left anterior descending artery (LAD). Acceptable target coverage was fulfilled in all plans. Although the most significant difference was seen in case of Dmax0.2cm3, LAD and two en face beams were able to decrease the dose maximally. The worst dosimetric outcome and the highest low-dose region for organs at risk were obtained for the scenario with lateral beam.

**Conclusions**: Comparison of different beam arrangements might be helpful at the stage of treatment planning for breast cancer. It shows which layout would be the least sensitive to any changes that might occur at the beam path and which of them would cause the least severe dosimetric differences.

**Acknowledgements**: KC has been partly supported by the EU Project POWR.03.02.00-00-I004/16.

## P106 -

### Spot scanning Proton Arc therapy (SPArc) with optimized energy layer selection settings reduces toxicity for head and neck cancer patients.

#### Bas De Jong^1^, Erik W. Korevaar^1^, Cecilia Battinelli^2^, Guillaume Janssens^3^, Johannes A. Langendijk^1^, Stefan Both^1^

##### ^1^University Medical Center Groningen- University of Groningen, Department of Radiation Oncology, Groningen, Netherlands ^2^RaySearch Laboratories AB, Research Department, Stockholm, Sweden ^3^Ion Beam Applications SA, Reseach, Louvain-la-Neuve, Belgium

**Introduction:** SPArc technology has recently shown dosimetrical gains for diverse indications. A relationship exists between plan quality, number of energy layers (ELs) -and beams in a SPArc plan. This work aims to investigate the number of ELs and beams required for an optimal plan quality and impact on toxicity for oropharyngeal cancer patients selected for IMPT based on the Dutch model-based approach (MBA).

**Methods:** The RaySearch EL algorithm iteratively selects ELs from beams equidistantly spaced over a 360 degree arc (figure 1). The number of ELs and beams were varied, to determine the relationship with the objective function indicating plan quality. SPArc plans, robust to range and setup uncertainty (3%, 3mm) with 20 beams and 360 ELs, were generated for ten oropharyngeal cancer patients previously treated with IMPT according to the MBA. SPArc and clinical IMPT plans were compared in terms of integral dose and NTCP for dysphagia and xerostomia, while target coverage was robust.

**Results:** Figure 2 illustrates the dependence of the objective function value on number of ELs and beams. We found that 360 ELs distributed over 20 beams generated near optimal quality SPArc plans. Relative to corresponding IMPT plans, an average reduction of 21±4% in integral dose was observed. The average NTCP for grade≥2 and grade≥3 dysphagia decreased with 3.7±2.5% and 0.9±0.6%, respectively, while the average NTCP for grade≥2 and grade≥3 xerostomia decreased with 4.2±2.2% and 1.4±1.0%, respectively.

**Conclusions:** SPArc demonstrates potential to further reduce toxicity relative to IMPT when 360 ELs and 20 beams are employed.

## P107 -

### Fast Treatment Planning System for protons and carbon ions therapy with a fast-MC code (FRED) based on GPU technology

#### Micol De Simoni^1,2^, Giuseppe Battistoni^3^, Patrizia De Maria^4^, Marta Fischetti^2,5^, Gaia Franciosini^1,2^, Michela Marafini^2,6^, Vincenzo Patera^2,5^, Alessio Sarti^2,5^, Adalberto Sciubba^5,7^, Marco Toppi^5,7^, Giacomo Traini^2,6^, Antonio Trigilio^1,2^, Angelo Schiavi^2,5^

##### ^1^La Sapienza- University of Rome, Physics, Rome, Italy ^2^INFN Istituto Nazionale Fisica Nucleare, Roma1, Rome, Italy ^3^INFN Istituto Nazionale Fisica Nucleare, Milano, Milan, Italy ^4^La Sapienza- University of Rome, Post-graduate School in Medical Physics- Department of Medico-Surgical Sciences and Biotechnologies, Rome, Italy ^5^La Sapienza- University of Rome, SBAI Scienze di Base e Applicate per l'Ingegneria, Rome, Italy ^6^CREF Museo Storico della Fisica e Centro Studi e Ricerche “E. Fermi”, Physics, Rome, Italy ^7^INFN Istituto Nazionale Fisica Nucleare, Frascati, Frascati, Italy

The advent of general programming GPU has prompted the development of MC codes that can dramatically reduce the treatment plan recalculation time comparing to standard MC codes based on CPU hardware. The possibility to evaluate a complete TPS within minutes, instead of hours, paves the way for many clinical applications where the time-factor is important. FRED (Fast paRticle thErapy Dose evaluator) is a software that exploits the GPU power to recalculate and optimise ion beam Treatment Plans System (TPS). The main goal in developing the FRED physics model is to balance accuracy, calculation time and GPU execution guidelines. Nowadays FRED is already used as a quality assurance tool in the proton clinical center of Maastricht and Krakow and as a research tool at several clinical and research centers in Europe (Krakow, Trento, Maastricht, Lyon and PSI). Lately, the code has been updated with the insertion of the interaction of carbon ions with matter. The implemented fragmentation model is a phenomenological model mainly based on extrapolation of the carbon fragmentation data taken at Ganil (laboratory of CAEN, France). The model has been tested against the full-MC code FLUKA, commonly used in particle therapy, and with few experiments found in the literature. In the next future, the accuracy of FRED dose recalculation will be compared with the CNAO TPS for carbon therapy to achieve clinical validation. In this contribution, the new FRED data-driven model of carbon ion will be presented as well as the updates in the proton's model.

## P109 -

### Bragg-peak degradation caused by heterogeneous materials – A CT histogram analysis as a predictor for the modulation power

#### Veronika Flatten^1,2,3^, Jan Burg^1,3^, Matthias Witt^1,2,3^, Pedro Fragoso Costa^4,5^, Jörg Wulff^4,6,7^, Christian Bäumer^4,6,7,8^, Beate Timmermann^4,6,7^, Uli Weber^9^, Rita Engenhart-Cabillic^1,2^, Klemens Zink^1,2,3^, Kilian-Simon Baumann^1,2,3^

##### ^1^University Medical Center Giessen-Marburg, Department of Radiotherapy and Radiooncology, Marburg, Germany ^2^MIT, Marburg Ion-Beam Therapy Center, Marburg, Germany ^3^University of Applied Sciences, Institute of Medical Physics and Radiation Protection, Giessen, Germany ^4^WTZ, West German Cancer Center, Essen, Germany ^5^University Hospital Essen, Clinic for Nuclear Medicine, Essen, Germany ^6^WPE, West Proton Therapy Center, Essen, Germany ^7^University Hospital Essen, Clinic for Particle Therapy, Essen, Germany ^8^TU Dortmund University, Faculty of Physics, Dortmund, Germany ^9^GSI Helmholtzzentrum für Schwerionenforschung, Biophysics Division, Darmstadt, Germany

The fine, sub-millimeter sized structure of lung tissue causes a degradation of the Bragg peak in particle therapy. This can lead to an underdose in the target volume in general and an overdose distal to the target. The quantity describing the materials' property predicting this degradation is the so-called modulation power (P_mod_). This micro structure of lung tissue or other heterogeneous tissue cannot be resolved in treatment-planning CTs. The present work investigates an estimation of the modulation power based on CT-histograms. Different modulating materials were scanned with a small-animal CT (voxelsize: 0.1mm^3^) and with a clinical CT with a 0.6mm slice thickness. The Bragg-Peak degradation was measured in a clinical proton beam and the modulation power was determined by means of deconvolution. Histograms of two materials (LN300 and polystyrene) for both CT resolutions are presented in figure 1. A model is established, which calculates the modulation power via the mean and the width of the clinical CT histogram. The calculated modulation power was then compared to the modulation power obtained in the proton beam measurement. The proportionality is shown in figure 2. For modulation powers in the clinical range (0.1mm - 0.45mm), the obtained values are in good agreement with the measured modulation power values. The model was then transferred to measurements and CT scans of porcine lung samples, allowing an estimation of the modulation power without in beam measurements (see Fig.2).

## P110 -

### Measurements of 16O fragmentation cross sections on C target with the FOOT apparatus

#### Marco Toppi^1,2^, Gaia Franciosini^1^

##### ^1^La Sapienza, Scienze di Base e Applicate per l'Ingegneria, Rome, Italy ^2^INFN - Istituto Nazionale di Fisica Nucleare, LNF - Laboratori Nazionali di Frascati, Frascati, Italy

In particle therapy, nuclear interactions of the beam with the patient's body causes fragmentation of both the projectile and target nuclei. In treatments with protons, target fragmentation generates short range secondary particles along the beam path, that may deposit a non-negligible dose especially in the entry channel. On the other hand, in treatments with heavy ions, such as C or other potential ions of interest, like He or O, the main concern is long range fragments produced by projectile fragmentation, that release the dose in the healthy tissues downstream of the tumor volume. Fragmentation processes need to be carefully taken into account when planning a treatment, in order to keep the dose accuracy within the recommended 3% of tolerance level. The assessment of the impact that these processes have on the released dose is currently very limited from the lack of experimental data, especially for the relevant fragmentation cross sections. For this reason, treatment plans are not yet able to include the fragmentation contribution to the dose map with the required accuracy. The FOOT (FragmentatiOn Of Target) collaboration designed an experiment to fill this gap in experimental data, aiming the measurement of the differential cross sections of interest. In this contribution, an overview of the FOOT experiment, including the present detector design and the expected performances will be discussed. In addition, preliminary results of a 400 MeV/u ^16^O beam impinging on a carbon target will be presented.

## P111 -

### Can deformable registration between CT and CBCT reliably predict a need for plan adaptation in proton therapy of H&N cancer?

#### Joanna Gora^1^, Angelica De Leon^1^, Gabriele Kragl^2^, Antonio Carlino^1^, Markus Stock^1^

##### ^1^MedAustron Ion Therapy Center, Medical Department, Wiener Neustadt, Austria ^2^Ordensklinikum Linz GmbH- Barmherzige Schwestern, Strahlentherapie, Linz, Austria

With the recent advances in deformable registration techniques, the ability of replacing control CTs (cCT) with CBCTs, as a trigger deciding about treatment plan adaptation for H&N cancer patients, was explored. Decreased cCTs number would decrease overall patient dose and improve the efficiency of the clinical workflow. The planning CT(pCT) was deformably registered to the cCT and CBCT (same day acquisition), using a hybrid algorithm implemented in RayStationV8B(RaySearch Laboratories). Detailed visual and quantitative comparison between both registrations was performed. Additionally, original dose distribution was recalculated on the deformed images to evaluate differences in planning goals and dose statistics. Overall results between both registrations looked very promising. DVF comparison revealed similar absolute lengths (within 2mm) and no visual irregularities(fig1). Dose recomputation and dose statistics on the deformed images for important OARs and targets were comparable (within 1Gy). However, after the detailed inspection, 3 regions, where the algorithm was struggling to perform well, were identified: oral and nose cavities, metal artefacts and skull bones. In those areas local dose differences up to 8Gy(different cavity fillings), and up to 4Gy(artifacts) were observed(fig2). For this patient CBCT would have triggered the same decision as cCT. However, when replacing a cCT with CBCT one needs to be careful. Systematic errors caused by the bone representation, metal and cavity fillings make algorithm very sensitive, which could cause errors in judgment. Finally, the presented workflow, is not entirely implemented in the TPS and therefore outsourcing to external software's is still needed, which would affect the clinical workflow.

## P112 -

### Multi-centric study on variable RBE dose calculations in European proton therapy institutions

#### Christian Hahn^1,2,3,4^, Jakob Ödén^5^, Alexandru Dasu^6,7^, Anne Vestergaard^8^, Jesper Folsted Kallehauge^8^, Claudia Pardi^9^, Faiza Bourhaleb^9^, Amélia Leite^10^, Ludovic de Marzi^10,11^, Edward Smith^12,13^, Adam Aitkenhead^12,13^, Michael Merchant^12,13^, Karen Kirkby^12,13^, Esther G. C. Troost^2,3,4,14,15^, Armin Lühr^1,2,4,15^

##### ^1^Technical University Dortmund- Faculty of Physics, Medical Physics and Radiotherapy, Dortmund, Germany ^2^German Cancer Consortium DKTK, Partner Site Dresden- and German Cancer Research Center DKFZ, Heidelberg, Germany ^3^Technische Universität Dresden, Department of Radiotherapy and Radiation Oncology- Faculty of Medicine and University Hospital Carl Gustav Carus, Dresden, Germany ^4^Technische Universität Dresden- Helmholtz-Zentrum Dresden-Rossendorf- Dresden- Germany, OncoRay – National Center for Radiation Research in Oncology- Faculty of Medicine and University Hospital Carl Gustav Carus, Dresden, Germany ^5^RaySearch Laboratories AB, RaySearch Laboratories AB, Stockholm, Sweden ^6^The Skandion Clinic, The Skandion Clinic, Uppsala, Sweden ^7^Uppsala University, Medical Radiation Sciences- Department of Immunology- Genetics and Pathology, Uppsala, Sweden ^8^Aarhus University Hospital, Danish Centre for Particle Therapy, Aarhus, Denmark ^9^I-SEE, Internet-Simulation Evaluation Envision, Torino, Italy ^10^Institut Curie- PSL Research University, Radiation Oncology Department- Proton Therapy Centre- Centre Universitaire, 91898 Orsay, France ^11^Institut Curie- PSL Research University- University Paris Saclay, Inserm LITO, 91898 Orsay, France ^12^The Christie NHS Foundation Trust, Christie Medical Physics and Engineering, Manchester, United Kingdom ^13^The University of Manchester, Division of Cancer Sciences- School of Medical Sciences- Faculty of Biology- Medicine and Health, Manchester, United Kingdom ^14^National Center for Tumor Diseases NCT- Partner Site Dresden- Germany: German Cancer Research Center DKFZ- Heidelberg- Germany, Faculty of Medicine and University Hospital Carl Gustav Carus- Technische Universität Dresden- Dresden- Germany- and- Helmholtz Association / Helmholtz-Zentrum Dresden - Rossendorf HZDR, Dresden, Germany ^15^Helmholtz-Zentrum Dresden-Rossendorf, Institute of Radiooncology – OncoRay, Dresden, Germany

Recent clinical reports show varying relative biological effectiveness (RBE) in proton therapy (PT). While a constant RBE ensures comparability in prescription and outcome reporting among institutions, a standard for clinical variable RBE calculations is lacking. This study quantifies differences among European PT institutions in their currently applied variable RBE calculations. Six European PT institutions generated clinically acceptable robust pencil-beam-scanning treatment plans for identical patients originating from five sites: brain, base-of-skull, head-and-neck, pancreas and prostate. Centres used their beam model, treatment planning software and an RBE of 1.1 (*D*_1.1_). Each centre recalculated the corresponding variable RBE weighted dose (*D_RBE_*) according to their local procedure, including choice of variable RBE model and parameters. Here, dosimetric parameters to targets (CTV) and OARs of three centres were analysed for the brain tumour patient. The inter-centre differences in *D*_1.1_ for all CTV dose parameters and the near-maximum dose of adjacent OARs (chiasm, brainstem) were below 1.4Gy(RBE) and 2.4Gy(RBE), respectively. The centre-specific variable RBE models consistently predicted overdosage in adjacent OARs and substantial deviations in *D*_RBE_ in the CTV (Fig.1). While inter-institutional dose differences remained comparable in OARs using *D_RBE_* instead of *D*_1.1_, they increased by 4-5Gy(RBE) in the CTV. Differences in *D*_RBE_ distributions between institutions reduced to those in *D*_1.1_ when all centres used the same variable RBE model (Fig.2). Variable proton RBE calculations are readily available in numerous European proton centres and provide additional information for treatment plan safety. Standardizing variable RBE calculations would enable consistent outcome reporting and facilitate variable RBE optimization in the future.

## P113 -

### Using the full potential of Bragg peak in FLASH proton therapy

#### Lucian Hotoiu^1^, Arnaud Pin^1^, Sylvain Deffet^2^, Edmond Sterpin^2,3^, Rudi Labarbe^1^

##### ^1^Ion Beam Applications S.A., Research, chemin du Cyclotron 3 1348 Louvain-la-Neuve, Belgium ^2^Universite catholique de Louvain, Molecular Imaging Radiotherapy and Oncology MIRO, Louvain-la-Neuve, Belgium ^3^KULeuven, Laboratory of Experimental Radiotherapy Department of Oncology Faculty of Medicine, Leuven, Belgium

FLASH irradiation implies delivering the treatment dose at high dose-rates (typically > 40 Gy/s) to elicit sparing in irradiated heathy tissue. To fulfil the FLASH time constraints and achieve high dose-rates in proton PBS irradiation, a working approach is scanning the pencil-beam in a monoenergetic delivery, whilst ensuring the target coverage within the generated Spread-Out-Bragg-Peak (SOBP). To obtain the SOBP in one shot, we propose a delivery technique unraveling the full potential of proton Bragg-peak for high dose-rate FLASH irradiations, alleviating the limitations in the current shoot-through, transmission-FLASH approach that negates the advantage of the Bragg-peak. The solution consists in passing the proton pencil-beam at the highest cyclotron energy through a range shifter setting the beam maximum range and through a spatially-non-uniform *ridge filter* (the “hedgehog”)**.** The ridge filter base height controls the distal modulation while the height of its spikes defines the proximal modulation depth at each position, giving a conformal dose distribution over the non-uniform CTV surfaces, as shown in Figure 2. The spike shapes determine the SOBP plateau flatness. The ridge filter and range shifter are patient and field specific, responsible in generating the SOBP covering the target volume. Its design is based on the weight of each beamlet in the field, by optimizing a standard IMPT plan using an analytical algorithm in *MIROpt* (http://openmiropt.org/), alongside the *MCsquare* dose engine (http://www.openmcsquare.org/). A modified *MIROpt version* optimizes the spot scanning trajectory and computes the percentile dose-rate at each CT voxel. Acknowledgements: This work is funded the Walloon Region, grant No.8341.

## P114 -

### Quantitative assessment of 3D dose rate for proton PBS FLASH radiotherapy and its application for lung hypofractionation treatment planning

#### Minglei Kang^1^, Wei Shouyi^1^, J Isabelle Choi^1^, Charles B Simone- II^1^, Haibo Lin^1^

##### ^1^New York Proton Center, Radiation Oncology, New York, USA

**Purpose**: To quantitatively assess target and organs-at-risk(OAR) dose rate based on 3 proposed proton PBS dose rate metrics and study FLASH intensity-modulated proton therapy(IMPT) treatment planning using transmission beams.

**Materials and Methods:** An in-house FLASH planning platform was developed to optimize transmission/shoot-through plans for 9 consecutive lung cancer patients previously planned with proton SBRT(Fig1). Dose and dose rate calculation codes were developed to quantify 3-type of dose rate calculation methods(dose-averaged-dose-rate(DADR), average-dose-rate(ADR), and dose-threshold-dose-rate(DTDR)) based on both phantom and patient treatment plans. Minimum MU/spot of 100 and 400 were both used in optimizing two fractionations, 34Gy in 1-fraction and 45Gy in 3-fraction(Fig2).

**Results:** OAR sparing and target coverage can be optimized with good uniformity(hotspot<110% of Rx). ADR, accounting for the spot dwelling and scanning time, gives the lowest dose rate; DTDR, not considering time but using a dose-threshold, gives an intermediate dose rate; DADR gives the highest dose rate without considering time or dose-threshold. All 3-dose-rate attenuate along the beam direction, and the highest dose rate regions often occur on the field edge for ADR and DTDR, whereas DADR achieves superior dose rate uniformity. Both ADR and DTDR have minimal MU/spot and fraction-dose dependence under a spot-peak-dose-rate(SPDR)<670Gy/s.

**Conclusion:** The large variation of dose rate calculated using 3 different methods indicates the uncertainties in tissue dose rate assessment. This is the first attempt to study the impact of varying dose rate modes, and more investigation and evidence studying of PBS parameters are needed to explore the correlation between FLASH efficacy and dose rate metrics.

## P115 -

### Field numbers and beam angles selection in Breath-Hold Intensity Modulated Proton Therapy (BH-IMPT) for Liver Stereotactic Body Radiation Therapy (SBRT).

#### Mintra Keawsamur^1^, Kanokphorn Thonglert^1^, Taweap Sanghangthum^1^, Petch Alisanant^1^, Napapat Amornwichet^1^, Sivalee Suriyapee^1^, Chonlakiet Khorprasert^1^

##### ^1^HRH Princess Maha Chakri Sirindhorn proton center- King Chulalongkorn Memorial Hospital- Thai red cross society, Faculty of medicine- Chulalongkorn University, Bangkok, Thailand

The field numbers and beam angles selection are the specific requirements that accounted for the target coverage and organs at risk (OARs) sparing. This research aims to compare the dosimetric performance of Breath-Hold Intensity Modulated Proton Therapy (BH-IMPT) plans generated with eight plans of fields number and beam angle directions in various tumor locations for Liver SBRT patient. The plans involved one field, two fields with an angular separation of 30, 45, 60, 90 and 180-degree between beams, and three fields with an angular separation of 50 and 90-degree between beams. The robust optimization was implemented. The best plan was selected in concerning of conformity, dose to liver, skin, and OARs. The two fields with an angular separation of 30 to 45-degree between beams are the best for sparing the normal liver, skin, and chest wall when the tumor is located in a peripheral area. However, the tumor located < 4 cm from skin, three fields with an angular separation of 50 achieved better dose reduction to skin and chest wall, while resulted similar outcomes in term of normal liver sparing to two field directions. The central tumor is often surrounded by the normal liver. Limited number of fields achieved low mean liver dose. Our results suggested 0 and 180-degree beams produced the least mean liver dose. This research guided appropriate beam directions and angles selection for BH-IMPT for Liver SBRT with regard to conformity and the least dose to liver, skin, and OARs.

## P116 -

### Dosimetric comparison of six different whole breast irradiation techniques in supine and prone position: 3DCRT, VMAT, IMPT, PAT, IMCT, CAT

#### Dong Wook Kim^1^, Hojin Kim^1^, Chea-Seon Hong^1^, Jinsung Kim^1^

##### ^1^Yonsei Cancer Center, Radiation Oncology, Seoul, Korea Republic of

**Purpose:** The purpose of this study is to compare the dose characteristics of six different whole breast irradiation (WBI) techniques in supine and prone position: three-dimensional radiation therapy (3D-CRT), volumetric arc therapy (VMAT), intensity-modulated proton therapy (IMPT), proton arc therapy (PAT), intensity-modulated carbon therapy (IMCT), carbon arc therapy (CAT).

**Methods:** For 14 patients, the plans were developed with six techniques both supine and prone positions. The prescribed dose (PD) was 50.4 Gy in 28 fractions, meeting the PTV criterion (V95=100). Robust plan has been carried out with setup (0.5 cm) and range (3%) uncertainty by RayS RayStation^TM^ planning system. Conformity, homogeneity and coverage were used to evaluate the dose to the PTV. The volume percentage receiving 10% (V10) and 5% (V5) of PD and mean dose to organs at risk (OARs) was evaluated.

**Results:** For PTV coverage, PAT was better than other in spite of all six EB-WBI techniques showed acceptable coverage. For OARs, proton and carbon plans were significantly lower than X-ray plans. V10% and V5% in left-side breast cancer (N=7) was relatively higher with 3D-CRT and VMAT in prone position. For proton and carbon plans, there are no significant dosimetric differences for the patient position and irradiation techniques.

**Conclusion:** All six WBI techniques showed acceptable coverage of the PTV. Relatively, unlike x-ray, which could spare more OAR doses in the prone position, proton/carbon beam plan showed similar results in the supine and prone positions. For proton and carbon plan, we could not find distinguished dosimetric advantage in arc plan for whole breast irradiation.

## P117 -

### A quantitative model to identify promising planning strategies for FLASH using scanned proton therapy

#### Miriam Krieger^1,2^, Steven van de Water^2^, Michael M Folkerts^3^, Alejandro Mazal^4^, Silvia Fabiano^2,5,6^, Nicola Bizzocchi^2^, Damien C Weber^2,5,7^, Sairos Safai^2^, Antony J Lomax^2,6^

##### ^1^Varian Medical Systems International AG, Varian Proton Solutions, Villigen PSI, Switzerland ^2^Paul Scherrer Institute, Center for Proton Therapy, Villigen PSI, Switzerland ^3^Varian Medical Systems Inc., Varian Proton Solutions, Palo Alto, USA ^4^Quironsalud, Centro de Protonterapia, Madrid, Spain ^5^University Hospital Zurich, Department of Radiation Oncology, Zurich, Switzerland ^6^ETH Zurich, Department of Physics, Zurich, Switzerland ^7^Inselspital Bern, Department of Radiation Oncology, Bern, Switzerland

**Purpose**: To quantify the potential FLASH effect for various pencil beam scanning proton therapy planning scenarios using a phenomenological FLASH effectiveness model.

**MaterialsandMethods**: In our phenomenological model, FLASH is triggered for every dose contribution that is >=5Gy and has a dose-rate >=40Gys, and it persists for any subsequently delivered voxel dose (regardless of dose or dose-rate) for 200ms. If triggered, a FLASH effectiveness of 0.67 (33% dose sparing) is assumed. Treatment scenarios were simulated for a Varian ProBeam machine with cyclotron currents of 800nA. Two patients from each of five different sites were simulated, each planned with a single fraction dose of 22.3Gy. Eight delivery scenarios were studied: upstream degrader vs. downstream range-shifter vs. transmission (i.e., no energy modulation), each for single or multi-field plans. In addition, multi-field IMPT plans, optimised to maximise dose heterogeneity within each field, were studied. For all scenarios, FLASH was quantified in terms of its effect on normal tissue integral dose in relation to the non-FLASH physical dose.

**Results**: FLASH was most effective for single-field plans, transmission deliveries and/or maximised field-dose heterogeneity (Figure 1). FLASH effectiveness strongly depended on fraction dose and dose threshold, while being largely independent of the persistence time (Figure 2).

**Conclusion**: Our model predicted that single-field transmission plans with high fraction doses could best achieve meaningful FLASH effects. As a result, the dose threshold may be an important limiting factor for FLASH. Future work to validate our model assumptions is required before clinical applicability can be ascertained.

**Disclosures**: MK and MMF are Varian employees.

## P118 -

### A direct machine-specific parameters incorporated Spot-scanning Proton Arc (SPArc) algorithm

#### G. Liu^1,2^, L. Zhao^1^, D. Yan^1^, X. Li^1^, X. Ding^1^

##### ^1^Beaumont Health System, Radiation Oncology, Royal oak, USA ^2^Union Hospital Tongji Medical College Huazhong University of Science and Technology, Cancer Center, Wuhan, China

**Purpose:** To address the challenges of generating a deliverable and efficient spot-scanning proton arc(SPArc) plan for a proton therapy system. We developed a novel SPArc optimization algorithm(SPArc_DMSP_) by directly incorporating machine-specific parameters such as mechanical constraints and delivery sequence.

**Method and Material:** A SPArc delivery sequence model(DSM_arc_) was built based on the machine-specific parameters of the prototype arc delivery system IBA ProteusONE®. The SPArc_DMSP_ resamples and adjusts each control point's delivery speed based on the DSM_arc_ calculation through the iterative approach(Fig1(a)). Users could set the expected arc delivery time and gantry max acceleration as a mechanical constraint during the SPArc_DMSP_ optimization. Four cases (brain, liver, head neck, liver, and lung cancer) were selected to test SPArc_DMSP_. Two kinds of SPArc plans were generated using the same planning objective functions:(1)SPArc_DMSP_ plan meeting the maximum allowable gantry acceleration speed(0.6deg/s^2^);(2)SPArc_DMSP-user-speed_ plan with a user pre-defined delivery time and acceleration speed<0.1deg/s^2^. Arc delivery sequence such as gantry speed, delivery time was simulated based on the DSM_arc_ and was compared.

**Results:** With a similar objective value, number of energy layers, and spots, both SPArc_DMSP_ and SPArc_DMSP-user-speed_ plans could be delivered continuously within the ±1 degree tolerance window(Table1). The SPArc_DMSP-user-speed_ plan could minimize the gantry momentum change based on users' preference (Fig1(b)and2).

**Conclusions:** For the first time, the clinical users could generate a SPArc plan by directly optimize the arc treatment speed and momentum changes of the gantry. This work paved the roadmap for the clinical implementation of proton arc therapy in the treatment planning system.

## P119 -

### Interactive dose modification: a novel approach to proton therapy treatment planning

#### M. Lowe ^1^

##### ^1^The Christie NHS Foundation Trust, Christie Medical Physics and Engineering, Manchester, United Kingdom

A novel approach to treatment planning for spot-scanning proton therapy is presented by which the user can interact intuitively with the dose distribution to modify the treatment plan. In this proof-of-principle implementation, the CT and dose distribution are displayed using standalone software. The user can left/right click or scroll to increase or decrease dose to any voxel. This is achieved by modification of spot weights. Spot weights are modified in proportion to their contribution to the selected point using an influence matrix describing the contribution of each spot to each voxel. In this implementation, the influence matrix is derived from a treatment planning system (TPS) using scripting, though alternative methods, or integration with the TPS, would enable this to be quicker. Subsequent interaction with the dose distribution is quick. Using a consumer laptop (Intel i7-10750H CPU), the dose distribution is updated in <0.5 s (with a resolution of <1x1x1 mm). Minimum spot weights are accounted for on the fly ensuring that the presented dose distribution is deliverable. Modified spot weights are returned to the TPS using scripting. From there the normal pathway to patient treatment can continue. The proposed approach allows the user to produce a desired dose distribution without knowing how to describe it using optimisation objectives, reducing the expertise needed to modify plans. Planning trade-offs are immediately apparent. Additionally, individual fields can be modified. This approach may complement existing automated planning approaches to simplify treatment planning. A demonstration of the software will be presented.

## P120 -

### Do proton therapy centers consider variable RBE in clinical practice?

#### Armin Lühr^1,2^, Lena Heuchel^1^, Christian Hahn^1,2,3^, Manjit Dosanjh^4^, Jörg Pawelke^2,5^, Brita Singers Sørensen^6^

##### ^1^TU Dortmund University, Faculty of Physics, Dortmund, Germany ^2^OncoRay – National Center for Radiation Research in Oncology, Faculty of Medicine and University Hospital Carl Gustav Carus- Technische Universität Dresden- Helmholtz-Zentrum Dresden-Rossendorf, Dresden, Germany ^3^Technische Universität Dresden, Department of Radiotherapy and Radiation Oncology- Faculty of Medicine and University Hospital Carl Gustav Carus, Dresden, Germany ^4^CERN, o, Geneva, Switzerland ^5^Helmholtz-Zentrum Dresden-Rossendorf, Institute of Radiooncology – OncoRay, Dresden, Germany ^6^Aarhus University Hospital, Department of Experimental Clinical Oncology and Danish Center for Particle Therapy- DCPT, Aarhus, Denmark

Recent clinical reports indicate the variability of proton relative biological effectiveness (RBE) as a function of, e.g., linear energy transfer (LET). This work analyses how proton therapy (PT) centers consider RBE in current clinical practice. In 2020, 25 European PT centers treating patients were asked to respond to an online questionnaire. It consisted of 38 questions and addressed six topics impacting clinical RBE: treatment field arrangement, robust optimization, variability of RBE in treatment planning, estimation of LET and RBE for patient treatment, RBE consideration for patient follow-up and future improvements. Twenty-four centers (96%) responded, showing the high level of interest in the topic. All centers prescribed a constant RBE of 1.1 (Fig. 1a). However, all but one center applied various measures to counteract a potentially variable RBE (Fig. 1b). Nearly all centers (21/22, 95%) considered RBE variability (primarily) in organs at risk (OAR) (Fig. 2a). Most commonly, treatment field arrangements were selected that mitigate an increased risk of toxicity due to potentially elevated RBE (Fig. 2b). Most centers (17/23, 74%) performed patient-specific calculations of, e.g., LET or variable RBE either to study possible clinical RBE effects or inform treatment planning. Going forward, centers are demanding more clinical evidence on RBE variability and standardized LET and RBE calculations available in commercial treatment planning systems. European PT centers are pursuing a two-pronged strategy: constant RBE prescription of 1.1 while actively accounting for RBE variability in OARs to reduce toxicity. They strongly urge clinical trials and more research to resolve current RBE discrepancies.

## P121 -

### A dosimetric comparison of a single isocentre PBS proton therapy technique and tomotherapy for bilateral breast cancer with nodes

#### Zubin Master^1^, Faye Lim^1^, Grace Kusumawidjaja^1^, Sung Yong Park^1^

##### ^1^National Cancer Centre Singapore, Department of Radiation Oncology, Singapore, Singapore

**Purpose:** To evaluate and compare a single isocentre PBS proton therapy technique with TomoTherapy for the treatment of bilateral breast cancer with nodes.

**Background:** Synchronous bilateral breast cancer with nodal involvement is a challenging site to treat with conventional radiotherapy. Advancements in proton therapy in PBS and multifield optimization (MFO) have created an opportunity to treat these complex volumes with comparable coverage, conformity, superior OAR sparing and less integral dose.

**Methods:** Five patients previously treated for bilateral breast cancer on TomoTherapy, were selected for this planning study. Treatment volume for all patients included both breasts and nodes on at least one side. These patients were re-planned for Proton Therapy, using PBS and robust MFO optimization. A single isocentre was used, with the isocentre placed along the midline and posterior to the treatment volume, to maximize coverage. Three beams with range shifters were used with angles of approximately 310, 0 and 50°. The combined CTV of the breast/chest wall and the nodal volumes was robustly optimized using MFO. The proton plans were compared to the TomoTherapy plans for target coverage and OAR doses, namely to the heart, lungs and cord. The plans were tested for robustness and integral dose was also calculated to quantify the dose to unclassified normal tissue.

**Results:** The proton therapy plans easily achieve comparable robust coverage for the target volume, and equivalent or better OAR sparing than the TomoTherapy plans. Moreover, the dose to the unspecified tissues (lateral chest-wall on both sides) is also drastically reduced.

## P122 -

### Time Optimized Intensity Modulated Proton Therapy using knowledge of beam delivery dynamics

#### A. Meijers^1^, R. Hytönen^2^, I. Huth^1^, J. Timmer^1^, T. Koponen^2^, C. Smith^1^, A. Knopf^3^

##### ^1^Varian Medical Systems, Varian Proton Solutions, Palo Alto, USA ^2^Varian Medical Systems, Radiation Oncology Solutions, Palo Alto, USA ^3^University Hospital of Cologne, Department of Internal Medicine- Center for Integrated Oncology Cologne, Cologne, Germany

**Introduction:** To gain greater control over proton treatment delivery times, we propose to interface the room-specific parametrized beam delivery dynamics algorithm with the treatment planning optimizer. This allows to optimize spot patterns, resulting in treatment plans that are more efficient in terms of delivery time, enabling Time Optimized Intensity Modulated Proton Therapy (TO-IMPT).

**Materials and Methods:** A standalone version of a ProBeam (Varian, USA) proton delivery system (PDS) scanning conversion algorithm was employed to calculate PDS settings required for the delivery of a given spot pattern in the Eclipse (Varian, USA) treatment planning system (TPS). A cost function was introduced, which considers spot monitor units (MU), irradiation time and cyclotron beam currents to calculate a cost per spot. Spots that contribute little dose, but are delivery time costly were revised and MUs were redistributed. To illustrate its delivery time controlling power, a hypo-fractionated liver cancer and lung cancer cases were optimized using the TO-IMPT for 3-field plans.

**Results:** Figure 1 shows the reduction in treatment delivery time and the target dose homogeneity as a function of percental spot number reduction. Using TO-IMPT, the delivery time for the exemplary cases could be reduced by up to 63% or 38% respectively, depending on the chosen dosimetric trade-off.

**Conclusions:** As a proof of concept, we demonstrated that more delivery time efficient IMPT plans can be created by making the optimizer aware of the beam delivery dynamics. TO-IMPT allows for a controlled trade-off between delivery speed and dosimetric characteristics based on patient-specific needs.

## P123 -

### Investigating water radiolysis during proton mini and micro beam irradiation with TOPAS-nBio

#### Celine Karle^1^, Miguel Molina Hernández^1^ Lucas Noberto Burigo, Riccardo Dal Bello, Joao Seco

##### ^1^DKFZ Heidelberg, e041, Heidelberg, Germany

The main focus of this project is to assess the chemical products of water radiolysis during proton mini and micro beams irradiation with TOPAS-nbio. Mini and micro beams are characterized by the alternation of high dose peaks and low dose valleys. The peak to valley distance commonly used is either millimeter or micrometer for mini or micro beam respectively. Molecular candidates, such as H_2_O_2_, are currently being investigated in order to potentially explain the mechanisms of this radiation therapy form. TOPAS-nbio is an extension of the TOPAS software to adjust it to radiobiological applications. Therefore, the MC simulation can be used to model the temporal production, interaction and diffusion of different radiolysis products. Firstly, a single proton beam, which irradiates a water phantom, is modeled. The produced chemical species during the pre-chemical and chemical state up to 1 μs are scored. The temporal change of the distribution of the molecules H_2_O_2_and HO_2_ of special interest since they reach a steady state after 1 μs, which could potentially explain the efficiency of micro and mini-beam radiation therapy. The steady-state conclusion has previously been drawn by the TRAX-CHEM simulation [Dal Bello, R., et al. (2020). Proposal of a Chemical Mechanism for Mini-Beam and Micro-Beam Efficacy, https://doi.org/10.3389/fphy.2020.564836]. To verify this finding, the temporal evolution of the relative abundance from the mentioned molecules and the H_2_O_2_ distribution with increasing distance from the beam axis is investigated with the TOPAS-nbio software and compared to the TRAX-CHEM results.

## P125 -

### Approach for potentially mitigating phantosmia in cranial irradiation with single proton energy layer shooting through the olfactory structure

#### Brian Neal^1^, Mingcheng Gao^2^, Terri Bevolo^2^, Dennis Mah^1^, William Hartsell^2^, Jonathan Yang^3^, Brian Chon^1^

##### ^1^New Jersey ProCure, Proton Therapy Center, Somerset, USA ^2^Northwestern University, Northwestern Medicine Proton Center, Warrenville, USA ^3^Memorial Sloan Kettering Cancer Center, Radiation Oncology, New York, USA

**Rationale**: Patients undergoing radiotherapy to the brain may experience an unpleasant phantom smell or nausea. In proton beam delivery, each layer giving significant dose to the olfactory structure has been shown to trigger the sensation. The sensation may be strong enough to cause involuntary motion, creating uncertainty in subsequent layer delivery. In extreme cases, it may require stopping treatment.

**Methods**: We developed a new strategy to minimize treatment disruption, by preventing spots from being placed near the olfactory structure. A single high energy layer is then added covering only the olfactory structure. This lets the optimizer create a homogenous dose in the entire cranial target, with most of the dose to the olfactory structure coming from a single energy layer. The default beam energy delivery order in our system is from highest energy to lowest energy. However, this can be manipulated to deliver the highest energy layer last. Thus, the phantosmia/nausea will be experienced after the majority of dose has been delivered to the rest of the cranial target. The patient can be taken off the table immediately after the sensation with no disruption to the treatment.

**Results**: Plans have been generated using posterior-anterior fields for application in craniospinal irradiations with whole brain radiation. Dose to the forehead slightly increases, which is clinically acceptable. We expect to report on preliminary clinical results on patients treated with this approach.

**Conclusions**: A novel planning approach has been developed to potentially mitigate nausea by using a single proton energy layer to treat the olfactory structure.

## P126 -

### Experimental verification of dose calculation algorithm for BNCT by a combination of Monte Carlo and superposition methods

#### M. Nojiri^1^, T. Takata^2^, H. Tanaka^2^, Y. Sakurai^2^

##### ^1^Kyoto University, Department of Nuclear Engineering- Graduate School of Engineering, Kumatori-cho- Sennan-gun- Osaka, Japan ^2^Institute for Integrated Radiation and Nuclear Science- Kyoto University, Particle Radiation Oncology Research Center, Kumatori-cho- Sennan-gun- Osaka, Japan

Clinical treatment using accelerator-based Boron neutron capture therapy (BNCT) was started. Accordingly, efficiency in the treatment planning becomes increasingly important. We have been progressing development of the hybrid algorithm, that is a combination of Monte Carlo (MC) and superposition methods, for the faster dose calculation. In this algorithm, moderation of neutrons was calculated by MC method, and then the thermalization was modeled as a kernel. We experimentally verified the accuracy of this algorithm. Calculation using hybrid algorithm was performed as follows; an isotropic kernel of thermal neutron flux generated by a point source with the energy of 1 eV in water, was prepared. Next, in-phantom flux distribution of neutrons terminating by cut-off energy of 1 eV, was calculated by MC method, and convolution-integrated with the kernel to derive the thermal neutron flux distribution. The thermal neutron flux distribution in a water phantom was experimentally determined by a gold activation method. A set of gold wires was irradiated in the phantom by the epithermal neutron beam at Kyoto University Reactor. Subsequently, the fluxes were determined from the activities of the gold. The calculation result by the hybrid algorithm showed good agreement with the measured result at the depth ≥ 4 cm, while overestimated it in the shallower region. Comparison with the calculation using the full-energy MC method was also made and showed the similar trend. As a result, it was confirmed that the calculation using the hybrid algorithm could partially reproduce the measured values. We will improve the accuracy of this algorithm.

## P127 -

### First dosimetric assessment of proton minibeam arc radiation therapy

#### Ramon Ortiz^1^, Ludovic De Marzi^2^, Yolanda Prezado^1^

##### ^1^Institut Curie, Recherche U1021-UMR3347, Orsay, France ^2^Institut Curie, Centre de Protonthérapie, Orsay, France

Proton Minibeam Radiation Therapy (pMBRT) (Prezado *et al*, 2013) and Arc therapy (Ding *et al*, 2013) are two radiotherapeutic approaches that, individually, have shown an enhancement of the normal tissue sparing potential. In this study, dose distributions resulting from the combination of these two techniques are assessed and compared with a single-field pMBRT treatment plan, which has been proven beneficial in previous in vivo animal studies (Prezado *et al*, 2018). The multi-field pMBRT (i.e., combination of pMBRT and Arc therapy) treatment plan involves 13 equally-spaced fields forming a 120-degree arc around the patient skull (Figure 1), diagnosed with a human glioblastoma at the centre of the brain. The single-field pMBRT consists of one beam in the coronal direction. The collimator utilized in both cases comprises 400 μm width slits separated by 4 mm centre-to-centre distance. The optimisation of the treatment plan was calculated by the ECLIPSE TPS and dose distributions were computed using the TOPAS toolkit. The combination of pMBRT and Arc therapy leads to a 30-90% reduction of peaks and valley doses to healthy tissues compared to the single field pMBRT approach. Furthermore, the addition of treatment fields does not modify the peak-to-valley dose ratios nor create hot spots in normal tissues. Therefore, the combination of both techniques does not reduce the dosimetric benefits of pMBRT but increases its potential of tissue sparing by reducing the dose delivered to non-tumour volumes.

## P128 -

### Comparisons of an improved pencil beam algorithm vs Monte Carlo dose calculation in inhomogeneous tissue.

#### Jeevan Pandey^1^, Jamil Lambert^1^, Ignacio Di Biase^1^, John Pettingell^1^

##### ^1^Rutherford Cancer Centre, Rutherford Cancer Centre, Newcastle, United Kingdom

The purpose of this study was to evaluate the accuracy of dose calculation in inhomogeneous regions generated by Pinnacle using a pencil beam algorithm compared with RayStation using both Monte Carlo (MC) and Pencil Beam algorithms. Fifteen patients with oropharyngeal carcinoma were selected for this case study. Plans were generated in Pinnacle using a Pencil Beam algorithm and compared to the Monte Carlo and pencil beam dose calculation algorithms from RayStation. Evaluation parameters such as target volume coverage, OARs sparing particularly in lung sparing and spinal cord sparing are analysed. PTV coverage shows that the improved PB algorithm (Pinnacle) overestimates the dose distribution compared to the MC algorithm (RayStation). There was not much difference on OARs sparing between the algorithms. In proton beam therapy, Monte Carlo algorithm is deemed as a gold standard algorithm to accurately calculated the dose distribution in inhomogeneous region (lung, oesophagus). Our results show that there are also differences in the dose calculation of different pencil beam algorithms in inhomogeneous regions which can have an effect on target coverage.

## P129 -

### Accounting for beam modulation due to microscopic lung heterogeneities in dose calculation and optimisation for particle therapy

#### Athena Paz^1^, Kilian-Simon Baumann^2,3^, Ulrich Weber^1^, Matthias Witt^3^, Klemens Zink^2,3^, Marco Durante^1,4^, Christian Graeff^1^

##### ^1^GSI Helmholtzzentrum für Schwerionenforschung GmbH, Biophysics, Darmstadt, Germany ^2^University of Applied Sciences, Institute of Medical Physics and Radiation Protection, Giessen, Germany ^3^Marburg Ion-Beam Therapy Center, Marburg Ion-Beam Therapy Center, Marburg, Germany ^4^Technical University Darmstadt, Technical University Darmstadt, Darmstadt, Germany

**Purpose:** Bragg peak degradation arising from density heterogeneities in the beam path leads to underdosage of the tumour volume and overdosage of healthy tissues distal to the target. Therefore, we adapted GSI's TRiP98 treatment planning system to compensate for the beam-modulating effect of heterogeneous lung tissue in physical and biological inverse treatment planning.

**Methods:** The implementation employs an analytical model that relates the severity of degradation to the established ‘modulation power' parameter and the total water-equivalent thickness of lung parenchyma traversed by the beam. The beam-modulating effect was reproduced through convolution of the reference Bragg curve with the model-derived Gaussian kernel. For biological doses, the degradation was determined by modulating dose-averaged *α*, *β* and LET distributions of the unperturbed beam. Carbon SOBP measurements behind lung substitute material were performed to validate the code.

**Results:** The passage through a 20-cm Gammex LN300 led to the decrease in target coverage and broadening of the SOBP distal fall-off. However, dose coverage is regained as a result of the optimisation (Figure 1). The patient case (Figure 2) revealed a 3.3% decrease in D95_PTV_ from degradation, which was reduced to a 0.2% difference after optimisation. Additionally, widening of the RBE distribution beyond the target distal edge is observed. This implies a degradation enhancement in the biological dose, which could be detrimental to healthy tissues behind the target.

**Conclusion:** With the capability to simulate and compensate lung dose perturbations, larger patient studies will follow to judge clinical effects also in IMPT, where margins might prove insufficient.

## P130 -

### Quantitative assessment and practical considerations of skin dose for head and neck cancer proton therapy

#### Minglei Kang^1^, Shouyi Wei^1^, Haibo Lin^1^, Arpit M Chhabra^1^, J Isabelle Choi^1^, Shaakir Hasan^1^, Charles B Simone II^1^, Madhur Garg^2^, Brian Yeh^3^, Christopher Barker^4^, Rafi Kabarriti^2^, Sonam Sharma^3^, Richard Bakst^3^, Nancy Lee^4^, Robert Press^1^

##### ^1^New York Proton Center, Radiation Oncology, New York, USA ^2^Montefiore Medical Center, Radiation Oncology, New York, USA ^3^The Mount Sinai Hospital, Radiation Oncology, New York, USA ^4^Memorial Sloan Kettering Cancer Center, Radiation Oncology, New York, USA

**Purpose**: Proton therapy has been shown to significantly reduce OARs doses for head and neck(HN), but its effect on skin is not well defined. This study aims to quantitively assess skin dose and develop clinical strategies to minimize skin reactions in HN intensity-modulated proton therapy(IMPT).

**Method/materials**: Ten consecutive HN cancer patients with low-risk node-negative necks previously treated using IMPT to 56Gy/30 fractions were re-planned using Rapid-Arc to evaluate skin dose difference. A skin structure(real_skin) was created using a 3mm margin from patient's surface and a 5mm expansion structure from skin surface into air was created(skin_air) to assess proximal dose spillage that could eventually contribute dose to skin due to the range and dose uncertainties from anatomy/setup changes(Fig1). A layer-removing method was applied as a mitigation technique to quantify differences in skin dose and target coverage.

**Results**: The real_skin D_0.03cc_ and D_5cc_ were 11%(6.2Gy) and 8%(4.3Gy) higher for IMPT than Rapid-Arc, respectively. The skin_air D_0.03cc_ and D_5cc_ were 13%(7.1Gy) and 13%(7.3Gy) higher for IMPT than Rapid-Arc, respectively. Removal of the first energy-layer reduced the D_0.03cc_ and D_5cc_ real_skin dose by 1%(0.6Gy) and 1.8%(1Gy) without any CTV coverage loss. Removing the first two layers significantly reduced skin_air D_0.03cc_ and D_5cc_ 4.7%(2.7Gy) and 5.2%(2.9Gy) while reducing CTV D95% by 3%(Fig2).

**Conclusion**: This study quantifies differences in skin dose between IMPT and Rapid-Arc therapy. Use of an adaptive technique to remove lower-layer proton spots reduces skin dose with minimal compromise in target coverage. Thoughtful consideration of planning strategies can mitigate IMPT skin dose while maintaining quality treatment.

## P131 -

### Effects of spot positions on the dose and dose rate of uniform FLASH transmission field

#### Michiko Rossi^1^, Ropo Matti^1^, Pierre Lansonneur^1^, Viljo Petäjä^1^

##### ^1^Varian Medical Systems, Proton Department, Helsinki, Finland

Preclinical studies have shown that radiation therapy delivered at ultra-high dose rates reduces normal tissue toxicity while preserving tumor control. Recently a method for calculating the dose rate delivered using pencil beam scanning has been proposed [1]. Using this method, we calculate and compare the dose and dose rate on a volume for a uniform field and examine the effects of varying spot distances, spot grid structures and scan patterns. A dose rate calculation algorithm using the proposed method in [1] is developed. The algorithm evaluates the dose distribution of each pencil beam using a dose calculation algorithm, and combines it with the individual spot timings which are calculated by modelling the scanning dynamics of the ProBeam treatment delivery system. This scanning model in the algorithm provides consistent timing information with the one reported by the ProBeam machine. For a uniform transmission field with fixed dose per volume, the spot positions and the order for which the spots in the field are delivered affect the dose and the dose rate. The study provides insight into if and how the dose rate can be optimized for FLASH transmission fields using the proposed dose rate definition.

## P132 -

### Instant coverage estimation in CIRT for lung cancer with tumor matching positioning

#### Makoto Sakai^1^, Yoshiki Kubota^1^, Nobuteru Kubo^1^, Daisuke Irie^1^, Mutsumi Tashiro^1^, Tatsuya Ohno^1^

##### ^1^Gunma University, Heavy Ion Medcial Center, Maebashi, Japan

**Background and Purpose:** Patient positioning by tumor matching is effective for carbon-ion radiotherapy (CIRT) in the case of lung cancer. However, the coverage, which is determined in the case of few patients, is unacceptably low. Thus, we propose a simple calculation algorithm to estimate the coverage at the time of irradiation, using only planning computed tomography (CT).

**Material and Methods:** The CT images acquired before treatment initiation for confirmation (Conf-CT) are obtained under the same conditions as that of the planning CT. The interfractional variation of the water-equivalent path length (WEL) of the chest wall is identified. The coverages are estimated using the proposed algorithm and compared with those recalculated by the treatment planning system with the Conf-CT. Using the proposed algorithm, we estimated the coverage with various tumor displacements in patients. Furthermore, the estimated coverages were plotted as two-dimensional maps.

**Results and discussion:** Although the interfractional-variation value depends on the chest region, the ratio was found to be consistent. The average (±standard deviation) of the interfractional-variation ratio was 5.1(±2.9)% and 4.7(±2.4)% for the horizontal and vertical beams, respectively. There were six unacceptable cases (coverage<95%), which were successfully detected using the proposed algorithm, based on the average value of the interfractional-variation ratio. The predictive coverage map (Figure) using the proposed algorithm indicated that the areas of acceptable coverage are spread along the rib bone. The algorithm is considerably simple and estimates the advisability for irradiation with TM on the clinical site if these maps are prepared a priori.

## P133 -

### Experimental verification of stopping power ratios in organic samples by dual energy CT for clinical application

#### Makoto Sakama ^1^

##### ^1^National Institute for Quantum and Radiological Science and Technology QST, QST Hospital, Chiba, Japan

With the aim of improving therapeutic effect and efficiency, multi-ion therapy combining various particle species, expansion of treatments for non-cancer minute diseases, and research on hypo-fractionated irradiations are being conducted, and more complex and accurate treatments are under development in National Institute of Radiological Sciences, QST. In order to perform such highly sophisticated treatments, it is essential to improve the accuracy of dose calculation and range estimation. In addition, the current conversion method of single energy CT (SECT) cannot deal with variations in the stopping power ratios within and between patients. In this study we explores the simple and robust conversion methods of dual energy CT (DECT) so that clinical application of DECT can proceed smoothly from the conversion method of SECT. Applying the stoichiometric calibration method currently used for treatments to DECT with the simple calibration phantoms [1], the stopping power ratios of several fluid organic samples obtained by DECT were verified by comparing with the results from range measurements, and the uncertainty and robustness were evaluated. Considering the current SECT imaging conditions, we examined the efficient operation method of DECT in treatment planning and report the results of estimation of the stopping power ratios under the planned operational condition using DECT.

## P134 -

### Comparative study of IMPT planning and delivery for lung SABR using two different PBS systems

#### Z. Su^1^, W. Hsi^1^, E. Brooks^1^

##### ^1^University of Florida Proton Therapy Institute, Department of Radiation Oncology, Jacksonville, USA

**Introduction**: Stereotactic Ablative Body Radiotherapy (SABR) is an effective treatment for early-stage lung cancer. This study investigates the clinical spot sizes, plan dosimetric quality, and delivery time needed for practical and effective treatment employing two different proton therapy delivery systems (Proteous-plus, P+ and Proteus-one, P1).

**Materials and Method**: A total of 9 lung patients with 10 tumors were selected for this study. Seven targets were prescribed and planned with 60Gy in 10 fractions; three targets with 48Gy in 4 fractions in Raystation 8A. To generate large spot size, P1 with 4cm range-shifter placed at 45cm away from isocenter. P+ plans used 7cm range-shifter placed 4cm away from patients; Target coverage was optimized and normalized to prescription dose covers 100-percent of GTV and 90-percent dose covers PTV. Dose to the OARs were compared using QUANTEC metrics. Each plan was split into three subsets to perform volumetric dose-repainting to reduce interplay effect. All plans delivery time were obtained and compare for efficiency.

**Results**: The in-air beam spots at isocenter were comparable between the two configurations (Table 1). All PTVs were covered by 90-percent prescription dose. OAR doses were comparable between P1 and P+ plans and within the QUANTEC limits (Table 2). The delivery time of P1 plans were significantly shorter than those of P+ system (Table 3).

**Conclusion**: Both systems can be used for lung SABR IMPT treatment with similar spot size and dosimetric quality. However, the significant shorter beam-on time of P1 system may lead to less patient movements during treatment, thus, less dose uncertainties.

## P135 -

### Robust evaluation and comparison of photon and proton plans for a craniopharyngioma case: Towards equitable photon-proton plan comparison In Australia.

#### Jonathan Sykes^1^, Adam Yeo^2^, Scott Penfold^3^, Matthew Jennings^3^, Hanh Vu^1^, Verity Ahern^1^

##### ^1^Sydney West Cancer Network, Radiation Oncology, Sydney, Australia ^2^Peter Maccallum Cancer Centre, Radiation Oncology, Melbourne, Australia ^3^Royal Adelaide Hospital, Radiation Oncology, Adelaide, Australia

To develop Australian expertise in proton therapy planning before the Australian Bragg Centre for Proton Therapy and Research opens, three virtual workshops were convened in 2020 with four hospitals and representatives of the Radiation Oncology, Medical Physics and Radiation Therapy professional bodies: RANZCR, ACPSEM and ASMIRT. We report the analysis of robust evaluation and comparison of photon and proton plans submitted to the second workshop. The CT scan and associated structures, Clinical Target Volume (CTV) and 13 organs at risk (OAR) for a paediatric craniophayngioma case were distributed and centres asked to provide their best photon and proton plan according to a set of planning objectives. For each plan, dose was calculated for uncertainty scenarios simulating 5mm setup and 3% range uncertainty. With these scenarios, voxel-wise, minimum (VoxMin), maximum (VoxMax) and mean (VoxMean) were calculated. Robust evaluation showed all plans (three centres) had small but acceptable loss of CTV coverage as shown by the VoxMin Dose Volume Histograms (Figure 1). One proton plan exhibited both greater loss in coverage (VoxMin) and increased hot-spots (VoxMax). OAR dose was typically less for proton plans but there was considerable variability in both proton and photon plans. One centre achieved similar OAR sparing with a photon plan compared to the typical proton plan. Nearly all plans were robust to set-up and range uncertainty allowing equitable comparison of OAR dose. There was significant variation in OAR sparing between centres indicating the need for a national consensus to achieve optimum plans, whether by protons or photons.

## P136 -

### Dosimetric impact of using different spot placement techniques in proton pencil beam therapy

#### Mahboob Ur Rehman^1^, Kevin Erhart^2^, Twyla Willoughby^3^, Sanford Meeks^3^, Patrick Kelly^4^, Omar Zeidan^3^

##### ^1^University of Central Florida, Physics, Orlando, USA ^2^.decimal LLC- Sanford- Florida, .decimal, Sanford, USA ^3^Orlando Health Cancer Institute, Medical Physics, Orlando, USA ^4^Orlando Health Cancer Institute, Oncology, Orlando, USA

Intensity modulated proton therapy (IMPT) has shown improvement in treatment plan quality as compared to conventional proton and photon-based radiotherapy techniques. It has been demonstrated that IMPT dosimetry can be further improved by implementation of advanced spot placement techniques. This work presents a plan quality comparison for five spot placement techniques. These include two grid-based (rectilinear/hexagonal) and three boundary contoured (concentric-contours, hybrid and optimized) techniques. Treatment plans were created for two different target volumes, (one cone-shaped and one spherical). An optimal set of planning parameters was defined for all treatment plans and the impact of spot placement techniques on the plan quality was studied in terms of lateral and distal dose falloff, normal tissue sparing, conformity and homogeneity of dose distributions, and total number of spots. For grid-based spot placement techniques, dose conformity is dependent on the target cross sectional shape, which changes for each proton energy. This variable conformity problem is shown to be mitigated by using boundary contoured techniques. However, in the case of concentric-contours, the conformity is improved but at the cost of decreased homogeneity. Hybrid and optimized spot placement techniques show more uniform dose distributions while maintaining the improved dose conformity. The optimized spot placement technique is shown to provide robust treatment plans with improved target coverage, homogeneity of dose, and minimal spots count. These results highlight that plan quality may be improved for many patients, without the need for expensive delivery equipment updates, simply by providing additional spot placement techniques in commercial treatment planning software.

## P137 -

### Evaluation of distal edge planning guidelines in proton PBS treatment planning of brain tumours

#### Anne Vestergaard^1^, Jesper Kallehauge^1^, Peter Lægdsmand^1^, Klaus Seiersen^1^, Bob Smulders^1,2^, Petra Witt Nyström^1,3^, Yasmin Lassen-Ramshad^1^, Morten Høyer^1^, Ole Nørrevang^1^, Stine Korreman^1^

##### ^1^Aarhus University Hospital, Danish Centre for Particle Therapy, Aarhus N, Denmark ^2^University of Copenhagen, Department of Oncology- Rigshospitalet, Chopenhagen, Denmark ^3^Skandionkliniken, Skandionkliniken, Uppsala, Sweden

**Background and purpose**: Proton treatment planning guidelines for brain tumours often include instructions to avoid beam angles placing the distal edge in the brainstem. The aim of this study was to quantify the cost in mean dose to the normal brain (Brain-CTV) and to evaluate the potential increase in variable RBE to the brainstem if the distal edge planning guideline is omitted.

**Methods and Materials**: Eighteen brain tumour patients treated according to in-house treatment planning guidelines were included. All patients received more than 52.5 GyRBE to the brainstem. The local guidelines imply that maximum one out of minimum three field can have the distal edge in the brainstem. The patients were re-planned (Replan) without the distal edge rule to improve other OAR doses, such as mean dose to Brain-CTV. Monte Carlo simulations (TOPAS v3.5) were used to calculate dose and dose averaged LET for both plans. Variable RBE doses were calculated using the McNamara model, assuming α/β=2 for normal tissue (RBE_var_). Mean dose to Brain-CTV and the brainstem V54GyRBE_var_ were compared between the two plans using Wilcoxon singed-rank test.

**Results**: The mean dose to Brain-CTV was reduced by a mean of 10%[-20;26] (p=0.001). V54GyRBE_var_ increased by a mean of 1 cc [-0.3;4.9] (p=0.001).

**Conclusion**: Allowing more beams having distal edge in the brainstem significantly reduced the mean dose to Brain-CTV, while significantly increasing the biological dose to the brainstem. Until advanced optimisation methods are clinically available simple planning guidelines, like avoiding the distal edge in more than one of minimum three fields, are warranted.

## P138 -

### Development of a new optimization algorithm for Arc Proton Therapy treatment planning

#### S. Wuyckens^1^, G. Janssens^2^, E. Sterpin^1,3^, J. Lee^1^, K. Souris^1^

##### ^1^UCLouvain, Molecular Imaging- Radiotherapy and Oncology MIRO, Brussels, Belgium ^2^Ion Beam Applications SA, Research department, Louvain-La-Neuve, Belgium ^3^KULeuven, Department of Oncology, Leuven, Belgium

**Introduction and purpose**: Arc Proton Therapy (ArcPT) is an emerging technique that has the possibility of improving both the outcome and the efficiency of the treatment. This modality constrains the number of energy layers as it primarily determines treatment delivery time. Therefore, the challenge lies in selecting as few energy layers as possible for each beam direction. In this study, we designed a beamlet-based algorithm for the optimization of ArcPT that distributes a number of energy layers similar to conventional therapy over more gantry angles.

**Material and methods**: The ArcPT optimization problem is formulated with an objective function defined conventionally as the quantitative evaluation criterion for the delivered dose distribution combined with new objectives respecting constraints on the number of energy layers used over the arc.The proposed method was tested on a lung tumor case and evaluated according to these criteria: objective function value, layer sparsity and DVH metrics.

**Results and discussion**: An ArcPT plan with only one energy layer per control point was successfully obtained with good dosimetry results (D95=96.5% prescription) as shown in fig. 1a and 1b.

**Conclusion**: The algorithm proposed for ArcPT treatment plan optimization has successfully solved the energy layer selection problem by generating acceptable plans with good dosimetry results. The definition of our objective function will be completed with a sequencing energy term that favors small consecutive energy decrements to further reduce irradiation time.

## P139 -

### Proton GRID and Lattice therapy using pencil beam scanning with breath-hold motion management.

#### M. Zhu^1^, P. Patel^1^, J. Bradley^1^, M. McDonald^1^, H. Chen^2^, J. Snider^3^, W. Regine^4^, K. Langen^1^

##### ^1^Emory University School of Medicine, Radiation Oncology, Atlanta, USA ^2^Emory Healthcare, Radiation Oncology, Atlanta, USA ^3^University of Alabama at Birmingham School of Medicine, Radiation Oncology, Birmingham, USA ^4^University of Maryland School of Medicine, Radiation Oncology, Baltimore, USA

**Purpose**: To characterize the dosimetric parameters and demonstrate the feasibility of proton GRID and Lattice therapy using a PBS system with breath-hold motion management technique.

**Methods**: CT images obtained on a patient with hepatocellular carcinoma, scanned with breath-hold technique were used. An in-house script generated evenly spaced cylinders and spheres within the GTV. The diameter of cylinders and spheres was 3mm; the center-to-center distance between the cylinders and spheres was 2.23cm. The cylinders and the sphere lattices were positioned initially along the 325^0^ gantry angle beam's eye view (BEV). One beam at 325^0^ was used to create a GRID plan and a Lattice plan; another Lattice plan was generated with one gantry 345^0^ and couch 60^0^ beam, avoiding the overlap of the spheres in the BEV. All plans were optimized to achieve uniform dose of 15 Gy to the cylinders or spheres while minimizing dose everywhere else.

**Results**: Figure 1(a-c) shows the dose distribution for the 3 plans. Dose profiles perpendicular to the CAX of the GRID plan, figure 1d, shows the peak dose of 15 Gy and peak-to-valley ratio of 6.8, 6.8, and 2.7 for the 3 plans. Along the CAX (figure 1e), GRID plan had uniform dose in the cylinders, peak-to-valley ratio was 1.3-1.6 and 3.7 for the two Lattice plans. All plans were delivered with a clinical proton system, the beam-on times were 32s, 32s, and 40s, respectively.

**Conclusion**: Delivering PBS proton GRID and Lattice treatment is feasible on a clinical proton system within one breath-hold.

## P140 -

### 4D robust optimization in small spot intensity-modulated proton therapy (IMPT) for distal esophageal carcinoma

#### H. Feng^1^, J. Shan^1^, J. Ashman^1^, W. Rule^1^, R. Bhangoo^1^, N. Yu^1^, J. Chiang^1^, M. Fatyga^1^, W. Wong^1^, S. Schild^1^, T. Sio^1^, W. Liu^1^

##### ^1^Mayo Clinic Arizona, Department of Radiation Oncology, Phoenix, USA

**Introduction:** In this study, we compared the dosimetric performances of small-spot 3D and 4D robustly optimized intensity-modulated proton (IMPT) plans in the presence of uncertainties and interplay effect simultaneously for distal esophageal carcinoma.

**Methods:** Thirteen patients were selected and re-planned with small-spot (∼2-6mm) 3D and 4D robust optimization in IMPT respectively. The internal clinical target volumes (CTV_high_, CTV_low_) were used in 3D robust optimization. Different CTVs (CTV_high4d_, CTV_low4d_) were generated by subtracting an inner margin of the motion amplitudes in 3 cardinal directions from the internal CTVs and used in 4D robust optimization. All patients were prescribed with the same doses to CTVs. Dose-volume-histogram (DVH) indices were calculated to evaluate plan quality. Comprehensive evaluations that consisted of 300 perturbed scenarios (10 different motion patterns to consider irregular motion and 30 different uncertainties scenarios combined, both were randomly sampled from the corresponding Gaussian distributions) were done to quantify robustness to uncertainties and interplay effect simultaneously. Wilcoxon signed-rank test was used for statistical analysis

**Results:** Compared to 3D robustly optimized plans, 4D robustly optimized plans had statistically improved target coverage and better sparing of lungs and heart (Fig. 1). 4D robustly optimized plans had better heart protection when uncertainties and interplay effect were considered (Fig. 2).

**Conclusions:** Even with small spots in IMPT, 4D robust optimization outperformed 3D robust optimization in terms of normal tissues protection and robustness to uncertainties and interplay effect. Our findings support the use of 4D robust optimization to treat distal esophageal carcinoma with small spots in IMPT.

## P141 -

### Identification of motion amplitudes in need for motion mitigation for synchrotron-based carbon ion therapy

#### Franciska Lebbink^1,2^, Markus Stock^1^, Dietmar Georg^2^, Barbara Knäusl^1,2^

##### ^1^MedAustron Ion Therapy Centre, Medical Physics, Wiener Neustadt, Austria ^2^Medical University of Vienna, Department of Radiation Oncology, Vienna, Austria

To irradiate tumors prone to interplay effects due to breathing at a synchrotron-based ion-beam facility, motion amplitudes that require motion mitigation need to be identified. Pencil beam scanned carbon beams and the ARDOS breathing phantom [1] were employed for investigating 8 motion scenarios, namely a tumor motion of 0.6, 1, 1.5 and 2cm alone and in combination with rib movement of 3mm and lung expansion of 2mm. A treatment plan to deliver a biologically-weighted dose of 5.0Gy was created on the static CT using RayStation7.99 (RaySearch) and delivered to the static and moving phantom 3 times for each scenario. The movement was synchronized with the beam and the dose was measured using PinPoint (PP) ionization chambers (TM30013, PTW) inside (PP1-2, 5), in the penumbra (PP3) and outside the tumor (PP4) [2]. For the center of the tumor (PP2) the dose variation was within 3% for all motion scenarios, only for 2cm tumor movement in combination with rib and lung it increased up to 7% (Figure 1). PP5 exceeded the 10% deviation with the 2cm motion scenarios. Dose deviations in the penumbra (PP3) increased from 6 to 29% for increasing amplitude. PP4 in distal target region received in a static scenario less than 0.1Gy but increased up to 0.3Gy with motion. The presence of rib and lung motion did not automatically deteriorate the results, which depended strongly on the chamber location. Although the measured high dose areas remained mostly unaffected, OARs adjacent to the tumor require motion mitigation for carbon ions.

## P142 -

### A computer simulation approach of the interplay effect of intrafraction organ motion in line scanning proton beam delivery.

#### Pei-Yi Lee^1^, Bing-Shen Huang^1^, Chung-Chi Lee^2^, I-Chun Cho^1^, Pei-Jiuan Juang^1^, Pei-Ju Chao^1^, Shen-Hao Li^2^, Chen-Shou Chui^1^

##### ^1^Kaohsiung Chang Gung Memorial Hospital, Proton and Radiation Therapy Center, Kaohsiung, Taiwan ^2^Linkou Chang Gung Memorial Hospital, Proton and Radiation Therapy Center, Taoyuan, Taiwan

**Purpose**: To investigate the interplay effect of intrafraction organ motion in line scanning proton beam delivery.

**Methods**: A simplified water phantom with a static spherical target was simulated in this study. A pencil beam line scanning plan was generated by using the RayStation 8B to deliver 72.6 Gy/22 fractions over the static planning target volume (PTV). The plan included energy layers, scanning patterns, and MU settings. For implementation on numerical computation, the line scanning pattern was discretized at 100 μsec time intervals. At each time instant, the pencil beam energy, position, and MU were determined from the plan and applied to a water phantom with a moving target for calculating the dose. Interplay effect was simulated with the target undergoing a sinusoidal motion with an amplitude of 1 cm and a motion period of 3 seconds. The homogeneity index and Gamma-index were used to evaluate the dose discrepancy between the static and the moving target results.

**Results**: For the static case, doses were uniform in the target. With organ motion, however, doses were distorted. The discrepancy reduced with repainting of 3 times per fraction and 22 fractions.

**Conclusion**: Intrafraction organ motion has a detrimental effect on dose distribution. This interplay effect can be calculated with the plan and the motion pattern. The effect is greatly reduced, however, with repainting and multiple fractions.

## P143 -

### Real-time volumetric imaging based on CT image deformation driven by internal fiducial markers for in-vivo range verification

#### Naoki Miyamoto^1,2^, Risa Hayashi^3^, Kohei Yokokawa^1^, Seishin Takao^1,2^, Koichi Miyazaki^1,2^, Sodai Tanaka^1,2^, Taeko Matsuura^1,2^, Shinichi Shihmizu^2,4^, Kikuo Umegaki^1,2^

##### ^1^Hokkaido University, Faculty of Engineering, Sapporo, Japan ^2^Hokkaido University Hospital, Department of Medical Physics, Sapporo, Japan ^3^Hokkaido University, Graduate School of Engineering, Sapporo, Japan ^4^Hokkaido University, Faculty of Medicine, Sapporo, Japan

**Purpose:** To develop real-time volumetric imaging technique based on CT image deformation driven by displacement of the internal fiducial markers for in-vivo range verification in particle therapy.

**Method:** The deformation vector fields (DVFs) and the displacement of the fiducial markers due to respiration are evaluated for each CT dataset of 4DCT with reference to the exhale phase CT. Then, estimation model to derive the DVF from the marker displacement is optimized for each voxel. During the treatment, the volumetric image can be generated in real-time by applying the DVF estimated from the marker displacement measured by real-time imaging technique to the reference volume. We evaluated the image synthesis accuracy by using clinical 4DCT datasets from six patients including the fiducial markers in lung.

**Results:** The volumetric images generated with the actual marker displacement showed good agreement of tumor location and surrounding organs. Root mean squared error of CT value between actual CT image and the synthetic CT image was less than 100 HU. Computation time to generate the image with volume of 150^3 mm^3 (150 pixels by 150 pixels by 60 slices) was about 70 ms. These results suggested that the water equivalent length (WEL) along beam path evaluated from the synthetic CT images could be applicable for real-time in-vivo range verification.

**Conclusion:** We showed the feasibility of the real-time volumetric imaging technique for in-vivo range verification.

## P144 -

### Proton treatment planning and 4D dose reconstruction evaluation in mediastinal lymphoma patients

#### Pietro Pisciotta^1^, Arturs Meijers^1^, Anne Niezink^1^, Anne Crijns^1^, Syahidah Muntoha^1^, Jekaterina Papina^1^, Visser Visser^1^, Adriaan C. Hengeveld^1^, Johannes A. Langendijk^1^, Gabriel Guterres Marmitt^1^, Stefan Both^1^

##### ^1^University Medical Centre Groningen, University of Groningen, Department of Radiation Oncology, Groningen, Netherlands

**Introduction**: Due to its time structure, the proton pencil beam scanning is more susceptible to intra-fractional motion raising concerns for its clinical deployment despite reducing dose in organs at risk, integral dose and potential neutron contamination in the field. The aim of this study was to investigate target coverage robustness in intensity modulated proton therapy(IMPT) treatments at our institution.

**Materials and methods**: Ten mediastinal lymphoma patients treated with IMPT were evaluated. A 4DCT was acquired for treatment planning and weekly repeated 4DCTs were collected to evaluate possible deviations in target coverage. Layer repainting (5x), large airgaps (∼20cm) and 3D-robust optimisation were utilised to generate clinical plans. Prior to physician approval, plan robustness was evaluated using the vox-wise min/max method for range and setup uncertainties (3%-5/6mm). In addition, treatment plan robustness prior and during the course of treatment was continuously evaluated using an in-house developed fraction-wise reconstruction of 4D-dose distribution and dose accumulation method employing weekly verification 4DCTs and patient specific planning, daily breathing motion patterns and treatment delivery log-files.

**Results**: Average maximal target motion was 12,3 mm (ranging from 6 to 20 mm). No clinically relevant changes in target coverage were found in the fraction-wise reconstructed 4D-dose distributions. The accumulated treatment course doses showed: V95>99.8% and D98>95% for all ten patients. Moreover, D98 remained within 1% from the pre-treatment evaluations.

**Conclusions**: Interplay and motion effects did not result in loss of homogeneity or coverage due to our treatment planning technique. 4D reconstructions enables to do pre-treatment evaluations and are a good predictor for plan robustness.

## P145 -

### Experimental validation of model-based 4D dynamic doses calculated by a commercial treatment planning system in proton pencil beam scanning

#### Y. Tominaga^1,2^, M. Oita^2^, J. Miyata^2,3^, Y. Sakurai^1^, T. Akagi^4^

##### ^1^Medical Co. Hakuhokai Osaka Proton Therapy Clinic, Radiotherapy, Osaka, Japan ^2^Okayama University, Graduate School of Interdisciplinary Science and Engineering in Health Systems, Okayama, Japan ^3^Kurashiki Central Hospital, Radiological Technology, Kurashiki, Japan ^4^Hyogo Ion Beam Medical Support, Support plan division, Tatsuno, Japan

**Purpose:** We implemented a model-based 4D dynamic dose (4DDD) calculation system for pencil beam scanning (PBS) proton therapy under free breathing. This study aims to validate the calculation accuracy of 4DDDs in comparison with measurements.

**Methods:** Three cubic verification plans were created by RayStation 9A in the TomoTherapy cheese phantom that four different material rods were inserted. These plans were created using 10 phase 4D-CT datasets of the phantom on the CIRS Dynamic Platform that was moved with amplitudes of 2.5, 3.75, and 5.0 mm (amp2.5, amp3.75, amp5.0). The 4DDDs were calculated by the in-house TPS scripting that considers the beam-on time, energy switching times, spot delivery times, and breathing periods. Beam measurements were performed with a 2D ionization chamber array in four different starting phases of a 4-second periodic sinusoidal curve. We evaluated the difference between the calculations and measurements with 2D gamma-index analysis.

**Results:** The gamma score of three static plans at 3%/3 mm were more than 98.3%. The mean gamma score of 4DDD plans were 98.2 ± 1.0% for the amp2.5, 98.1 ± 1.5% for the amp3.75, and 97.0 ± 2.2% for the amp5.0, respectively. All measurements resulted in above 92.7%. The dose differences of 2592 points were within ± 5.0% (ranges from -5.8% to 6.4%) except for eight points.

**Conclusion:** Even if the starting phases were changed, our 4DDD calculations less than 5.0 mm amplitudes were good agreements with the measurements. This system enables us to evaluate the pretreatment dose at various respiratory cycles, amplitudes, and starting phases.

## P146 -

### Robust optimized 4D-conformal IMPT in hypo-fractionated carbon ion therapy for complex NSCLC patients

#### Moritz Wolf^1^, Christian Graeff^1^

##### ^1^GSI Helmholtz Centre for Heavy Ion Research, Biophysics, Darmstadt, Germany

**Introduction:** 4D-optimization in lung cancer therapy typically results in ITV-like extended margins (4D-ITV), which increases OAR dose exposure. We propose 4D-optimized, conformal, motion-synchronized treatment plan libraries (4D-conformal) for higher conformity and improved OAR sparing.

**Materials and Methods:** In 4D-conformal IMPT, one plan is generated in each 4DCT phase to conformally cover the target. The resulting library of plans requires synchronization to respiratory motion during delivery. For both treatment modalities, robust worst-case optimization (±3mm setup and ±3.5% range uncertainty) is compared to conventional optimization (3mm isotropic margins). Plan quality is assessed in 21 dose scenarios combining setup and range uncertainties. The plans are evaluated for 5 complex NSCLC patients with ≥2 tumors (13 in total), exhibiting motion amplitudes up to 17mm. Prescription doses range from 1x20Gy to 5x7Gy (median: 1x24Gy), with priority on acceptable OAR sparing. Statistical significance is calculated with a Wilcox paired signed-rank test.

**Results:** Comparing conventional 4D-ITV and robust 4D-conformal plans (Fig.1), there was no significant difference in D95 target coverage, but CN significantly increased, which resulted in better OAR sparing for 4D-conformal plans (Fig.2). For robust 4D-conformal plans, the increased target coverage comes at cost of slightly lower OAR sparing.

**Conclusion:** Robust 4D-conformal IMPT offers significant reduction in lung and heart exposure while preserving target coverage.

## P147 -

### Cardiac sparing techniques for regional nodal irradiation in left-sided breast cancer patients treated with pencil beam scanning proton therapy

#### J. Isabelle Choi^1,2^, Weijun Xiong^1^, Chengyu Shi^1^, Julie Moreau^1^, Andy Shim^1^, Haibo Lin^1^, Jana Fox^1,3^, Shaakir Hasan^1,3^, Robert H. Press^1,4^, Richard Bakst^1,4^, Arpit M. Chhabra^1^, Charles B. Simone- II^1,2^, Oren Cahlon^1,2^

##### ^1^New York Proton Center, Radiation Oncology, New York, USA ^2^Memorial Sloan Kettering Cancer Center, Radiation Oncology, New York, USA ^3^Montefiore Medical Center, Radiation Oncology, New York, USA ^4^Icahn School of Medicine at Mount Sinai, Radiation Oncology, New York, USA

**Purpose:** Regional nodal irradiation (RNI) for the adjuvant treatment of locally advanced breast cancer poses a significant planning and delivery challenge, especially with the routine inclusion of the internal mammary chain. Pencil beam scanning (PBS) proton therapy (PT) allows for significant normal tissue sparing of thoracic organs-at-risk. We hypothesize that the use of deep inspiration breath hold (DIBH) may further enhance cardiac sparing for patients with left-sided breast cancer receiving RNI.

**Method/Materials:** Five consecutive patients treated with RNI for left-sided breast cancer using PBS PT who had DIBH and free breathing (FB) simulation scans available were identified at a single institution. CTV-based robust planning was employed. Treatment plans were developed using multi-field optimization with two fields (AP and en face) using a 5cm range shifter. Robust optimization parameters of 5mm for setup error and 3.5% for range uncertainty were utilized.

**Results:** CTV coverage was similar between the DIBH and FB scans. With DIBH, average maximum and mean heart doses decreased by 23% (FB 4770.2cGy vs. DIBH 3677.2cGy; p=0.005) and 36% (FB 144.9cGy vs. DIBH 92.5cGy; p=0.112), respectively. No other organs-at-risk were significantly different, with only 2.3% (p=0.244) and 2.7% (p=0.178) differences in left anterior descending artery maximum and mean doses, respectively, and 4.1% (p=0.450) difference in left lung mean dose.

**Conclusion:** Our preliminary study demonstrates that the use of DIBH for left-sided breast cancer patients may further enhance cardiac sparing of PT, with a significant reduction in maximum heart dose and a strong trend in reduction of heart mean dose.

## P148 -

### A temporal motion model for improving robustness of 4D optimized PBS plan: proof of concept

#### Y. Zhang^1^, N. Vatterodt^1^, S. Safai^1^, D. Weber^1^, A. Lomax^1^

##### ^1^Paul Scherrer Institut, Center for Proton Therapy, Villigen-PSI, Switzerland

**Aim:** By optimizing beam weight and delivery timeline according to a pre-treatment 4DCT, 4D optimized PBS proton plans that mitigate motion effects can be obtained (fig 1). However, such 4D optimized plans are highly sensitive to differences between motions assumed during optimization and those during delivery. We propose here a temporal motion model for guiding optimized 4D plan delivery, which mitigates such differences for motion variations within pre-defined uncertainty-bands.

**Methods:** The nominal feature motion representing a patient-specific breathing pattern was used to calculate a reference 4D optimized plan. The temporal motion model, in the format of uncertainty-bands, was defined for both amplitude and period around this feature motion (fig.2ab). This concept was investigated using three 4D geometric phantoms with varying nominal motion scenarios during 4D optimization. The obtained 4D plans were then recalculated by 10 different motion patterns within the uncertainty-band (fig2b). For exploring the limits of the 4D plan robustness, uncertainty-bands with differences up to +/-1s in period and +/-10mm in amplitude were evaluated. 4D optimization with/without rescanning were considered for all above scenarios.

**Results:** As fig2c shows, by applying the temporal motion model, comparable plan (within 10% of the nominal 4D optimized plan) can be achieved for varying motions occurring within uncertainty-bands of up to ±600ms (period) and ±4mm (amplitude), independent of geometry or initial motion. Combining rescanning into the 4D optimization can further reduce the impact of motion variations.

**Conclusions:** The temporal motion model is an effective approach to improve robustness of 4D optimized plan.

## P149 -

### Localization of possible morphological changes in patients during proton therapy by the in-beam INSIDE PET monitoring system.

#### Andrea Berti^1^, Aafke Kraan^2^, Alessandra Retico^2^, Giuseppe Battistoni^3^, Damiano Del Sarto^1,2^, Veronica Ferrero^4^, Elisa Fiorina^4,5^, Francesco Laruina^2^, Enrico Mazzoni^2^, Matteo Morrocchi^1,2^, Francesco Pennazio^4^, Valeria Rosso^1,2^, Giancarlo Sportelli^1,2^, Viviana Vitolo^6^, Giuseppina Bisogni^1,2^

##### ^1^University of Pisa, Physics, Pisa, Italy ^2^INFN, Physics, Pisa, Italy ^3^INFN, Physics, Milan, Italy ^4^INFN, Physics, Turin, Italy ^5^CNAO, Physics, Pavia, Italy ^6^CNAO, Medicine, Pavia, Italy

In-beam Positron Emission Tomography (PET) can be used for in-vivo non-invasive treatment monitoring in proton therapy. Even though PET monitoring has been frequently applied for this purpose, there is no straightforward method to translate the information from PET images into easy to interpret information for clinical personnel. The purpose of this work is to propose an innovative method for analyzing in-beam PET monitoring images to locate, quantify and visualize regions with interfractional anatomical changes occurring over the course of the treatment. We created a series of CT scans for a patient with squamous cell carcinoma treated with proton therapy, simulating the emptying of the sinonasal cavity. We performed multiple Monte Carlo simulations of the treatment using the geometry of the INSIDE detector installed at CNAO, and we investigated how the expected PET monitoring signal changes over time. Then we performed voxel-wise two-tailed statistical tests, resembling the Voxel-Based Morphometry method commonly used in neuroimaging, to determine whether the PET signal was compatible with expectations, or not. Disagreements between expectations and data results were visualized with specific colored patterns, that can be visualized overlaid onto the CT scan. Fig. 1 gives an example of such a pattern. In this work we present the most recent results of the application of this method.

## P150 -

### Flat panel proton radiography in high-precision image-guided mouse brain proton irradiation

#### Elisabeth Bodenstein^1^, Elke Beyreuther^1,2^, Johanna Bock^1^, Antje Dietrich^1,3^, Jörg Pawelke^1,4^, Michael Schürer^1,5^, Theresa Suckert^1,3^, Johannes Müller^1,4^, Armin Lühr^6^

##### ^1^OncoRay, National Center for Radiation Research in Oncology- Faculty of Medicine and University Hospital Carl Gustav Carus- Technische Universität Dresden- Helmholtz-Zentrum Dresden-Rossendorf, Dresden, Germany ^2^Helmholtz-Zentrum Dresden-Rossendorf, Institute of Radiation Physics, Dresden, Germany ^3^German Cancer Consortium DKTK, partner site Dresden- German Cancer Research Center DKFZ, Heidelberg, Germany ^4^Helmholtz-Zentrum Dresden-Rossendorf, Institute of Radiooncology - OncoRay, Dresden, Germany ^5^National Center for Tumor Diseases NCT, partner site Dresden- German Cancer Research Center DKFZ, Heidelberg, Germany ^6^TU Dortmund- Faculty of Physics, Medical Physics and Radiotherapy, Dortmund, Germany

Development of radiation therapy is strongly driven by in-vivo experiments, which are, however, subject to increasingly strict ethical and technical requirements. Proton irradiation of small animal organs demands for highly versatile experimental setups and intelligent protocols to mimic clinical workflows. To realize precise positioning and treatment, on-site imaging with proton radiography was integrated into an existing beam setup for mouse brain sub-volume proton irradiation at University Proton Therapy Dresden. A flat panel detector was installed on proton beam axis behind the mouse position. Transmission radiographic images were acquired at high energy (200 MeV) with a single-scattered proton beam. Image quality was optimized regarding resolution, contrast and minimal dose deposition in the animal. The hippocampus as target region for current mouse irradiation experiments was determined by registration of mouse brain atlas data with pre-treatment off-site CT scans and x-ray images. The developed workflow allows precise brain irradiation with lateral target positioning accuracy <0.2 mm. Imaging with dose depositions <20 mGy in mice was achieved. For accurate irradiation, the designated target volume (right hippocampus) was aligned with the collimated treatment beam by registering the radiography image with an off-site x-ray image with custom-made software (Figure 1). Immunohistochemically staining of DNA damage on histological whole-brain tissue sections validated successful mouse positioning and irradiation (Figure 2). In conclusion, proton radiography enables effective high-precision lateral alignment of proton beam and target volume in mouse irradiation experiments with limited dose exposure.

## P151 -

### Improving The Quality Of Proton Radiography Using Deep Learning: A Phantom Study

#### Serdar Charyyev^1^, Yang Lei^1^, Joseph Harms^1^, Bree Eaton^1^, Mark McDonald^1^, Jeffrey Bradley^1^, Jun Zhou^1^, Tian Liu^1^, Rongxiao Zhang^2^, Xiaofeng Yang^1^

##### ^1^Emory University, Radiation Oncology, Atlanta, USA ^2^Dartmouth College, Radiation Oncology, Lebanon, USA

Purpose: Proton radiography (PRG) is very valuable for beam's-eye-view target localization in proton radiotherapy, especially for shoot-through proton FLASH. However, PRG has very poor contrast and spatial resolution. To deal with this issue, we propose a machine-learning-based method to use kV digitally reconstructed radiograph (DRR) to improve PRG image quality. Methods: We used a residual attention cycle-consistent generative adversarial network framework to learn the nonlinear mapping between PRGs and DRRs. Residual blocks with attention gates were used to force the model to focus on the difference between the two images. Cycle-consistent generative adversarial networks were used to let the PRG-to-DRR mapping be close to a one-to-one mapping by introducing an inverse DRR-to-PRG mapping. To assess the accuracy of our method, we used a head-and-neck phantom with 179 PRG-DRR pairs from non-redundant angle projections using a double-scattering system. The DRRs acquired from CT acted as learning targets in the training process and were used to evaluate results from the proposed method using a six-fold cross-validation scheme. Results: The corrected PRGs showed an average peak signal-noise-ratio of about 16.82±0.52dB, an average normalized cross-correlation about 99.97±0.01% and an average structural similarity index of 90.61±0.07% compared to DRRs. The corrected PRG images have similar spatial resolution and contrast as a DRR.Conclusion: We proposed a novel deep-learning-based approach to improve the image quality for PRG. The results strongly indicate the high quality of PRG imaging by our method and show its potential feasibility for proton radiotherapy.

## P152 -

### Employing neuronal networks for efficient Monte Carlo simulation of the Imaging Ring system

#### H. Fuchs^1,2^, L. Zimmermann^1^, S. Alijani^1^, P. Kuess^1,2^, D. Georg^1,2^

##### ^1^Medical University of Vienna, Department of Radiation Oncology, Vienna, Austria ^2^MedAustron Iontherapy Center, Non-clinical research, Wr. Neustadt, Austria

**Introduction**: The Imaging Ring system (MedPhoton, Salzburg, Austria) is a versatile imaging solution installed at the MedAustron Iontherapy center (Austria). Full Monte Carlo (MC) simulations are slow and precalculating parts of a system and storing the output in phase space files is time consuming and creates large files (>5GB).We present a workflow for efficient MC calculations, starting with a full MC model, a subsequent training of a neuronal network to be used as a fast and convenient particle generator for efficient simulations.

**Material and Methods**: GateRTionv1.0/Geant4.10.3p3 was used to simulate the X-ray head of the Imaging Ring system based on the manufacturer documentation starting with the initial electron beam. The model was tuned to match experimental data such as HVL (Nomex, PTW, Freiburg, Germany) measurements. Simulations were performed for 60, 80, 100, and 120 kV. Photons exiting the X-ray tube were stored in a phase space file. A conditional Generative Adversarial Network (GAN) (pytorch-lightning v0.8.5, 3 layers, 400 neurons, RMSprop training function) modified from the gaga-phsp project[1] was trained using this phase space data as input (batch-size 10e4, 20 epochs).

**Results**: HVL values agree well with the experimental data with maximum deviations of 0.4mm. Training took less than 3 hours using a GTX2070 (Nvidia, Santa Clara, US). 1e7 photons were found to be sufficient for convergence with good data reproduction (Figure 1).

**Conclusion**: Introducing a conditional GAN into MC simulations allows to improve simulation efficiency as well as to reduce the cumbersome use of large phase space files.

### P153 - Impact of Contrast enhancement filter in automatic image registration in lung cancer

#### K. Hirotaki ^1^

##### ^1^National Cancer Center East Hospital, Radiorogy, Kashiwa, Japan

**Background**: Automatic image registration (AIR) performance is dependent on the image's feature and quality.

**Purpose**: To evaluate the effectiveness of using the contrast enhancement filter (CEF) to improve AIR accuracy in lung cancer.

**Method**: This study is a retrospective analysis of 9 lung cancer patients. The 2D-kV images were acquired with the projection angle 0, 90 degrees (AP), and 35, 125 degrees (Obl). Bone registration using 2D-kV image was performed pretreatment daily with the VeriSuite software (MedCom, Germany). The algorithms used in the software were based on a mutual information algorithm. Normalized mutual information (NMI) was used to evaluate the similarity. The accuracy of AIR with and without CEF was compared with the manual registration. The invalid ratio was calculated as the number of AIR invalid cases divided by the number of patients.

**Result**: The NMI was 0.58±0.07(mean±1SD), 0.46±0.08, with and without CEF, respectively. The mean translational difference was < 1.00 mm in all cases. The mean rotational difference (yaw/pitch/roll) was 0.15±0.17, 0.25±0.22, 1.17±1.13 and 0.22±0.30, 0.43±0.66, 0.76±1.13 degrees in AP images without and with CEF, and 0.24±0.35, 0.27±0.36, 0.84±1.01 and 0.28±0.39, 0.35±0.47, 0.53±0.63 degrees in Obl images without and with CEF. Invalid ratio was 56%,22%,22%,0% in AP without CEF, AP with CEF, Obl without CEF, Obl with CEF, respectively.

**Conclusion**: AIR with CEF would achieve higher accuracy in the lung region. Obl image with CEF reduced the rotation error improved and the robustness of AIR operation.

## P154 -

### Real-time surface-guided radiation therapy using multiple Azure Kinect

#### Chanil Jeon^1^, Sangwoon Jeong^1^, Wonjoong Cheon^2^, Youngyih Han^1^

##### ^1^Samsung medical center, Department of radiation oncology, Seoul, Korea Republic of ^2^National Cancer Center, Proton therapy center, Goyang, Korea Republic of

**Purpose**: Immobilizing and monitoring a patient in radiation treatment are important elements of accurate radiation therapy. Although the patient's movement is restricted mostly using immobilization devices, some unconscious motions in millimeters are to be expected. Mostly, RGB cameras are used for monitoring patient in clinic but this visual measurement method cannot verify a patient's small movement quantitively. Therefore, a system of real-time surface guided radiation therapy (SGRT) with multiple Azure Kinect was developed.

**Methods**: Two Azure Kinect (Kinect V4, Microsoft, USA) were used. Extrinsic calibration of the cameras was executed with a calibration checkerboard (60cm x 40cm, calib.io, Denmark) and OpenCV computer vision library. For surface visualization of patient, 3D point-cloud data (1280x720) of human body phantom (Atom phantom, CIRS Inc, US) were acquired real-time with Azure Kinect Development Kit. These data were compared to pre-saved 3D point-cloud data which have position before movement by each pixel with real-time concurrent operation using Nvidia CUDA Toolkit. Comparison result data was transferred to visualization process (OpenGL graphic library) as point-cloud color information.

**Results**: Patient surface position change was detected accurately by color difference in the visualization process of real-time surface-guiding system with Azure Kinect. The Proposed system detected phantom's position displacement over 2 mm stably.

**Conclusion**: Because the proposed system utilizes high resolution 3D point-cloud and general-purpose computing on graphics processing units (GPGPU) data handling by CUDA, delicate and fast patient monitoring of radiation therapy can be achieved.

## P155 -

### Microstructure characterization of skull-base chordoma for particle therapy from conventional diffusion MRI

#### Letizia Morelli^1^, Giulia Buizza^1^, Giulia Riva^2^, Giulia Fontana^3^, Sara Imparato^4^, Alberto Iannalfi^2^, Ester Orlandi^2^, Marco Palombo^5^, Chiara Paganelli^1^, Guido Baroni^1^

##### ^1^Politecnico di Milano, Department of Electronics Information and Bioengineering DEIB, Milan, Italy ^2^National Center of Oncological Hadrontherapy, Radiotherapists Unit, Pavia, Italy ^3^National Center of Oncological Hadrontherapy, Clinical Bioengineering Unit, Pavia, Italy ^4^National Center of Oncological Hadrontherapy, Radiology Unit, Pavia, Italy ^5^University College London, Department of Computer Science, London, United Kingdom

**Purpose**: To characterize tumour microstructure through conventional apparent diffusion coefficient (ADC) in skull-base chordoma (SBC) patients in proton and carbon-ion radiotherapy.

**Methodology**: Patients affected by SBC, who underwent diffusion-weighted MRI (DWI, b-values=50,400,1000 smm^−2^) before treatment and were enrolled for proton (n=25) or carbon-ion (n=26) therapy at the National Center of Oncological Hadrontherapy (CNAO), were retrospectively selected. Tumour volumes were propagated by rigidly registering planning CT contours on DWI. Clinically verified local control information (LC; median follow-up time: 61.3 months; overall LC=80.4%) was collected. From mono-exponential fits of DWI, ADC maps were estimated using different sets of b-values: b=50,1000, b=400,1000 and b=50,400,1000 smm^−2^. A published computational framework (doi:https://doi.org/10.1002/mp.14689) was employed to produce quantitative maps of cells' radius, volume fraction (vf) and mean diffusivity (D) from ADCs. Histogram-based metrics (mean, median, interquartile range, entropy) were considered for voxel-wise analyses. Logistic regression models were encapsulated in a leave-pair-out cross-validation routine to define the best patients' separation in terms of LC. Receiver operating characteristic (ROC) curves were built against LC for proton and carbon-ion patient groups, separately.

**Results**: From ROC analyses (Figure1), the highest area under the curve (AUC=0.96) for the carbon-ion group was given by vf entropy, with tumour heterogeneities being associated with opposite LC, and by median D for the proton group, with average tumour properties better explaining LC differences. ADC showed slightly inferior performance in carbon-ion (AUC=0.95) and proton (AUC=0.62) groups.

**Conclusions**: Estimation of microstructural parameters could inform upon changes in tumour microstructure occurring during particle therapy, implying advantages for patient stratification and treatment optimization.

## P156 -

### Advanced image guidance for upright particle radiation therapy treatment rooms.

#### Niek Schreuder^1^, Rock Mackie^2^, Stephen Towe^3^, Harry Freeman^2^

##### ^1^LEO Cancer Care, Medical Physics, Knoxville, USA ^2^Leo Cancercare, Medical Physics, Wisconsin, USA ^3^Leo Cancercare, Medicalo Physics, Surrey, United Kingdom

The benefits of particle beam imaging are well understood since the early days of particle beam therapy. The use of proton radiography (P-Rad) has been demonstrated in the 70's at the Harvard cyclotron laboratory and again in 1996 when the first proton P-Rad images on animals were obtained at the Paul Scherrer Institute in Switzerland. Despite of all the documented benefits P-Rad has not been implemented clinically at any particle therapy facility. Other forms of particle beam imaging include Positron Emission Tomography PET) and Prompt Gamma imaging which has been used clinically but not implemented on a significant scale. The main reason for the lack of widespread implementation of these important technologies is that it's very difficult and expensive to install these technologies in rotating gantries. Since the fixed beam rooms at the majority of proton therapy facilities are mainly used for prostate treatments, the need to install these technologies in existing fixed beam rooms was limited. Introducing upright treatments now changes this paradigm. Upright treatments using technologies such as the upright patient positioner and upright CT scanner being developed by Leo Cancer Care will allow for treating the majority of disease sites in fixed beam rooms. This in turn justifies the installation and clinical implementation of particle beam imaging technologies. We will show how P-Rad and Prompt PET imaging systems will be installed and implemented in the clinical workflow of future upright particle therapy treatment rooms equipped with the Leo upright patient positioners and upright CT scanners.

## P157 -

### Calibration setting sensitivity of water equivalent path length assessment via flat panel proton radiography

#### C. Seller Oria^1^, G. Guterres Marmitt^1^, J. Free^1^, J.A. Langendijk^1^, S. Both^1^, A.C. Knopf^1^, A. Meijers^1^

##### ^1^University Medical Center Groningen- University of Groningen, Radiation Oncology, Groningen, Netherlands

**Purpose:** Proton range uncertainties can compromise the effectiveness of proton therapy treatments. Water equivalent path length (WEPL) assessment using flat panel proton radiography (FP-PR) can provide means of range uncertainty detection. Given that WEPL accuracy assessment relies on the FP detector calibration, we investigated three calibrations to evaluate the sensitivity of WEPL accuracy with respect to different calibration configurations.

**Materials and Methods:** PR fields with multiple energy layers, directed towards water slabs of increasing thickness were simulated to obtain a calibration dataset. The calibration parameters investigated were the energy spacing between layers (dE) and the slab thickness increments (dX). Three configurations were tested: (a) with dE = 1 MeV and dX = 1 mm, (b) with dE = 3 MeV and dX = 2 mm, and (c) with dE = 5 MeV and dX = 5 mm. For an electron density phantom, WEPL values from FP-PR simulations using calibrations (a), (b) and (c) (Figure 1) were compared against WEPL values obtained from multi-layer ionization chamber PR simulations (ground truth). Relative WEPL errors for 16 tissue equivalent inserts were determined, as well as the mean relative WEPL error across all inserts for each calibration configuration.

**Results:** Mean relative WEPL error across all inserts in the phantom were -0.3% for (a) and (b), and -1% for (c) (Figure 2).

**Conclusion**: WEPL accuracy sensitivity to different FP-PR calibration configurations in terms of dE and dX indicates that a careful optimization of calibration settings is required to assure highly accurate WEPL assessment via FP-PR.

## P158 -

### Passive magnetic shielding for in-beam MR imaging during proton pencil beam irradiation

#### Ekaterina Semioshkina^1^, Bradley M. Oborn^1,2^, Aswin L. Hoffmann^1,3^

##### ^1^Helmholtz-Zentrum Dresden-Rossendorf, Institute of Radiooncology – OncoRay, Dresden, Germany ^2^University of Wollongong, Centre for Medical Radiation Physics CMRP, Wollongong, Australia ^3^Faculty of Medicine and University Hospital Carl Gustav Carus- Technische Universität Dresden, Department of Radiotherapy and Radiation Oncology, Dresden, Germany

**Introduction**: Dynamic magnetic fringe fields produced by the scanning magnets (SMs) of a proton pencil beam scanning (PBS) research beamline have shown to cause severe image artefacts during in-beam MR imaging [1]. In this study we investigate the effect of different design parameters of a passive magnetic shield positioned around the SMs on the reduction in magnitude of the magnetic fringe fields the SMs produce.

**Methods**: A finite element model of the PBS beamline was used to calculate the magnetic fringe fields produced by the SMs. Parameters investigated for a carbon steel magnetic shield included: geometry, material thickness, number of material layers, size of the air gap between layers. The shielding factor (SF) at the projected position of the MR isocenter was calculated. Previous measurements at our facility showed that a SF of at least 20 is required for artefact-free MR imaging during PBS dose delivery.

**Results**: A cost-efficient way to achieve the required SF was to use a multilayer cylindrical shield. A SF of 21 was achieved for two concentric layers of 10 mm thickness with a 10 mm air gap. The SF can be further increased to 25 by an additional layer. Setting the air gap equal to the layer thickness gave the highest shielding performance.

**Conclusion**: Computer simulations showed that a passive magnetic shield around the SM can provide the required SF using a multilayer cylindrical geometry with an interlayer air gap equal to the layer thickness.

## P159 -

### Evaluation of patient specific synthetic CT correction methods for lung tissue

#### Adrian Thummerer^1^, Paolo Zaffino^2^, Gabriel Guterres Marmitt^1^, Arturs Meijers^1^, Joao Seco^3,4^, Johannes Langendijk^1^, Antje Knopf^1,5^, Maria Francesca Spadea^2^, Stefan Both^1^

##### ^1^University Medical Center Groningen UMCG, Department of Radiation Oncology, Groningen, Netherlands ^2^Magna Graecia University Catanzaro, Department of Experimental and Clinical Medicine, Catanzaro, Italy ^3^Deutsches Krebsforschungszentrum DKFZ, Department of Biomedical Physics in Radiation Oncology, Heidelberg, Germany ^4^Heidelberg University, Department of Physics and Astronomy, Heidelberg, Germany ^5^Center for Integrated Oncology Cologne, Department I of Internal Medicine, Cologne, Germany

**Introduction:** In various anatomical locations (e.g., H&N, pelvis) deep convolutional neural networks (DCNN) have shown promising results for creating CBCT-based synthetic CTs (sCT). These sCTs can enable accurate proton dose calculations and thereby facilitate CBCT-based adaptive proton therapy workflows. However, in the thorax DCNNs fail to reliably generate accurate Hounsfield units (HU) for lung tissues affecting proton dose calculations. In this work we investigated the dosimetric impact of planning CT (pCT) based sCT-correction strategies.

**Methods:** A DCNN was used to generate CBCT-based sCTs for 33 lung cancer patients. Three techniques to correct sCT lung HUs were investigated. Method A utilizes deformable image registration to substitute HUs from the deformed lung region of the pCT. Method B uses a CBCT scatter correction technique, implemented in our treatment planning system (TPS, RayStation-9A), and applies it to the sCT. Finally, method C uses a smoothed difference map between sCT and the deformed pCT to improve HU accuracy of the sCT (see Fig1). To evaluate the dosimetric impact, we calculated doses on the sCTs and a same day verification CT for all 33 patients and performed gamma analysis (3%/3mm).

**Results:** Figure 2 shows a boxplot of gamma pass ratios for the uncorrected sCT and each of the correction methods. Average gamma pass ratios of 93.9±4.5%, 95.6±4.0%, 96.5±3.1% and 96.9±2.2% were observed for the uncorrected sCT and sCT_A,B,C_ respectively.

**Conclusion:** The investigated correction techniques all improved dose calculation accuracy on synthetic CTs. However, only method C resulted in clinically acceptable pass ratios above 90% for all 33 patients.

## P160 -

### A novel diaphragm based position verification protocol to improve daily target dose coverage in IMPT for distal oesophageal cancer

#### Sabine Visser^1^, Lydia A. den Otter^1^, Cássia O. Ribeiro^1^, Erik W. Korevaar^1^, Stefan Both^1^, Johannes A. Langendijk^1^, Christina T. Muijs^1^, Nanna M. Sijtsema^1^, Antje Knopf^1^

##### ^1^UMCG, Radiotherapy, Groningen, Netherlands

**Purpose:** When treating distal oesophageal cancer with intensity modulated proton therapy (IMPT), variations in the diaphragm position of the patient can cause underdosage of the target volume. We investigated whether target dose coverage can be maintained in case of diaphragm shifts by introducing a novel diaphragm based position verification (PV) method.

**Materials and Methods**: Weekly repeated 4DCTs of ten oesophageal cancer patients were rigidly aligned to the planning 4DCT using the vertebral column (PV_Bones_). Using this registration, inter-fractional cranial-caudal target displacements and their relation with diaphragm variations were investigated. This information served as an input to correct PV_Bones_ for the diaphragm position, resulting in PV_Diaphragm_. A third registration was performed based on the target volume (PV_Target_). Subsequently, for each registration method, target dose coverage of IMPT plans was robustly evaluated on the repeated 4DCTs.

**Results:** The cranial-caudal mean target displacement was congruent with nearly half of the diaphragm displacement (Fig. 1; y=0.439x), which was used for the diaphragm position correction in PV_Diaphragm_. Target dose coverage using PV_Bones_ was only adequate (D_98%_ ≥94%) when diaphragm displacements <12 mm were observed. For larger diaphragm displacements, the target dose coverage improved for PV_Diaphragm_ and PV_Target_, since target displacements were better accounted for (Fig. 2). An additional benefit was observed for PV_Diaphragm_, as the densities in the beam path were more similar to the planning situation.

**Conclusion:** If large displacements of the diaphragm are observed during PV, an adjustment of PV based on bony anatomy with half of the diaphragm displacement improves target dose coverage.

## P161 -

### Quantifying the dose uncertainties due to Bragg peak degradation induced by lung tissue in proton therapy of thoracic cancers

#### Kilian-Simon Baumann^1,2,3^, Veronika Flatten^1,2,3^, Uli Weber^4^, Matthias Witt^2^, Fabian Eberle^1,2^, Stefan Lautenschläger^1,2^, Rita Engenhart-Cabillic^1,2^, Klemens Zink^1,2,3^

##### ^1^University Medical Center Giessen-Marburg, Department of Radiotherapy and Radiooncology, Marburg, Germany ^2^Marburg Ion-Beam Therapy Center, Marburg Ion-Beam Therapy Center, Marburg, Germany ^3^University of Applied Scinences, Institute of Medical Physics and Radiation Protection, Giessen, Germany ^4^Helmholtzzentrum fuer Schwerionenforschung, Biophysics Division, Darmstadt, Germany

**Introduction**: Sub-millimetre-sized heterogeneities like lung tissue cause Bragg peak degradation. If not considered in treatment planning this can significantly influence the dose distribution in lung cancer patients. Until now there was no possibility to quantify these dose uncertainties since the lung tissue is not being resolved sufficiently in clinical CT images. In this study a method was developed to reproduce the Bragg peak degradation effects by applying a density modulation within the voxels associated with the lung making a determination of the corresponding dose uncertainties possible.

**Material/Methods**: Treatment plans of five lung cancer patients were optimized based on clinical CT data where the lung's fine structure is not resolved and hence Bragg peak degradation effects are not accounted for. These plans were then re-calculated using the Monte Carlo Code TOPAS. First, based on the original CT data. This dose distribution corresponds to the prediction from the treatment-planning system. In a second simulation, the density within the voxels associated with the lung was modulated, reproducing Bragg peak degradation effects and hence giving the realistic dose distribution in the patient.

**Results**: In table 1 dose differences in the CTV between the prediction from the treatment-planning system and the dose distribution in the patient are shown. The Bragg peak degradation leads to an underdosage of the CTV of up to 5%.

**Conclusion**: For the first time it is possible to estimate the dose uncertainties due to the Bragg peak degradation induced by lung tissue in proton therapy of lung cancer patients.

## P162 -

### Optimization of prompt gamma imaging and positron emission tomography system for range verification in carbon therapy: a Monte Carlo study

#### Bo-Wi Cheon^1^, Hyojun park^1^, Wook-Geun Shin^1^, Hyun-Joon Choi^2^, Hyun Cheol Lee^1^, Chul Hee Min^1^

##### ^1^Yonsei University, Department of Radiation Convergence Engineering, Wonju, Korea Republic of ^2^Severance Christian Hospital, Department of Radiation Oncology, Wonju, Korea Republic of

Prompt gamma (PG) imaging and positron emission tomography (PET) have been suggested to evaluate the particle beam range in real-time and after the treatment, respectively. The aim of this study is to design the optimal geometry for PG-PET system, which is capable for both PG and PET measurements, to be used in carbon therapy by using Geant4 Monte Carlo toolkit. The PG-PET system consisted of two-dimensional scintillator array and a parallel-hole collimator as shown in Fig 1. The optimal geometry of the PG-PET system was decided by delivering carbon beam to a rectangular PMMA phantom and comparing detection efficiency for PG and PE with different collimator hole sizes, collimator thicknesses, scintillator materials and scintillator thicknesses. For a quantitative evaluation, figure of merit was defined as a production of peak amplitude and ratio between the signal and noise, which follows the equation: FOM = Signal/Noise * peak. Further, 3D images for the PG and PE distributions were obtained with optimized PG-PET system. Among the nominates, the optimal thickness and hole size of the collimator were determined as 200 mm and 6 mm, respectively. For the scintillator, LaBr_3_ of 60 mm thickness was decided as the optimal. As shown in Fig 2, the 3D image was able to locate the Bragg-peak of the carbon beam within the difference of 2 mm compared to the actual dose distribution. In this study, the optimal geometry was decided for the PG-PET detection system. Experimental validation of the PG-PET system will be performed in future study.

## P163 -

### Investigating the feasibility of TOPAS for Monte Carlo track structure simulations of GEANT4-DNA example applications

#### L. Derksen^1^, T. Pfuhl^2^, R. Engenhart-Cabillic^3,4^, K. Zink^1,3,4^, K.S. Baumann^1,3,4^

##### ^1^University of Applied Sciences, Institute of Medical Physics and Radiation Protection, Giessen, Germany ^2^GSI Helmholtzzentrum für Schwerionenforschung GmbH, Biophysics division, Darmstadt, Germany ^3^University Medical Center Giessen-Marburg, Department of Radiotherapy and Radiooncology, Marburg, Germany ^4^Marburg Ion-Beam Therapy Center MIT, Department of Radiation Oncology, Marburg, Germany

**Purpose**: The purpose of this work is to investigate the feasibility of TOPAS-nBio for track structure simulations using tuple scoring and ROOT/Python-based post-processing.

**Material/Methods**: Examples of track structure simulations as they are included in GEANT4 were re-calculated with TOPAS. The results of both codes were compared to each other. The simulations contained investigations of different physics lists, calculation of energy-dependent range, stopping power, mean free path and w-value. The implementation of the examples in TOPAS was particularly interesting since the examples are not pre-programmed and no corresponding scorer is available. In TOPAS-nBio a tuple scorer was applied to save all relevant tracking information that were post-processed using ROOT and Python.

**Results**: In Figure 1, calculated ranges of electrons, protons and alpha particles simulated with GEANT4 and TOPAS as well as the deviations between both simulations are shown. Considering electrons, large relative deviation can be observed at small, absolute ranges. For electron energies above 500 eV, deviations are smaller than 2 %. Comparing proton ranges, deviations are high since different tracking cuts were used in both codes.The results of all simulations using TOPAS-nBio show in general deviations below 5% with those using GEANT4-DNA. Thus, we have presented a feasible way to implement the example applications included in GEANT4-DNA in TOPAS-nBio.

**Conclusion**: With our results we could show the potentials of applying the tuple scorer in Monte Carlo track structure simulations. Using this scorer, each relevant information of the track structure can be accessed, which can be analyzed as preferred after the simulation.

## P164 -

### Objective function for optimization of dose heterogeneity in spatially fractionated proton therapy.

#### Emil Fredén^1^, Anders Ahnesjö^2^

##### ^1^Stockholm University, Department of Physics, Stockholm, Sweden ^2^Uppsala University, Department of Immunology- Genetics and Pathology, Uppsala, Sweden

**Purpose**: The full-width-at-half-maximum of peaks, center-to-center spacing between peaks, and valley-to-peak-dose-ratio are normally reported as dose metrics in spatially fractionated radiation therapy (SFRT). These metrics are not well-defined for clinically relevant dose distributions. We propose a spatial frequency-based objective function as a dose heterogeneity measure for organs at risk (OARs) to facilitate automated optimization of clinically relevant SFRT dose distributions.

**Methods**: We computed a set of proton therapy dose distributions based on multi-aperture collimated fields for which the aperture dimensions and center-to-center spacing were varied (Picture 1). We used a box-shaped planning target volume (PTV) and a spherical OAR. PTV coverage- and hotspot constraints were introduced. A mask was used to select the OAR dose for simple computation of its objective function value. The dose distribution yielding the largest objective function value was considered optimal.

**Results**: Objective function values were found to increase with decreasing aperture dimensions and increasing center-to-center spacing. The optimum was found on the solution space boundary implied by the constraints (Picture 2).

**Conclusion**: Our objective function can facilitate automated optimization of entrance channel dose heterogeneity. It can be applied to arbitrarily shaped OARs and dose distributions produced by irregularly shaped fields.

## P165 -

### Challenges in Monte Carlo simulations as clinical and research tool in particle therapy: a review

#### Aafke Kraan^1^, Giuseppe Battistoni^2^, Silvia Muraro^2^

##### ^1^Istituto Nazionale di Fisica Nucleare- Sezione di Pisa, Physics, Pisa, Italy ^2^INFN Milano, Physics, Milano, Italy

The interest in Monte Carlo (MC) techniques in medical physics has been rapidly increasing in the past years. This is the case especially in particle therapy, where accurate simulations of different physics processes in complex patient geometries are crucial for a successful patient treatment and for many related research and development activities. Among the applications of MC simulations are dose calculations, design and commissioning of novel clinical facilities, shielding and radiation protection, commissioning of treatment planning systems, and prediction and interpretation of data for range monitoring strategies. While the use of general-purpose MC codes is primarily devoted to research, fast MC codes are getting more and more frequently used in clinical practice, especially in the form of specialized codes oriented to dose calculations. Despite the increased use of MC simulations for patient treatments, the existing literature suggests that there is still a number of challenges to be faced in order to increase the accuracy of MC calculations for patient treatments and to facilitate their use in clinics. In this presentation, we discuss some of the ongoing developments and challenges in this context. Undoubtedly, a multi-disciplinary approach is required to overcome many of the remaining challenges. Here we focus on a few specific topics that are of interest for particle therapy scientists:

- Developments in modelling of nuclear physics interactions (see figures 1 and 2).
- Developments in MC simulations including calculations of RBE, LET, and microdosimetry
- Developments in MC based treatment planning
- Developments in fast MC codes

## P166 -

### Evaluation of the organ specific secondary neutron dose in pencil beam scanning, double scattering and spatially fractionated proton therapy

#### Amelia Maia Leite^1^, Maria Giorgi^1^, Maria Grazia Ronga^1^, Yolanda Prezado^2^, Yoann Ristic^3^, Yann Perrot^3^, Francois Trompier^3^, Ludovic De Marzi^1,4^

##### ^1^Institut Curie- Radiation Oncology Department- Orsay- France, Centre de Protontherapie d'Orsay, Orsay, France ^2^Institut Curie, PSL Research University- University Paris Saclay- Inserm U 1021- CNRS UMR 3347, Orsay, France ^3^Institut de Radioprotection et de Sûreté Nucléaire, Service de Dosimétrie Externe, Fontenay-aux-Roses, France ^4^Institut Curie, PSL Research University- University Paris Saclay- Inserm LITO, 91898- Orsay, France

**Purpose**: The Center of Protontherapy of Orsay (ICPO) has a long history of intracranial treatments both with the double scattering (DS) and pencil beam scanning (PBS) techniques, and is actively investigating a promising spatial fractionated radiotherapy using proton minibeams (pMBRT). This work shows a comprehensive comparison of the organ specific secondary neutron dose due to each of these treatment modalities, assessed using Monte Carlo (MC) algorithms.

**Methods**: A MC model of a universal nozzle was benchmarked by comparing the neutron ambient dose equivalent (H*(10)) in the gantry room with respective measurements obtained using a WENDI—II counter. The secondary neutron dose was evaluated for clinically relevant intracranial treatments of patients in different age groups, in which the out-of-field doses were scored in anthropomorphic phantoms merged to the images of the patients.

**Results**:The MC calculated H*(10) values show a good agreement with the measurements and follow the expected tendency in which PBS yields the lowest dose, followed by pMBRT and DS (Figure 1). For the intracranial treatments the estimated in-field secondary neutron dose ratio of pMBRT to PBS is 3.7±0.6 and the ratio of pMBRT to DS dose is 2.6±0.1, while the out-of-field neutron dose ratios were evaluated to be 13±2 and 0.7±0.1, respectively.

**Conclusion**: This work stands to be the first that realistically quantifies the secondary neutron dose contribution of pMBRT clinical treatments. The established method will enable epidemiological studies of the long term effects of intracranial treatments at ICPO, notably radiation induced second malignancies.

## P167 -

### Emulating BLOB (“Boltzmann-Langevin One Body”) a low energy nuclear reaction model with Deep Learning algorithms

#### Lorenzo Rossi^1^, Giulia Tuttobene^1^, Barbara Caccia^2^, Andrea Ciardiello^1^, Giuseppe A. P. Cirrone^3^, Maria Colonna^3^, Andrea Dotti^4^, Riccardo Faccini^1^, Stefano Giagu^1^, Paolo Napolitani^5^, Luciano Pandola^3^, Cecilia Voena^6^, Carlo Mancini Terracciano^1^

##### ^1^Sapienza Università degli Studi di Roma, Dipartimento di Fisica, Roma, Italy ^2^Istituto Superiore di Sanità, National Center for Radiation Protection and Computational Physics, Rome, Italy ^3^INFN, Laboratori Nazionali del Sud, Catania, Italy ^4^SLAC, none, Menlo Park CA, USA ^5^Universite Paris-Saclay- CNRS/IN2P3, IJCLab, Orsay, France ^6^INFN, Roma1, Rome, Italy

Monte Carlo (MC) simulations are of utmost importance in Ion-therapy and for such applications the nuclear interaction models are crucial. Geant4 is one of the most widely used MC toolkit, also for Ion-therapy simulations. However, recent literature has highlighted the limitations of its models in reproducing the yelds of secondaries measured in ions interaction below 100 MeV/n. To mitigate such a shortcoming, we interfaced a model dedicated to these reactions, BLOB (“Boltzmann-Langevin One Body”), with Geant4 obtaining promising results. However, the BLOB computation time is of the order of several minutes per interaction. Even if from the BLOB final state it is possible to sample many physical states, such a running time is too large for any practical application. To overcome this limitation, we tested several generative Deep Learning (DL) algorithms to emulate the BLOB final states. The figure shows the latent space of one of the selected algorithms. The colors denote the impact parameter of each event. The fact that events with similar impact parameter are nearby indicates that the algorithm has correctly classified such events.

The obtained double differential cross sections of fragment production in the interaction of a 12C beam at 62 MeV/u with a thin carbon target are comparable with the ones obtained coupling BLOB with Geant4 therefore we can have the BLOB full model reliability without its computational overhead. We are working to interface the generative part of the DL algorithm to Geant4 to use it as a nuclear reaction model.

## P168 -

### Dose calculation accuracy in particle therapy: A comparative study of protons and carbon ions in RayStation TPS

#### Sirinya Ruangchan^1,2^, Hugo Palmans^3,4^, Barbara Knäusl^1,3^, Dietmar Georg^1,3^, Monika Clausen^1^

##### ^1^Medical University of Vienna, Department of Radiation Oncology, Vienna, Austria ^2^King Chulalongkorn Memorial Hospital, Department of Radiology, Bangkok, Thailand ^3^MedAustron Ion Therapy Center, Division of Medical Physics, Wiener Neustadt, Austria ^4^National Physical Laboratory, Radiation Dosimetry, Teddington, United Kingdom

An assessment of dose calculations for protons^1^ and carbon ions is presented focusing on the comparison of both particle types. Spot maps were optimized and the resulting final dose distributions were calculated in the treatment planning system RayStation (RaySearch, Sweden), employing the analytical pencil beam (PB) algorithm for carbon ions (c-PB, v3.0) and protons (p-PB, v4.2), as well as the Monte Carlo algorithm for protons (p-MC, v4.1). Calculations were benchmarked against measurements with PinPoint ionization chambers (PTW, Freiburg) in a water phantom with embedded tissue-mimicking inhomogeneities (bone, lung, soft tissue). Dose calculation performance was evaluated inside and outside of target volumes. Different configurations for clinically relevant beams with and without range shifter and different air gaps were investigated (Figure1). The differences inside the target, averaged over all investigated configurations, were similar for all algorithms: 1.0% for c-PB, 1.3% for p-PB and 0.9% for p-MC. The largest local dose underestimation of 18.8% was observed for p-PB behind the target (Figure 2). The c-PB dose calculations in heterogeneous geometries were more accurate compared to the p-PB calculations in configurations without a range shifter (inside and outside the target). For c-PB the dose distributions in the target were in line with the results from p-MC calculations. However, local dose overestimation of more than 10% were found at distances of up to 1.5 cm behind the target for bone-lung structures, which might contribute to incorrect dose predictions for critical organs and lead to increased risk of side effects.

## P169 -

### Validation of dose-averaged LET computed in RayStation TPS against Gate/Geant4 for carbon ion beams

#### Mansure Schafasand^1^, Andreas Resch^2^, Erik Traneus^3^, Lars Glimelius^3^, Piero Fossati^4^, Markus Stock^1^, Antonio Carlino^1^

##### ^1^EBG MedAustron GmbH, Medical Physics, Wiener Neustadt, Austria ^2^Medical University of Vienna, Radiation Oncology, Vienna, Austria ^3^RaySearch Laboratories AB, Physics, Stockholm, Sweden ^4^EBG MedAustron GmbH, Medical, Wiener Neustadt, Austria

The high linear energy transfer (LET) in carbon ion beams compared to protons is one of the main motivations to use these ions in particle therapy. The quality of charged particle beams is often quantified by LET reported as its first or second moments of the fluence spectrum, track- or dose-averaged LET (LET_d_) respectively. This study was conducted to evaluate the LET_d_-scoring functionality of a non-clinical version of the RayStation (RS, RaySearch Laboratories) treatment planning system for carbon ion beams. For this study, three carbon ion plans were designed, containing a 4x4x4 cm^3^ target centered at depths 5, 12 or 24 cm in water. The LET_d_ was computed in a research version of RS 9A. The dose grid resolution in depth was set to 1 mm. The same setup was simulated in Gatev9.0/Geant4.10.6.p01 (G4) and the LET_d_ (for carbon and all fragments with atomic number Z≤6) was compared in depth. For future LET-based optimization, the LET-based filtered dose was additionally benchmarked. Negligible LET_d_ (of carbon and all fragments) difference was observed in low gradient regions. A larger difference (±8%) between RS and G4 was found on the target edges, where high dose- and LET-gradients were present (Figure 1). The agreement of the LET-based filtered dose was similar (Figure 2). A good agreement was observed between the computed LET_d_ in RS and G4. Hence, the LET_d_-scoring functionality provided in RS can be reliably applied for future studies focusing on LET-based optimization for carbon ion beams.

## P170 -

### Virtual Particle Monte Carlo (VPMC), a new concept to avoid simulating secondary particles in proton therapy dose calculation

#### Jie Shan^1^, Mirek Fatyga^1^, Wei Liu^1^

##### ^1^Mayo Clinic Arizona, Radiation Oncology, Phoenix, USA

**Purpose**: In proton therapy dose calculation, conventional Monte Carlo (MC) simulation may not take full advantage of GPUs because the random secondary-particle-generating processes result in unbalanced workload for different threads of GPUs. Therefore, we propose a novel concept of virtual particle (VP) in MC (VPMC) to avoid simulating secondary particles with clinically acceptable accuracy in proton therapy dose calculation.

**Method**: The dose of one primary proton and its secondaries, named realistic particles, in conventional MC codes, can be recalculated by simulating multiple VPs. Each VP corresponds to one realistic particle. However, VPs start at the same starting position of the primary proton. After converting the histories of realistic particles into histories of VPs that generate the same dose as realistic particles, we can get a probability model to describe the behaviors of VPs. The model includes a continuing-slowing-down-approximation model and a large angle event model corresponding to nuclear interactions. To simplify the job, we ignored neutrons and gamma rays, locally deposited the dose of electrons, heavy ions and nuclear fragments, and converted the tracks of deuterons into tracks of protons. Then we calculate dose by simulating only VPs.

**Results**: In this study we implemented a simple VP model and benchmarked it with MCsquare. The 3D Gamma analysis (1%/1mm)/(2%/2mm) against MCsquare is 97.8%/99.4% in water phantoms and 96.5%/99.4% in inhomogeneous phantoms, respectively. The calculation is highly efficient ( particles per second with 6 Nvidia T4 GPUs).

**Conclusions**: VPMC achieved high accuracy and high efficiency in proton therapy dose calculation.

## P171 -

### Optimization of bolus shape for boron neutron capture therapy using epi-thermal neutron beam

#### Takushi Takata^1^, Hiroki Tanaka^1^, Yoshinori Sakurai^1^, Akinori Sasaki^2^, Minoru Suzuki^1^

##### ^1^Kyoto University, Institute for Integrated Radiation and Nuclear Science, Osaka, Japan ^2^Kyoto University, Graduate School of Engineering, Kyoto, Japan

In Boron Neutron Capture Therapy (BNCT), epithermal neutron beam has been utilized to treat a deep-seated tumor due to its high penetration ability based on the thermalization within a patient. However, the thermal neutron buildup causes dose deficiency in a case that a tumor locates in a vicinity of a patient surface. In such a case, a bolus consisting of hydrogen-rich material has been used to improve the dose distribution. In present clinical BNCT, a bolus with a uniform thickness and a simple shape is adopted. Aiming to more aggressively increase tumor dose, a method to optimize the bolus shape was studied. The optimization was performed as following. First, a beam direction was determined according to location of PTV and OAR. Then, the control points defining the bolus thickness were assigned on the beam incident surface of patient. The bolus region was modeled in the patient geometry by interpolating the thicknesses defined at the control points. The thicknesses at the control points were adjusted to maximize the PTV dose under the OAR dose regulation through the optimization calculation. The Simulation Environment for Radiotherapy Applications (SERA), a treatment planning system for BNCT, was used for modeling of patient geometry including the bolus and dose calculations throughout the optimization process. As an example, a case for parotid cancer extended to subcutaneous region was examined. Increase in tumor dose was confirmed by using the bolus designed by the optimization method. The detailed results including further examination will be reported.

## P172 -

### The effect of difference in range uncertainty definition on dose distribution

#### Ivanka Sojat Tarp^1^, Kenneth Jensen^1^, Vicki Trier Taasti^2^

##### ^1^Aarhus University Hospital, Danish Center for Particle Therapy, Aarhus, Denmark ^2^Maastricht University Medical Centre+, Department of Radiation Oncology MAASTRO- GROW – School for Oncology, Maastricht, Netherlands

**Purpose:** Different strategies are used to incorporate range uncertainties into proton treatment planning. The aim of this study is to investigate the effect on dose distribution when 3.5% range uncertainty is added to either CT numbers or stopping power ratios (SPRs).

**Methods:** Treatment plans were created for patients with head-and-neck (HN), liver and prostate cancer. Each treatment plan was recalculated adding positive and negative range uncertainties of 3.5% to either the CT number or SPR to compare the two uncertainty evaluation strategies. Recalculated dose distributions were evaluated by subtracting the CT number manipulated calculation from the SPR manipulated calculation for both negative and positive range uncertainties. Difference in target dose for the two uncertainty evaluation strategies was calculated. To investigate differences between the results for the three treatment sites, the amount of air/lung, soft and bone tissue that the beams passes through was extracted.

**Results:** There was a small dose difference between dose evaluations whether the range uncertainty was added to the CT number or SPR. The largest difference was observed around the tumor where the dose was highest (Figure 1). Average dose difference for the target and the amount of lung tissue traversed by the beams was substantially higher for the liver plan (Table 1).

**Conclusion:** Adding range uncertainty to the CT number or SPR introduces a dose distribution difference. Even though the difference is minor, considerations must be made when choosing the range uncertainty strategy, and values cannot simply be taken from the literature without context.

## P173 -

### A fast deterministic method for calculating doses and fluence spectra for proton beam therapy

#### O. Vassiliev^1^, S. Kry^1^, C. Peterson^2^, D. Grosshans^3^, R. Mohan^1^

##### ^1^The University of Texas MD Anderson Cancer Center, Department of Radiation Physics, Houston, USA ^2^The University of Texas MD Anderson Cancer Center, Department of Biostatistics, Houston, USA ^3^The University of Texas MD Anderson Cancer Center, Department of Radiation Oncology, Houston, USA

Treatment plan optimization in proton beam therapy involves multiple calculations of spatial distributions of physical dose and relative biological effectiveness (RBE). We develop a novel fast and accurate method for calculation of doses and fluence spectra. Fluence spectra provide physical data that is sufficient for RBE calculations using almost any of the current models. Our method is deterministic, it does not involve any random sampling. It is based on exactly the same physics as the most rigorous Monte Carlo algorithms, which ensures high accuracy of our method. Monte Carlo algorithms rely on multiple scattering models to generate random particle trajectories. Our algorithm uses the same models, but differently, to find the solution using an iterative procedure. By eliminating random sampling, we achieve high computing speed. Our algorithm accounts for: energy loss straggling using Vavilov distribution, inelastic nuclear reactions using the cross sectional data of Geant4 software, and particle trajectory curvature using tabulated detour factors from PSTAR database. We have tested our algorithm for 40 to 220 MeV protons propagating in water. The CPU time was 1.6-3.8 sec on an HP workstation. Our method agreed with MCNPX 2.7.0 Monte Carlo code. In the plateau region the maximum difference in dose (Gy/particle) was 2% and the rms was 0.3%. The maximum distance to agreement near the Bragg peak was 0.8 mm. The maximum difference in the energies of fluence spectra maximum was 0.6 MeV and the maximum discrepancy in fwhm was 0.9 MeV. These spectra comparisons included depths around the Bragg peak.

## P174 -

### Effects of multi scanning patterns with different spot sizes and spacings on lateral dose distribution for proton radiotherapy

#### Meiling Xu^1^, Michael Moyers^2^, Jun Zhao^3^, Liu Hong^4^

##### ^1^Cancer Hospital Chinese Academy of Medical Sciences- Shenzhen Center, Radiation Therapy Dept., Shenzhen, China ^2^Shanghai Proton and Heavy Ion Center, Medical Physics, Shanghai, China ^3^Fudan University Shanghai Cancer Center, Radiation Therapy, Shanghai, China ^4^Yibiya Beijing Granule Accelerator Technology Co.-Ltd., Radiation Therapy, Shanghai, China

**Purpose**: To investigate how the spot sizes and spacings with different types of spot patterns affect the lateral dose distribution for modulated scanning proton beams.

**Methods**: Three types of spot patterns (rectangular, hexagonal and contour-guided) were simulated to produce dose distribution with different spot spacings and sizes. The ratio c (c=spot spacing/FWHM) was varied from 0.1 to 1.0 to evaluate the impact of the beam spot size and spacing on the homogeneity of lateral dose distribution. The single beam spot kernel was generated by using a double Gaussian model based on the measurement of EDR2 film.

**Results**: Under ideal conditions, the homogeneity can be within ±3% when c ≤ 0.85 for rectangular, c ≤ 0.97 for hexagonal and c ≤ 0.4 for contour-guided array of spots. For large spacing, the flatness is better with a hexagonal pattern than for a rectangular pattern; for small spacing (c < 0.6), the flatness is approximately the same for both patterns. For all spacings, the number of spots is less for rectangular patterns than for hexagonal patterns. When physical uncertainties are simulated, hexagonal patterns were more robust than rectangular ones.

**Conclusions**: To achieve good flatness with a large spot spacing, hexagonal patterns are recommended but to minimize the number of spots rectangular patterns are recommended. To produce a lateral dose distribution that is more conformal to the target, a contour-guided scanning pattern may be used but only if intensity modulated optimization is also implemented.

## P175 -

### PRO-GLIO: PROton versus photon radiotherapy in patients with diffuse grade II and III GLIOmas - a randomized study

#### Petter Brandal^1^, Malin Blomstrand^2^, Katja Werlenius^2^, Hillevi Rylander^3^, Tonje Haug Nordenmark^4^, Cecilie Kiserud^1^

##### ^1^Oslo University Hospital, Oncology, Oslo, Norway ^2^Sahlgrenska University Hospital, Oncology, Göteborg, Sweden ^3^The Skandion Clinic, Oncology, Uppsala, Sweden ^4^Oslo University Hospital, Physical Medicine and Rehabilitation, Oslo, Norway

**Background**: Proton therapy holds promise of less radiotherapy related (late) side effects because of the ability to spare non-neoplastic tissue surrounding the radiotherapy target volume. Considering the brain as an organ vulnerable to radiotherapy, this promise is very attractive for cerebral neoplasms. Diffuse grade II and III gliomas which include astrocytomas and oligodendrogliomas, however, pose a particular challenge. Because of their diffusely infiltrative nature it is pertinent to ask if better radiotherapy dose sparing of brain tissue outside the radiotherapy target volume will lead to shorter survival. Will the use of proton therapy hunting for a better quality of survival and lowered risk of unwanted late effects compromise overall survival?

**Methods**: A randomized open-label multicenter phase III study - PRO-GLIO - is planned. 225 patients with IDH-positive diffuse grade II and III gliomas (oligodendrogliomas and astrocytomas) will be randomized 1:1 to proton or photon radiotherapy. IDH-positivity is chosen as an inclusion criterium to ensure a patient cohort with favorable median overall survival. The primary endpoint is next intervention free survival (NIFS) and the two key secondary endpoints are fatigue (PROMS-based) and the neurocognitive general ability index (GAD). Further secondary and explorative endpoints include overall survival, progression-free survival as measured by RANO, health-related quality of life, local control/pattern of relapse, and the health economical parameter incremental cost effectiveness ratio.

**Aim/conclusion**: PRO-GLIO aims at establishing proton therapy as a safe treatment modality for IDH-positive diffuse gliomas, as well as proving protons ability to reduce unwanted late effects in this patient group

## P176 -

### Evaluation of radiologic changes and clinical outcome in children with brainstem glioma who received proton vs. photon therapy

#### Youngkyong Kim^1^, Joo-Young Kim^2^

##### ^1^Kyung Hee University Hospital, Radiation Oncology, Seoul, Korea Republic of ^2^Research Institute and Hospital- National Cancer Center, Proton Therapy Center, Goyang, Korea Republic of

**Purpose:** To assess the radiologic response and clinical outcome in pediatric brainstem glioma treated with proton and x-ray radiation therapy (PRT and XRT).

**Materials and methods:** Between September 2003 and December 2018, 34 children with received PRT (n=14) and XRT (n=20) for brainstem glioma. The prescribed dose was median 56 Gy (range, 50–60 Gy) with daily 1.8-2.4 Gy. Change of enhancement pattern and cystic portion was evaluated in brain magnetic resonance (MR) within 6 months of RT finish. PostRT necrosis was classified into 4 levels; no, mild, moderate, and severe.

**Results:** Median follow-up duration was 9.6 months (range, 2.5-137.4). The age at RT was median 6 years (range, 3-18). There was no difference in the incidence of postRT necrosis and survival between the PRT and XRT groups. Concurrent or adjuvant chemotherapy, hydrocephalus, and intervention for hydrocephalus didn't influence on postRT necrosis. Regardless of RT modality, children with initial enhancement and necrosis/cystic portion of preRT MR showed more postRT moderate to severe necrosis (p=0.007 and p=0.069, respectively) and those without postRT necrosis suggested prolonged overall survival [median 19.5 months (95% CI, 2.7-36.3) vs. 10.8 months (95% CI, 6.3-15.4), p=0.022)].

**Conclusions:** PreRT MR finding and postRT necrosis would be useful in predicting patient with favorable survival for pediatric brainstem glioma.

## P177 -

### Early outcomes following proton therapy for low-grade gliomas in adults

#### Konrad Urbanek^1^, Dominika Wojton-Dziewońska^1^, Olga Urbanowicz^1^, Agnieszka Chrostowska^1^, Elżbieta Pluta^1^, Anna Patla^1^, Kamil Kisielewicz^2^, Eleonora Góra^2^, Mariusz Wszołek^1^, Damian Kabat^2^, Renata Kopeć^3^, Tomasz Skóra^1^

##### ^1^Maria Sklodowska-Curie National Research Institute of Oncology, Department of Radiotherapy, Kraków, Poland ^2^Maria Sklodowska-Curie National Research Institute of Oncology, Department of Medical Physics, Kraków, Poland ^3^Institute of Nuclear Physcics Polish Academy of Sciences, Bronowice Cyclotron Center, Kraków, Poland

**Purpose**: The aim of the study is to describe early clinical outcomes in patients treated with spot-scanning beam proton radiotherapy for low grade gliomas.

**Methods and Materials**: We analyzed the outcomes of pencil beam scanning proton beam therapy of 86 patients with low grade gliomas treated at our facility from December 2016 to December 2019. Mean age at diagnosis was 41 (range 19-76). 49.4% of patients were female. Median dose delivered was 54 GyRBE in 30 fractions. Prior to irradiation 68.6% of the patients underwent at least partial resection and in 31.4% of patients stereotactic biopsy was performed. Most common histopathologic subtype was diffuse astrocytoma (50% of patients). For each patient, we recorded tumor location, extent of resection, dose-volume comparison with alternative photon plan and use of adjuvant chemotherapy. Acute and late toxicity scores were recorded using CTCAE version 5.0. Maximum grade of fatigue, headache, insomnia, nausea, vomiting, alopecia, dermatitis and need for steroids over the radiation therapy treatment course were recorded, and rates of acute toxicity were calculated.

**Results**: After a median follow-up of 23.6 months 13 failures were observed. The mean time to recurrence was 18.8 months (range, 6.0-55.4 months). 3-year progression-free survival and 3-year overall survival rates were 81.0% and 89.1%, respectively. There were neither acute nor late G3 toxicities due to radiotherapy.

**Conclusion**:Proton radiotherapy is an effective and safety treatment for low grade gliomas. Despite the high volumes of irradiated brain, the toxicity of treatment is low. The results are encouraging, and further follow-up is pending.

## P178 -

### Adjuvant proton beam therapy in skull base chordoma

#### Katarzyna Holub^1^, Sébastien Froelich^2^, Philippe Herman^3^, Alexandre Carpentier^4^, Loic Feuvret^5^, Guillaume Lot^6^, Stéphanie Bolle^7^, Claire Alapetite^8^, Sylvie Helfre^8^, Farid Goudjil^8^, Isabelle Pasquie^8^, Catherine Nauraye^8^, Valentin Calugaru^8^, Remi Dendale^8^, Hamid Mammar^8^

##### ^1^Institut Curie- Universitat de Barcelona- SEOR-CRIS Foundation- Spain, Department of Radiation Oncology, Paris, France ^2^Hôpital Lariboisière, Department of Neurosurgery, Paris, France ^3^Hôpital Lariboisière, Head and Neck Department, Paris, France ^4^Hôpital La Pitié Salpêtrière, Department of Neurosurgery, Paris, France ^5^Hôpital La Pitié Salpêtrière, Department of Radiation Oncology, Paris, France ^6^Fondation Rothschild, Department of Neurosurgery, Paris, France ^7^Gustave Roussy, Department of Radiation Oncology, Villejuif, France ^8^Institut Curie, Department of Radiation Oncology, Paris, France

**Background:** Chordomas are rare malignant tumors of the axial skeleton. Due to their invasive character, microscopic resection in skull base (SB) located lesions is usually impossible and adjuvant Proton beam (PB) alone or in combination with photon beam (PhB) radiotherapy is recommended to prevent recurrence.

**Methods:** A total of 130 consecutive patients (67 females, 63 males) with SB chordoma treated with PB alone (n=119) or in combination with PhB (n=11) between 2013 and 2018 were retrospectively reviewed. Median age was 53.3 years old (range: 18.2-85.0), median dose was 73.8 Gy RBE (range: 68.0-75.6). One-hundred twenty six patients underwent upfront surgery with a total resection in 38 cases, four patients had only biopsy; 107 patients had a tumor diameter <5 cm.

**Results:** After the median follow-up of 38.5 months (range: 1.1-92.3), 12 deaths and 18 local relapses were reported, resulting in 90.8% OS and 86.2% LC rate. Actuarial 5-year OS rate was 88%. Female gender (p=0.023), tumor diameter ≥ 5cm (p=0.011) and dual component irradiation [PB+PhB] (p=0.036) were associated with worse OS. Only grade 1-3 toxicities were observed.

**Conclusions**: PB allows high-dose radiotherapy in radio-resistant SB chordomas that are close to radiosensitive brain structures, resulting in high control rate and low toxicity.

## P179 -

### Preliminary outcomes of skull-base chondrosarcoma patients treated with pencil-beam scanning proton therapy.

#### Tomasz Skóra^1^, Dominika Wojton-Dziewońska^1^, Agnieszka Chrostowska^1^, Elżbieta Pluta^1^, Anna Patla^1^, Kamil Kisielewicz^2^, Eleonora Góra Eleonora Góra^2^, Konrad Urbanek^1^, Emilia Krzywonos^1^, Tomasz Kajdrowicz Tomasz Kajdrowicz^3^, Damian Kabat Damian Kabat^2^

##### ^1^Maria Sklodowska-Curie National Research Institute of Oncology, Department of Radiotherapy, Kraków, Poland ^2^Maria Sklodowska-Curie National Research Institute of Oncology, Department of Medical Physics, Kraków, Poland ^3^Institute of Nuclear Physcics Polish Academy of Sciences, Bronowice Cyclotron Center, Kraków, Poland

**Purpose**: The purpose of this study was to present early clinical outcome of spot-scanning beam proton radiotherapy for patients with skull-base chondrosarcoma.

**Methods and Materials**: The study group comprised 20 patients with histologically confirmed chondrosarcoma of the base skull with a median age of 49 years (range, 22-68 years). They were treated with proton radiotherapy in the Krakow Branch of the Maria Sklodowska-Curie *National Research Institute* of *Oncology from November 2016 to December 2019*. The median delivered dose was 70.0 GyRBE (range, 70.0-74.0 GyRBE). Most oft he patients (90.0%) underwent upfront surgery. Local failure-free survival and overall survival were calculated. The treatment toxicity was evaluated according to CTCAE (version 5.0)

**Results**: After a median follow-up of 27.9 months (range, 4.6-46.4 months), neither any local nor distant failures were observed. At the time of last follow-up all patients were alive. High-grade (G3) acute and late radiation-induced toxicity was observed in 1 and 2 patients, respectively.

**Conclusion**: High-dose pencil beam scanning proton therapy in the management of skull base chondrosarcoma is an effective treatment with acceptable toxicity profile. The longer follow-up period is needed to confirm presented results.

## P180 -

### Visual outcomes after proton therapy for uveal melanoma

#### Nanda Horeweg^1^, Lennart J. Pors^1^, Femke P. Peters^1^, Ann Schalenbourg^2^, Alessia Pica^3^, Jan Hrbacek^3^, Khan H. Vu^4^, Jaco C. Bleeker^4^, Coen R.N. Rasch^1^, Martine J. de Jager^4^, Gré P.M. Luyten^4^, Marina Marinkovic^4^

##### ^1^Leiden University Medical Center, Radiation Oncology, Leiden, Netherlands ^2^University of Lausanne- Jules-Gonin Eye Hospital, Department of Ophthalmology-, Lausanne, Switzerland ^3^Paul Scherrer Institute- ETH Domain, Radiation Oncology, Villigen, Switzerland ^4^Leiden University Medical Center, Ophthalmology, Leiden, Netherlands

**Objective**: To assess visual outcomes after proton therapy for uveal melanoma and to determine risk factors for loss of vision acuity.

**Methods**: This is a retrospective study of Dutch uveal melanoma patients treated with proton therapy (60.0 CGE in 4 fractions) abroad (mainly Switzerland), from 1987-2019. Actuarial estimates of visual acuity and secondary enucleation were calculated according to Kaplan-Meier's methodology. Risk factors for loss of vision <0.2 were identified using Cox' proportional hazards models. Visual acuity over time was analysed by hierarchical clustering.

**Results**: There were 103 patients (104 eyes); mean age was 58 years (range 24-85). Median tumor diameter and height were 18.7 (IQR 3.8) and 8.4 mm (IQR 3.2) respectively (median volume 1163mm^3^, IQR 823). Tumours were localized centrally (10.6%), mid-peripherally (65.4%) and peripherally (33.7%). Tumour stages were T1-2 (11.6%), T3 (26.0%) and T4 (62.5%). Median follow-up was 7.0 years (SE 1.0); 20.1% (SE 4.3%) of the eyes were enucleated at 5 years. Visual acuity over time is presented in table 1. Pre-treatment retinal detachment (p=0.005), tumour volume (p=0.001), central localisation (p=0.039), retina D90 (p=0.004) and macula D90 (p=0.002) were associated with visual loss <0.20. The relation between visual acuity, tumour localisation and dose parameters is presented in figure 1.

**Conclusion**: Progressive decrease in visual acuity after proton therapy for uveal melanoma starts within one year and results in loss of useful vision in 4 out of 5 patients, with proximity to the macula and tumor volume as the most important risk factors.

## P181 -

### Feasibility of pencil beam scanning proton therapy of ocular melanomas with a conventional gantry beam line

#### Sheng Huang^1^, Minglei Kang^1^, Qing Chen^1^, Christopher Barker^2^, Charles Simone^1^, Haibo Lin^1^

##### ^1^New York Proton Center, Medical Physics, New York, USA ^2^Memorial Sloan Kettering Cancer Center, Radiation Oncology, New York, USA

**Purpose**: To evaluate the feasibility of proton therapy of ocular melanomas using a non-dedicated treatment planning system (TPS) and proton pencil beam scanning gantry beam line.

**Methods**: The commercial Eclipse TPS was used to generate a robust multifield optimized (rMFO) intensity-modulated proton plan for representative ocular tumor patients. Doses were compared among the initial plan and 40 additional scenarios of combined setup errors and range uncertainties. An in-house fast Monte Carlo dose calculation platform was used to assess the dosimetric impact of 3 tantalum fiducial markers for imaging-guidance treatment.

**Results**: rMFO planning accounting for 2mm setup uncertainty and 3.5% range uncertainty was performed, utilizing 3 fields at different optimal gantry angles. All plans achieved satisfactory target coverage (TC), with at least 95% of CTV receiving the full prescription of 50 Gy RBE in 5 fractions while achieving clinical dose limits of all organ at risks. The average target coverage remained D95=97.7% over 40 scenarios. Monte Carlo dose calculation revealed up to an 11% local dose shadow within target and D95 decreased by 3.2% if the tantalum marker is in the field of the beam path (Figure).

**Conclusion**: Non-dedicated TPS and gantry beam line can be used to effectively treat ocular tumors. This procedure is feasible with relatively low doses to anterior structures and achieves acceptable plan robustness. Fiducial markers, in certain circumstances, cause dose shadows and could theoretically compromise local tumor control. Optimized beam angle and fiducial positioning should be considered.

## P182 -

### Eye and vision sparing in proton therapy of orbital rhabdomyosarcoma of eyelids

#### Chankyu Kim^1^, Young Kyung Lim^1^, Yang-Gun Suh^1^, Joo-Young Kim^1^, Young Kyu Lee^1^, Myeongsoo Kim^1^, Tae Ho Kim^1^, Haksoo Kim^1^, Jong Hwi Jeong^1^, Dongho Shin^1^, Se Byeong Lee^1^

##### ^1^National Cancer Center Korea, Proton Therapy Center, Goyang-si, Korea Republic of

**Purpose:** This study reports proton therapy using customized eye shields in the treatment of orbital rhabdomyosarcoma (RMS) of eyelids, for eye and vision sparing of pediatric patients.

**Methods:** The customized eye shields were designed and prepared to protect lens of pediatric patients during proton therapy. The eye shields were made of special plastic to block the proton beam and prevent image artifacts in CT. To verify the physical effectiveness before applying to the treatment, dose distributions depending on the depth were measured using EBT3 films and an ion chamber. The proton therapy plans using the proposed eye shields were compared with the conventional electron therapy plans.

**Results:** CT images of the brain were obtained without image artifacts. The measured results showed that the eye shields blocked out proton beam efficiently in a protected area. In comparison with the electron therapy plans, the proton therapy plans using the proposed eye shields satisfied the clinical goals while delivering a lower dose to surrounding OARs.

**Conclusions:** In this study, the customized plastic eye shields for the treatment of orbital RMS with proton therapy were suggested. The physical effectiveness was verified and the clinical profits were discussed. The proposed eye shields can be used for protecting of lens in other treatments with proton therapy.

## P183 -

### Neuroendocrine toxicities in paediatric CNS proton therapy: An update on clinical evidence

#### Mikaela Dell'oro^1,2^, Michala Short^1^, Puthenparampil Wilson^3,4^, Hien Le^1,2^, Peter Gorayski^1,2^, Scott Penfold^3,5^, Eva Bezak^1,5^

##### ^1^University of South Australia, Cancer Research Institute, Adelaide, Australia ^2^Royal Adelaide Hospital- SA Health, Radiation Oncology, Adelaide, Australia ^3^Royal Adelaide Hospital- SA Health, Medical Physics, Adelaide, Australia ^4^University of South Australia, UniSA STEM, Adelaide, Australia ^5^University of Adelaide, Department of Physics, Adelaide, Australia

**Objectives**: Proton beam therapy (PBT) may be considered the gold standard of care for paediatric patients with CNS cancers due to its ability to maximally spare normal tissues. However, knowledge of radiobiological parameters affecting normal tissue complication probability (NTCP) for structures such as they hypothalamic-pituitary region in this vulnerable cohort is still limited. We systematically reviewed current literature to evaluate childhood CNS cancer clinical and patient reported outcomes treated with PBT.

**Methods**: A search strategy was conducted on MEDLINE® database in November 2020, limited to endocrine and neurological late side-effects.

**Results**: Twenty publications met the inclusion criteria. The effectiveness of PBT has been highlighted across the various publications; however, there are large variations in normal tissue tolerance and toxicity reporting for the hypothalamic-pituitary region (Table 1). This makes estimation of NTCP difficult, with the potential for significant late effects to go unmeasured. Endocrinopathy is correlated with mean hypothalamus dose ≥ 40 GyE, however dose delivery can be up to 54.6 GyE for paediatric patients. Similarly, there is a need to report neurological and cognitive effects in a standardised way.

**Conclusion**: There is a relative paucity of radiobiological data for paediatric CNS cancer patients undergoing PBT. A consensus of paediatric dose constraints for the treatment of brain tumours can help optimise individual patient plans to reduce NTCP. To mitigate this gap, registries have been established to collect data, which may prove the best way forward for standardisation of reporting and validation of NTCP estimates to inform clinical protocols and minimise side-effects.

## P184 -

### Proton therapy with a bio-absorbable spacer for Ewing sarcoma: the world's first pediatric case of space-making proton therapy

#### H. Iwata^1^, M. Kamei^2^, D. Takagi^2^, M. Naoko^3^, Y. Ito^4^, K. Mitsuhiro^5^, O. Hiroyuki^1^, S. Yuta^6^, T. Fukumoto^7^, S. Ryohei^7^

##### ^1^Nagoya Proton Therapy Center- Nagoya City West Medical Center, Radiation Oncology, Nagoya, Japan ^2^Nagoya City University Graduate School of Medical Sciences, Neonatology and Pediatrics, Nagoya, Japan ^3^National Hospital Organization Nagoya Medical Center, Pediatrics, Nagoya, Japan ^4^Nagoya City West Medical Center, Pediatrics, Nagoya, Japan ^5^Nagoya Proton Therapy Center- Nagoya City West Medical Center, Proton Therapy Physics, Nagoya, Japan ^6^Nagoya City University Graduate School of Medical Sciences, Radiology, Nagoya, Japan ^7^Kobe University Graduate School of Medicine, Hepato-Biliary-Pancreatic Surgery, Kobe, Japan

**Purpose/Objectives**: Proton therapy helps to reduce the exposure dose to the surrounding normal organs in comparison with X-ray therapy. However, radical radiation-dose administration is impossible in some patients in whom a bulky tumor is adjacent to the digestive tract, and exposure of the normal organs cannot be avoided. This is the first report of proton therapy in which bio-absorbable spacer (Neskeep®) were inserted for pediatric cancer.

**Methods**: The child was an 11-year-old boy with Ewing's sarcoma of the sacrum, protruding into the retroperitoneal cavity and adjacent to the rectum. After 5 courses of VAIA therapy, two 5-mm-thick Neskeep® were inserted into the retroperitoneal cavity between the tumor and digestive tract. A dose of 55.8 GyE/31Fr for scanning proton therapy was planned. Another virtual treatment plan was made on CT before insertion, and the dose distribution was compared. Furthermore, the absorbance of Neskeep® was evaluated.

**Results**: There were no serious adverse events, and the boy was discharged 4 days after insertion. Regarding comparison of dose distribution, the exposure doses for the surrounding normal organs were markedly reduced when the tumor target-covering rate was maintained. There has been no infection or ileus related to the insertion of Neskeep®. The absorbance of Neskeep® within 8 and 22 weeks after insertion was about 10% and ≥95%, respectively.

**Conclusion**: Neskeep® was safely placed for a child, which further improved the proton dose distribution. It may be an option to avoid both radiation-induced and surgery-related complications in the absence of spacer removal and to continue multidisciplinary treatment without interrupting anticancer drugs.

## P185 -

### Pencil-beam proton radiotherapy for patients with Atypical Teratoid Rhabdoid Tumor (ATRT) of the central nervous system: single center practice.

#### Natalia Martynova^1^, Nikolay Vorobyov^1^, Konstantin Boiko^2^, George Andreev^3^, Denis Antipin^1^, Roman Frolov^1^, Julia Gutsalo^1^, Andrey Shapovalov^2^, Ruzilya Girfanova^2^, Andrey Lubinskiy^3^, Ekaterina Smirnova^3^, Ekaterina Spiridenko^3^

##### ^1^Dr. Berezin Medical Institute MIBS, Proton Therapy Department, Saint-Petersburg, Russian Federation ^2^Dr. Berezin Medical Institute MIBS, Pediatric Oncology Department, Saint-Petersburg, Russian Federation ^3^Dr. Berezin Medical Institute MIBS, Physics Department, Saint-Petersburg, Russian Federation

**Introduction:** Atypical teratoid rhabdoid tumor of the central nervous system is a rare tumor affecting very young children. Treatment of ATRT includes different regimens of radiotherapy depending on age, stage, treatment protocols. Proton therapy allows to reduce acute and late toxicity. This reduction (among other benefits) helps to finish irradiation without interruptions. It matters for children receiving craniospinal irradiation as well as for children receiving radiotherapy after high dose chemotherapy.

**Materials and methods:** We reviewed data of 30 patients with ATRT treated with pencil-beam proton therapy from 06.2018 to 12.2020. We analyzed treatment characteristics, outcomes and other factors that could be prognostic for progression free survival.

**Results:** Median age at the start of radiotherapy was 28 months. Patients received local radiotherapy (18 patients) and craniospinal irradiation (12 patients). Median craniospinal dose was 29.8 Gy (24-36 Gy). Four patients got re-irradiation course because of local or leptomeningeal progression. One of them received craniospinal re-irradiation. Two of the patients did not finish course of radiotherapy because of distant progression. Median follow-up was 10 months. No interruptions were needed for any of the patients. Inability to achieve gross tumor resection (p=0.01) and M3-stage (p=0.03) were the most unfavorable prognostic factors for progression-free survival.

**Conclusion:** This is one of the largest reports of using pencil-beam proton therapy for children with ATRT of CNS treated in one facility. Proton therapy was well tolerated even by patients receiving re-irradiation course. Further research should be conducted in order to get information about late toxicity and survival outcomes.

## P186 -

### Commission and clinical implementation of Audiovisual-Assisted Therapeutic Ambience in pencil beam scanning proton radiation therapy

#### Zhiyan Xiao^1^, Dale James^1^, Katlin kuhn^1^, Lawrie Skinner^2^, Yongbin Zhang^1^, Eunsin Lee^1^, Anthony Mascia^1^, Ralph Vatner^1^, John Breneman^1^

##### ^1^Cincinnati Children's Hospital/University of Cincinnati Medical Center, Proton Therapy Center, Cincinnati, USA ^2^University of Stanford, Radiation Oncology, Stanford, USA

**Purpose**: Audiovisual-assisted therapeutic ambience in radiation therapy (AVATAR) has been applied in photon RT with the goal to reduce the need for daily anesthesia through immersion in video. The purpose of this work is commission and clinical implementation of AVATAR in Pencil Beam Scanning Proton Radiation Therapy.

**Methods**: Start from CT simulation, AVATAR system is included in CT images (Fig.1A) for body contouring. Treatment planning study develops to verify the dosimetry distribution disturbed by AVATAR. The guideline with the considerations on beam angles, nozzle clearance and airgap setting were proposed in use of AVATAR.

**Results**: The WED (water equivalent distance) of screen, wire connect to screen and holder are 0.2 mm, 2 mm and 6 mm, respectively. The proton beam passing the screen does not has noticeable dosimetry impact on dose distribution. For comfortable viewing, the screen needs to be placed at least 10-15cm away from patient eyes. No any clearance concern unless using anterior beams. For the treatment without range shifter, the nozzle fully retract, no clearance concern with any beam angles (Fig1B). In case of range shifter employed for tumor located at shallow anterior side (Fig.1C, D), the air gap would be at least 18 cm for coplanar anterior beam, depending on where ISO center is. The increasing spot size with increased air gap might cause a larger penumbra on the surrounding tissues. The comparison plans with/without AVATAR is supposed to generate for doctor review to make decision.

**Conclusion**: The clinical guideline was established for AVATAR implementation in PBS treatment.

## P187 -

### Impact of breast size on dosimetric indices in proton versus X-ray radiotherapy for breast cancer.

#### Lisa Cunningham^1,2^, Scott Penfold^1^, Eileen Giles^2^, Hien Le^1,2^, Michala Short^2^

##### ^1^Royal Adelaide Hospital, Radiation Oncology, Adelaide, Australia ^2^University of South Australia, UniSA Cancer Research Institute, Adelaide, Australia

Deep inspiration breath hold (DIBH) radiotherapy is a technique used to manage early-stage left-sided breast cancer. This study compared dosimetric indices of patient-specific X-ray versus proton therapy DIBH plans to explore differences in target coverage, radiation doses to organs at risk and the impact of breast size. Radiotherapy plans of sixteen breast cancer patients previously treated with DIBH radiotherapy were re-planned with hybrid inverse-planned intensity modulated X-ray radiotherapy (h-IMRT) and intensity modulated proton therapy (IMPT). The total prescribed dose was 40.05 Gy in 15 fractions for all cases. Comparisons between the clinical, h-IMRT and IMPT evaluated doses to target volumes, organs at risk and correlations between doses and breast size. Although no differences were observed in target volume coverage between techniques, the h-IMRT and IMPT were able to produce more even dose distributions and IMPT delivered significantly less dose to all organs at risk than both X-ray techniques (Table 1). A moderate negative correlation was observed between breast size and dose to the target in X-ray techniques, but not IMPT (Figure 1). Both h-IMRT and IMPT produced plans with more homogeneous dose distribution than forward-planned IMRT and IMPT achieved significantly lower doses to organs at risk compared to X-ray techniques.

## P188 -

### Cardiac dose sparing comparison of radiation techniques for comprehensive regional nodal radiation therapy for left-sided breast cancer: A planning study

#### Heejoo Ko^1^, Jee Suk Chang^2^, Jin Young Moon^2^, Won Hee Lee^2^, Chirag Shah^3^, Jin Sup Andy Shim^4^, Min Cheol Han^2^, Jong Geol Baek^2^, Ryeong Hwang Park^2^, Yong Bae Kim^2^, Jin Sung Kim^2^

##### ^1^The Catholic University of Korea, College of Medicine, Seoul, Korea Republic of ^2^Yonsei Cancer Center- Yonsei University College of Medicine, Department of Radiation Oncology, Seoul, Korea Republic of ^3^Taussig Cancer Institute- Cleveland Clinic, Department of Radiation Oncology, Cleveland- Ohio, USA ^4^New York Proton Center, New York Proton Center, New York, USA

**Purpose:** How modern cardiac sparing techniques and beam delivery systems using advanced x-ray and proton beam therapy (PBT) can reduce incidental radiation exposure doses to cardiac and pulmonary organs individually or in any combination is poorly investigated.

**Methods:** Among 15 patients with left-sided breast cancer, partial wide tangential 3D-conformal radiotherapy (3DCRT) delivered in conventional fractionation (CF) or hypofractionated (HF) schedules; PBT delivered in a CF schedule; and volumetric modulated arc therapy (VMAT) delivered in an HF schedule, each under continuous positive airway pressure (CPAP) and free-breathing (FB) conditions, were examined. Target volume coverage and doses to organs-at-risk (OARs) were calculated for each technique. Outcomes were compared with one-way analysis of variance and the Bonferroni test.

**Results:** Target volume coverage was within acceptable levels in all interventions, except for the internal mammary lymph node D95 in 3DCRT. The mean heart dose (MHD) was the lowest in PBT (<1 Gy) and VMAT-CPAP (2.2 Gy) and the highest in 3DCRT-CF-FB. The mean lung dose (MLD) was the highest in 3DCRT-CF-FB and the lowest in both VMAT-HF-CPAP and PBT.VMAT-HF-CPAP and PBT delivered a comparable maximum dose to the left ascending artery.

**Conclusions:** Modern PBT using scanning technique can achieve the lowest doses to most OARs in the regional nodal treatment for left-sided breast cancer. However, both proton and VMAT in combination with CPAP can minimize the radiation exposure to heart and lung with optimal target coverage. Continued efforts are needed to minimize radiation exposures during RT treatment to maximize its therapeutic index.

## P189 -

### Dosimetric comparison of intensity-modulated proton radiotherapy versus intensity-modulated photon-based radiotherapy for breast cancer

#### R. Lin^1^, J. Shan^2^, T. Yuan^1^, C. Qian^1^

##### ^1^Guangzhou Concord Cancer Center, Radiation Oncology, Guangzhou, China ^2^Mayo Clinic Arizona, Radiation Oncology, Scottsdale, USA

**Purpose:** This study aims to compare the dosimetric differences in intensity-modulated proton therapy (IMPT) using pencil beam scanning technology and intensity-modulated photon-based radiotherapy (IMRT) in hypofractionated whole breast irradiation (HF-WBI).

**Methods and Materials:**8 breast cancer (BC) patients with pathological stage T1∼2N0M0 were immobilized and underwent 4D-CT scanning used deep inspiration breath hold (DIBH) technology. IMPT plan and IMRT plan were both designed for each patient. Prescription dose and regimen was 40.05 Gy (relative biological effect [RBE])/15fx with a 10 Gy (RBE)/5fx boost. Dose of 95% of the target volume should not less than prescription dose. D1, D2, D50, D95, D98, D99, Conformity index (CI) and homogeneity index (HI) were used to evaluate the target coverage. Organs at risk (OARs) were evaluated using Dmean and Dmax. Ipsilateral Lung and Contralateral Lung were evaluated additionally using V5, V10, V20, V30.

**Results:** For PTV, there was no significant difference in target coverage between IMRT and IMPT plans. But for the OARs, the mean dose (Dmean) to Heart (*P* = 0.012), Ipsilateral Lung (*P* = 0.036), Contralateral Lung (*P* = 0.012) and Spinal Cord (*P* = 0.012) were significantly reduced in IMPT plans. IMPT also showed a tendency to reduce the V20 (*P* = 0.05) and V30 (*P* = 0.05) of Ipsilateral Lung.

**Conclusion:** Compared to IMRT, IMPT using pencil beam scanning technology can spare OARs without compromising target coverage in BC patients undergoing HF-WBI, which potentially reduce the incidence of radiation-related adverse effects.

## P190 -

### Cardiac node exposure with IMPT versus VMAT for left breast cancers

#### Pierre Loap^1^, Vincent Servois^2^, Gilles Dhonneur^3^, Krassen Kirov^3^, Alain Fourquet^1^, Youlia Kirova^1^

##### ^1^Institut Curie, Department of Radiation Oncology, Paris, France ^2^Institut Curie, Department of Radiology, Paris, France ^3^Institut Curie, Department of Anesthesiology, Paris, France

**Purpose and objective**: Conduction system exposure could result in rhythmic disorders. Breast protontherapy reduces cardiac dose but impact on conduction system has never been evaluated. This study compares doses to atrioventricular (AVN) and sinoatrial nodes (SAN) with VMAT and intensity-modulated protontherapy.

**Material and Methods**: SAN and AVN delineation guidelines were proposed by a multidisciplinary staff based on radio-anatomy and histology articles**.** Seven left-sided breast cancer patients treated with tumorectomy and VMAT were included. Patients received 51.8Gy to the whole breast, 50.4Gy to axillary and supraclavicular nodes and 63Gy to the tumor bed. IMPT plans were generated with similar PTV constraints. The endpoint was the mean and maximum doses to the SAN and AVN.

**Result**: The SAN was delineated by a 2 cm-diameter sphere, centered on the junction between the superior vena cava and the right atrium, tangent to the external wall of the right atrium. The AVN was delineated by a 2 cm-diameter sphere centered on the junction of the cardiac cavities, 1 cm above the last slice where the left atrium is visible (Figure 1.A-B). IMPT reduces mean AVN dose from 1.636 Gy to 0 Gy, mean SAN dose from 2.579 Gy to 0.006 Gy, maximum AVN dose from 2.703 Gy to 0 Gy and maximum SAN dose from 4.272 Gy to 0.059 Gy (p<0.01) (Figure 1.C-D).

**Conclusion**: Protontherapy delivers virtually no dose to conduction nodes and should suppress radiation-induced conduction disorder risk.

## P191 -

### Impact of baseline serum cholinesterase in elderly patients with treatment naive hepatocellular carcinoma treated with proton beam therapy

#### Takashi Iizumi^1^, Toshiyuki Okumura^1,2^, Toshiki Ishida^2,3^, Yuichi Hiroshima^1^, Masatoshi Nakamura^1^, Shosei Shimizu^1^, Yuta Sekino^1^, Takashi Saito^1^, Keiichi Tanaka^4^, Reiko Kanuma^4^, Daichi Takiizawa^5^, Haruko Numajiri^1^, Masashi Mizumoto^1,2^, Kei Nakai^1,2^, Hideyuki Sakurai^1,2^

##### ^1^Tsukuba University Hospital, Radiation Oncology, Tsukuba, Japan ^2^University of Tsukuba, Radiation Oncology, Tsukuba, Japan ^3^Ibaraki Prefectural Central Hospital, Raditaion Oncology, Tsukuba, Japan ^4^Tsuchiura Kyodo General Hospital, Radiation Oncology, Tsuchiura, Japan ^5^Hitachi General Hospital, Radiation Oncology, HItachi, Japan

**Purpose**: The purpose of this study is to assess the baseline serum cholinesterase level (ChE) as a predictor of survival in a retrospective cohort of elderly patients with treatment naive hepatocellular carcinoma (HCC) treated with proton beam therapy (PBT).

**Methods**: A total of 94 patients over 75 years old with treatment-naive HCC who underwent PBT between November 2001 and November 2014 were studied. Overall survival (OS) was evaluated according to Kaplan-Meier method and compared by the log-rank test. Univariate and multivariate Cox proportional hazards regression model was used to explore prognostic factors predictive of OS. To create dichotomous variables, ROC curve analysis was applied to continuous variables.

**Results**: On univariate analysis, baseline ChE (hazard ratio [HR], 0.50; 95% confidence interval [CI] 0.30-0.82; P < 0.01), serum albumin level (HR, 0.47; 95% CI 0.28-0.79; P < 0.01), and des-gamma-carboxy prothrombin level (DCP) (HR, 2.02; 95% CI 1.04-3.89; P = 0.04) were statistically significant predictors. On multivariate analysis, baseline ChE (HR, 0.54; 95% CI 0.30-0.98; P = 0.04), and DCP (HR, 2.19; 95% CI 1.12-4.26; P = 0.02) were statistically significant predictors. Similarly, patients with a baseline ChE of ≥ 193 IU/l showed significant longer survival than those with a baseline ChE of < 193 IU/l with a median OS of 32.7 months and 57.0 months (P < 0.01).

**Conclusion**: Lower ChE was a significant predictor of poor prognosis for elderly patients with treatment naive HCC treated with PBT. Baseline ChE cutoff of 193 IU/l may be useful for the prognostic predictor for these patients.

## P192 -

### Carbon-ion radiotherapy for primary or postoperative recurrent pancreatic cancer: the initial experience of i-ROCK (ion-beam Radiation Oncology Center in Kanagawa).

#### Nobutaka Mizoguchi^1^, Kio Kano^1^, Wataru Anno^1^, Keisuke Tsuchida^1^, Yosuke Takakusagi^1^, Itsuko Serizawa^1^, Daisaku Yoshida^1^, Tadashi Kamada^1^, Hiroyuki Katoh^1^

##### ^1^Kanagawa Cancer Center, Department of Radiation Oncology, Yokohama, Japan

**Objective**: To evaluate the efficacy and safety of carbon-ion radiotherapy (CIRT) for primary or postoperative recurrent pancreatic cancer at i-ROCK.

**Methods and Materials**: Patients with primary or postoperative recurrent pancreatic cancer treated with CIRT from June 2018 to Sep 2019 were retrospectively analyzed. Patients with pathologically or clinical confirmed invasive ductal adenocarcinoma of the pancreas were eligible. The prescribed dose was 55.2 Gy (RBE) in 12 fractions. Overall survival (OS), local recurrence (LR), distant metastasis-free survival (DMFS), progression-free survival (PFS) and toxicity were evaluated.

**Results**: Twenty-six patients were included. Median age was 76 years (range, 36-87). Stage distribution was Stage I, 7 (27%); II, 5 (19%); and III, 14 (54%). Two patients were postoperative recurrent pancreatic cancer. Median follow-up was 18.4 months (range, 9.2-28.9). 21 patients (80%) received concurrent chemotherapy. 1-year rates of OS, LC, DMFS and PFS were 88.7%, 92.3%, 57.4% and 38.5%, respectively. One patient had early grade 3 biliary tract infection. One patient experienced late grade 3 bile duct stenosis. Late grade 3 biliary tract infection was observed in one patient. No patients developed late grade 4 or 5 toxicity.

**Conclusions**: This analysis demonstrated that CIRT at i-ROCK is a feasible treatment for primary or postoperative recurrent pancreatic cancer.

## P193 -

### Escalated-dose proton therapy with elective nodal irradiation and concomitant chemotherapy for unresectable, borderline resectable, or medically inoperable pancreatic adenocarcinoma

#### Cooper Rapp^1^, Michael S. Rutenberg^2^, Christopher G. Morris^1^, R. Charles Nichols- Jr.^2^

##### ^1^University of Florida College of Medicine, Radiation Oncology, Gainesville, USA ^2^University of Florida College of Medicine, Radiation Oncology, Jacksonville, USA

**Purpose**: Report outcomes of a phase II single-institution trial of escalated-dose proton therapy with elective nodal irradiation and concomitant chemotherapy for patients with unresectable, borderline resectable, or medically inoperable pancreatic adenocarcinoma.

**Methods**: Patients were treated to 40.5 GyE in 18 fractions to gross disease and elective nodal volumes with a 22.5 GyE 10-fraction boost to gross disease. Oral capecitabine (1000mg BID) was given on radiation treatment days. Primary objective was to achieve a 1-year overall survival rate of 75%. Secondary objectives included assessing gastrointestinal toxicity and quality of life, and evaluating the safety of subsequent resection.

**Results**: At enrollment, 10 (67%) patients were unresectable, 3 (20%) were borderline, and 2 (13%) refused surgery. Toxicity consisted of a single patient who experienced grade 3 nausea requiring cessation of capecitabine. All other patients completed radiotherapy and chemotherapy as prescribed. Median percentage weight loss during treatment was -3.0% (-9.6% - +12.0%). Two initially borderline patients ultimately underwent resection. Neither patient experienced intraoperative or postoperative complications. Median follow-up was 0.93 years (0.21-2.14). The 1-year overall survival rate was 47%. Three enrolled patients are currently alive: 2 with no evidence of disease and 1 with stable disease. One patient died of intercurrent disease. Of the remaining 11 patients who died following tumor recurrence, 3 failed locally, with 8 failing distantly.

**Conclusion**: Protocol therapy was well-tolerated. Patients undergoing surgery did not experience operative or perioperative complications – suggesting that patients with borderline resectable or even resectable disease may benefit from this regimen as neoadjuvant therapy.

## P194 -

### Dosimetric study of proton therapy using scanning method for localized prostate cancer patients in comparison with wobbler method and VMAT

#### M. Takagi^1^, Y. Hasegawa^1^, K. Tateoka^1^, Y. Takada^1^, M. Hareyama^1^

##### ^1^Sapporo Teishinkai Hospital, Proton Therapy Center, Sapporo, Japan

**Introduction**: In recent years, proton therapy (PT) for localized prostate cancer has been gradually shifting to scanning methods from wobbler methods. We performed a planning study to compare the dose distribution of the scanning method with that of the wobbler and the volumetric modulated arc therapy (VMAT) for localized prostate cancer patients.

**Materials/Methods**: Thirty prostate cancer patients were randomly selected. Three radiotherapy plans were created for each patient with the same prescribed dose (76.0 Gy or Gy [RBE] in 38 fractions) and compared using dose-volume histograms. Dose constraints were clinical target volume (CTV) D98 ≥ 73.0 Gy (RBE), rectal wall V65 < 17%, V40 < 35%, and bladder wall V65 < 25% and V40 < 50%, respectively. CTV doses, bladder and rectum wall dose volumes (V10 - V75), Dmean, and Dmax were calculated and tested by Wilcoxon's rank-sum test. P < 0.05 was determined to be statistically significant.

**Results**: The dose coverage of CTV was favorable with the scanning and the wobbler than with the VMAT. Wide ranges of the rectal and bladder wall volumes of V10 - V70 were significantly lower with the scanning method compared with the other two methods (P < 0.05). The wobbler method yielded better dose distribution of the rectum and bladder wall in the low dose range (V10-20) and near the maximum dose compared with the VMAT.

**Conclusion**: Compared with the wobbler method and the VMAT, the scanning method enables further reduction of the rectal and bladder doses while maintaining the CTV dose.

## P195 -

### Changes of obturator internus muscle after Helical Tomotherapy for prostate cancer and efficacies of proton therapy using a scanning-dedicated machine.

#### T. Takaoka^1^, T. Yanagi^1^, M. Tomida^1^, Y. Shibamoto^2^

##### ^1^Narita Memorial Proton Center, Radiation Oncology, Toyohashi, Japan ^2^Nagoya City University Graduate School of Medical Sciences, Radiology, Nagoya, Japan

**Introduction**: Narita Memorial Proton Center (Toyohashi, Aichi, Japan) started proton therapy in September 2018 for patients with malignant tumors using a compact proton beam machine dedicated to pencil beam scanning. (Proteus® One, Ion Beam Applications S.A., Belgium). Obturator internus muscle (OIM) is one of important muscles related to external rotation and urinary incontinence. We report changes of OIM and efficacies of proton therapy using pencil beam scanning.

**Materials and methods**: Between January 2013 and December 2016, 28 patients with prostate cancer treated with Helical Tomotherapy (intensity-modulated radiation therapy) were analyzed retrospectively. Prescribed doses to cover 50% of the target were 72.6Gy in 33 fractions for low-risk and 74.8Gy in 34 fractions for intermediate- and high-risk. Computed tomography (CT) images were acquired before treatment and after 12 and 24 months. To assess the volume of OIM, the CT images were analyzed in RayStation systems. Proton therapy plans were generated using CT images of patients treated with Helical Tomotherapy.

**Results**: The ratios of posttreatment (12 and 24 months) OIM compared with pretreatment OIM were median 0.82 (range, 0.78-1.03) and median 0.81 (range, 0.76-1.02). The median of V_40Gy_,V_50Gy_,V_60Gy_,V_70Gy_, and mean dose to OIM were 82%, 48%, 32%, 17%, and 52Gy, respectively. In the proton therapy planning, the median of V_40GyE_,V_50GyE_,V_60GyE_,V_70GyE_, and mean dose to OIM were 45%, 33%,23%,9%, and 36GyE, respectively.

**Conclusion**: The OIM atrophy was observed in patients (22/28) after the prostate cancer treatment. Proton therapy using pencil beam scanning improves the intermediate- and high-exposed volume of OIM and achieve comparable target dose coverage.

## P196 -

### Evaluation of interfractional changes in each session number in carbon-ion radiotherapy for prostate cancer using daily in-room computed tomography

#### Keisuke Tsuchida^1^, Shinichi Minohara^2^, Yohsuke Kusano^2^, Kio Kano^1^, Wataru Anno^1^, Yosuke Takakusagi^1^, Nobutaka Mizoguchi^1^, Itsuko Serizawa^1^, Daisaku Yoshida^1^, Kamada Tadashi^1^, Hiroyuki Katoh^1^

##### ^1^Kanagawa Cancer Center, Radiation Oncology, Yokohama, Japan ^2^Kanagawa Cancer Center, Section of Medical Physics and Engineering, Yokohama, Japan

**Purpose**: To evaluate the dosimetric robustness of pencil-beam scanning carbon-ion radiotherapy (CIRT) for prostate cancer using in-room computed tomography (CT).

**Materials and Methods**: In-room CT is set in the treatment room in our facility. In the treatment, patients are set using orthogonal x-ray FPD images based on the bone structures, then just before or after the irradiation, in-room CT images are taken under the treatment conditions. We analyzed the in-room CT images obtained from prostate cancer patients who received CIRT between December 2015 and January 2016. The prescription dose was set at 51.6 Gy (RBE) in 12 fractions over 3 weeks. Dose constraints were CTV %V95 ≥ 95%, CTV %V90 ≥ 98% and rectum %V80 < 10 cc. We delineated the clinical target volume and rectum for in-room CT images, then we verified dose distributions.

**Results**: Eleven patients with 131 treatment sessions were identified. The median displacement of CTV center between in-room and planning CT was (AP, LR SI) = (-1.5, 0.0, 0.0) (mm). The median dose of CTV %V95 and %V90 were 100% (range 75.8 - 100%) and 100% (range 83.3 - 100%). The median difference of rectum %V80 between in-room and planning CT was -0.72 (range -4.53 - 16.27) cc. The number of sessions which satisfied the dose constraints of CTV %V95, CTV %V90 and rectum %V80 were 123 (93.9%), 124 (94.7%) and 117 (89.3%).

**Conclusion**: This study demonstrated that planning dose distributions were robust in the actual treatment sessions of pencil-beam scanning CIRT for prostate cancer.

## P197 -

### Exploratory investigation of dose-linear energy transfer (LET) volume histogram (DLVH) for adverse events study in intensity-modulated proton therapy (IMPT)

#### Yunze Yang^1^, Carlos Vargas^1^, Ronik Bhangoo^1^, William Wong^1^, Steven Schild^1^, Thomas Daniels^1^, Sameer Keole^1^, Jean-Claude Rwigema^1^, Jennifer Glass^1^, Jiajian Shen^1^, Todd DeWees^2^, Tianming Liu^3^, Martin Bues^1^, Mirek Fatyga^1^, Wei Liu^1^

##### ^1^Mayo Clinic Arizona, Department of Radiation Oncology, Phoenix, USA ^2^Mayo Clinic Arizona, Division of Biostatics, Phoenix, USA ^3^University of Georgia, Department of Computer Science, Athens, USA

**Purpose**: We proposed a novel tool of dose-linear-energy-transfer (LET) volume histogram (DLVH) and performed an exploratory study to investigate rectal bleeding in prostate cancer treated by intensity-modulated proton therapy (IMPT).

**Methods**: DLVH was constructed with dose and LET as two axes, while the normalized volume of the structure was contoured in the dose-LET plane as iso-volume lines (Fig.1a). We defined DLVH index, DL*v*_%_(*d*,*l*), *i.e*., *v*_%_ of the structure have a dose of ≥ *d* Gy and an LET of ≥*l* keV/μm similar to dose-volume histogram index D*v*_%_. Nine prostate cancer patients with rectal bleeding (CTCAE grade≥2) were included as the adverse event group, while 48 patients with no complication were considered as the control group (Fig.1b). *P*-value map was constructed by comparison of the DLVH indices of all patients between the two groups (Fig.2a). Dose-LET volume constraints (DLVCs) were derived based on the *p*-value map. The obtained DLVCs were further cross-validated and tested using a support-vector-machine (SVM)-based normal tissue complication probability (NTCP) model.

**Results**: We extracted two DLVCs. DLVC1:*V*(dose/LET:2.5keV/μm at 75Gy to 3.2keV/μm at 8.65Gy)<1.27%, revealed high LET volume effect. DLVC2:*V*(72.2Gy, 0keV/μm)<2.23%, revealed high dose volume effect. The SVM-based NTCP model with two DLVCs (Fig.2b) provided slightly superior performance than using dose only with an area-under-the-curve of 0.798 vs. 0.779 for the testing dataset (Fig.2c).

**Conclusions**: Our results demonstrated the importance of “hot spots” in both LET and dose in inducing rectal bleeding. The SVM-based NTCP model confirmed the derived DLVCs as good predictors of rectal bleeding for IMPT.

## P198 -

### Systematic review of deep inspiration breath hold in proton therapy and IMRT for mediastinal lymphoma

#### Chirayu Patel^1^, Jennifer Peterson^2^, Marianne Aznar^3^, Yolanda Tseng^4^, Scott Lester^2^, Deanna Pafundi^5^, Stella Flampouri^6^, Bradford Hoppe^5^

##### ^1^MGH, Radiation Oncology, Boston, USA ^2^Mayo Clinic, Radiation Oncology, Rochester, USA ^3^University of Manchester, Division of Cancer Sciences, Manchester, United Kingdom ^4^University of Washington, Radiation Oncology, Seattle, USA ^5^Mayo Clinic, Radiation Oncology, Jacksonville, USA ^6^Emory, Radiation Oncology, Atlanta, USA

**Purpose:** To systematically review all dosimetric studies investigating the impact of deep inspiration breath hold (DIBH) compared with free breathing (FB) in mediastinal lymphoma patients treated with proton therapy as compared to IMRT-DIBH.

**Materials and Methods:** A systematic search in PubMed was done to identify studies of mediastinal lymphoma patients with dosimetric comparisons of proton-FB and/or proton-DIBH with IMRT-DIBH including mean heart, lung, and breast doses (named MHD, MLD, and MBD respectively). Case reports were excluded. As of December 2020, eight studies fit these criteria.

**Results:** The trends in dose are summarized in the table. MHD was reduced (n=2), similar (<1 Gy difference, n=2), or worse (2.5 Gy worse, n=1) for proton-FB compared with IMRT-DIBH. MLD and MBD in all studies were reduced for proton-FB compared with IMRT-DIBH. Proton-DIBH led to lower MHD (2.3-7.4 Gy difference,) and MLD (0.7-1.1 Gy difference) compared to proton-FB, while MBD remained within 0.3 Gy in all studies. Compared with IMRT-DIBH, proton-DIBH reduced the MHD (1.5-10.1 Gy, n=7) or was similar (n=1). MLD (1.7-3.9 Gy) and MBD (1.5-7.8 Gy) were reduced with proton-DIBH in all studies. Integral dose was similar between proton-FB and proton-DIBH, and both were substantially lower than IMRT-DIBH.

**Conclusion:** Accounting for heart, lung, breast, and integral dose, proton therapy (FB or DIBH) was superior to IMRT-DIBH. Proton-DIBH can lower dose to the lungs and heart even further compared with proton-FB. However, the substantial increase in physics resources required at simulation and treatment must be considered to ensure accurate reproducibility for proton-DIBH treatment delivery.

## P199 -

### Outcomes and toxicities of curative reirradiation for head and neck carcinomas

#### Arnaud Beddok^1^, Caroline Saint-Martin^2^, Samar Khrili^1^, Anne Chilles^1^, Ludivine Catteau^1^, Farid Goudjil^3^, Sofia Zefkili^1^, Olivier Choussy^4^, Christophe Le Tourneau^5^, Rémi Dendale^6^, Irène Buvat^7^, Gilles Créhange^1^, Valentin Calugaru^1^

##### ^1^Institut Curie, Radiation Oncology, Paris, France ^2^Institut Curie, Statistics, Saint-Cloud, France ^3^Institut Curie, Proton Therapy Center, Paris, France ^4^Institut Curie, Head and Neck surgery, Paris, France ^5^Institut Curie, Medical Oncology, Paris, France ^6^Institut Curie, Proton Therapy Center, Orsay, France ^7^Institut Curie, Laboratoire D'imagerie Translationnelle Oncologique, Orsay, France

Despite the constant improvement of techniques, locoregional recurrences (LR) after radiation therapy (RT) for head and neck cancer (HNC) remain frequent. In a curative context, in case of inoperability or incomplete resection, reirradiation (reRT) can be discussed. The objective of this study was to retrospectively analyze the outcomes and toxicities of patients treated with curative reRT (either by intensity-modulated radiation therapy [IMRT], or by proton therapy [PT]) for recurrent HNC. Twenty-three patients were re-irradiated from 30/08/2012 to 08/04/2019 (47.8% with IMRT and 52.2% with PT) with curative intent for advanced HNC. The median time between the two irradiations was 23.9 months. The median maximum dose prescribed to the CTV was 66 Gy (EBR), the median PTV was 124.3 cc. After a median follow-up of 15 months from the end of reRT, a total number of 18 patients (78.2%) developed LR. The local control (LC) rate and survival without LR at 18 months were: 29.3 and 26.1%. Most patients (74%) did not have grade 2 toxicity before the start of reRT. Grade ≥ 2 osteoradionecrosis and temporal radionecrosis occurred in 13.4% and 8.7% of patients, respectively. Carotid blowout occurred in three patients (13.4%). Dysgeusia was significantly more frequent in the photon than in the proton group (p=0.017). Curative reRT in HNC is possible for selected cases, but the LR rate in the irradiated field and the risk of toxicity grade ≥ 2 remain high. Improved selection criteria and definition of target volumes may improve the outcome of these patients.

## P200 -

### Sparing of the tubarial salivary glands with proton radiation therapy for HPV-associated oropharynx cancer

#### Christopher Wright^1^, Daniel Y. Lee^1^, Michele Kim^1^, Andrew R. Barsky^1^, Boon-Keng Kevin Teo^1^, John N. Lukens^1^, Samuel Swisher-McClure^1^, Alexander Lin^1^

##### ^1^University of Pennsylvania, Department of Radiation Oncology, Philadelphia, USA

**Purpose:** The recently identified bilateral macroscopic tubarial salivary glands (TSGs) present an opportunity for toxicity mitigation for patients receiving head and neck radiotherapy (Valstar, et al. Radiotherapy and Oncology 2020). Here, we show superior dosimetric sparing of the TSGs with proton radiotherapy (PRT) compared to intensity-modulated radiotherapy (IMRT) for patients treated postoperatively for human papillomavirus (HPV)-associated oropharyngeal squamous cell carcinoma (OPSCC).

**Methods:** This was a retrospective, single-institution study of patients treated with adjuvant PRT for HPV-associated OPSCC from 2015-2019. Each patient had a treatment-approved, equivalent IMRT plan to serve as a reference for comparison. The primary endpoint was dose delivered to the TSGs by modality, assessed via a two-tailed, paired t-test. We also report disease control outcomes for the cohort, via the Kaplan-Meier method.

**Results:** Sixty-four patients were identified. The mean RT dose to the tubarial salivary glands was 23.6 Gy (95% confidence interval 21.7-25.5) and 30.4 Gy (28.6-32.2) for PRT and IMRT plans (p < 0.0001), respectively (Figure 1). With a median follow-up of 25.2 months, the two-year locoregional control, progression-free survival and overall survival were 97.8% (85.6-99.7%), 94.1% (82.8-98.1%) and 98.1% (87.4-99.7%), respectively.

**Conclusions:** Our study suggests that significant normal tissue sparing of the recently identified TSGs is achievable with PRT. The apparent gains with PRT did not impact disease control outcomes, with only 1 observed locoregional recurrence (0 local, 1 regional). Further studies are warranted to explore the impact of the improved dosimetric sparing of the TSGs conveyed by PRT on patient toxicity and quality of life.

## P201 -

### Synchrotron 360° gantry clinical performance in the initial 100 patients: experience at Clinica Universidad de Navarra

#### Felipe Calvo Manuel^1^, Juan Diego Azcona Armendáriz^2^, Santiago Martín^1^, Borja Aguilar^2^, Javier Serrano^1^, Mauricio Cambeiro^1^, Alejandro García-Consuegra^1^, Javier Aristu^1^

##### ^1^Clínica Universidad de Navarra, Radiation Oncology, Madrid, Spain ^2^Clínica Universidad de Navarra, Radiation Physics, Madrid, Spain

**Background**: Proton beam therapy (PBT) is an efficient high-energy particle radiation technology accessible in modern clinical practice.

**Methods**: Between March 2020 and January 2021 a total of 100 patients, with a median age of 48 (range 2-86) were treated with synchrotron 360° gantry at Clinica Universidad de Navarra Cancer Center in Madrid (Spain). Initial clinical observations are described.

**Results**: Anatomic regions treated were: intracraneal (n=28), skull base (n=14), pelvis (n=20), para-spinal (n=5), head and neck (n=14), thorax (n=5), upper abdomen (n=5), craniospinal (n=9). Adenocarcinoma (n=20) and chordoma/chondrosarcoma (n=15) were frequent histological subtypes. 29 patients had re-irradiation indications. 8 developed positive test for SARS-COV2 . 60 patients received >50Gy RBE. 4 patients were treated with extreme hypofractionation schemes (37 moderate, 59 with standard fractionation). Median number of fractionation was 25 (5-37). Median number of beam incidences were 3 (1-5). Acute toxicity (RTOG grade = >3) was observed in 5 patients. Median follow up time is 4 months. Restaged patients shown a 62% objective remission (complete 35%).

**Conclusion**: Synchrotron technology with 360° has proven versatile performance in contemporary open clinical practice.

## P202 -

### Early outcomes of patients with sacral chordoma treated with high-dose proton beam therapy.

#### Tomasz Skóra^1^, Dominika Wojton-Dziewońska^1^, Elżbieta Pluta^1^, Anna Patla^1^, Mariusz Wszołek^1^, Jadwiga Nowak-Sadzikowska^1^, Konrad Urbanek^1^, Kamil Kisielewicz^2^, Eleonora Góra^2^, Renata Kopeć^3^, Damian Kabat^2^

##### ^1^Maria Sklodowska-Curie National Research Institute of Oncology, Department of Radiotherapy, Kraków, Poland ^2^Maria Sklodowska-Curie National Research Institute of Oncology, Department of Medical Physics, Kraków, Poland ^3^Institute of Nuclear Physcics Polish Academy of Sciences, Bronowice Cyclotron Center-, Kraków, Poland

**Purpose**: The aim of the study is to evaluate the short-term outcomes in terms of tumor control and toxicity of patients with sacral chordoma treated with spot-scanning beam proton radiotherapy.

**Methods and Materials**: From November 2016 through December 2019, 16 patients with sacral chordoma were treated with proton radiation therapy at our institution. Median patient age was 64.5 years (range, 42-81 years). The male to female ratio was reported as 1:1. The median radiation therapy dose for chordomas was 74.0 GyRBE. Nine patients (56.3%) underwent surgery before radiotherapy. In 2 cases (12.5%) surgical stabilization with titanium hardware was performed. Disease-free survival and overall survival were evaluated. The early and the late treatment toxicity was evaluated according to CTCAE (version 5.0).

**Results:** With a median follow-up period of 21.9 months (range, 4.7-46.9 months), the estimated 3-year disease-free survival and overall survival rates were 70.3% and 100.0%, respectively. Four patients (25.0%) experienced disease recurrence: local failures (n=3) and distant metastases (n=1). The mean time to disease reccurence was 29.5 months (range, 2.3-44.4 months). Acute grade ≥3 toxicities were observed in 1 patient. Late toxicities higher than G2 were not noticed so far.

**Conclusion**: For sacral chordomas high dose proton therapy remains a highly effective therapeutic method with acceptable toxicity. Nevertheless, a longer follow-up period is needed to confirm these results.

## P203 -

### Clues to address barriers for access to proton therapy in the Netherlands

#### Salina Thijssen^1^, Maria J.G. Jacobs^2^, Luca Heising^2^, Rachelle R. Swart^1^, Carol X. Ou^2^, Cheryl Roumen^1^, Liesbeth J. Boersma^1^

##### ^1^Department of Radiation Oncology Maastro- GROW School for Oncology, Maastricht University Medical Centre+, Maastricht, Netherlands ^2^Tilburg School of Economics and Management Tilburg, Tilburg University, Tilburg, Netherlands

**Purpose:** Proton therapy (PT) is a form of radiotherapy (RT) which allows more accurate dose-delivery than the routinely used photon therapy. Despite the significant advantages in reduced toxicity, only about 0.5% of patients from a non-connected Proton Therapy Center (PTC) is referred to PT, compared to approximately 5% in PTC-connected centers. The objective of this research is to identify the barriers impacting PT referrals in the Netherlands.

**Methods:** All 330 referring members of the Dutch Society for Radiation Oncology (NVRO) were asked to complete a survey, in the period of the 15th of June and 19th of August 2020. The survey was developed based on the Consolidated Framework for Implementation Research (CFIR). A pilot test was conducted with 5 independent referrers to measure the survey's feasibility.

**Results:** In total, 42% of the referring members completed the survey. Of the respondents, 70% work at an academic hospital, and 55% at a center without PTC. The most prominent referral barriers mentioned are the patient's choice (n = 65) (specifically travel time), logistics (n = 45) (specifically lead time, workflow and administrative burden), and perceived insufficient relative advantage (n = 38). Furthermore, knowledge (p < 0.001) and attitudes (p = 0.047) significantly influence referral to PT.

**Conclusion:** Key barriers for access to PT are travel distance, logistics, and lack of patient's- and referrer's awareness. The clues to address those barriers can be lifted by increasing awareness and education amongst referrers and patients, by reducing logistical barriers, and providing more evidence on benefits.

## P204 -

### New radiobiology beam line at the 18 MeV proton cyclotron facility at CNA (Seville, Spain)

#### Anna Baratto Roldán^1^, María del Carmen Jiménez Ramos^1^, María Isabel Gallardo^2^, Miguel Antonio Cortés Giraldo^2^, José Manuel Espino^1,2^

##### ^1^Centro Nacional de Aceleradores, Centro Nacional de Aceleradores, Sevilla, Spain ^2^Universidad de Sevilla, Departamento de Física Átomica- Molecular y Nuclear, Sevilla, Spain

In current proton therapy clinical practice, a constant Relative Biological Effectiveness (RBE) value of 1.1 is used, despite increasing evidences that proton RBE depends on many variables and increases with Linear Energy Transfer (LET) towards the distal region of the Bragg peak, possibly leading to toxicity in healthy tissue beyond the target. In this context, radiobiological investigations with non-clinical proton accelerators have gained interest in the last decades, since they can help collect accurate biological data at proton energies typically found at the Bragg peak region of clinical beams (below 40 MeV). Low energy proton accelerators, like the 18 MeV proton cyclotron installed at the National Centre of Accelerators (CNA, Seville, Spain), provide the perfect tool for this kind of studies, producing high LET beams with narrow energy distributions. With this purpose, we designed and mounted a radiobiology beam line at the CNA cyclotron, for the experimental study of RBE in mono-layer cell cultures. We established a protocol for the irradiation of biological samples consisting in: (1) an accurate alignment of the target position and a verification of the beam profile homogeneity with radiochromic EBT3 films, (2) a precise characterization of the beam parameters and dosimetry through a Geant4 Monte Carlo simulation of the beam line and (3) the control of environmental parameters, such as temperature and humidity, to avoid extra stress to the cells. With this protocol and our setup, we performed a successful irradiation of mono-layer cell cultures, proving the validity of the beam line for radiobiology experiments.

## P205 -

### PARP inhibition combined with protons enhance cell kill and might potentiate anti-tumor immune signaling

#### Mariam Ben Kacem^1^, Scott J. Bright^1^, Broderick X. Turner^1^, David B. Flint^1^, Mandira Manandhar^1^, Conor H. McFadden^1^, Simona F. Shaitelman^1^, Gabriel O. Sawakuchi^1^

##### ^1^MD Anderson Cancer Center, Department of Radiation Physics, Houston, USA

**Purpose:** PARP plays a role in both base excision repair and alternative non-homologous end-joining, which repair base damages (BD) and double strand breaks (DSBs), respectively. Clustered BD may in turn be converted into DSBs, which when not properly repaired can generate micronuclei, precursors of immune activation. We hypothesize that protons, with their more complex DNA damage yield than photons, sensitize cancer cells to radiation more than photons when combined with a PARP inhibitor (PARPi), and that this combination might also potentiate anti-tumor immune signaling.

**Methods:** To quantify the effect of a PARPi on radiosensitization and anti-tumor immune signaling, we obtained clonogenic cell survival data for H460, H1299, PANC-1 and Panc 10.05 cell lines and quantified cGAS-positive micronuclei (MN-cGAS^+^) for H1299, PANC-1, HCC1937 (BRCA mut) and HCC1937 (BRCA complemented) cell lines. Cells were treated with 6 MV x-rays or 9.9 keV/mm protons (dose-weighted LET in water) alone or with a PARPi (0.1 or 1 mM, Olaparib).

**Results:** PARPi radiosensitizes multiple cell lines to both photons and protons (Fig1A-H). Protons+PARPi provide the largest sensitization compared to all other treatments (Fig1J-M). The effect of PARPi on RBE either provides a modest increase (5%) or a significant decrease (-10%) in RBE (Fig1N-Q). MN (Fig2A-D) and MN-cGAS^+^ (Fig2E-H) are enhanced by both photons and protons. However, protons+PARPi increases MN-cGAS^+^ compared to all other treatment combinations.

**Conclusion:** Protons+PARPi enhance cell kill and are associated with precursors of anti-tumor immune signaling. Additional investigation is warranted to evaluate if these effects are seen in vivo.

## P206 -

### Can micronuclei be used as a marker of relative biological effectiveness?

#### Charlotte Heaven^1,2^, Hannah C. Wanstall^1,2^, Nicholas T. Henthorn^1,2^, John W. Warmenhoven^1,2^, Samuel P. Ingram^1,2^, Amy L. Chadwick^1^, Elham Santina^1^, Jamie Honeychurch^1^, Christine K. Schmidt^1^, Karen J. Kirkby^1,2^, Norman F. Kirkby^1,2^, Neil G. Burnet^2^, Michael J. Merchant^1,2^

##### ^1^University of Manchester, Division of Cancer Sciences, Manchester, United Kingdom ^2^The Christie NHS Foundation Trust, Manchester Academic Health Science Centre, Manchester, United Kingdom

Micronucleus (MN) formation is routinely used as a biodosimeter for acute radiation exposures. Strongly correlating with dose, MN are suggested to indicate radiation modality, differentiating between particle and photon irradiation. The “gold standard” for measuring MN formation is Fenech's cytokinesis-block micronucleus (CBMN) cytome assay. Here we present a comprehensive analysis of the literature investigating MN induction trends *in vitro,* collating 201 publications, with 2,572 data points. Particular attention was given to cell type, dose, linear energy transfer (LET), and radiation modality (photons and heavy ions), to establish whether MN can be used as a marker of DNA damage and relative biological effect (RBE). Analysis revealed two different measures of MN: MN per cell and percentage of cells containing MN. The former was more commonly reported; however the latter produced more consistent results between studies. Overall, there was an increase in MN formation with dose, although the exact relationship could be determined. Due to large inter-study variation, differentiations could not be made between radiation modality, cell type, or LET. The concentration of cytochalasin-B used in the CBMN assay had no effect on MN induction; however, excluding studies that deviated from the CBMN protocol reduced variation. Our analyses imply that changes due to physical (modality, LET) and biological (cell type) factors are lost in the noise of processing and interpretation. Therefore, the current literature cannot be used to establish a MN RBE for protons or other ionising radiation. Future studies should use the standardised assay to better probe these, as yet, unclear relationships.

## P207 -

### ATR inhibition disrupts DNA damage and cell cycle response to proton and photon radiation

#### Mandira Manandhar^1^, Scott James Bright^1^, Broderick X. Turner^1^, David B. Flint^1^, Mariam Ben Kacem^1^, Simona F. Shaitelman^1^, Gabriel O. Sawakuchi^1^

##### ^1^UT MD Anderson Cancer Center, Radiation Physics, Houston, USA

**Purpose:** There is interest in combining DNA repair inhibitors with radiotherapy to treat radioresistant tumors. We hypothesized that combining radiation with AZD6738, an inhibitor of ATR (ATRi), radiosensitizes cancer cells due to ATR's role in DNA repair and the cell cycle, and that this effect is greater for protons compared to photons due to the more complex DNA damage induced by protons.

**Method:** H1299 cells were treated with 0.1 or 1 μM AZD6738 for 1 h prior to irradiation. Cells were exposed to 6 MV x-rays or 9.9 keV / protm protons (dose-weighted LET in water) for clonogenic cell survival and to evaluate for mitosis (Phosho-Histone 3) and cell cycle stages (flow cytometry).

**Results:** ATRi radiosensitized both photons and protons (Figure 1A-B). The relative biological effectiveness (RBE) had a non-significant increase for protons + ATRi comperd to protons alone (Fig 1C). At 6 h after radiation, photons and protons arrested cells in G2, and 1 μM but not 0.1 μM of ATRi abrogated G2 arrest (Figure 2A). Significantly higher amounts of mitotic cells were observed after protons + ATRi (1 μM) versus photons + ATRi (1 μM) (Figure. 2B).

**Conclusions:** Induction of robust G2 checkpoint activation was observed after photons and protons alone. Protons + ATRi disrupted the cell cycle checkpoint and increased the proportion of cells in mitosis. Our results suggest that ATRi is effective at inducing sensitivity of cancer cells to radiation, a phenomenon that may be due to the role of ATRi on cell cycling.

## P208 -

### Estimation of Relative Biological Effectiveness on proton and 4He Spread-Out Bragg Peaks with Fluorescent Nuclear Tracks Detectors

#### Ivan Muñoz^1,2^, Lucas Burigo^1,3^, Steffen Greilich^1,4^, Oliver Jäkel^1,3,5^

##### ^1^German Cancer Research Center - DKFZ, Division of Medical Physics in Radiation Oncology, Heidelberg, Germany ^2^Heidelberg University, Faculty of Physics and Astronomy, Heidelberg, Germany ^3^German Cancer Research Center - DKFZ, Heidelberg Institute for Radiation Oncology - HIRO, Heidelberg, Germany ^4^Berthold Technologies GmbH, Radiation Protection and Bioanalytics, Bad Wildbad, Germany ^5^University Hospital Heidelberg, Heidelberg Ion Beam Therapy Center - HIT, Heidelberg, Germany

**Aim**: To develop a novel method to assess Relative Biological Effectiveness (RBE) distributions on proton and ^4^He Spread-Out Bragg Peaks (SOBPs) from the Linear Energy Transfer (LET) spectra measured with Fluorescent Nuclear Track Detectors (FNTDs).

**Methods**: FNTDs were exposed behind various thicknesses of RW3-plastic to proton and ^4^He SOBPs (10.0 - 15.0 cm depth). FNTDs were scanned with a dedicated reader and the LET-spectra were determined from the mean fluorescence intensity of single ion-tracks. The RBE was calculated using two published Phenomenological Models (PMs), consisting of fitted curves to in-vitro data, and the Microdosimetric Kinetic Model (MKM). The dose-average LET (protons) and track-average LET (^4^He-ions) were used as free-parameters on the PMs. The lineal energy distributions, required by the MKM, were generated from the LET-spectra and the chord-length probability distribution (sphere of 5.0 μm radius). Results were compared with Monte Carlo simulations using TOPAS.

**Results**: In all cases, the RBE increases towards the distal edge of the SOBPs, with a more pronounced increase for ^4^He-ions. For protons, the RBE varies from 1.08 to 1.21 and from 0.97 to 1.25 for the PM and MKM, respectively. In the case of ^4^He-ions, the RBE ranges from 1.22 to 1.67 and from 1.09 to 1.64 for the PM and MKM, respectively. For the distal-penumbra of the ^4^He SOBP, the RBE decreases as the radiation field is primarily composed of secondary protons. Differences between the PMs and the MKM can be partly attributed to the different reference radiation originally used to establish the RBE.

## P209 -

### Differential generation of DNA double strand breaks along a proton Bragg peak as revealed by atomic force microscopy

#### Dalong Pang^1^, Cara Frame^1^, Yuan Yuan^1^, Tianjun Ma^1^, Yu Chen^1^, Anatoly Dritschilo^1^

##### ^1^Georgetown University Hospital, Radiation Medicine, Washington, USA

**Purpose**: To investigate proton RBE in DNA double strand breaks (DSB) produced by therapeutic proton beam at different positions of Bragg peak and to correlate DSB as a direct cause of the RBE for cell survival

**Materials and Methods**: puC19 plasmid DNA in solution was irradiated on a Mevion proton system to doses of 0.5, 1 and 2 kGy at different positions of a 110 MeV pristine proton beam. The radiation beam monitor units (MU) were adjusted so that the doses delivered at the Bragg peak remain the same as at the plateau. The irradiated DNA samples were imaged on an atomic force microscope for measurement of DNA fragment lengths. The lengths of the DNA fragment were measured and ranged from a few nanometers to 850 nm. The fragments were subsequently grouped into length bins in 50 nm interval to construct DNA fragment length distribution profiles.

**Results**: At each of the doses delivered, DNA irradiated at the Bragg peak exhibited much greater DNA fragmentation with more broken and shorter DNA fragments than that produced in the plateau; the amount of broken fragments increases with dose, demonstrating a clear effect of the differential LET in DSB induction.

**Conclusions**: The varying LET of a Bragg peak leads to observable differences in DNA DSB induction. At the Bragg peak the induced DSBs are substantially more than that produced in the plateau and correlate to shorter DNA fragments. This difference in DSB generation may be correlated to the observed enhancement of RBE for cell survival.

## P210 -

### Laser-driven ultra-high dose rate protons: platform technology development for radiobiological research.

#### Antoine Snijders^1^, Jianhui Bin^2^, Lieselotte Obst-Huebl^2^, Jian-Hua Mao^1^, Hang Chang^1^, Kei Nakamura^2^, Qing Ji^2^, Zachary Kober^2^, Anthony Gonsalves^2^, Stepan Bulanov^2^, Carl Schroeder^2^, Cameron Geddes^2^, Eric Esarey^2^, Eleanor Blakely^1^, Sven Steinke^2^

##### ^1^Lawrence Berkeley National Laboratory, Biological Systems & Engineering, Berkeley, USA ^2^Lawrence Berkeley National Laboratory, Accelerator Technology and Applied Physics Division, Berkeley, USA

Radiotherapy is the standard of care for more than 50% of all cancer patients. Improvements in radiotherapy (RT) technology have increased tumor targeting and normal tissue sparing, especially with the recent interest in ultra-high dose rates required for FLASH-RT effects. Using Berkeley Lab's BELLA PW laser facility, we have developed the capabilities to investigate the radiobiological effects of ultra-high dose rate (10^7 Gy/s), laser-driven (LD) proton bunches with energies ∼ 2 MeV. We will describe BELLA beamline modifications to transport ions away from the existing BELLA laser-target interaction zone to a custom-built sample holder and we present the first radiobiological results using our new experimental platform to deliver LD proton bunches at 0.2 Hz repetition rate to cell monolayers grown over a 1 cm diameter field. Dose-dependent cell survival measurements of human normal and tumor cells exposed to either LD protons or conventional 300 kVp X-rays will be presented. Our findings provide evidence that compact laser-driven proton sources enable a new and promising platform for investigating the physical, chemical and biological mechanisms underlying the FLASH effect. The work was supported by Laboratory Directed Research and Development (LDRD) funding from Lawrence Berkeley National Laboratory provided by the Director, and the U.S. Department of Energy Office of Science Offices of High Energy Physics and Fusion Energy Sciences, under Contract No. DE-AC02-05CH11231. Work at BELLA was also supported by LaserNetUS (https://www.lasernetus.org/).

## P211 -

### Protons combined with an inhibitor of ATR amplify micronuclei and potentiate anti-tumor immune signaling

#### Broderick Turner^1^, Scott Bright^1^, David Flint^1^, Conor Mcfadden^1^, Gabriel Sawakuchi^1^, Simona Shaitelman^1^

##### ^1^University of Texas at MD Anderson Cancer Center, Radiation Physics, Houston, USA

**Purpose**: The cGAs-STING pathway results in downstream activation of cytokines and signals that can lead to antitumor immunity. Here we show that the combination of protons with an inhibitor of ATR (ATRi), a key protein in DNA damage response, amplify the production of precursors to antitumor immune activation, specifically cGAS-positive micronuclei (MN-cGAS^+^), more so than photons+ATRi.

**Methods**: H1299 and PANC-1 cell lines were treated with 6 MV x-rays and 9.9 keV/mm (dose-weighted LET in water) protons alone or with an ATRi (1 μM, AZD6738). We then assessed MN snd MN-cGAS^+^ 24 and 72 h after radiation+ATRi. Micronuclei and cGAS positive nuclei were assessed a fraction of total nuclei and were counted using a custom ImageJ macro to auto-count.

**Results**: Protons+ATRi amplify MN compared to photons alone, protons alone and photons+ATRi for both cell lines (H1299 and PANC-1), timepoints (24 and 72 h) and doses (2 and 5 Gy) (Fig 1A-D). Importantly, these trends also persist for MN-cGAS^+^ (Fig 2A-D).

**Conclusions**: Combining protons with ATR inhibition amplify cGAS-positive micronuclei, which has been reported as anti-tumor immune signaling. Additional in vivo research is warranted to evaluate the potential of radiation with DNA repair inhibition to activate antitumor immunity in these otherwise aggressive tumors.

## P212 -

### Evaluation of cell sensibilization properties of boron-containing compounds under proton beam irradiation.

#### Georgiy Andreev^1^, Eva Kuus^1^, Nikolay Verlov^2^, Nikolay Vorobuyev^1^, Alexey Mikhailov^1^, Andrey Lyubinskiy^1^, Andrey Burdakov^2^, Andrey Konevega^2^, Ivan Plugar^1^, Natalia Martynova^1^, Roman Frolov^1^, Denis Antipin^1^

##### ^1^MIBS, Proton therapy, Saint-Peteresburg, Russian Federation ^2^Petersburg Nuclear Physics Institute named by B.P.Konstantinov of national research center “Kurchatov institute', Cell Biololgy, Saint-Petersburg, Russian Federation

Proton therapy is one of the most effective types of modern radiation therapy techniques. Up to date a variety of treatment protocols developed for many different types of tumors which are widely used internationally. To evaluate the efficiency of such protocols and for their further optimization and better safety it is necessary to thoroughly evaluate mechanisms of cell death, which is induced by the different doses of radiation, and as well estimate indirect effect of ionizing radiation on the different organs and entire organism. Also radiomodificators action in radiotherapy is of a big interest for the purpose of increase of tumor cells radiosensitivity and normal tissues radioresistance. In this study we have irradiated with protons human glioblastoma cells (A172 and Tr) with and without addition of sodium borocaptate (BSH) on the Varian Probeam therapy system with a single dose of 2, 4, 6, 8, 10 and 20 Gy. After irradiation the following parameters were estimated: size of cell population, cell morphology (microscopy), cell metabolism (MTT-test). Within the first stage of experiments dose-response curves for selected cell lines were built. Data recieved from population cell growth analysis and MTT-test confirmed reverse exponential dependence of cell surviaval from recieved dose of ionizing radiation. Substantial morphological changes of the cells in population were observed with doses higher than 4 Gy. Sodium borocaptate addition didn't cause reliable shift of dose-effect curve comparing to the control group of cells. A172 cells have shown a slight trend towards increase of radioresistance when adding BSH.

## P213 -

### DNA repair inhibitors modulate cancer cell survival to photons and protons

#### Scott Bright^1^, David Flint^1^, Broderick Turner^1^, Mandira Manandhar^1^, Conor Mcfadden^2^, David Martinus^1^, Mariam Ben Kacem^1^, Simona Shaitelman^3^, Gabriel O Sawakuchi^1^

##### ^1^MD Anderson, Radiation Physics, Houston, USA ^2^UT Southwestern, Cell Biology, Dallas, USA ^3^MD Anderson, Radiation Oncology, Houston, USA

Radiotherapy's (RT) primary mechanism of action is DNA damaged induced cell death. Thus, inhibitors of DNA damage response (DDRi) combined with RT has the ability to enhance the RT's cytotoxic effects. To date, research has been focused on combining DDRi with photons, whilst combinations with protons have remained understudied. We hypothesized that protons+DDRi offers greater sensitization than photons+DDRi. We investigated the response of 4 cancer cell lines: NCI-H460, NCI-H1299, PANC-1 and Panc 10.05 treated with 5 DDRis targeting ATM (KU55933), ATR (AZD6738), DNA-PKcs (NU7441), PARP (AZD2281) and Rad 51 (B02), at various concentrations (0.1-10 μM) and irradiated with 6 MV x-rays or 9.9 keV/μm protons (dose-weighted LET in water). Cell survival was measured and fit to the linear quadratic model, from which we quantified the dose at the 10% survival fraction (D_10%_), total radiosensitivity enhancement (TRE at D_10%_ = D_10%,DMSO, photon_/D_10%,DDRi, photons or protons_) and relative biological effectiveness (RBE at D_10%_ = D_10%,DDRi,photons_/D_10%,DDRi,protons_). We observed that DDRis radiosensitize both photons and protons (Fig1A-B). TRE was increased up to 3.51±0.09 for protons+ATMi (10 μM) (Fig1C-D). RBE ranged from 0.79±0.03 to 1.54±0.09 (Fig1E) and were negatively correlated with photon TRE (r= -0.6160) (Fig2A), suggesting that the greater the DDRi-induced sensitization, the less benefit there will be from the proton LET effect. Nonetheless, the TREs from combining DDRi with protons were generally larger (Fig2B) (30±3% on average), suggesting that even when DDRi reduce proton RBE, there is still a large net radiosensitivity gain when combining them with protons, potentially offering greater clinical benefit.

## P214 -

### Boron neutron capture therapy: A review of clinical applications

#### T. Malouff^1^, D. Seneviratne^1^, D. Ebner^2^, W. Stross^1^, M. Waddle^2^, D. Trifiletti^1^, S. Krishnan^1^

##### ^1^Mayo Clinic, Radiation Oncology, Jacksonville, USA ^2^Mayo Clinic, Radiation Oncology, Rochester, USA

Boron neutron capture therapy (BNCT) is an emerging treatment modality aimed at improving the therapeutic ratio for traditionally difficult to treat tumors. BNCT utilizes boronated agents to preferentially deliver boron-10 to tumors, which, after undergoing irradiation with neutrons, yields litihium-7 and an alpha particle. The alpha particle has a short range, therefore preferentially affecting tumor tissues while sparing more distal normal tissues. To date, BNCT has been studied clinically in a variety of disease sites, including glioblastoma multiforme, meningioma, head and neck cancers, lung cancers, breast cancers, hepatocellular carcinoma, sarcomas, cutaneous malignancies, extramammary Paget's disease, recurrent cancers, pediatric cancers, and metastatic disease. We aim to provide an up-to-date and comprehensive review of the studies of each of these disease sites, as well as a review on the challenges facing adoption of BNCT.

## P215 -

### Acute skin reaction of BNCT

#### Kei Nakai^1^, Masatoshi Nakamura^1^, Teruhito Aihara^2^, Tetsuya Yamamoto^3^, Yoshitaka Matsumoto^1^, Hiroaki Kumada^1^, Akira Matsumura^4^, Sakurai Hideyuki^1^

##### ^1^University of Tsukuba, Department of Radiation Oncology- Faculty of Medicine, Tsukuba, Japan ^2^Osaka Medical College, Kansai BNCT Medical Center, Takatsuki- Osaka, Japan ^3^Yokohama city University School of Medicine, Department of Neurosurgery, Yokohama, Japan ^4^Ibaraki Prefectural University, Department of of Health Sciences, Ami- Inashiki, Japan

**Introduction**: We had treated glioblastoma patients, and recurrent head and neck cancers by BNCT. Patients were treated using a Reactor, and recently, we had a chance to observe 4 cases of acute skin reaction of Accelerator-based BNCT. This report is focusing on skin damage after the BNCT.

**Material and Methods**: We had treated 17 cases of glioblastoma from 1999 to 2010. 4case of recurrent head and neck cancer in 2014. 4cases of recurrent head and neck cancer in 2020 at Southern Tohoku BNCT research center. A retrospective analysis from the medical chart and summarized documentation.

**Result and Discussion**: In the brain tumor BNCT, the most common acute adverse event was mild erythema (CTC-AE Grade 1), and alopecia was prolonged. In the latter protocol, a patient tolerated additional 40Gy external X-ray irradiation. In the H&N cancer patient who underwent reactor-based BNCT, all cases had the previous radiotherapy. Acute radiation dermatitis was observed in all cases. 3 cases were Grade 1, 1 case developed Grade 2. In 2020, 4patients underwent accelerator-based BNCT at Sothern Tohoku BNCT research center. Patients developed transient hair loss, more extensive than the beam port diameter. All patients recovered within 3 months, but the cause of widespread hair loss has not been adequately discussed yet.

**Conclusion**: Previous reactor-based BNCT cases were retrospectively analyzed. Acute radiation dermatitis was generally tolerable but careful planning and observation are required. Recent accelerator-based BNCT developed transient hair loss, the cause was not clear.

## P216 -

### Application and follow up for BNCT in four patients with recurrent Head and Neck Cancer

#### Masatoshi Nakamura^1^, Kei Nakai^1^, Keiichiro Baba^1^, Takashi Saitoh^1^, Ayako Okawa^1^, Masahiro Nakayama^2^, Kayoko Ohnishi^3^, Kenji Yamagata^4^, Masashi Mizumoto^1^, Toshiyuki Okumura^1^, Hideyuki Sakurai^1^

##### ^1^Department of Radiation Oncology- Faculty of Medichine- University of Tsukuba, Radiation Oncology, 1-1-1- Tennodai- Tsukuba- Ibaraki, Japan ^2^Department of Otolaryngology- Head and Neck Surgery- Faculty of Medicine- University of Tsukuba, Otolaryngology, 1-1-1- Tennodai- Tsukuba- Ibaraki, Japan ^3^Department of Radiology- School of Medicine- International University of Health and Welfare, Radiology, 4-3- Kozunomori- Narita- Chiba, Japan ^4^Department of Oral and Maxillofacial Surgery- Institute of Clinical Medicine- Faculty of Medicine- University of Tsukuba, Oral and Maxillofacial Surgery, 1-1-1- Tennodai- Tsukuba- Ibaraki, Japan

Japanese FDA approved BNCT for recurrent head and neck cancers on June 2020. In our hospital, weekly head and neck cancer conferences are held.In 2020, 160 newly diagnosed cases and 113 cases of follow-up and recurrence were discussed treatment strategies. Of the cases of recurrence, we consulted to southern TOHOKU hospital 5 cases in 2020 and 3 patients were underwent BNCT, and one case referred from outside the hospital and was also treated. All cases have been followed up at the BNCT facility and the referral hospital. (1)80y M of tongue cancer of T3N3bMo. first treated 70Gy radiotherapy, residual and regrowth tumor were treated with BNCT 8mo after the XRT. CR for 8mo. (2)84y M of larynx cancer of cT2N2bM0 treated with 66Gy/33fr. For primary and lymph node recurrence, BNCT was performed 2 years after initial treatment. CR for 7mo. (3)77y F, Gingival carcinoma of mandibular, first treatment was surgical removal and reconstruction and chemo-radiotherapy (50Gy/25fr). For local recurrence, BNCT was performed 5years after surgery. CR for 6mo. (4) 68y M, maxillary sinus cancer, cT4aN2bM0, BNCT was performed 10month after CCRT (CDDP and XRT 70Gy/35fr) for primary regrowth. He recurred 4mo after BNCT and started anticancer drugs. In this case, inflammation and infection occurred in the irradiated area. Although there are still a small number of cases and short follow up periods, 3 from 4cases kept good condition more than 6mo. The BNCT is intended for patients with advanced cancer, and adverse events are considered to be acceptable.

## P217 -

### 18F-borono-Lphenylalanine Positron Emission Tomography (18F-BPA PET) scans - their roles and future in Oncology

#### Daniel Quah^1^, Si Xuan Koo^2^

##### ^1^National Cancer Centre Singapore, Radiation Oncology, Singapore, Singapore ^2^Singapore General Hospital, Department of Nuclear Medicine and Molecular Imaging, Singapore, Singapore

Boron neutron capture therapy (BNCT) is a non-invasive therapeutic alternative or adjunctive option for head and neck cancers, high grade gliomas and some melanomas. A pre-requisite for BNCT is the effective delivery of Boron-10 into tumour cells via boron-containing compounds that operate on the premise of tumour cell nuclei being highly attractive to biomolecules (such as amino acids and sugars [Boronophenylalanine-fructose]). The 4-borono-2-18F-fluoro-phenylalanine (18F-BPA) Positron Emission Tomography (PET) scan allows quantification of the tissue distribution of the boron-containing compound to better estimate subsequent BNCT outcome. 18F-BPA PET scans also show good spatial correlation with 18F-FDG scans, suggesting that the efficacy of BNCT may not be hampered in biologically active, more aggressively proliferating sub volumes of tumours. This review aims to validate 18F-BPA PET scans' efficacy in predicting BNCT treatment outcomes. In addition, we will also discuss the potential role of these and similar scans in other diagnostic and therapeutic arenas, for both local and international patients.

## P218 -

### The effects of hypoxia and starvation on U2OS cells

#### Anggraeini Puspitasari^1,2^, Martina Quartieri^1^, Beatrice Cambien^3^, Angélique Martelli^3^, Julius Oppermann^1^, Charnay Chuningham^4^, Fabio Squarcio^5^, Olga Sokol^1^, Marco Durante^1^, Walter Tinganelli^1^

##### ^1^GSI Helmholtzzentrum für Schwerionenforschung GmbH, Biophysics, Darmstadt, Germany ^2^Gunma University, Heavy Ion Medical Center, Maebashi, Japan ^3^UMR E4320- Federation Claude Lalanne- Université Côte d'Azur, Faculté de médecine, Nice, France ^4^iThemba Labs, Laboratory for Accelerator-Based Science, Cape Town, South Africa ^5^University of Bologna, Department of Biomedical and Neuromotor Sciences, Bologna, Italy

Radiotherapy is the least invasive method to treat cancer. However, although local control in early-stage malignancies easily exceeds 90%, overall survival remains dismal due to distal metastasis. Cancer cells with unregulated growth, metabolism, and lack of contact inhibition create an incomplete and inefficient tumor architecture that generates large areas where oxygen and nutrients are scarce. Under these stressor conditions, such as hypoxia and starvation, cancer cells may change their characteristics to escape from the primary tumor, enter the bloodstream to form metastatic deposits or re-establish themselves in the cancer's primary site. The underlying mechanisms behind the development of these cells are still poorly understood. In this study, we investigated the behaviour of acute (one day) and long-term (four weeks) effects of hypoxia (1%), starvation (5%FBS, serum starvation) and radiation on an osteosarcoma cell line U2OS. We performed a cells aggregation study to see how the cells behave and looked into transmembrane and actin-binding proteins expression in condition with and without stressors. We found that the cells under hypoxic condition showed fewer aggregates and alteration of the adhesion protein.

## P219 -

### Determination of the domain radii for different cell lines in the context of the Microdosimetric Kinetic Model

#### Alejandro Bertolet^1^, Alejandro Carabe-Fernández^2^

##### ^1^Massachusetts General Hospital, Radiation Oncology, Boston, USA ^2^Hampton University Proton Center Institute, Physics, Hampton- VA, USA

**Purpose:** To determine cell line-specific radiobiological domain sizes based on the Microdosimetric Kinetic Model (MKM). A model for the domain radius serves to introduce a relative biological effectiveness (RBE) model generally applicable to ion therapy.

**Methods and material:** The MKM postulates the concept of domain as representative of the maximum distance for sublethal lesions to pairwise interact to form a lethal lesion. Data from clonogenic assays compiled in the Particle Irradiation Data Ensemble (PIDE) database were analyzed to determine the domain size for a set of different cell lines using the parameters α and β from fitting the linear-quadratic model to the survival fraction.

**Results:** Domain radii were determined for 61 cell lines, including 39 human tumor cells, 7 human normal cells, 6 rodent tumor cells and 9 rodent normal cells. We found relations between domain size and α/β ratio for x-rays; and linear energy (y) and LET, enabling a general RBE model depending only on the LET and the α and β parameters.

**Conclusions:** Determining the domain size for different cell lines is possible from clonogenic assays and allows to adapt the MKM model to factor individual radiosensitivity in. Domain radii can be generally formulated as a function of clonogenic survival parameters.

## P221 -

### The BIANCA model provides reliable RBE predictions for different cells irradiated by protons, C-ions and He-ions in treatment-like scenarios

#### Mario Pietro Carante^1^, Alessia Embriaco^1^, Giulia Aricò^2^, Alfredo Ferrari^3^, Christian P. Karger^4^, Wioletta Kozlowska^5^, Andrea Mairani^6^, Stewart Mein^6^, Ricardo Ramos^1^, Paola Sala^7^, Francesca Ballarini^8^

##### ^1^INFN Italian National Institute of Nuclear Physics, Section of Pavia, Pavia, Italy ^2^CERN, European Organization for Nuclear Research, Geneve, Switzerland ^3^University Hospital Heidelberg and Gangneung-Wonju National University, Physics Department, Milano, Italy ^4^German Cancer Research DKFZ, Department of Medical Physics in Radiation Oncology, Heidelberg, Germany ^5^Cern and Medical University of Vienna, Physics Department, Geneve, Switzerland ^6^Heidelberg University Hospital, HIT Heidelberg Ion-beam Therapy center, Heidelberg, Germany ^7^INFN Italian National Institute of Nuclear Physics, Section of Milano, Milano, Italy ^8^University of Pavia and INFN Italian National Institute of Nuclear Physics, Physics Department- Section of Pvaia, Pavia, Italy

BIANCA (*BIophysical ANalysis of Cell death and chromosome Aberrations*) is a two-parameter biophysical model assuming that radiation induces DNA “critical lesions” that produce chromosome aberrations, some of which lead to cell death. To perform RBE predictions for hadron therapy, first we tuned the model parameters to produce a radiobiological database for V79 cells, chosen as a reference. Afterwards, we developed an approach to produce analogous databases for other cell lines, for which the photon response is known. This approach does not require any further parameter adjustment, thus providing full predictions for, in principle, any cell line of interest. These databases can be read by a radiation transport code and/or a treatment planning system (TPS). After interfacing BIANCA with the FLUKA MC transport code, this approach was validated on *in vitro* cell survival and RBE data for different cell lines exposed to protons and C-ions (Picture), as well as *in vivo* animal data. More recently, the work was extended to He-ion irradiation, following the renewed interest for these ions in cancer therapy. More specifically, very good agreement was found with *in vitro* data on the survival of CHO cells exposed at different positions along a mono-directional Spread-Out Bragg Peak of ^4^He ions, and with survival curves of renal adenocarcinoma cells along both a pristine peak and a SOBP. This work suggests that BIANCA provides reliable predictions for different cell lines exposed to different ions, and can represent a valuable approach for the biological optimization of patient treatment, including future He-ion treatments.

## P222 -

### Validation of Geant4 Electromagnetic physics models for RBE-weighted dose calculation of the heavy-ion therapy

#### Hyo Kyeong Kang^1^, Yongdo Yoon^2^, Min Cheol Han^2^, Jin Sung Kim^2^

##### ^1^Yonsei University College of Medicine, Department of Integrative Medicine, Seoul, Korea Republic of ^2^Yonsei University College of Medicine- Yonsei Cancer Center, Department of Radiation Oncology, Seoul, Korea Republic of

**Purpose:** Geant4 has become widely utilized to calculate the model-based relative biological effectiveness (RBE) dose distribution for heavy-ion therapy. Unfortunately, electromagnetic (EM) physics models used in Geant4 is not validated for microdosimetric kinetic model (MKM) yet. In this study, we validated various EM physics models included in Geant4 by calculating microdosimetric energy deposition.

**Methods:** To validate the EM physics models in Geant4, a cylindrical volume used for the MKM was constructed, which represents a HSG cell. Subsequently, the corresponding parameters, e.g. saturation-corrected specific energy (Z_sat) and saturation-corrected dose-mean-specific energy (Z*_1d), were calculated for each mono energies of various particles. The Geant4 simulations were run separate times with different EM physics models. The simulation results were compared with published data from Inaniwa et al. (2010).

**Results:** The validation process was successfully implemented in Geant4 (Figure 1), and our tests showed that Z_sat and Z*_1d calculated by all EM physics models used in this study are statistically consistent with published data in low-energy regions for heavy-ion particles, but in the range of specific energies (>1 MeV/u), G4EMStandardPhysics_option3 model had discrepancies comparing with the published data.

**Conclusion:** Our validation results would allow selecting the EM physics models to calculate the MKM-based dose calculation in Geant4 and other Geant4-based codes, such as TOPAS. More detailed results for the other assessments including various EM physics options will be presented at 2021 PTCOG annual meeting.

## P223 -

### Risk of radiation induced second primary cancer for left sided breast radiotherapy

#### Alexandre Santos^1,2^, Andreas Kotsanis^1^, Lisa Cunningham^2,3^, Scott Penfold^1,2^

##### ^1^The University of Adelaide, School of Physical Sciences, Adelaide, Australia ^2^Royal Adelaide Hospital, Department of Radiation Oncology, Adelaide, Australia ^3^University of South Australia, Allied Health and Human Performance, Adelaide, Australia

**Introduction**: Proton therapy has been proposed as a technique to improve the long-term quality of life of breast cancer patients. The purpose of this study was to investigate the risk of radiation induced second cancer induction due to IMPT compared to hybrid IMRT (h-IMRT) for left sided breast treatments.

**Methods**: In this study, 15 female left-sided whole breast cancer patients were scanned in DIBH. The Pinnacle treatment planning system was used to simulate the two plans for each patient. Using the dose-volume histograms (DVHs) from these plans, the mean lifetime attributed risk (LAR) for both lungs and the contralateral breast were evaluated using the BEIR VII and Schneider full risk models.

**Results**: The IMPT treatment plans result in less dose to the lungs and contralateral breast then that of the h-IMRT, shown in figure 1. The results from both risk models show lower LAR estimates for the IMPT treatment plan compared to the h-IMRT treatment plan. This result was observed for all organs of interest and was consistent amongst the two separate risk models. For both treatment plans, the organs from most to least at risk were: ipsilateral lung, contralateral breast and contralateral lung. In all cases, the risk estimated via the BEIR VII model was higher that the Schneider full risk model.

**Conclusion**: The use of proton therapy for breast treatments leads to reduced risk estimates for radiation induced secondary cancers. Therefore proton therapy shows promise in improving the long term treatment outcome of breast patients.

## P224 -

### Simple ion-type-independent RBE modelling is tested for in-vivo data

#### Liheng Tian^1^, Lauritz Kluender^1^, Armin Lühr^1,2^

##### ^1^Technische Universität Dortmund, Medical Physics, Dortmund, Germany ^2^Technische Universität Dresden- Helmholtz-Zentrum Dresden-Rossendorf, OncoRay- National Center for Radiation Research in Oncology- Faculty of Medicine and University Hospital Carl Gustav Carus, Dresden, Germany

Recent clinical evidence for varying relative biological effectiveness (RBE) raised concern in proton therapy, while decades of clinical experience with variable RBE exist for carbon ion therapy. In contrast to linear energy transfer (LET), using beam quality Q = Z^2^/E (Z = ion charge, E = energy) lead to simple RBE modeling being ion-type-independent in vitro. This work investigates the feasibility of combined Q-based RBE modelling for protons and carbon ions in vivo. Published rat spinal cord dose response data (Saager et al.) were analyzed for proton (2 fractions) and carbon (1, 2, 6 fractions) irradiations at 4 and 6 SOBP positions, respectively, and compared to corresponding photon irradiations. Monte Carlo simulations provided dose-averaged Q values at each irradiation position. Using the F_E_ method (Fig. 1A, C), the Q-dependencies of the linear quadratic model parameters β and α/β were determined from which a Q-dependent RBE model was derived. For carbon irradiations, the slopes found in the F_E_ plot and, thus, β values did not differ significantly for different Q values (Fig. 1). Consequently, β was assumed to be constant, which resulted in α/β as function of Q following a common trend for protons and carbon ions. The resulting RBE values for proton and carbon ions reproduced experimental data well with difference < 0.1 (one standard deviation) (Fig. 2). Ion-type-independent RBE modeling based on Q and clinical carbon ion experience may be a simple alternative to empirical LET-based RBE models in future proton therapy. Validation with other ions is advised.

## P225 -

### Determination of hypofractionated dose scheme for prostate tumor based on tumor control probability in carbon ion radiotherapy.

#### Yushi Wakisaka^1^, Masashi Yagi^2^, Toshiro Tsubouchi^3^, Noriaki Hamatani^3^, Masaaki Takashina^3^, Tatsuaki Kanai^3^

##### ^1^Osaka Heavy Ion Therapy Center, Department of Radiation Technology, Osaka, Japan ^2^Osaka University Graduate School of Medicine, Department of Carbon Ion Radiotherapy, Suita, Japan ^3^Osaka Heavy Ion Therapy Center, Department of Medical Physics, Osaka, Japan

**Introduction**: This study aims to obtain parameters of linear-quadratic (LQ) model by tumor control probability (TCP) analysis, which will lead to estimate the prescription dose for hypofractionated carbon ion radiotherapy (CIRT) for prostate tumor.

**Methods**: TCP was expressed using LQ coefficients α and β, their standard deviations σ_α_ and σ_β_, and the number of initial clonogenic cells N_0_ which are different for each risk-group of prostate tumors. TCP model was fitted by the previously reported 5-year biochemical progression-free survival (BFS) of X-ray radiotherapy (XRT) and CIRT, then we obtained N_0_ for each risk-group and α, β, σ_α_, σ_β_ for XRT and CIRT. The relative biological effectiveness for CIRT was fixed to 2.435 at the center of spread-out Bragg peak. The accuracy of the fitting was evaluated by root mean squared error (RMSE) and coefficient of determination (R^2^), weighted by the number of patients for each data point.

**Results**: Fig. 1 and 2 show the results of TCP analysis on XRT and CIRT respectively. Since the clinical data had different number of fractions, the dose was converted to equivalent dose in 2 Gy fraction (EQD2). The RMSE and R^2^ were 7.0 % and 0.84 for XRT, and 6.9 % and -1.32 for CIRT, respectively. In order to improve the accuracy of the TCP analysis for CIRT, we plan to add clinical data or conduct cell experiments in the future.

**Conclusion**: The LQ parameters were derived by TCP analysis. With additional experiments, we eventually aim to determine the prescription dose for hypofractionated CIRT for prostate tumor.

## P226 -

### Using the Hi-C technique to model DNA structure with different chromatin compaction in TOPAS-nBio

#### Dohyeon Yoo^1^, Nicolas Henthron^2^, Samuel Ingram^2^, Michael J. Merchant^2^, John Warmenhoven^2^, Aimee McNamara^1^, Karen Kirkby^2^, Kathryn D. Held^1^, Harald Paganetti^1^, Jan Schuemann^1^

##### ^1^Massachusetts General Hospital, Radiation Oncology, Boston, USA ^2^The University of Manchester, Division of Cancer Sciences- Faculty of Biology- Medicine and Health, Manchester, United Kingdom

TOPAS-nBio is an open-source MC application for nanodosimetry developed to advance our understanding of radiobiology effects at the (sub-)cellular scale. In the first beta release of TOPAS-nBio, a human fibroblast nucleus model was provided that includes chromatin territories with the same DNA density across the nucleus. However, the DNA density varies for each cell, chromatin territory and cell cycle, consisting of higher and lower DNA density (heterochromatin and euchromatin, respectively). The aim of this study is to design a realistic DNA geometry containing both heterochromatin and euchromatin. For this, we use a previously developed method of filling a nucleus with Hi-C technique using topologically associated domains (TADs) represented by spheres. Figure 1 shows a 3D rendered images of TADs for a Hi-C nucleus and the implemented in TOPAS-nBio. Hi-C data consist of several thousand TADs that classify the chromosome territories (shown by color). Each TAD (bead) was filled with voxels using three different voxel sizes (12, 24, and 48 nm) depending on the TAD radius to efficiently model DNA packing inside the TADs. Figure 2 shows how DNA is wrapped around a nucleosome located in the 12 nm voxels and how the TADs are connected. Various geometries of DNA packing will be placed inside the TADs to construct heterochromatin and euchromatin regions. This will allow studies of DNA damage considering the different chromatin compactions.

## P227 -

### Mouse skin damage after mixed beams of carbon ions is well predicted by knowing that after mono-peak beams

#### Yukari Yoshida^1^, Maika Yamaguchi^1^, Koichi Ando^1^, Akihisa Takahashi^1^, Tatsuaki Kanai^1^

##### ^1^Gunma University, Heavy Ion Medical Center, Maebashi- Gunma, Japan

Carbon-ion beams (C-ions) can deliver a high dose to a tumor while minimizing radiation damage to surrounding normal tissues because of their characteristic feature of dose concentration. As the dose to normal tissues increase in case the dose to tumor be increased, studies of normal tissue damage are indispensable. Additionally, carbon ion therapy uses a mixed beam that consists of various linear energy transfers (LETs). The aim of this study is to investigate the LET dependence of normal skin damage of beam mixing and to evaluate the predictive numeration. The right hind leg of C3H/He mice were irradiated with 290 MeV/nucleon C-ions in 1-4 fractions separated by 24 hours. C-ions used were mono-peak beams (13-71 keV/μm) and mixed beams (mixture of 15 and 71 keV/μm mono-peak beams). The dose response curves were obtained from the skin reaction data scored for up to 5 weeks. The survival parameters of α and β values were calculated by the linear-quadratic model. In mono-peak beams, a clear LET dependence of skin reaction was observed such that an increase in the LET shifted the dose response curves for skin reaction to the left. The isoeffective dose decreased with increasing LET. The α values linearly increased with increasing LET while the β values were almost constant. Survival parameters of mixed beams was in good agreement with the that of mono-peak beams. These results indicate that normal skin damage caused by mixed beams would well be predicted by the that caused by mono-peak beams.

## P228 -

### Effect of FLASH versus Non-FLASH proton radiation on human head and neck cancer cells and normal tongue cells

#### L. Wang^1^, M. Yang^2^, X. Wang^2^, Y. Zhang^1^, N. Sahoo^2^, X.R. Zhu^2^, S. Frank^3^

##### ^1^The University of Texas MD Anderson Cancer Center, Experimental Radiation Oncology, Houston, USA ^2^The University of Texas MD Anderson Cancer Center, Radiation Physics, Houston, USA ^3^The University of Texas MD Anderson Cancer Center, Radiation Oncology, Houston, USA

**Purpose:** FLASH-Radiotherapy (F-RT) has been identified as a promising modality to deliver curative dose to tumor instantaneously in milliseconds while mitigating normal tissue damage. Researches of F-RT are primarily on electron-FLASH, studies of FLASH- (F-PRT) versus conventional-proton radiotherapy (C-PRT) on human head and neck cancer (HNSCC) and normal cells are absent, these were investigated herein.

**Methods:** Radiation-induced hydrogen-peroxide (H2O2) in double-distilled water (ddH2O) were quantified (Amplex® Red Hydrogen-Peroxide Assay Kit, measuring relative fluorescence units [RFUs] at excitation 530 nm, emission 590 nm) after a 30-Gy of irradiation (F-PRT at 300-Gy/s; C-PRT at 0.592-Gy/s). HNSCC (HN5) and human normal tongue (NT, [Hs680.Tg]) cell lines were exposed to various single-dose radiation (F-PRT at 161-Gy/s [for >6Gy], or at 1012-Gy/s [for <6Gy]; C-PRT at 0.592-Gy/s). Cell viabilities were determined using MTT assay (measuring absorbance optical density at 560 nm).

**Results:** At 1.5 hours post-irradiation, C-PRT generated significantly more H2O2 versus F-PRT (174440.9±10983.0 versus 134145.6±9471.5 RFU; *P*=0.009). At 4 days post-irradiation, F-PRT (versus C-PRT) led to less cell viability in HN5 cells (3.4-Gy [0.79 times], 5.7-Gy [0.86 times], 10-Gy [0.90 times]); and similar or more cell viability in Hs680.Tg cells (3.4-Gy [1.02 times], 5.4-Gy [1.19 times], 8.3-Gy [1.33 times], 9.3-Gy [1.06 times]).

**Conclusion**: Compared with C-PRT, F-PRT induced less H2O2 in ddH2O, reduced cell viability in the HNSCC HN5 cells, and resulted in higher level of cell viability in the NT Hs680.Tg cells. Further studies using extended HNSCC and NT cell lines and animal models are warranted to validate these results.

## P229 -

### Impact of proton relative biological effectiveness in treatment planning study for patients with brain and skull base tumors

#### Magdalena Garbacz^1^, Jan Gajewski^1^, Renata Kopeć^2^, Nils Krah^3^, Marzena Rydygier^2^, Angelo Schiavi^4^, Emanuele Scifoni^5^, Tomasz Skóra^6^, Francesco Tommasino^7^, Antoni Rucinski^1^

##### ^1^Institute of Nuclear Physics Polish Academy of Sciences, Division of Applications of Physics, Krakow, Poland ^2^Institute of Nuclear Physics Polish Academy of Sciences, Cyclotron Center Bronowice, Krakow, Poland ^3^CNRS, Creatis, Lyon, France ^4^Sapienza University of Rome, Department of Basic and Applied Sciences for Engineering, Rome, Italy ^5^Trento Institute for Fundamental Physics and Applications, Istituto Nazionale di Fisica Nucleare, Trento, Italy ^6^National Institute of Oncology - National Research Institute, Krakow Branch, Krakow, Poland ^7^Trento Institute for Fundamental Physics and Applications, University of Trento, Trento, Italy

We will present the Monte Carlo (MC) retrospective study of biological dose computed with constant and variable relative biological effectiveness (RBE) model for brain and skull base patients treated at proton beam therapy center in Krakow. The purpose is to quantify the dose enhancement and especially to characterize extension in biological range when applying variable RBE (vRBE) model (McNamara et al. 2015). The oncological patient data were divided by tumor localization into two groups: brain and skull base, consisting of 50 and 45 patients, respectively. The MC calculations were performed using GPU-based MC engine FRED (Schiavi et al. 2017), which was commissioned and validated against measurements in Krakow. Dose volume histograms (DVHs) were analysed for planning target volume (PTV) and selected organs at risk (OARs). The dose deposited in PTV and OARs was compared to the clinical dose and dose constraints. The calculated doses were higher when considering variable RBE, as well as regions of the high dose (e.g. V95 values). Mean(std) biological range extension was 0.44(0.14) cm 0.45(0.11) cm for brain and skull base patients (see Fig.1), respectively. The worst-case (the largest range extension) DVHs for the patient from the skull base tumor group is presented in Fig.2. The presented results from dose calculations and robustness analysis shows that the modification of dose distributions when using the vRBE model can significantly modify the shape of the DVH curve and extend the biologically effective proton range.

## P230 -

### In vitro biological characterization of extremely small zinc-doped iron oxide nanoparticles for proton therapy verification with PET imaging

#### Marta Ibañez^1^, Irene Fernández-Barahona^2,3^, Marta Oteo^1^, Rocío Santacruz^1^, Víctor M. Luján^1^, Natalia Magro^1^, Fernando Herranz^3^, Miguel Ángel Morcillo^1^

##### ^1^Centro de Investigaciones Energéticas- Medioambientales y Tecnológicas CIEMAT, Unidad de Aplicaciones Biomédicas y Farmacocinética, Madrid, Spain ^2^Universidad Complutense de Madrid, Facultad de Farmacia, Madrid, Spain ^3^Instituto de Química Médica- Consejo Superior de Investigaciones Científicas CSIC, Grupo de nanomedicina e imagen molecular NanoMedMol, Madrid, Spain

Iron oxide nanoparticles are extensively explored magnetic materials with application in drug delivery, magnetic hyperthermia, photoacoustic and magnetic resonance imaging among others. The possibility of obtaining Gallium-64/66/68 after proton irradiation in the presence of zinc points out nanoparticles as a way for dose verification by PET imaging in proton therapy. Here, we developed Zn-doped iron oxide nanoparticles citrate-coated (IONP@Zn-cit). We assessed nanoparticle-induced cytotoxicity using MTT assays in the V79 cell line (Figure a). IONP@Zn-cit showed a dose-dependent cytotoxicity effect after 24 hours; EC50 obtained was 86.32 mg Zn/mL. Furthermore, cellular uptake was assessed as intracellular Zn after incubation with different amounts of radiolabelled nanoparticle (^67^Ga-IONP@Zn-cit). After 24 hours, V79 showed a concentration-dependent accumulation, between 1.84-2.66 pg/cell (Figure b). Finally, to evaluate the effect of the IONP@Zn-cit on cells ability to produce colonies, we performed clonogenic assays under X-ray irradiation. With non-toxic nanoparticle concentrations, V79 cells were irradiated at 1, 5, and 10 Gy. Results were analysed using the Linear Quadratic Model (Figure c) and no changes in radiosensitivity due to the nanoparticles were observed by analysing α/β ratios, mean inactivation doses (MID), enhancement factor and colony areas (Figure d). Our next aim will be to evaluate if the addition of these NPs can affect cell survival after proton irradiation and demonstrate the feasibility of using them for dose verification in proton therapy by PET imaging.

## P231 -

### Using deep learning to predict the biologically effective dose distribution on lung cancer patients

#### Bo Zhao ^1^

##### ^1^Tsinghua University, The Institute of Medical Physics and Engineering- Department of Engineering Physics, Beijing, China

To develop a biologically effective dose (BED) prediction model considering relative biological effectiveness (RBE) and patient anatomy for achieving a reliable evaluation of lung cancer patients. A database containing images and proton plans of 116 lung cancer patients was studied. Patient-specific parameters of gross tumor volume (GTV) and identified organs at risk (OARs) were extracted via Numpy and simple ITK. BED distributions of the original treatment plans were generated by Geant4 Monte Carlo simulations, based on published RBE models and radiobiological parameters of the lung cancer cell line. Then, the BED and structure maps were resampled to have a voxel resolution of 3 × 3 × 3 mm3. A developed deep learning architecture, P-B Nestnet, was adopted as the training framework. The early-stop mechanism was adopted on the validation set to avoid overfitting. The evaluation criteria of predictive accuracy contain the maximum and the mean BED of all targets, as well as BED-volume metrics. The statistical outcomes demonstrate that the P-B Nestnet model predicts BED distributions accurately. The average absolute biases of max and mean BED for GTV are 2.4% and 3.2%, respectively. For OARs, this model is capable of predicting the errors of the max and mean BED within 5.6% and 4.1%, respectively. The study developed a model capable of predicting BED distribution accurately for lung cancer patients in proton radiotherapy. The predicted BED map enables a quick intuitive evaluation of the treatment plan. It shows a potential toward cost-comparativeness of proton versus photon therapy in real clinical practice.

## P232 -

### Pencil beam scanning Proton beam therapy for moving tumors: Preliminary experience from PROMO prospective registry

#### Sham Sundar^1^, Srinivas Chilukuri^1^, Ashwathy Mathew^1^, Kartikeswar Patro^2^, Ashok Reddy^1^, Nagarjuna Burela^1^, Utpal Gaikwad^1^, Rishan Thimma^1^, Sapna Nangia^1^, Dayananda Sharma^2^, Rakesh Jalali^1^

##### ^1^Apollo Proton Cancer Centre, Radiation Oncology, Chennai, India ^2^Apollo Proton Cancer Centre, Medical Physics, Chennai, India

**Purpose**: To analyze preliminary dosimetric and clinical outcomes of patients with moving tumors treated with pencil beam scanning proton therapy(PBT) as part of a prospective registry.

**Materials and Methods**: Twenty consecutive patients with thoracic and upper abdominal tumors underwent planning CT in free breathing(FB) or breath hold(BH) based on tumor motion assessed on 4DCT scan acquired using surface tracking. BH-4DCT scans were also acquired to assess residual motion. All plans were generated using robust optimization to iCTV. Patients were treated using daily CBCT and continuous surface tracking during treatment delivery. Weekly quality assurance (QA) imaging was performed to assess dose perturbations.

**Results**: Eight patients of lung, 5 hepato-pancreatic, 4 mediastinaland 3 upper gastro-intestinal tumors were treated to median dose and dose per fraction of 55.5CGE(30.6-60CGE) and 2.25CGE(1.8-7CGE) respectively. Median volume of iCTV was 263.5cc(15-3600cc). Eleven patients were treated in FB and rest in BH. Median tumor motion in patients treated in BH and FB in x,y,z axis were 0.5,1.3,0.5cm and 0.2,0.4,0.3cm respectively; while maximum residual target motion in BH scans was 0.1,0.3 and 0.2cm. Median D98 of iCTV was 98.6% (93-100) and robust for atleast 3mm setup error and 3.5% range uncertainty. Median number of QACT's per patient was 4(2-5). Four patients required adaptive re-planning once while 3 patients required twice. All patients completed their planned dose of treatment without any grade-3 toxicities. With a median follow-up of 9 months, the local control in the treated lesions was 90%.

**Conclusion**: Image-guided PBT for moving tumors is feasible and early outcomes seem encouraging.

## P233 -

### Institutional audit of geriatric patients treated with pencil beam scanning proton beam therapy (PBS-PBT) – Toxicity and early outcomes

#### Utpal Gaikwad^1^, Sapna Nangia^2^, Srinivas Chilukuri^2^, Ashwathy Mathews^2^, Rakesh Jalali^2^

##### ^1^Apollo Proton Cancer Center- Chennai- India, Radiation Oncology, Chennai, India ^2^Apollo Proton Cancer Center, Radiation Oncology, Chennai, India

**Introduction**: Retrospective audit of 37 consecutive geriatric patients (> 65 years) treated with IG PBS-PBT, to analyze acute toxicities and early outcomes.

**Patients and Methods**: Among first 264 patients treated with PBS PBT, 37 were >/= 65 years. Their demographic data, treatment modality, toxicities and outcomes were analyzed.

Results: Thirty-seven (27 males, 10 females) patients, 35.2%(13) were between 65-70years, 32.4%(12) between 71-75years and 32.4% (12) >76years old were assessed. Two or more co-morbidities were noted in 40.5% patients, median age adjusted CCI score was 7 (5-8) and median Frailty score 5 (4-7). Head-neck and thoracic cancers constituted 24% each, brain and prostate constituted 19% each, abdominal malignancies, 9%, and breast cancers, 5%. Locally advanced cancer was noted in 59% and metastatic in 11%. Median EQD2 dose was 63GyE (45-88), median fractions 25 (6-35) and median OTT 29 days (8-51). 86.7% eligible patients received concurrent systemic therapy, 84.5% of whom completed planned dose. Grade 2 and 3 acute toxicity was noted in 19 (54.1%), and 9 (24.3%),respectively; 1 developed Grade 4 hematological toxicity.

**Results**: An unplanned treatment gap was experienced in 6(16.2%) median gap, 2 days (2-4 days). Unplanned hospitalizations were noted in 5 patients (13.5%), median stay, 3 days (2-5 days). At a median Follow-up of 10 months, LC was 86.8 %, with none having late grade 4 toxicities

**Conclusion**: Modern PBT is well tolerated in geriatric cancer patients across all sub-sites, and early results suggests better therapeutic ratio can be achieved. Benefits of PBT in this age group should be explored further.

## P234 -

### Public funding for proton beam therapy in the Australian healthcare system

#### S. Penfold^1^, M. Penniment^2^, H. Le^2^, P. Gorayski^2^, D. Page^3^

##### ^1^Australian Bragg Centre for Proton Therapy and Research, Medical Physics, Adelaide, Australia ^2^Royal Adelaide Hospital, Radiation Oncology, Adelaide, Australia ^3^Commercial and General, Health Project Management, Adelaide, Australia

Australia's first proton therapy facility, the Australian Bragg Centre for Proton Therapy and Research (ABCPTR), is currently under construction in Adelaide, South Australia. In 2020 the parent company of the ABCPTR, the South Australian Health and Medical Research Institute (SAHMRI), lodged an application for proton beam therapy in patients under 25 years of age and certain adult cancers of the head and spine, to be listed on the Australian Medicare Benefits Schedule (MBS). The MBS provides government funding to ensure that all Australians have access to evidence-based medical care in an equitable manner. Applications for MBS funding are assessed based on the evidence of safety, efficacy and cost-effectiveness of the treatment. In this presentation we will outline the application made by SAHMRI, including key published evidence and cost-utility modelling, and summarize the recommendations made by the Medical Services Advisory Committee.

## P235 -

### High-dose proton therapy offers dosimetric advantages over photon therapy for thoracic esophageal cancer treatment.

#### Sornjarod Oonsiri^1^, Sarin Kitpanit^1^, Danita Kannarunimit^1^, Chakkapong Chakkabat^1^, Chawalit Lertbutsayanukul^1^, Anussara Prayongrat^1^

##### ^1^Chulalongkorn University, Radiation Oncology, Bangkok, Thailand

**Purpose**: To compare dosimetric performances of high-dose (≥60Gy) intensity-modulated radiation therapy (IMRT) with intensity-modulated proton therapy (IMPT) using pencil beam scanning for the treatment of thoracic esophageal cancer.

**Materials and Methods**: The IMPT plans with three different beam angle configurations (figure 1) were generated on computed tomography datasets of 25 patients (upper=3, mid=12, lower=10) who were previously treated with IMRT. The prescription doses were 50-54 Gy(RBE) and 60-64 Gy(RBE), at 2 Gy(RBE) per fraction, to the low-risk and high-risk planning target volumes (PTV), respectively. For IMPT plans, robust optimization was applied. Dose-volume parameters of the target volumes and organs at risk (OARs) between IMRT and IMPT plans were compared using paired t-tests for means or Wilcoxon signed-rank tests for medians.

**Results**: Target coverage was comparable between all treatment plans. In comparison with IMRT, all IMPT plans resulted in significantly lower doses to OARs (Table 1). Beam configuration B achieved the lowest lung dose (mean = 7.0Gy(RBE) and V_20_ = 14.2Gy(RBE), p<0.001) while beam configuration C resulted in the lowest heart dose (mean = 13.1Gy(RBE), V_30_ = 17.5Gy(RBE), p<0.001) and liver dose (mean = 3.2Gy(RBE), V_30_ = 4.3Gy(RBE), p=0.005).

**Conclusion**: The IMPT plans resulted in a good target coverage and significantly spared the OARs, as compared with IMRT plan. IMPT beam configuration B or C is recommended in IMPT planning for thoracic esophageal cancer, however, the selection between these two plans depends on tumor location.

## P236 -

### Proton therapy center of Kutaisi international university, Georgia

#### Revaz Shanidze^1^, Mariam Abuladze^1^, Abesalom Iashvili^1^, Levan Ivanishvili^1^, Akaki Lomia^1^, Tengiz Mdzinarishvili^1^, Mariam Osepashvili^1^, Alexander Tevzadze^1^, Vakhtang Tsagareli^1^, Edisher Tskhadadze^1^

##### ^1^Kutaisi International University, Hadron therapy center, Kutaisi, Georgia

New Hadron Therapy Center (HTC) at the Kutaisi International University in Georgia, will be equipped with 2 superconducting synchrocyclotrons IBA S2C2, providing maximum energy of accelerated protons 230 MeV. The cyclotrons are currently in production at IBA (Ion Beam Applications S.A. , Louvain-la-Neuve, Belgium). One of these accelerators is part of IBA single gantry Proteus©ONE system for the proton therapy, while the other one is the main device for a new research infrastructure at Kutaisi International University in Georgia. The HTC is funded by the International Charity Foundation Cartu, the largest charity foundation in Georgia. Opening of HTC is planned in the 2024. Research with the proton beam in 70-230 MeV energy range is foreseen in multiple disciplines, including basic and applied nuclear physics, radiation biology and medicine as well as material sciences and detector development and testing. As the only cyclotron-based research facility in Georgia and South Caucasus, HTC will become a research hub for international projects. The projects at HTC will be selected by International Advisory Committee (IAC). The Status of the HTC at Kutaisi International University will be presented in the talk.

## P238 -

### Assessing concordance between patient-reported and investigator-reported CTCAE for acute urinary and bowel toxicity after proton beam therapy for prostate cancer

#### R. Kowalchuk^1^, D. Hillman^2^, T. Daniels^3^, C. Vargas^4^, J.C. Rwigema^4^, W. Wong^4^, A. Dueck^5^, C.R. Choo^1^

##### ^1^Mayo Clinic, Radiation Oncology, Rochester, USA ^2^Mayo Clinic, Statistics, Rochester, USA ^3^NYU Langone, Radiation Oncology, New York, USA ^4^Mayo Clinic, Radiation Oncology, Phoenix, USA ^5^Mayo Clinic, Statistics, Phoenix, USA

**Purpose**: We report acute patient-reported outcomes using CTCAE (PRO-CTCAE) of proton beam radiotherapy for high or unfavorable-intermediate risk prostate cancer in a prospective clinical trial. PRO-CTCAE were correlated with investigator reported-CTCAE (IR-CTCAE) to assess the degree of concordance.

**Methods and materials**: 11 PRO-CTCAE questions assessed GI, GU, or erectile function side effects. The correlation scheme between PRO-CTCAE and IR-CTCAE was independently developed by two physicians. Analyses of PRO-CTCAE and IR-CTCAE were conducted using both descriptive terms and the converted grade scores. The Kappa statistic described the degree of concordance.

**Results**: 55 patients were included (Table 1). IR-CTCAE underestimated diarrhea compared to PRO-CTCAE at the end of treatment (EOT), with a 28% rate of underestimation (11% by ≥ 2 toxicity grades). Similarly, urinary tract pain was underestimated in 45% of cases (17% by ≥ 2 grades) at EOT. Differences were less pronounced at baseline or 3 months after radiotherapy. The incidence of urinary urgency and frequency tended be overestimated prior to treatment (36% and 24%, respectively) but underestimated at EOT (35% and 31%, respectively). The degree of interference with daily activities was consistently overestimated by investigators (45%-85%). Finally, erectile dysfunction showed a 36-56% rate of discordance by ≥ 2 toxicity grades (Table 2).

**Conclusions**: We present a novel correlation between IR-CTCAE and PRO-CTCAE in the setting of proton therapy for prostate cancer. There was low agreement, with physicians underestimating the frequency and severity of urinary symptoms and diarrhea at the end of treatment. Continued use of PROs should be strongly encouraged.

## P239 -

### Clinical implementation of a 6D treatment chair for fixed ion beam lines

#### Jiayao Sun^1^, Lin Kong^1^, Zhi Chen^1^, Dan You^1^, Xiaodong Wu^1^, Yinxiangzi Sheng^1^

##### ^1^Shanghai Proton And Heavy Ion Center, medical physics, Shanghai, China

**Purpose:** To verify the practicality and validity of a treatment chair with six degrees of freedom (6DTC) through demonstrating the efficacy of the workflow in clinical settings and analyzing the obtained technical data during the use of the 6DTC.

**Methods and Materials:** A clinical study was designed and conducted to test the clinical treatment workflow and the safety of the 6DTC. Based on the demonstrated dosimetric advantages, fifteen patients with head and neck tumors were selected and treated with the 6DTC. The positional error at the first beam position (PE-B1) and the second beam position (PE-B2) were analyzed and compared with the results from daily quality assurance (QA) procedures of the 6DTC and imaging system performed each day before clinical treatment.

**Results:** The QA results showed sub-millimeter mechanical accuracy of the 6DTC over the course of the clinical study. For 150 patient treatment fractions, the mean deviations between PE-B1 and PE-B2 were 0.13mm (SD 0.88mm), 0.25mm (SD 1.17mm), -0.57mm (SD 0.85mm), 0.02° (SD 0.35°), 0.00° (SD 0.37°), and -0.02° (SD 0.37°) in the x, y, z (translational), and u, v, w (rotational) directions, respectively. The deviation between PE-B1 and PE-B2 for fifteen patients was shown in Figure 1.

**Conclusions:** The performance stability of the 6DTC was satisfactory. The position accuracy in an upright posture with the 6DTC was verified and found adequate for clinical implementation.

## P241 -

### 3D dose measurement for commissioning and pre-treatment QA

#### Juan Perez^1^, Alejandro Mazal^1^, Juan Antonio Vera Sanchez^1^, Juan Castro Novais^1^, Carme Ares^1^, Raymond Miralbell^1^, Amaia Ilundain^1^, Lina Mayorga^1^, Isabel Lorenzo Villanueva^1^, Elisabet Canals de las Casas^1^

##### ^1^Centro de Protonterapia Quirónsalud, Radiation Oncology, Pozuelo de Alarcón, Spain

**Objective**: To measure 3D dose distribution of PBS beams to be used during commissioning and patient specific pre-treatment QA.

**Method**: Using a 2D array of paralell plane ion chambers (MatriXX One, IBA Dosimetry) a series of PBS beams has been measured for a system based on a cryogenic synchrocyclotron and a compact gantry (Proteus One, IBA). The measurement set involves fields for TPS validation, verification of ranges in heterogeneous media (including geometrical phantoms and biological samples) and patient specific pre-treatment QA. 3D dose distribution is reconstructed by stacking 2D planes measured using MatriXX with isocenter at RW3 phantom surface entrance and gantry at 0°. Every measured plane requires a delivery of the beam under verification, a movement of the couch and change in RW3 slices over the detector. The same set of measurement can be performed with DigiPhant PT (IBA Dosimetry) and gantry at 90°. 3D measurement is compared with calculated dose distribution using 3D gamma analysis 2%-2mm (local dose) and statistics of calculated-measured range (R90, R80, R50 and R20) is extracted along beam area.

**Results**: Average percentage of points fulfilling gamma < 1 is above 92%. Average range difference is below 1mm homogeneous and heterogeneous geometric phantoms. In phantoms made of biological samples mean range error is 1.5 mm, with maximum differences up to 2.1 mm.

**Conclusions**: 3D dose measurements are feasible, but it's a time consuming task in RW3. The use of DigiPhant PT helps to perform this verification very efficiently and with a high level of accuracy.

## P242 -

### Beam angle comparison for distal esophageal carcinoma patients treated with intensity-modulated proton therapy

#### Hongying Feng^1^, Terence Sio^1^, William Rule^1^, Ronik Bhangoo^1^, Pedro Lara^1^, Christopher Patrick^1^, Shawn Korte^1^, Mirek Fatyga^1^, William Wong^1^, Steven Schild^1^, Jonathan Ashman^1^, Wei Liu^1^

##### ^1^Mayo Clinic Arizona, Department of Radiation Oncology, Phoenix, USA

**Introduction:** In this work, we compared the dosimetric performances of IMPT plans with two different beam-angle configurations (the Right-Left oblique posterior beams and the Superior-Inferior oblique posterior beams, i.e. Group R-L and Group S-I) for the treatment of distal esophageal carcinoma in the presence of uncertainties and interplay effect.

**Method:** Twenty patients treated by IMPT at our institution were retrospectively selected, evenly divided into two groups with their clinical target volumes (CTVs—high and low dose levels) and respiratory motion amplitudes matched. Dose-volume-histogram (DVH) indices and the corresponding DVH bandwidths were calculated to quantify plan quality and robustness respectively. Interplay effect was evaluated using four-dimensional (4D) dynamic dose calculation. Normal tissue complication probability (NTCP) for heart, liver, and lung was calculated, respectively, to estimate the clinical outcomes. Wilcoxon signed-rank test was used for statistical analysis.

**Results:** Compared with plans in Group R-L, plans in Group S-I resulted in significantly better organs-at-risk (OAR) protection except a slightly higher but clinically acceptable spinal cord D_max_ without (Fig. 1) and with interplay effect (Fig. 2) considered. Similar plan robustness was observed between the two groups. NTCP for liver was significantly better in Group S-I.

**Conclusions:** Our study showed that plans in Group S-I would better spare adjacent OARs lateral to the target and are more resilient to interplay effect by irradiating in the S-I direction that usually has the largest respiratory motion. The results supported the routine use of the S-I oblique posterior beams for the treatments of distal esophageal carcinoma by IMPT.

